# Non-Gaussian Distributions Affect Identification of Expression Patterns, Functional Annotation, and Prospective Classification in Human Cancer Genomes

**DOI:** 10.1371/journal.pone.0046935

**Published:** 2012-10-31

**Authors:** Nicholas F. Marko, Robert J. Weil

**Affiliations:** 1 Cancer Research United Kingdom Cambridge Research Institute and Department of Applied Mathematics and Theoretical Physics, Cambridge University, Cambridge, United Kingdom; 2 Brain Tumor and Neuro-Oncology Center, Cleveland Clinic, Cleveland, Ohio, United States of America; University of North Carolina School of Medicine, United States of America

## Abstract

**Introduction:**

Gene expression data is often assumed to be normally-distributed, but this assumption has not been tested rigorously. We investigate the distribution of expression data in human cancer genomes and study the implications of deviations from the normal distribution for translational molecular oncology research.

**Methods:**

We conducted a central moments analysis of five cancer genomes and performed empiric distribution fitting to examine the true distribution of expression data both on the complete-experiment and on the individual-gene levels. We used a variety of parametric and nonparametric methods to test the effects of deviations from normality on gene calling, functional annotation, and prospective molecular classification using a sixth cancer genome.

**Results:**

Central moments analyses reveal statistically-significant deviations from normality in all of the analyzed cancer genomes. We observe as much as 37% variability in gene calling, 39% variability in functional annotation, and 30% variability in prospective, molecular tumor subclassification associated with this effect.

**Conclusions:**

Cancer gene expression profiles are not normally-distributed, either on the complete-experiment or on the individual-gene level. Instead, they exhibit complex, heavy-tailed distributions characterized by statistically-significant skewness and kurtosis. The non-Gaussian distribution of this data affects identification of differentially-expressed genes, functional annotation, and prospective molecular classification. These effects may be reduced in some circumstances, although not completely eliminated, by using nonparametric analytics. This analysis highlights two unreliable assumptions of translational cancer gene expression analysis: that “small” departures from normality in the expression data distributions are analytically-insignificant and that “robust” gene-calling algorithms can fully compensate for these effects.

## Introduction

### Background

Microarray-based assays of gene expression have become a mainstay of basic and translational cancer research. A significant number of modern investigations rely on these tools to inform hypothesis generation [1], for pathway analysis [2], [3], for pharmacogenomics and drug discovery [4], and for developing molecular-based disease classification strategies [5], [6]. Additionally, gene expression data are becoming progressively more important for informing clinical diagnosis and patient management [7], [8], and microarray-based genomic profiles are now being used to guide patient enrollment and stratification in large-scale clinical trials [9], [10].

Against this backdrop, the importance of accurate interpretation of microarray results and the significant consequences of systematic analytic errors becomes apparent. In the early days of microarray analysis, high experimental costs and significant technical variability limited the available information with which comprehensive analyses of the practical effects of subtle biases in microarray data or in its interpretation could be studied [11]. This, in turn, necessitated that certain mathematical and biological assumptions be made [12], [13], and the lack of adequate data precluded in-depth investigation of the validity of these assumptions.

### The Assumption of Normality in Two Related Types of Expression Datasets

One common assumption is that data from microarray-based genome expression analyses conform to a standard Gaussian (normal) distribution. This assumption is rarely explicit but rather is most commonly made implicitly when investigators apply analytic algorithms predicated upon the Gaussian assumption. Distribution-related assumptions are relevant to at least two, distinct sets of expression data generated in microarray analyses, and the normality assumption has been variably (often implicitly) applied to both [12]–[15].

The first dataset to which distribution is relevant comprises the complete set of individual expression values across all genes and all samples in a given experiment. For example, in a study examining the expression of 25,000 genes in 100 tumors, this is the set of all 2.5 million gene expression values. The distribution of this composite dataset may be particularly relevant to downstream clustering and class discrimination analyses, as many of these algorithms are typically applied to the entire dataset as a whole. When algorithms predicated upon a standard Gaussian distribution are used, the normal assumption is implicitly introduced.

The second dataset to which distribution is relevant is the dataset comprising the individual expression values for a single gene across the entire range of experimental samples. Continuing the previous example, this experiment would generate 25,000 such datasets, each with 100 data points. The distribution of these 100 data points may be particularly relevant to studies that examine the consistency of behavior of a specific gene in a specific tumor type or analyze the pattern of its change across a range of “classes” or “grades” of a specific tumor. Here the distribution may provide a useful description of the behavior of this single gene across multiple independent samples, but the normal assumption may be implicitly introduced if algorithms used to analyze the behavior of this gene are predicated upon a standard Gaussian distribution.

The assumption of normality has been explicitly investigated in gene expression analysis, although to a limited degree. While it initially appeared to have both theoretical [16] and empiric support [11], [17], more recent analyses have suggested the possibility of non-Gaussian distributions for gene expression data [18]–[21]. At present, however, most of these observations are derived from simulated [19], [21], heterogeneous [20], [21], or non-clinical datasets [18]–[21].

### Significance

The possibility that gene expression data violate the normality assumption may be of considerable significance to clinical and translational investigators. Most current and proposed medical applications of microarray expression data are derived from analyses predicated upon this assumption, many of which have relied upon parametric statistics for gene calling and class discovery [6]–[8]. Translational oncologists are among the most avid consumers of microarray data and the most likely to propose its clinical application, so a logical place to begin an investigation of the magnitude, extent, and clinical implications of non-Gaussian distributions in gene expression data is with large, publicly-available cancer genome databases [22], [23]. Notwithstanding, this issue is fundamental to the current analytic paradigm for gene expression data in general, and we anticipate that the findings of this investigation will have significance beyond the sphere of translational molecular oncology.

**Figure 1:**
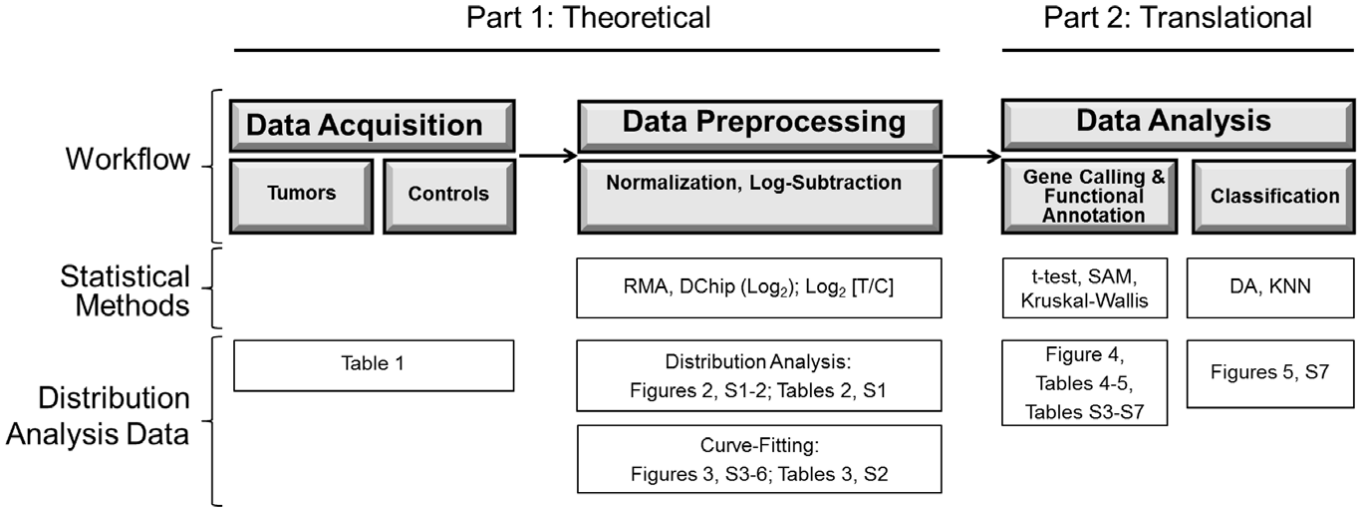
Overview of Analytic Workflow. The flow diagram depicts typical microarray analysis workflow (top section), the statistical methods used at each step (middle section), and the corresponding tables and figures in this manuscript that present analyses at each level (bottom section).

The present investigation has two objectives and has been structured in two parts: the first is theoretical – to study the distributions of cancer gene expression data – both at the individual gene and at the complete dataset level – and to assess the extent to which these deviate from normality. This provides the foundation for the second, translational objective: to study the implications of non-Gaussian gene expression distributions on clinically-oriented genomic analyses. The experimental model has been deliberately designed to recapitulate faithfully the workflow of a typical, translational pipeline for gene expression analysis (Figure 1).

## Results

### Distribution Analysis – Complete Datasets

**Figure 2:**
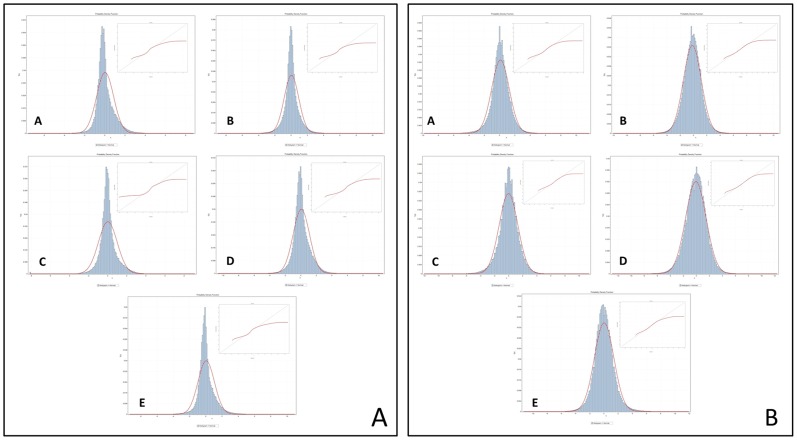
Cancer gene expression datasets are not normally-distributed. The source data for these graphs are the Log_2_-subtracted datasets. All bin widths have been set to 200 to improve visualization. Red curves represent the best-fit normal distribution. The primary image gives the histogram with the superimposed theoretical normal curve. The inset presents the quantile-quantile (QQ) plot, where deviation from the line (y = x, black) illustrates deviation of the empiric from the theoretical normal distribution. Left panel shows data normalized with the RMA method. Right panel shows data normalized with the DChip method. A: Brain; B: Breast; C: Colon; D: Gastric; E: Ovarian.

We first examined the distributions of the complete set of individual expression values across all genes and all samples in each of five experiments (the first type of data set described in the introduction). Table 1 summarizes the results of the central moments analysis of five, large-scale (n = 180, each) human cancer genomes, which was performed after normalization with either the robust multichip average (RMA) [24] or the DChip [25] methods. These data demonstrate that, while the means and standard deviations suggest approximate normality (μ range: −0.18–0.10; σ range: 0.84–1.58), the third and fourth central moments depart from normality in a statistically-significant manner. Fisher's indices of skewness and kurtosis, which are considered significant at α<0.05 when they exceed ±1.96, are >100 for all samples. Additionally, the *F*-test of the variance demonstrates statistically-significant departures from normality for all samples (Tables 1, S1). All five cancer gene expression distributions therefore depart significantly from the normal distribution. This is further supported by the results of the one-way and two-way KS tests, which demonstrate significant departures from normality for all of the datasets. Moreover, the findings of the central moments analysis suggest that these distributions have slight but significant skewness, are markedly kurtotic, and are heavy-tailed (Figure 2). Similar results from data normalized using both the RMA [24] and the DChip method [25] suggest that this departure from normality is unlikely to be a function of the normalization algorithm, and analysis of both Log_2_-transformed and Log_2_-subtracted data suggests that it is not related to Log subtraction (Tables 1, S1; Figures S1, S2).

**Table 1:** Central Moments Analysis.

		Log2 Subtracted			
		RMA		DChip	
		Statistic	Test Statistic	Statistic	Test Statistic
**Brain**					
	Patients (features)	180 (9,841,500)		180 (9,841,500)	
First Moment	Mean	0.020	<0.0001, <0.0001*	0.104	<0.0001, <0.0001*
Second Central Moment	Variance	0.710	<.0001 [0.706,0.710]**	1.336	<.0001 [1.328,1.336]**
	Standard Deviation	0.843		1.156	
Third Central Moment	Skew	0.836	<0.0001 (1.01×10^3^)#	−0.110	<0.001 (−140.7)#
Fourth Central Moment	Excess Kurtosis	3.912	0.002 (2.5×10^3^)†	2.429	0.002 (1.6×10^3^)†
One-Sample Kolmogorov-Smirnov		0.118	<.0001 (±0.0004)‡	0.053	<.0001 (±0.0004)‡
Two-Sample Kolmogorov-Smirnov		0.118	<.0001φ	0.053	<.0001φ
**Breast**					
	Patients (features)	180 (9,841,500)		180 (9,841,500)	
First Moment	Mean	0.033	<0.0001, <0.0001*	−0.180	<0.0001, <0.0001*
Second Central Moment	Variance	0.813	<.0001 [0.809,0.813]**	1.818	<.0001 [1.808,1.818]**
	Standard Deviation	0.902		1.349	
Third Central Moment	Skew	0.223	<0.0001 (286.2)#	−0.280	<0.001 (−35.9)#
Fourth Central Moment	Excess Kurtosis	5.268	0.002 (3.4×10^3^)†	1.531	0.002 (980.3)†
One-Sample Kolmogorov-Smirnov		0.115	<.0001 (±0.0004)‡	0.090	<.0001 (±0.0004)‡
Two-Sample Kolmogorov-Smirnov		0.115	<.0001φ	0.090	<.0001φ
**Colon**					
	Patients (features)	180 (9,841,500)		180 (9,841,500)	
First Moment	Mean	0.002	0.105, 0.012*	−0.044	<0.0001, <0.0001*
Second Central Moment	Variance	0.991	<.0001 [0.986,0.991]**	1.778	<.0001 [1.768,1.779]**
	Standard Deviation	0.996		1.334	
Third Central Moment	Skew	−1.640	<0.0001 (−2.1×10^3^)#	−0.278	<0.001 (−356.1)#
Fourth Central Moment	Excess Kurtosis	17.590	0.002 (1.1×10^4^)†	1.622	0.002 (1.1×10^3^)†
One-Sample Kolmogorov-Smirnov		0.112	<.0001 (±0.0004)‡	0.050	<.0001 (±0.0004)‡
Two-Sample Kolmogorov-Smirnov		0.112	<.0001φ	0.050	<.0001φ
**Gastric**					
	Patients (features)	180 (9,841,500)		180 (9,841,500)	
First Moment	Mean	0.052	<0.0001, <0.0001*	−0.094	<0.0001, <0.0001*
Second Central Moment	Variance	1.120	<.0001 [1.113,1.120]**	2.482	<.0001 [2.468,2.483]**
	Standard Deviation	1.058		1.575	
Third Central Moment	Skew	0.228	<0.0001 (369.2)#	−0.177	<0.0001 (−220.9)#
Fourth Central Moment	Excess Kurtosis	3.981	0.002 (2.6×10^3^)†	1.374	0.002 (879.6)†
One-Sample Kolmogorov-Smirnov		0.066	<.0001 (±0.0004)‡	0.107	<.0001 (±0.0004)‡
Two-Sample Kolmogorov-Smirnov		0.066	<.0001φ	0.017	<.0001φ
**Ovarian**					
	Patients (features)	180 (9,841,500)		180 (9,841,500)	
First Moment	Mean	0.036	<0.0001, <0.0001*	0.009	<0.0001, <0.0001*
Second Central Moment	Variance	1.027	<.0001 [1.022,1.028]**	1.889	<.0001 [1.878,1.890]**
	Standard Deviation	1.014		1.374	
Third Central Moment	Skew	0.827	<0.001 (1.06×10^3^)#	0.350	<0.0007 (447.8)#
Fourth Central Moment	Excess Kurtosis	5.692	0.002 (3.6×10^3^)†	2.320	0.002 (1.5×103)†
One-Sample Kolmogorov-Smirnov		0.099	<.0001 (±0.0004)‡	0.048	<.0001 (±0.0004)‡
Two-Sample Kolmogorov-Smirnov		0.099	<.0001φ	0.048	<.0001φ

Results of the central moments analysis for each of the five tumor types, using each of two normalization methods. RMA = Robust Multichip Average.

* = t-test 2 class unpaired, Wilcoxon Rank-Sum vs. simulated.

** = *f*-test.

# = Standard Error of Skewness (Fisher's Skewness Index).

† = Standard Error of Kurtosis (Fisher's Kurtosis Index).

‡ = p-value [95% CI].

φ = p-value.

These findings are not necessarily surprising, as neither of the normalization methods nor the process of log-transformation are specifically intended to produce normality; however, this analysis demonstrates using multiple expression datasets that none of these transformations are sufficient to produce Gaussian data. Accordingly, it cannot be safely assumed that data that have been “normalized” using any of these methods actually conform to a “normal” (standard Gaussian) distribution.

### Distribution Analysis – Individual Genes

**Figure 3:**
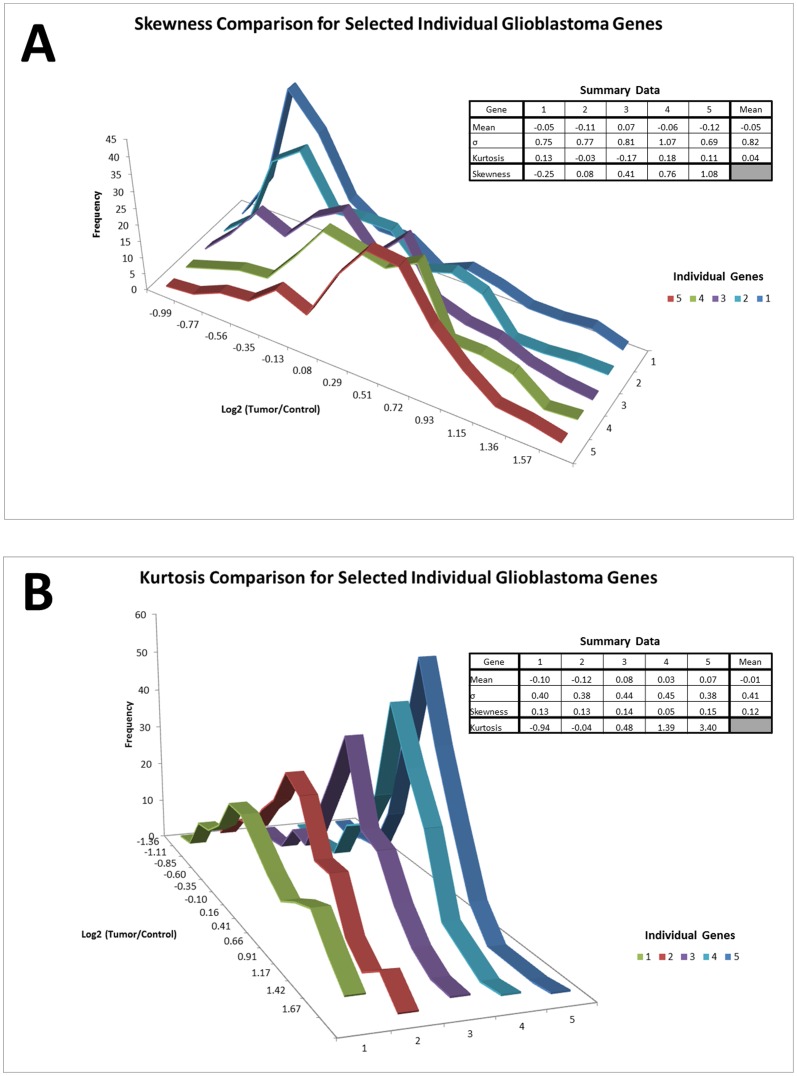
Single-Gene Expression Distributions are not Gaussian. These graphs illustrate the wide range of potential skewness (A) and kurtosis (B) that exist in the expression distributions of individual genes comprising the cancer expression datasets. This refutes the assumption that the expression data for individual genes follow an approximately Gaussian distribution around the gene's mean expression level. Data for these graphs was taken from the log_2_-subtracted, RMA-normalized glioblastoma expression data. For the skewness comparison, five genes with comparable means, standard deviations, and kurtosis were selected from subsets of genes representing approximately the 10^th^, 25^th^, 50^th^, 75^th^ and 90^th^ percentiles for per-gene skewness contained in the dataset. Similarly, for the kurtosis comparison, five genes with comparable means, standard deviations, and skewness were selected from subsets of genes representing approximately the 10^th^, 25^th^, 50^th^, 75^th^ and 90^th^ percentiles for per-gene kurtosis contained in the dataset. The identities of the genes are not germane for comparative purposes.

We also examined the data distributions of individual genes across the 180 samples of each of the 5 cancer data sets. Many investigators examining data from an experiment containing microarrays of multiple, similar tumors may assume that an “overexpressed” gene would exhibit a Gaussian distribution centered around a positive mean value, an “underexpressed” gene will have a similar distribution around a negative value, and a gene whose expression is unchanged will have a Gaussian distribution centered around zero. Our analysis, however, demonstrates that variable degrees of skewness and kurtosis as well as marked deviations from unity among the standard deviations are characteristic of the expression distributions for individual genes. Table 2 summarizes the results of this analysis, and Figure 3 gives an illustrative example of this effect by plotting the distributions selected genes from the brain tumor (glioblastoma) data set.

**Table 2:** Single Gene Distribution Variability.

		Σ					Skewness					Kurtosis				
		mean	median	min	Max	σ	Mean	median	min	max	σ	mean	median	min	max	σ
**Brain**	RMA	0.37	0.28	0.04	3.32	0.27	0.53	0.39	−7.79	12.39	0.90	1.82	0.47	−1.81	161.65	6.30
	DChip	0.58	0.48	0.00	3.72	0.33	−0.07	−0.12	−13.42	13.42	0.97	1.47	0.37	−2.02	180.00	6.72
**Breast**	RMA	0.47	0.40	0.08	3.71	0.32	0.76	0.49	−5.66	12.35	1.20	3.20	0.73	−1.64	160.94	9.79
	DChip	0.78	0.72	0.08	3.83	0.32	−0.15	−0.19	−5.69	12.20	0.64	1.00	0.49	−1.67	156.34	2.29
**Colon**	RMA	0.35	0.29	0.05	2.75	0.21	0.40	0.27	−4.93	12.71	0.89	1.80	0.68	−1.73	166.98	5.62
	DChip	0.69	0.59	0.03	3.89	0.36	−0.32	−0.31	−6.60	13.42	0.89	1.50	0.57	−1.67	180.00	4.52
**Gastric**	RMA	0.53	0.43	0.09	4.00	0.36	0.84	0.84	−6.83	12.44	1.27	3.67	1.90	−1.73	162.49	7.02
	DChip	0.89	0.80	0.10	4.22	0.40	−0.32	−0.34	−7.52	13.42	0.82	1.77	1.01	−1.82	180.00	3.33
**Ovarian**	RMA	0.49	0.44	0.09	3.04	0.30	0.81	0.48	−6.05	12.35	1.52	4.84	1.29	−1.57	160.99	10.69
	DChip	0.75	0.69	0.10	3.48	0.29	−0.43	−0.36	−6.75	7.10	0.92	2.32	0.92	−1.48	78.97	4.30

These data show deviation from the parameters of a Gaussian distribution for the standard deviations (σ), skewness, and kurtosis of the expression distributions of individual genes comprising the cancer expression datasets. If the assumption that individual gene expression distributions are Gaussian were correct, then the mean σ should approximate 1, the mean skewness should approximate 0, and the mean kurtosis should approximate 3. The deviations from these theoretical parameters exhibited by the individual gene expression distributions in all five cancer datasets refute the Gaussian assumption for individual genes.

### Curve Fitting

**Figure 4:**
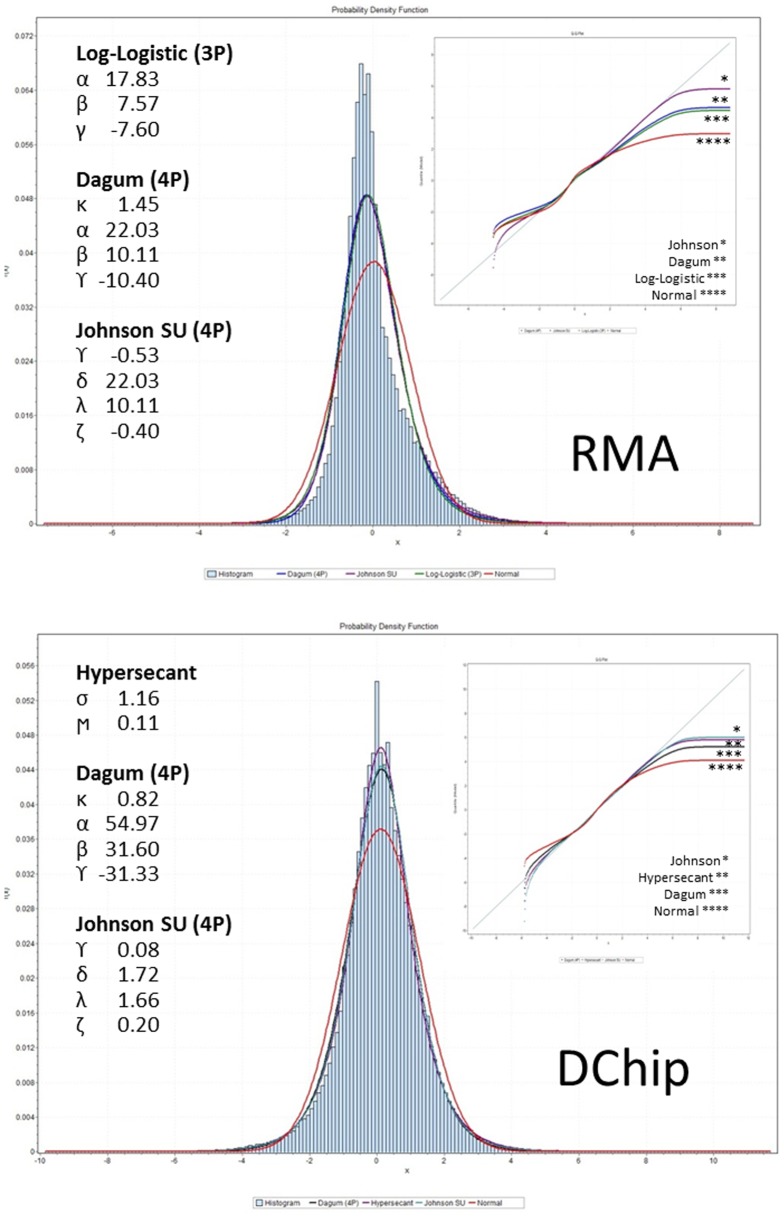
Distribution Fitting. Distribution fitting for the brain cancer dataset for RMA (top) and DChip (bottom) normalized data. The three best-fit curves are superimposed on the histogram, and the normal distribution curve is included for comparison. The specific parameters for the best-fit distributions are given. The inset displays the quantile-quantile (QQ) plot for the best-fit and normal distributions. These charts demonstrate that multiparameter distributions capable of modeling skewness and kurtosis better characterize the data than the standard Gaussian (normal) distribution. Similar graphs for additional tumor types are given in figures S2, S3, S4, S5.

Empiric curve fitting was used to further investigate the actual morphology of the cancer gene expression distributions (Table 3; Figures 4, S3, S4, S5, S6). This analysis suggests that complex, multi-parameter distributions are required to more accurately model the expression data distributions. In general, the best-fit distributions were those that are parameterized to model skewness, kurtosis, and heavy tails. These include multi-parameter distributions related to the β-prime (Pearson VI, capable of modeling skewness) (e.g. Log-logistic, Dagum, Burr), kurtotic distributions (e.g. hyperbolic-secant), and the versatile, 4-parameter Johnson SU [26].

**Table 3:** Empiric Distribution Fitting.

Tumor	RMA Normalized				DChip Normalized			
	Best-Fit Distributions	Kolmogorov-Smirnov			Best-Fit Distributions	Kolmogorov-Smirnov		
		α	Critical Value	Test Statistic		α	Critical Value	Test Statistic
Brain	Log-Logistic (3P)	0.05	0.0292	0.0595	Hyperbolic Secant	0.05	0.0319	0.0135
	Dagum (4P)	0.05	0.0292	0.0595	Dagum (4P)	0.05	0.0319	0.02
	Johnson SU	0.05	0.0292	0.0662	Johnson SU	0.05	0.0319	0.0203
Breast	Johnson SU	0.05	0.0255	0.0518	Burr (4P)	0.05	0.0337	0.0067
	Dagum (4P)	0.05	0.0255	0.0544	Johnson SU	0.05	0.0337	0.0095
	Log-Logistic (3P)	0.05	0.0255	0.0548	Hyperbolic Secant	0.05	0.0337	0.0216
Colon	Laplace	0.05	0.0274	0.0563	Dagum (4P)	0.05	0.0332	0.0141
	Hyperbolic Secant	0.05	0.0274	0.0828	Johnson SU	0.05	0.0332	0.0182
	Johnson SU	0.05	0.0274	0.1133	Hyperbolic Secant	0.05	0.0332	0.0183
Gastric	Log-Logistic (3P)	0.05	0.029	0.0498	Johnson SU	0.05	0.0366	0.0083
	Dagum (4P)	0.05	0.029	0.0505	Logistic	0.05	0.0366	0.017
	Johnson SU	0.05	0.029	0.5831	Hyperbolic Secant	0.05	0.0366	0.0308
Ovarian	Log-Logistic (3P)	0.05	0.0274	0.6245	Johnson SU	0.05	0.0343	0.0051
	Dagum (4P)	0.05	0.0274	0.0637	Dagum (4P)	0.05	0.0343	0.0116
	Johnson SU	0.05	0.0274	0.0646	Hyperbolic Secant	0.05	0.0343	0.0239

Results of the empiric distribution fitting for each of the five cancer genomes normalized using each of two normalization methods. RMA = Robust Multichip Average. The top three distributions in each category appear in the order of their overall goodness of fit.

While these distributions fit the data more accurately than the normal distribution, KS testing indicates that they are imperfect fits (Table 3). Moreover, there is no single distribution that is clearly superior for modeling all sets of expression data. Overall, this analysis confirms the significant departures from normality associated with the cancer genome expression data and demonstrates the complex nature of the underlying expression distributions.

### Gene Calling & Functional Annotation

Up to this point the analysis has been focused on investigating the actual distributions of gene expression datasets and comparing these to a theoretical, normal distribution. This analysis has demonstrated that human cancer gene expression data is not normally-distributed, either on the experiment or on the single-gene level. An appropriate next question would be whether these deviations from normality affect commonly-performed gene expression analytics, including molecular classification, gene calling, and functional annotation.

**Figure 5:**
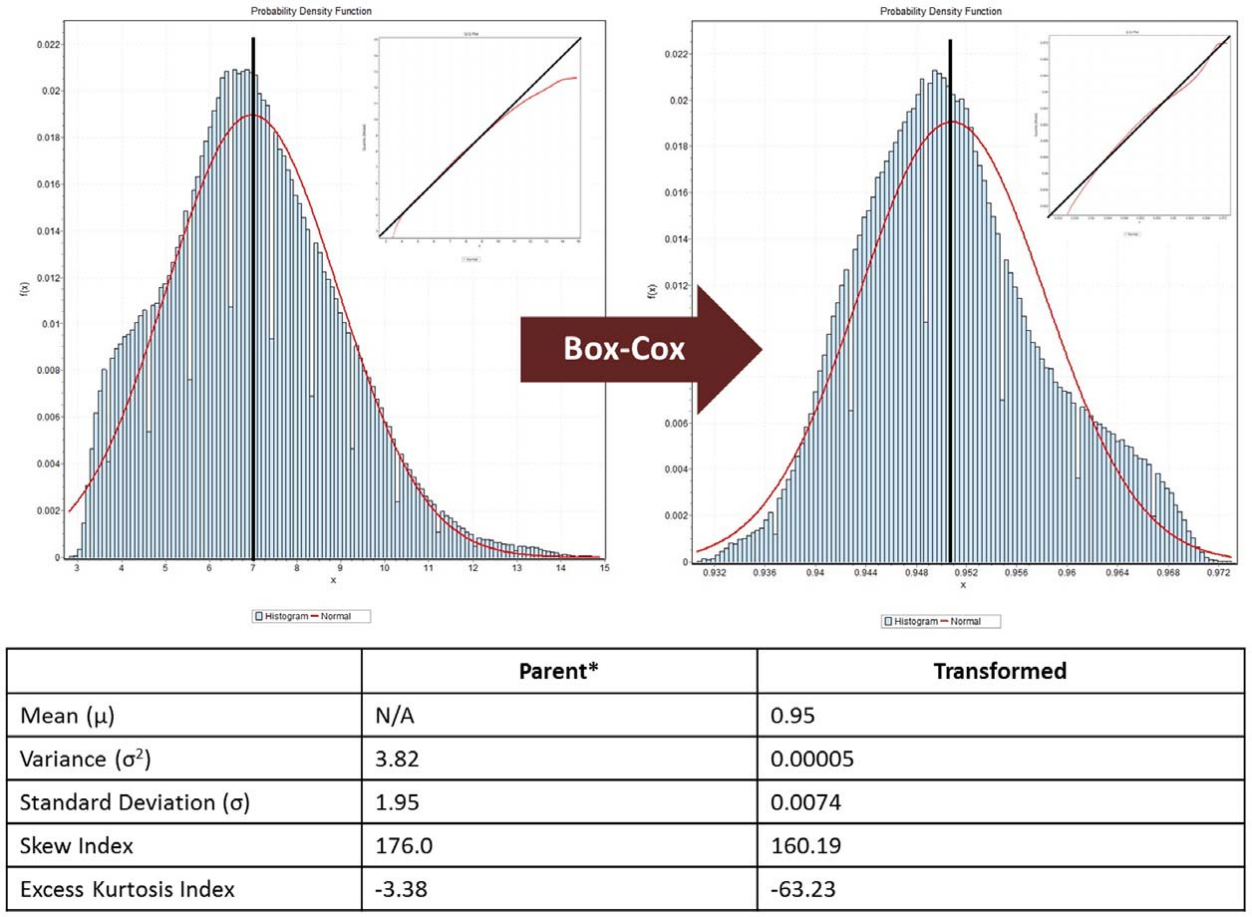
Distribution Transformation. A Box-Cox transformation applied to the low-grade glioma dataset (left) results in a distribution that more closely approximates a normal distribution (right). Note that the parent distribution was recentered to a zero mean to compensate for the default mean of the Robust Multichip Normalization output of 7. This transformed distribution was then used to analyze distribution-dependent effects on identification of differentially-expressed genes, functional annotation, and prospective molecular classification.

To investigate this question, we performed an analysis of a gene expression dataset from 23 low-grade gliomas (LGG), including a unique subset of eleven tumors with intact chromosomes 1p and 19q (arbitrarily designated *Class 1*) and another subset of eight oligodendrogliomas with chromosome 1p/19q codeletions [5], [27] (arbitrarily designated *Class 2*), was used to study the effects of the data distribution on identification genes that are differentially-expressed between known tumor subsets. This was accomplished by applying a uniform transform (Box-Cox [28]) to the expression dataset to improve the normality of the data distribution and then comparing the results of gene calling algorithms applied to the parent and transformed datasets (Figure 5). In this way only the shape of the distribution has changed, and the null hypothesis is that this transformation should have no effect on gene calling if the methods are sufficiently “robust” to distribution morphology or are truly “distribution-independent.”

The two-sided student's *t*-test with a standard Bonferroni correction (*p*<0.01), identified 50 differentially-expressed genes between *Class 1* and *Class 2* using the parent distribution and 55 using the transformed distribution (9.1% difference). Forty-nine (49) of 56 total differentially-expressed genes were common to both lists (87.5%), while 7 were uniquely identified in only one of the two lists (12.5%) (Tables 4A, S3).

**Table 4:** Gene Calling and Functional Annotation.

A	SAM^1^		Kruskal-Wallis^2^		T-Test^1^		LIMMA^4^	
	Parent	Transformed	Parent	Transformed	Parent	Transformed	Parent	Transformed
**Number of Genes with Statistically-Significant Differential Expression between Class 1 and Class 2**	759	478	1801	1800	50	55	2866	2981
**Total number of unique genes identified (present in either list)**	760		1801		56		3047	
**Genes Apperaing only in One List**	282	1	1	0	1	6	156	181
**Total Genes Appearing in Only One List (%)**	283 (37.2)		1 (0.1)		7 (12.5)		337 (11.1)	
**Genes Common to Both Lists (%)**	477 (62.8)		1800 (99.9)		49 (87.5)		1800 (99.9)	

B	SAM^1^		Kruskal-Wallis^2^					
	Parent	Transformed	Parent	Transformed				
**Number of Enriched Terms**	39	35	49	49				
**Total Number of Enriched Terms**	46		49					
**Terms Appearing only on one list**	11	7	0	0				
**Total Terms Appearing in Only One List (%)**	24 (39.1)		0 (0.0)					
**Terms Common to Both Lists (%)**	28 (60.9)		49 (100.0)					

**A:** Gene calling. The number of differentially-expressed genes identified by each of three methods (columns) in the low-grade glioma data set. SAM = Significance Analysis of Microarrays.

**B:** Differences in Functional Annotations. The number of GO and KEGG terms for which differential enrichment was identified in lists of differentially-expressed genes generated by each of two methods (columns). SAM = Significance Analysis of Microarrays.

1 = Two-class unpaired, false-discovery rate = 0.00.

2 = Two-class unpaired, Benjamini-Hochberg corrected, false-discovery rate  = 0.05.

3 = Two-sided, p = 0.01, Standard Bonferroni Correction.

4 = Two-class, α = 0.05.

Even with the stringent Bonferroni correction, the *t*-test is a parametric test that makes assumptions regarding the shape of the underlying distribution. To eliminate this effect, we applied two, nonparametric methods for gene calling. A two-class, unpaired significance analysis of microarrays (SAM) [29] identified 759 differentially-expressed genes in the parent and 478 in the transformed distribution (37.2% difference). Of 760 total genes, 477 (62.8%) were common to both lists while 283 (37.2%) were unique to only one of the two lists (Tables 4A, S4). A two-class, unpaired Kruskal-Wallis (KW) test identified 1,801 differentially-expressed genes in the parent distribution and 1800 in the transformed distribution. There was 99.9% overlap in these gene lists (Tables 4A, S5).

An alternate strategy for gene calling uses linear modeling for microarrays (LIMMA) [30] a Bayesian approach to linear modeling to calculate a moderated *t*-test. While this method assumes normality of the underlying data, it is viewed by many to be superior to standard and corrected *t*-tests and is considered robust to a variety of confounding mathematical and statistical effects [31]. LIMMA identified 2,866 differentially-expressed genes in the parent and 2,981 in the transformed distribution. Of 3,047 total genes, 2,710 (88.9%) were common to both lists while 337 (11.1%) were unique to only one of the two lists (Tables 4A, S6).

The effects of the distribution on functional annotation were studied first by using DAVID [32], [33] to annotate for gene ontology (GO) [34], [35] and Kyoto Encyclopedia of Genes and Genomes (KEGG) [36] terms in the gene lists previously generated by the SAM and KW analyses and then by performing a statistical enrichment analysis for the annotated terms. This identified 46 unique terms in the SAM lists, with 60.9% overlap between the enriched terms in the parent and transformed lists. Conversely, analysis of the lists generated by the KW analysis identified 49 enriched terms, all of which were identical in the lists from the parent and transformed datasets (100.0% overlap) (Tables 4B, S7, S8).

### Classification

**Figure 6:**
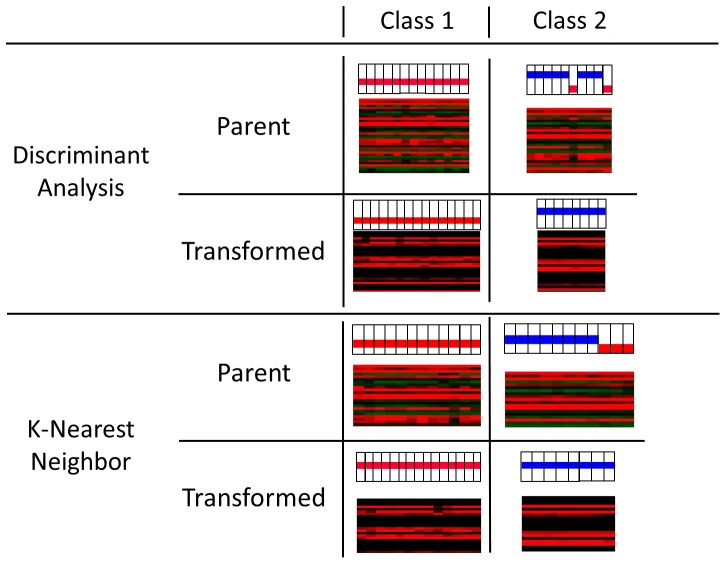
Distribution-Dependent Effects on Molecular Tumor Subclassification. Two methods of prospective molecular classification, the parametric Discriminant Analysis (DA, top) and the nonparametric K-Nearest Neighbors classifier (KNN, bottom), were used in conjunction with the parent and transformed low-grade glioma expression datasets to study distribution-dependent effects molecular tumor subclassification. Class 1 represents low-grade, 1p/19q-intact gliomas, and Class 2 represents chromosome 1p/19q codeleted, low-grade oligodendrogliomas. The topmost color bars represent the known class of each sample (black boxes; red = Class 1, blue = Class 2). The area below the color bars is a portion of the gene expression profile (red = underexpressed, green = overexpressed). DA used in conjunction with the parent (non-normal) distribution produces two misclassifications and KNN produces 3, while both methods used with the transformed dataset result in accurate molecular subclassification.

Gene expression data are frequently used as the basis for attempts at molecular-based subclassification of tumors with similar histology but different clinical phenotypes. We exploited the *a priori* knowledge [5] of two such groups within the low-grade glioma dataset (*Class 1* and *Class 2*) to simulate the classification process and to study the relationship of the results to the shape of the underlying data distribution. Discriminant analysis (DA) and k-nearest neighbors (KNN) classifiers were trained on a subset of the tumors with representatives from each class and were then used to classify ten, novel tumors into one of the two classes. Identical analyses were performed on data from the parent and transformed distributions. The results of these analyses demonstrate a 20% difference in class assignment (2/10 samples) for the DA and 30% (3/10 samples) for the KNN classifier when used with the parent data but identical classifications for both models when used with the transformed dataset (Figure 6). This effect is independent of the initial method of data reduction (SAM or *t*-test) (Figure S7).

## Discussion

### Gene Expression Data are not Normally-Distributed

The distribution of gene expression data is typically assumed to conform to a standard Gaussian (normal) distribution [11], [17]. This assumption may be attributable to a combination of three factors. First, this behavior may be (arguably) predicted by the central limit theorem [16]. Second, basic analyses of gene expression datasets, which generally include calculations of the mean and standard deviation as well as visual inspection of the data distribution, usually reveal bell-shaped curves with means (μ) centered near zero and standard deviations (σ) approximately equal to one. Third, in the early days of gene expression analysis when these assumptions were codified, datasets were small and observed differences from these theoretical values may not have achieved statistical significance.

The modern era of expression analysis, characterized by decreased cost and increased sample availability, now affords the luxury of working with datasets that include several times more samples and exponentially-more features than those of the past. These datasets, like the ones examined herein, allow more precise analysis of the distributions of expression data. In this analysis we have gone beyond calculating μ and σ (which, in fact, appear at first glance to be consistent with normality in these data) and have performed a comprehensive analysis of the higher-order central moments for these distributions. This analysis exploits the availability of nearly 10^8^ features per dataset to allow statistical significance assessments of seemingly-minor deviations from normality. In so doing, it reveals that these deviations achieve a high degree of statistical significance for all of the first four central moments. This provides convincing evidence that these cancer gene expression data do not conform to a standard Gaussian distribution (Figure 2, Table 1) and that categorical assumptions of normality for these types of datasets may be invalid.

### Gene Expression Data Exhibits Complex Distribution Characteristics

Empiric curve fitting identifies, in an unbiased fashion, distributions that most accurately model the observed distributions of the expression data. Analysis of the empirically-fit distributions provides additional information regarding data distribution and can be used to draw general conclusions regarding the types of downstream analyses that may be applicable to these datasets. This analysis demonstrates that the expression distributions are not well modeled by simplified, two- parameter distributions (such as the normal distribution) but instead require distributions with multiple (3–4) shape parameters to model the data accurately. Several derivatives of the β-prime distribution (e.g. Log-logistic, Dagum, Burr [37], [38]) were empirically identified as useful models for this data. This is logical given that the β-prime is related to the Pearson type VI distribution, which is one of a family of distributions originally used to model skewed data [38]. The hyperbolic secant distribution was also commonly identified among these empiric models. This is a more straightforward, 2-parameter distribution with an exaggerated kurtosis [39], and its identification as a useful model for these data underscores the kurtotic nature of the datasets. Finally, the 4-parameter Johnson SU [26] is a versatile distribution to model skewed and kurtotic data. Together the Johnson family of distributions covers the entire skewness-kurtosis spectrum, and the SU distribution is particularly useful with logarithmic data [38]. In aggregate, the identification of these particular families (β-prime/Pearson, hyperbolic-secant, Johnson) highlights the skewness and kurtosis of these datasets and emphasizes the inadequacy of the normal distribution to model accurately cancer gene expression data.

The goal was to use the process of distribution fitting to learn as much as possible about the underlying data structure of the cancer transcriptome, not to identify a single, “best-fit” distribution for cancer gene expression data. In fact, the KS analysis (Table 3) demonstrates that none of the 57 distributions (Table S2) against which these data were tested provided an ideal model for the underlying data. It remains unclear if a single distribution can describe the cancer transcriptome faithfully, and it is likely that no two cancer gene expression datasets will have the same, “best-fit” distribution. We hypothesize that the complex shape of the aggregate distributions may reflect their composition of various, unique distributions of the component genes. Further investigating this mixture-model hypothesis and its implications for gene calling is outside the scope of this report but merits further investigation.

Notwithstanding, identifying such a theoretical model for the aggregate distribution is not necessarily required to conduct high-quality analysis of expression data. Instead, investigators who work with gene expression data may wish to perform similar analyses to those described in order to understand the nature of the distribution of their unique datasets. This will then allow them to verify that their downstream analyses are not confounded by inaccurate assumptions regarding the shape of the data distributions.

### Non-Gaussian Distributions Affect Gene Calling and Functional Annotation

Having demonstrated that cancer gene expression data are not normally-distributed, a critical question is the degree to which these deviations from normality affect downstream, translational analyses. Considerable effort in translational oncology has been applied to identifying unique, genotypic subsets of tumors with clinically-significant phenotypic correlations, so we focused our analysis of the analytic effects of non-Gaussian distributions in this domain.

One common goal of translational investigation is to identify a set of genes with differential expression between two, known or suspected tumor subsets. We investigated this question by applying a normal-transformation to the LGG dataset, using three different algorithms to identify differentially-expressed genes between *Class1* and *Class 2* in both the parent and in the transformed data, and then performing a semi-quantitative analysis of the resulting gene lists.

The Bonferroni-corrected *t-*test identified 50 differentially-expressed genes in the parent and 55 in the transformed distribution and resulted in a distribution-dependent variability of 12.5% (see *Text S1*, for additional discussion of this calculation) (Table 4A). The extent to which this variability reflects the parametric assumptions of the classifier is difficult to determine, because the stringency of the Bonferroni correction results in a small list of differentially-expressed genes. LIMMA [30], which is considered more robust than basic and corrected *t*-tests despite its fundamental assumption of normality, was also sensitive to changes in the underlying data distribution, with an 11.1% difference in gene calling noted between the parent and transformed distributions (Table 4A, S6). Conversely, the nonparametric KW test identified 1,801 differentially-expressed genes, of which 1,800 (99.9%) were common to both lists (Table 4A, S5). Although this simulation cannot definitively demonstrate the superiority of nonparametric methods in gene calling, the result suggests that the output of nonparametric methods for identification of differential expression may be less sensitive departures from normality in the underlying distribution than their parametric counterparts. This may be logical, given the nature of the assumptions (or the lack thereof) made by parametric and nonparametric algorithms (respectively), but additional, theoretical and applied investigations will provide clarity.

Nonparametric methods may not be sufficient, however, to completely offset the effect of non-Gaussian distributions on gene calling. SAM, a nonparametric algorithm [29], identified 759 differentially-expressed genes in the parent distribution and 478 in the transformed distribution, with a distribution-dependent variability of 37.2% (Table 4A, S4). Despite SAM's classification as a nonparametric test, these data suggest that the shape of the underlying distribution has a measurable effect on the result of its analyses. This degree of variability is noteworthy, particularly from an algorithm that is considered to be among the most robust of the nonparametric gene calling tools [29], [40].

This simulation illustrates the potential effects of non-Gaussian distributions on gene calling. More importantly, it provides direct evidence against two common beliefs regarding gene expression analysis: that “small” departures from normality are insignificant to practical gene expression analyses and that “robust” gene calling algorithms can compensate completely for these effects.

### Non-Gaussian Distributions Affect Molecular Classification

An important objective of translational oncology research is to identify patterns of gene expression that distinguish phenotypically-significant subclasses of malignant disease. A common strategy is to use gene expression profiles in conjunction with *a priori* knowledge of the clinical phenotype of interest (e.g. prognosis, response to therapy) to train molecular classifiers that are subsequently capable of making phenotypic predictions for novel tumors using gene expression data. This process is mathematically-complex, and we performed a series of simulations to analyze the extent to which these training and prospective classification strategies may be affected by non-Gaussian data distributions.

We used a representative subset of 13 randomly-selected LGGs as the training set for two “robust” molecular classifiers, the parametric DA classifier and the nonparametric KNN classifier. Each classification strategy was used in conjunction with both the parent and the transformed data distributions, allowing the effects of the shape of the distribution on the classification algorithm to be examined. The results of this analysis (Figure 6) demonstrate a 20% difference in the resulting classification for DA and a 30% difference for KNN when using the parent versus the transformed data. These results suggest that the shape of the distribution has measurable effects on molecular classifiers that are considered “robust” and that both parametric and nonparametric classifiers may be sensitive to these effects.

It is important to note that the goal of this analysis was to perform a semi-quantitative analysis of the *differences* in the classifications attributable to the shape of the data distribution rather than to compare the classification *accuracy*. While both classifiers resulted in classifications that better fit our current disease models when used in conjunction with the transformed data, it is impossible to know if this truly represents an improvement in accuracy without knowing with certainty that our current disease models accurately reflect the true nature of the disease. At present the classification scheme applied in this analysis [5] remains only one potential model for subclassification of low-grade gliomas, the ultimate accuracy of which remains to be definitively determined. Notwithstanding, the primary purpose of these simulations was to investigate the possibility of distribution-depended *differences* in classification (regardless of the ultimate *accuracy*), and we have illustrated that the potential for such differences exists for both parametric and nonparametric, prospective, molecular classifiers.

### Conclusions

Cancer gene expression profiles are not normally-distributed, either on the complete-experiment or on the individual-gene level. They exhibit complex, heavy-tailed distributions characterized by statistically-significant skewness and kurtosis. The non-Gaussian distribution of these data affects identification of differentially-expressed genes, functional annotation, and prospective molecular classification. These effects are not fully corrected by multiple-testing and other “robust” modifications to *t*-test strategies, including Bonferroni corrections or linear modeling (LIMMA). The effects may be mitigated in some circumstances, although not completely eliminated, by using nonparametric analytics. This analysis therefore provides direct evidence refuting two, common beliefs in translational cancer gene expression analysis: that, “small,” departures from normality in the expression data distributions are insignificant in practice and that “robust” gene calling algorithms can fully compensate for these effects.

## Materials and Methods

### Definitions

The term *normal distribution* will refer in this manuscript to the special instance of the Gaussian distribution with mean (μ) equal to zero and standard deviation (σ) equal to unity (also known as the “standard normal” or “standard Gaussian” distribution). The term *first central moment* will be taken to mean the *first moment*, which describes the mean of a distribution. All other references to higher-order *central moments* will be used literally.

### Analytic Model

We examined the distributions of cancer gene expression data and investigated the translational implications of deviations from normality. To increase the practical applicability of these results, the experimental model has been structured to simulate the flow of expression data through a prototypic analytic pipeline, similar to those used in modern basic and translational gene expression investigations. An overview of the pipeline, the statistical methods used at each step, and the corresponding analyses of the data distributions and their implications are presented in Figure 1.

### Data Acquisition

Five publicly-available gene expression datasets derived from five unique, primary human cancer types were analyzed, including brain (glioblastoma), breast (adenocarcinoma), colon (adenocarcinoma), gastric (adenocarcinoma), and ovarian (adenocarcinoma) cancers, each representing the largest available dataset meeting inclusion criteria (Table S9). These five datasets were downloaded from the Gene Expression Omnibus [23].

To control for potential effects of cross-platform and cross-assay variability, all included expression data were assayed using the Affymetrix® Human Genome U133A-Plus 2.0 array. Because the five datasets contained a variable number of arrays, sample size-related bias was controlled by setting the experimental sample size to 180 for each group (corresponding to the smallest of the 5 datasets). A total of 180 samples for each of the five cancer types were therefore selected at random from the parent datasets. These five sets of 180 samples (a total of 900 samples) comprised the experimental datasets. A sixth, smaller dataset containing expression data for 23 low-grade gliomas was also used for analyses of gene calling, functional annotation, and tumor classification. This dataset has been previously generated by our group and comprises at least two molecular tumor subclasses, thereby facilitating this analysis [5].

Similarly, gene expression data from normal corresponding tissues, assayed with the U133A-Plus 2.0 array, were analyzed and used as the normal controls in the Log-subtraction-based analyses.

### Data Processing

To simulate typical analytic workflow of a translational molecular oncology investigation, raw expression data were normalized using the RMA method [24] with the RMA Express software package [41]. This method consists of three steps: background adjustment, quantile normalization, and summarization. To control for the possibility of bias introduced by the RMA method, the same source data was also independently normalized using the DChip algorithm [25], with default parameters, and the effects of these two normalization methods were compared (see *Results*). All normalized data were represented in Log_2_ converted form.

Remaining consistent with a prototypic translational analysis workflow, we next calculated expression ratios for each feature (relative to normal expression) using Log-subtraction. The gene expression profiles for normal tissue corresponding to each tumor type were calculated by averaging three, comparable gene expression profiles from the appropriate normal tissue, and the average value was Log_2_-converted and then subtracted from the Log_2_-converted expression of the corresponding feature in each tumor sample. This is standard practice in many semi-quantitative gene expression analyses; see the *Text S1* for further details.

### Distribution Analysis

A mathematical analysis of the first four central moments was performed for each of the sample distributions. Differences between the observed values of the central moments and those of the theoretical normal distribution were calculated. Testing for statistical significance of these differences was accomplished by comparing the values from the expression data sets to those of a simulated normal distribution of 10^7^ elements (μ = 0.000, σ = 1.001, skew = −0.025, kurtosis = 0.272, range = −9.54–5.36). Means were compared using both parametric and nonparametric tests (student's *t-test* and KW), variances were compared using the *F*-test, and skew and kurtosis were compared using standard error and Fisher's coefficients of skewness and kurtosis [42]. Additionally, both the one-sample and two-sample Kolmogorov-Smirnov (KS) test [43] (α = 0.05) were used to compare the sample distributions to the theoretical or simulated normal distribution, and the KS statistic was tested for significance using the *p*-value of the KS calculation (Figures 2, S1, S2; Tables 1, S1).

### Empiric Curve Fitting

Curve fitting was performed on all ten Log_2_-subtracted datasets (five normalized with RMA and the identical five normalized with D-Chip) by first constructing expression density histograms for each of the datasets and then by empiric fitting and parameter estimation for each of 57 potential distributions (Table S2). Optimal bin widths were calculated using the Freedman-Diaconis method [44]. Goodness-of-fit was assessed using the KS test (α = 0.05) and by examining the probability-probability (PP) plot of the empirical versus the theoretical cumulative density functions (CDF) and the quantile-quantile (QQ) plot of the observed versus expected distribution quantiles. The three best-fit distributions for each dataset were primarily determined based upon rank of the KS coefficient. When this index was not adequately representative or when approximate equivalence existed, the best-fit PP and QQ plots were used as tie-breakers (Figures 4, S3, S4, S5, S6; Table 3).

### Gene Calling and Functional Annotation Analysis

A semi-quantitative analysis of the effects of the non-Gaussian distributions of the dataset on gene calling and sub-classification within a single tumor type was performed using previously-published gene expression data from a set of low-grade gliomas [5]. This dataset comprises 23 LGGs, and previous investigations have suggested the presence of at least two distinct, molecular subgroups within this dataset, corresponding to a set of chromosome 1p/19q codeleted oligodendrogliomas and a set of 1p/19q intact low-grade gliomas [5].

A central moments analysis was conducted (as above) on this dataset, again suggesting that it was not normally-distributed (data not shown). A systematic, Box-Cox transformation [28] was then applied to the Log_2_-subtracted dataset in order to better fit the data to a normal distribution, and a central moments analysis was again performed to verify that the transform had significantly improved the normality of the distribution (Figure 5).

Next, a series of identical classification and gene calling procedures was performed on the pre- and post-transformation datasets and the results were compared to estimate, in a semi-quantitative fashion, the effects of the shape of the underlying distribution on identification of differentially-expressed genes, functional annotation, and molecular classification. We used the *a priori* definition of the two, genotypically- and phenotypically-distinct tumor subsets contained within the LGG dataset to define two classes [5], arbitrarily designated as *Class 1* for the intact gliomas and *Class 2* for the codeleted oligodendrogliomas. We applied four different statistical tests to identify genes with statistically-significant differences between the two subsets: one nonparameric method, the two-class, unpaired student's *t*-test with a standard Bonferroni correction for multiple testing (*p*<0.01) [45]; two nonparametric methods, the two-class, unpaired SAM [40] test (with false discovery rate [FDR = 0]) and the two-class KW [46] test (with a Benjamini-Hochberg correction [FDR<0.05]); and a linear modeling strategy (LIMMA) [30], α = 0.05), a Bayesian approach based on a moderated *t*-test that assumes normality but is considered robust [31]. These tests were applied to both the parent (untransformed) dataset and the (Box-Cox)-transformed dataset, and the results were compared to identify potential differences in identification of differentially-expressed genes affected solely by the shape of the distribution. A summary of the results of this analysis is presented in Table 4, and the detailed results are given in Tables S3, S4, S5, S6. Functional annotation of the gene sets derived from one parametric method (SAM) and one nonparametric method (KW) for gene calling was performed using DAVID [32], [33] to identify statistically-overrepresented (Bonferroni-corrected *p*<0.01) GO [34], [35] and KEGG [36] terms in each of these lists. The functional annotation results were compared semi-quantitatively (Tables 4B, S7, S8).

### Molecular Classification Analysis

The effect of the distribution on the accuracy of prospective, molecular classification algorithms was tested using a DA classifier and a KNN classifier. The DA [47] classifier is a multi-step algorithm that first applies an ANOVA analysis to select genes that should be near optimal for partitioning the unknown samples based upon permutations of gene expression in the training set. Next, a multivariate partial least squares method is used for gene dimensional reduction. This is followed by a polychotomous discriminant analysis. The KNN model [48] was applied using a 2-class, 4-neighbors model. Both strategies are considered robust tools for classification.

For both approaches, the Bonferroni-corrected t-test (*p*<0.01) was first used to eliminate genes whose expression did not differ significantly from control. Next, a training set consisting of 8 samples randomly selected from *Class 1* and 5 from *Class 2* (13 total) was used to train the classifier, which was then tested against a novel set known to contain 7 *Class 1* and 3 *Class 2* tumors (10 total). These classification strategies were applied to both the parent and to the transformed distributions, and the classification results were compared in a semi-quantitative fashion (Figure 5). To eliminate potential bias associated with the initial, *t-*test-based gene selection, the process was repeated for the KNN clustering with SAM replacing the *t-*test in the analytic model (Figure S7).

## Supporting Information

Figure S1 **Distribution analysis for Log_2_ (unsubtracted) data normalized using the Robust Multichip Average (RMA) method.** The source data for these graphs are the Log_2_ (unsbtracted) datasets. Bin widths have been set between 190–250 to improve visualization. Red curves represent the best-fit normal distribution. The primary image gives the histogram with the superimposed theoretical normal curve. The inset presents the quantile-quantile (QQ) plot, where deviation from the line (y = x, black) illustrates deviation of the empiric from the theoretical normal distribution. A: Brain; B: Breast; C: Colon; D: Gastric; E: Ovarian.
(TIF)

Figure S2 **Distribution analysis for Log_2_ (unsubtracted) data normalized using the DChip method.** The source data for these graphs are the Log_2_ (unsbtracted) datasets. Bin widths have been set between 190–250 to improve visualization. Red curves represent the best-fit normal distribution. The primary image gives the histogram with the superimposed theoretical normal curve. The inset presents the quantile-quantile (QQ) plot, where deviation from the line (y = x, black) illustrates deviation of the empiric from the theoretical normal distribution. A: Brain; B: Breast; C: Colon; D: Gastric; E: Ovarian.
(TIF)

Figure S3 **Distribution Fitting.** Distribution fitting for the breast cancer dataset for RMA (top) and DChip (bottom) normalized data. The three best-fit curves are superimposed on the histogram, and the normal distribution curve is included for comparison. The specific parameters for the best-fit distributions are given. The inset displays the quantile-quantile (QQ) plot for the best-fit and normal distributions. These charts demonstrate that multiparameter distributions capable of modeling skewness and kurtosis better characterize the data than the standard Gaussian (normal) distribution.
(TIF)

Figure S4 **Distribution Fitting.** Distribution fitting for the colon cancer dataset for RMA (top) and DChip (bottom) normalized data. The three best-fit curves are superimposed on the histogram, and the normal distribution curve is included for comparison. The specific parameters for the best-fit distributions are given. The inset displays the quantile-quantile (QQ) plot for the best-fit and normal distributions. These charts demonstrate that multiparameter distributions capable of modeling skewness and kurtosis better characterize the data than the standard Gaussian (normal) distribution.
(TIF)

Figure S5 **Distribution Fitting.** Distribution fitting for the gastric cancer dataset for RMA (top) and DChip (bottom) normalized data. The three best-fit curves are superimposed on the histogram, and the normal distribution curve is included for comparison. The specific parameters for the best-fit distributions are given. The inset displays the quantile-quantile (QQ) plot for the best-fit and normal distributions. These charts demonstrate that multiparameter distributions capable of modeling skewness and kurtosis better characterize the data than the standard Gaussian (normal) distribution.
(TIF)

Figure S6 **Distribution Fitting.** Distribution fitting for the ovarian cancer dataset for RMA (top) and DChip (bottom) normalized data. The three best-fit curves are superimposed on the histogram, and the normal distribution curve is included for comparison. The specific parameters for the best-fit distributions are given. The inset displays the quantile-quantile (QQ) plot for the best-fit and normal distributions. These charts demonstrate that multiparameter distributions capable of modeling skewness and kurtosis better characterize the data than the standard Gaussian (normal) distribution.
(TIF)

Figure S7 **Distribution-Dependent Effects on Molecular Tumor Subclassification Based on Two Different Methods of Gene Calling.** Two different gene calling methods, the student's *t*-test (top) and the Significance Analysis for Microarrays (SAM, bottom) were used to verify that the gene calling method used in conjunction with the K-Nearest Neighbors classifier (KNN). Both analyses use the parent (untransformed) low-grade glioma expression datasets. Class 1 represents low-grade astrocytomas, and Class 2 represents chromosome 1p/19q codeleted, low-grade oligodendrogliomas. The topmost color bars represent the known class of each sample (black boxes; red = Class 1, blue = Class 2). The area below the color bars is a portion of the gene expression profile (red = underexpressed, green = overexpressed). Both gene calling algorithms lead to identical results, indicating that this is not the primary reason for the observed differences in molecular classification (see Figure 6).
(TIF)

Table S1 **Central Moments Analysis using Log_2_ (unsubtracted) data.** Results of the central moments analysis for each of the five tumor types, using each of two normalization methods. RMA = Robust Multichip Average. * = t-test 2 class unpaired, Wilcoxon Rank-Sum vs. simulated ** = *f*-test # = Standard Error of Skewness (Fisher's Skewness Index) † = Standard Error of Kurtosis (Fisher's Kurtosis Index) ‡ = p-value [95% CI] φ = p-value.
(XLS)

Table S2 **Empiric Distributions.** Categorized list of the 57 empiric distributions tested in the curve fitting analysis.
(XLS)

Table S3 **Differentially-Expressed Genes between Low-Grade Glioma Class 1 and Class 2, as identified by the student's t-test.** Affymetrix ID is the unique probeset identifier of the gene. The second column indicates whether each gene is present in the lists from both the parent (untransformed) and transformed (Box-Cox) distributions.
(XLS)

Table S4 **Differentially-Expressed Genes between Low-Grade Glioma Class 1 and Class 2, as identified by Significance Analysis for Microarrays (SAM).** Affymetrix ID is the unique probeset identifier of the gene. The second column indicates whether each gene is present in the lists from both the parent (untransformed) and transformed (Box-Cox) distributions. FDR = False Discovery Rate.
(XLS)

Table S5 **Differentially-Expressed Genes between Low-Grade Glioma Class 1 and Class 2, as identified by the Kruskal-Wallis test.** Affymetrix ID is the unique probeset identifier of the gene. The second column indicates whether each gene is present in the lists from both the parent (untransformed) and transformed (Box-Cox) distributions. FDR = False Discovery Rate.
(XLS)

Table S6 **Differentially-Expressed Genes between Low-Grade Glioma Class 1 and Class 2, as identified by linear modeling (LIMMA).** Affymetrix ID is the unique probeset identifier of the gene. The second column indicates whether each gene is present in the lists from both the parent (untransformed) and transformed (Box-Cox) distributions.
(XLS)

Table S7 **Functional Category Enrichment Analysis (SAM).** Functional category (GO and KEGG) enrichment analysis for the list of genes differentially-expressed between low-grade glioma Class 1 and Class 2, as identified by the Significance Analysis for Microarrays (SAM) algorithm. Only categories with a Bonferroni-corrected p-value<0.01 are included. The GO or KEGG term is given in the left column, and the second column indicates whether each gene is present in the lists from both the parent (untransformed) and transformed (Box-Cox) distributions. Descriptive statistics are given at the bottom of the list.
(XLS)

Table S8 **Functional Category Enrichment Analysis (KW).** Functional category (GO and KEGG) enrichment analysis for the list of genes differentially-expressed between low-grade glioma Class 1 and Class 2, as identified by the Kruskal-Wallis test. Only categories with a Bonferroni-corrected p-value<0.01 are included. The GO or KEGG term is given in the left column, and the second column indicates whether each gene is present in the lists from both the parent (untransformed) and transformed (Box-Cox) distributions. Descriptive statistics are given at the bottom of the list.
(XLS)

Table S9 **Datasets.** These are the cancer gene expression datasets used in this analysis. GEO indicated data take from the Gene Expression Omnibus.
(XLS)

Text S1 (DOC)
