# Supplementary figures and images for: Non-Gaussian Distributions Affect Identification of Expression Patterns, Functional Annotation, and Prospective Classification in Human Cancer Genomes
Source: PLoS One. 2012 Oct 31;7(10):e46935. doi: 10.1371/journal.pone.0046935 (PMC3485292; doi:10.1371/journal.pone.0046935)

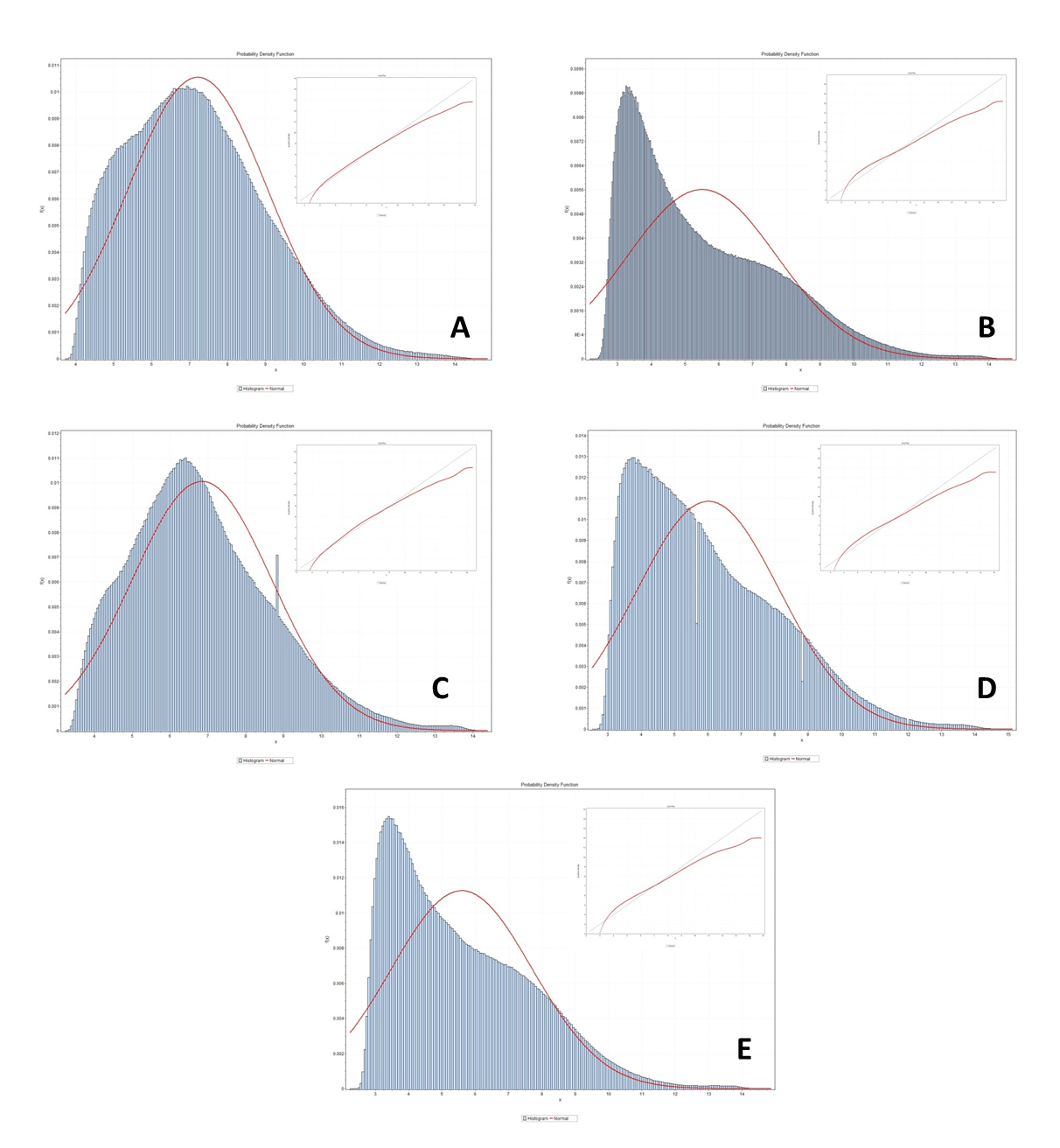

Supplement: Figure S1 — Distribution analysis for Log2 (unsubtracted) data normalized using the Robust Multichip Average (RMA) method. The source data for these graphs are the Log2 (unsbtracted) datasets. Bin widths have been set between 190–250 to improve visualization. Red curves represent the best-fit normal distribution. The primary image gives the histogram with the superimposed theoretical normal curve. The inset presents the quantile-quantile (QQ) plot, where deviation from the line (y = x, black) illustrates deviation of the empiric from the theoretical normal distribution. A: Brain; B: Breast; C: Colon; D: Gastric; E: Ovarian. (TIF) [file pone.0046935.s001.tif]

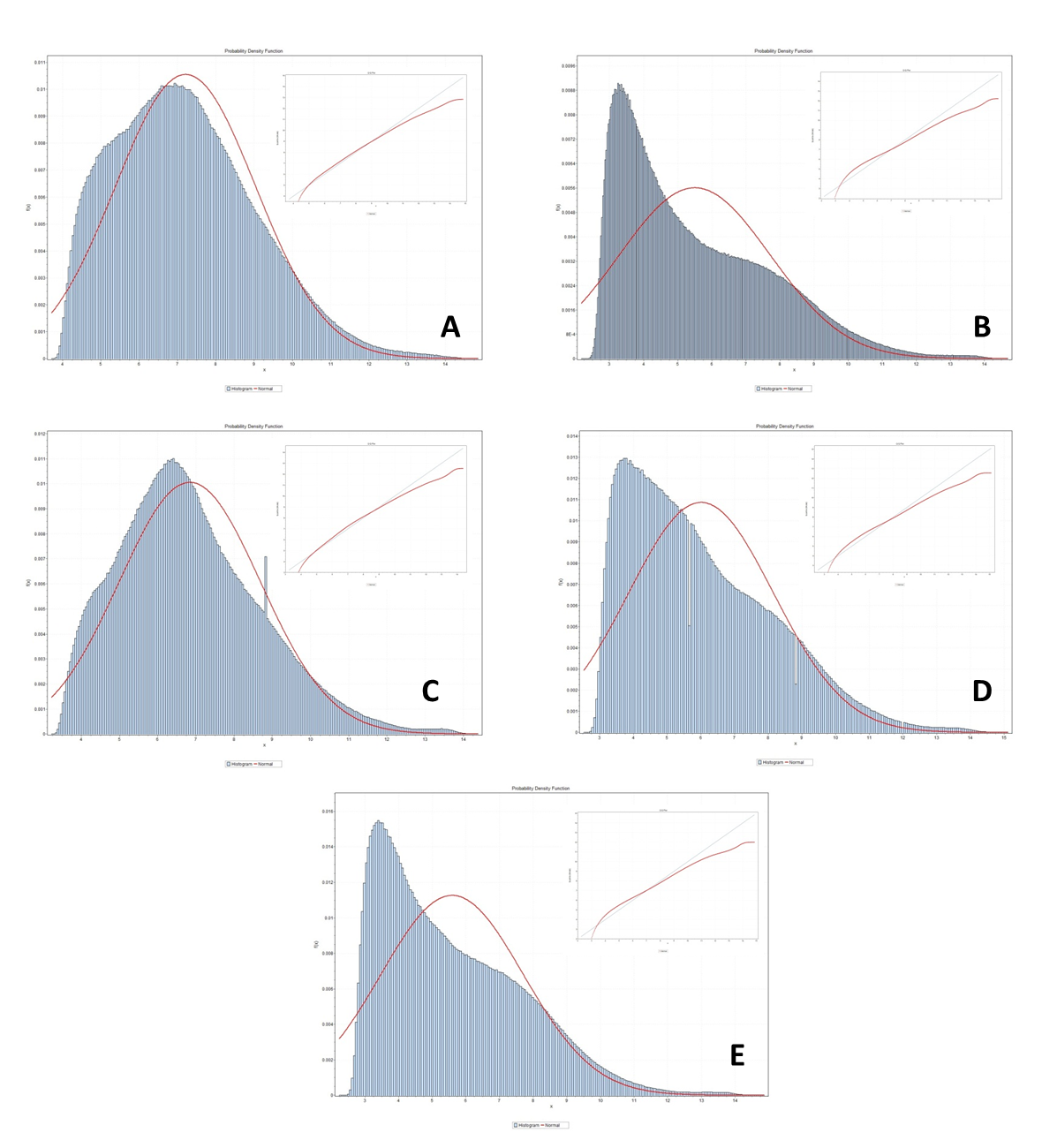

Supplement: Figure S2 — Distribution analysis for Log2 (unsubtracted) data normalized using the DChip method. The source data for these graphs are the Log2 (unsbtracted) datasets. Bin widths have been set between 190–250 to improve visualization. Red curves represent the best-fit normal distribution. The primary image gives the histogram with the superimposed theoretical normal curve. The inset presents the quantile-quantile (QQ) plot, where deviation from the line (y = x, black) illustrates deviation of the empiric from the theoretical normal distribution. A: Brain; B: Breast; C: Colon; D: Gastric; E: Ovarian. (TIF) [file pone.0046935.s002.tif]

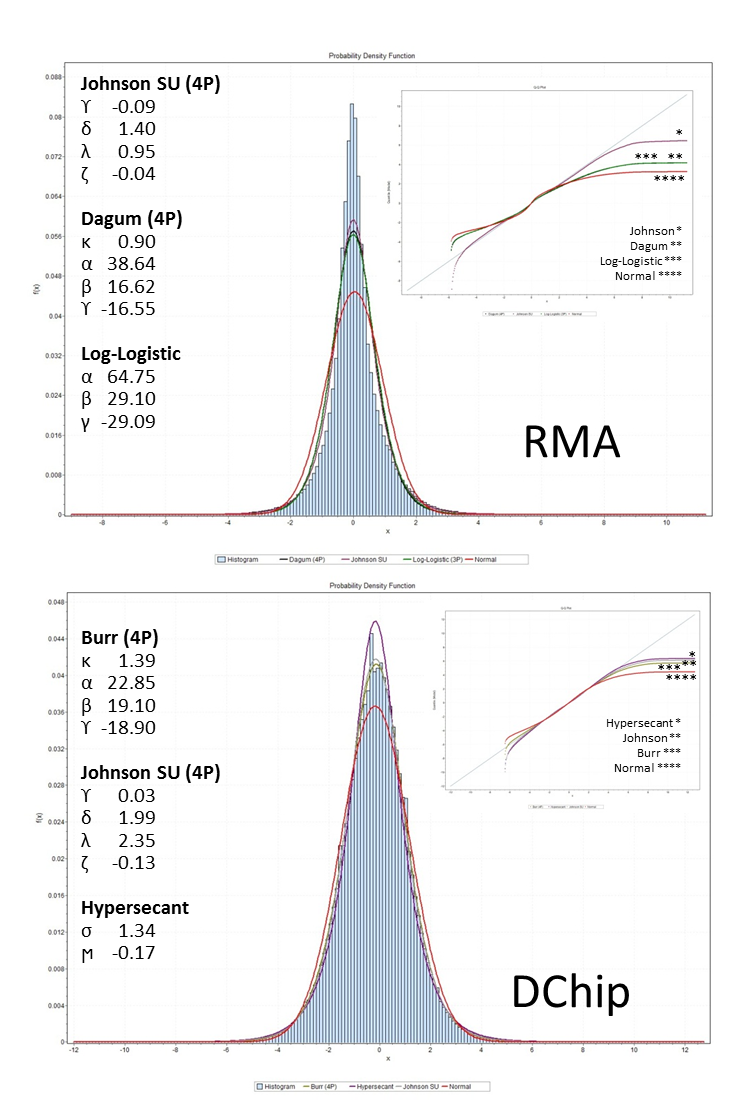

Supplement: Figure S3 — Distribution Fitting. Distribution fitting for the breast cancer dataset for RMA (top) and DChip (bottom) normalized data. The three best-fit curves are superimposed on the histogram, and the normal distribution curve is included for comparison. The specific parameters for the best-fit distributions are given. The inset displays the quantile-quantile (QQ) plot for the best-fit and normal distributions. These charts demonstrate that multiparameter distributions capable of modeling skewness and kurtosis better characterize the data than the standard Gaussian (normal) distribution. (TIF) [file pone.0046935.s003.tif]

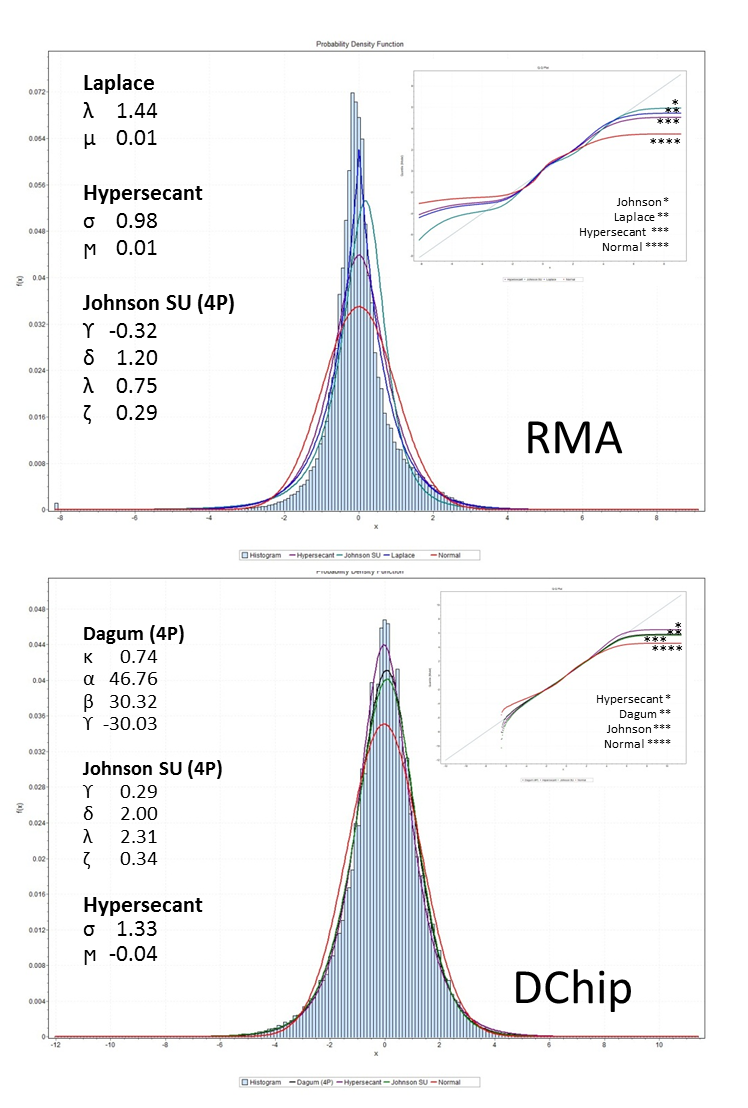

Supplement: Figure S4 — Distribution Fitting. Distribution fitting for the colon cancer dataset for RMA (top) and DChip (bottom) normalized data. The three best-fit curves are superimposed on the histogram, and the normal distribution curve is included for comparison. The specific parameters for the best-fit distributions are given. The inset displays the quantile-quantile (QQ) plot for the best-fit and normal distributions. These charts demonstrate that multiparameter distributions capable of modeling skewness and kurtosis better characterize the data than the standard Gaussian (normal) distribution. (TIF) [file pone.0046935.s004.tif]

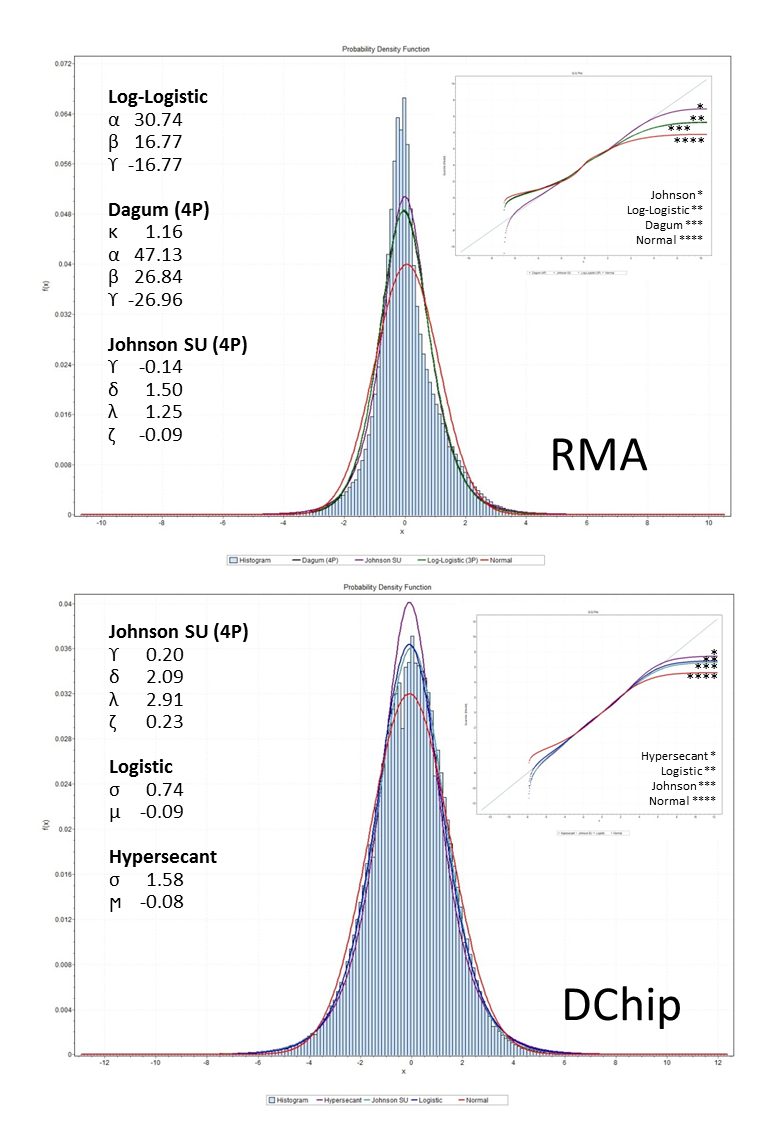

Supplement: Figure S5 — Distribution Fitting. Distribution fitting for the gastric cancer dataset for RMA (top) and DChip (bottom) normalized data. The three best-fit curves are superimposed on the histogram, and the normal distribution curve is included for comparison. The specific parameters for the best-fit distributions are given. The inset displays the quantile-quantile (QQ) plot for the best-fit and normal distributions. These charts demonstrate that multiparameter distributions capable of modeling skewness and kurtosis better characterize the data than the standard Gaussian (normal) distribution. (TIF) [file pone.0046935.s005.tif]

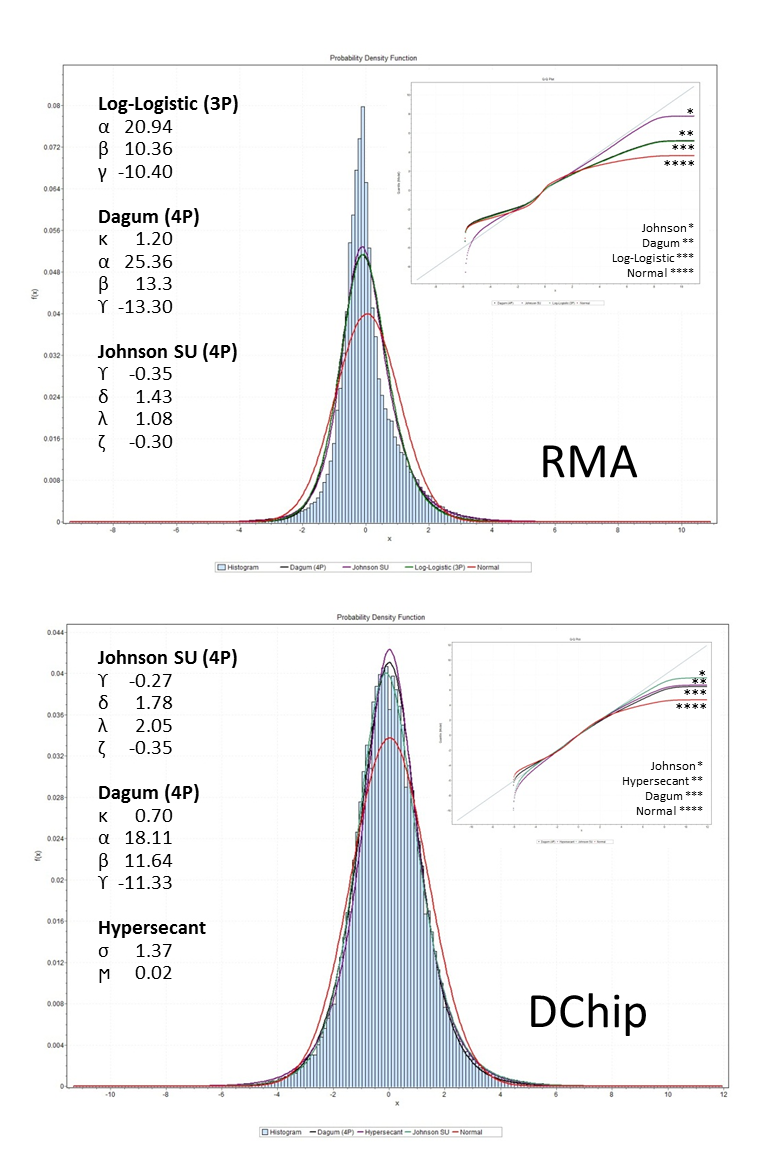

Supplement: Figure S6 — Distribution Fitting. Distribution fitting for the ovarian cancer dataset for RMA (top) and DChip (bottom) normalized data. The three best-fit curves are superimposed on the histogram, and the normal distribution curve is included for comparison. The specific parameters for the best-fit distributions are given. The inset displays the quantile-quantile (QQ) plot for the best-fit and normal distributions. These charts demonstrate that multiparameter distributions capable of modeling skewness and kurtosis better characterize the data than the standard Gaussian (normal) distribution. (TIF) [file pone.0046935.s006.tif]

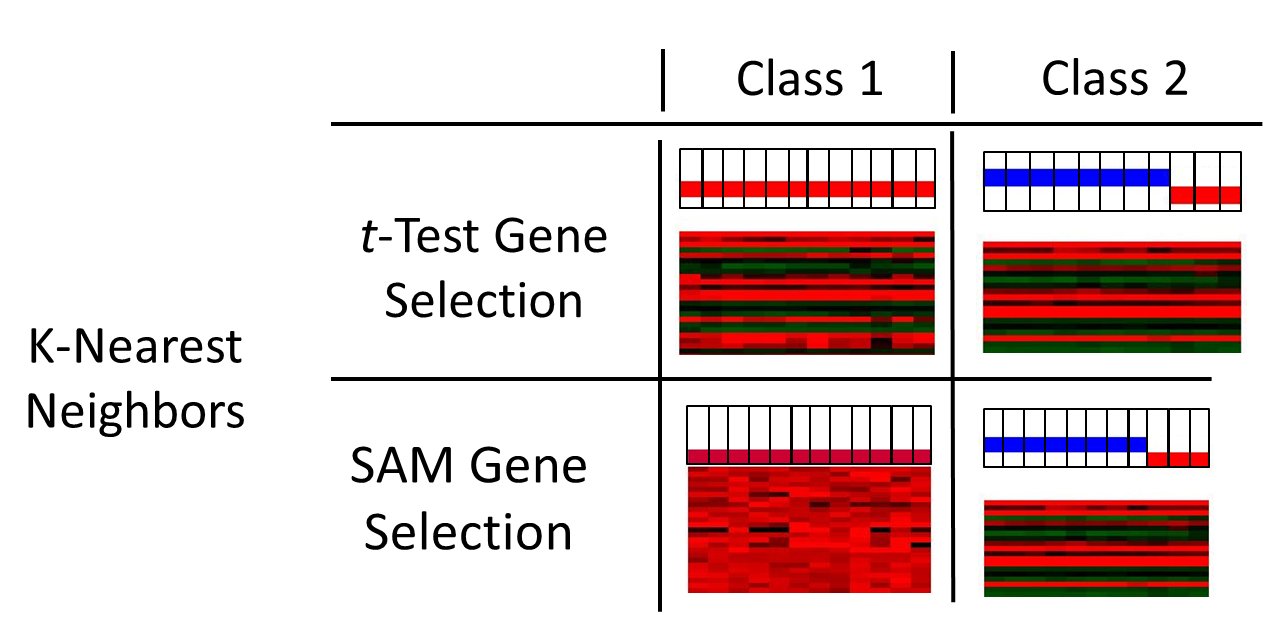

Supplement: Figure S7 — Distribution-Dependent Effects on Molecular Tumor Subclassification Based on Two Different Methods of Gene Calling. Two different gene calling methods, the student's t-test (top) and the Significance Analysis for Microarrays (SAM, bottom) were used to verify that the gene calling method used in conjunction with the K-Nearest Neighbors classifier (KNN). Both analyses use the parent (untransformed) low-grade glioma expression datasets. Class 1 represents low-grade astrocytomas, and Class 2 represents chromosome 1p/19q codeleted, low-grade oligodendrogliomas. The topmost color bars represent the known class of each sample (black boxes; red = Class 1, blue = Class 2). The area below the color bars is a portion of the gene expression profile (red = underexpressed, green = overexpressed). Both gene calling algorithms lead to identical results, indicating that this is not the primary reason for the observed differences in molecular classification (see Figure 6). (TIF) [file pone.0046935.s007.tif]
