# Supplementary material for: Non-Gaussian Distributions Affect Identification of Expression Patterns, Functional Annotation, and Prospective Classification in Human Cancer Genomes
Source: PLoS One. 2012 Oct 31;7(10):e46935. doi: 10.1371/journal.pone.0046935 (PMC3485292; doi:10.1371/journal.pone.0046935)
Supplement: Text S1 — (DOC) [file pone.0046935.s017.doc]

**Supplementary Discussion**

**Background**

*The Assumption of Normality.* The assumption that large-scale gene expression data follows a standard Gaussian (normal) distribution is supported by “visual inspection” of microarray data histograms and was strengthened by early empiric investigations. The continued belief in a standard distribution when analyzing expression data may be, in part, because it facilitates the application of parametric statistical methods to these analyses (including *t-*tests, linear models, ANOVA, and others) and because it has simplified the development of the commonly-used microarray-specific analytics. Certainly, microarray analysis is less complex when the sample is considered to conform to a normal distribution. The validity of this assumption, however, remains (like the Scottish legal verdict) ‘not proven.’

The current era of rapidly-declining costs, increasing reproducibility, and widespread application of microarray analyses now provides numerous, large-scale databases (often >10^8^ data points) against which these long-held assumptions regarding the biology of the transcriptome and the mathematics of its analysis can be tested. The first compelling evidence that the underlying distribution of gene expression data may not be Gaussian came from Hardin and Wilson. Using publicly-available data from 59 Affymetrix® arrays with a robust distribution analysis, they arrived at the opposite conclusion from their predecessors regarding this dataset and reported a non-Gaussian distribution. Additional evidence that the convenient assumption of normality may be invalid is beginning to surface among investigations of microarray noise modeling and clustering theory. Here, several have suggested that nonparametric models may outperform parametric methods, and their investigations using simulated and real datasets attribute this observation to non-Gaussian data distributions.

The implications of the potential non-Gaussian distribution of gene expression data are not, however, limited to noise modeling applications. For example, we recently investigated inconsistencies among reported, phenotypically-significant expression profiles in glioblastoma and identified non-Gaussian distributions as one potential contributor to variability in reported molecular classification schema for these tumors. These findings suggest that deviations from normality have not only theoretical and technical importance for expression analysis, but also potential implications for the accuracy of translational investigations when they abrogate the fundamental assumptions of the analytic models used to identify clinically-significant patterns of gene expression.

*Translational Significance.* In modern translational investigations, gene expression data is frequently used to screen for differentially-expressed genes that are subsequently investigated as potential drug targets or as markers for prognosis or response to therapy. Additionally, molecular markers identified through transcriptomic screening are now being used to stratify patients for clinical trials enrollment. In this context the importance of accurate identification of differentially-expressed genes and molecularly-defined subclasses is critical because the consequence of errors is both clinically and economically significant. At best these errors may result in considerable amounts of wasted time and resources invested in the study of erroneously-identified targets, while at worst they could confound the results of clinical trials or compromise the optimal management of study participants.

**Methods**

*Platform Selection.* The Affymetrix® system was selected as the primary platform for analysis because it represents a common platform used in basic and translational analyses. Additionally, this platform was selected because its automated fluidics and array processing system provides considerable uniformity in experimental conditions and because the characteristics of its signal and noise profiles are among the best characterized among microarray platforms. The Human U133A-Plus 2.0 array was selected as the primary assay because of its comprehensive coverage of the human transcriptome and because of its frequency of use in basic and translational cancer transcriptomic investigations^18^.

*Analysis of Log_2_ Transformed Data.* Log-transformation of microarray data is a common step in microarray analysis pipelines. This transformation is performed for two reasons. First, it exploits the properties of logarithms to convert division operations to subtraction, which improves computational time and efficiency and eliminates the problem of the zero denominator (Eq. S1).


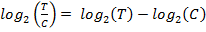
 Eq. S1

Second, this transforms the log-scale data generated in array analysis to data that can be represented with polynomial models. This is particularly helpful for the present analysis of distributions.

Notwithstanding, similar analyses can be performed using the raw (untransformed) data obtained directly from the normalization process. An example of such an analysis is given in Figures S2-3. This results in a dataset that must be compared against a logarithmic model rather than a Gaussian model. While this is mathematically possible, the results are more difficult to visualize and are inherently less meaningful to non-mathematicians. Accordingly, we have conducted the analyses in this investigation using only log-transformed data.

*Log Subtraction.* Gene expression data is semi-quantitative in nature, and the results of these analyses are typically presented in expression ratios relative to a normal control. However, because the process of log-subtraction of one entire genome from another is essentially an unbalanced transformation, there is a theoretical possibility of introducing bias during this process. To investigate this potential source of bias, we performed the distribution analyses on both Log_2_ and Log_2_-subtracted data. The results of the Log_2_-subtracted (see Eq. S1) analyses, which are the most commonly applied in the basic and translational research setting, are presented in Figure 2 and Table 3. For comparison, the results of the Log_2_ (unsubtracted) analyses are presented in Figure S2-3 and Table S1. Comparison of these analyses demonstrates that the process of log subtraction is not a major source of bias affecting the distributions of the expression data.

*Histogram Construction.* Three methods for optimal bin width selection were tested, including the “square root” method, Scott’s method, and the method of Freedman-Diaconis. While the results of empiric curve fitting did not differ appreciably between histograms constructed using each of these methods (data not shown), the method of Freedman-Diaconis produced histograms with the least noise. This, combined with its theoretical advantages, led us to select this method for bin width estimation.

*Data Transformation.* Two systematic transformation methods were tested, the Box-Cox and the square root transforms. Central moments analysis data from each of these methods demonstrated that the Box-Cox method produced a better fit to the theoretical normal distribution (data not shown), so this method was selected for use in the final analysis. More complex, distribution-based transformations (e.g. the Johnson family of transformation) could not be used because the experimental distribution did not sufficiently conform to the requisite distributions (e.g. the Johnson family of distributions).

*Variability Assessment of Gene Lists and Functional Annotation Lists.* Assessing the distribution-dependent variability of the lists of differentially-expressed genes generated in the distribution transformation simulation results in a potentially-confusing series of variability statistics. Each analysis generates three lists of genes: a list of genes present in only the parent distribution’s analysis, a list of genes present in only the transformed distribution’s analysis, and a list of genes common to both. The number of genes appearing in only one list can then be represented as a percentage of the total number of genes in that list or as a percentage of the total number of unique genes present in either list. This generates six statistics for each comparison. Additional complexity could result from cross-list comparisons.

In our analysis we have elected to use the total number of unique genes identified (from either list) as the denominator. This provides the largest possible denominator and minimizes potential overestimation of the variability rates. When the total number of genes appearing only in one list is used as the numerator, we have described this statistic as the distribution-dependent variability. We believe that maximizing the denominator reduces the risk of overstating the variability, while including all unique genes in the numerator provides a realistic estimate of the variability. Notwithstanding, we recognize that there are alternate potential strategies for calculating the percentage of variability between these lists, and we encourage readers to use whatever numerator-denominator combinations they find most intuitive.

*Analyses on Alternate Platforms.* In addition to the theoretical arguments presented in the discussion against a more comprehensive, multi-platform investigation of non-normality in cancer genomes, we believe that several practical factors would complicate such an analysis even if it were to be undertaken. First, a series of large, publicly-available datasets of identical tissue run on a number of platforms would have to be available for multiple cancer types, and we are presently unaware of any such data. Even if such a dataset was available, however, a cross-platform comparison between different vendors’ array analysis systems would be subject to various confounding effects, including but not limited to differences in experimental protocols, in the chip-assay system, and the image acquisition and processing. If the results of such a simulation identified differences in distributions then there would be evidence to support that there are some platform effects on the data distribution, but determining the comparative accuracy and precision of the measurements would necessarily be relative and therefore of limited value. Moreover, the absence of differences in the data distributions across platforms does not allow for any definitive statement to be made regarding the degree to which the observed distributions truly reflect the underlying genotype, as systematic biases related to the overall method could still be present and would not be quantifiable. Because even under the best of circumstances this type of analysis would yield results that were inconclusive and uninformative, we do not believe that the results would justify the investment of cost and efforts

An alternate and preferable strategy that may provide more definitive data regarding the etiology of the observed, non-Gaussian distribution would be to examine large datasets of whole-transcriptome sequencing data. This would control for both platform- and technique-associated biases and would therefore provide a more definitive answer to the fundamental question. We look forward to performing such an analysis when datasets of adequate quantity, quality, and scope become available.

**Limitations**

One limitation of this investigation is that it has been performed entirely on array data generated using the Affymetrix® Human Genome U133A-Plus 2.0 array. This leaves open the question of whether the observed non-Gaussian distribution of the expression data reflects a fundamental characteristic of the cancer transcriptome or a natural attribute of the Affymetrix® chip or platform. While we encourage investigators using other gene expression platforms to analyze critically the distributions of their datasets and to consider that their results may differ to some degree from ours, we also note two points that make this perceived limitation less substantial. First, the results of additional, similar testing on alternate platforms will remain semi-quantitative. Accordingly, even if alternate datasets from different platforms demonstrate differences in the shape of their distributions from those described herein, it is impossible to know which is “accurate” and which is “biased” without a known reference distribution (which currently does not exist for cancer transcriptomes). Comparative analyses of data distributions from alternate platforms are, therefore, fundamentally unable to provide an adequate answer to the question of the etiology of the observed, non-Gaussian distribution. Instead, approaches such as whole-transcriptome sequencing (RNA-seq) may be more likely to provide useful information for addressing this question and would likely be a more efficient endeavor than simply repeating the present analysis on alternate gene expression platforms. Second, from a translational perspective, a critical first step to dealing with non-Gaussian distributions is to realize *that* this effect exists, and to quantify it, before we can begin to understand fully *why* it exists. It is critical that investigators are aware of the fact that cancer gene expression datasets may violate the assumption of normality and that this may affect down-stream analyses. However, whether and to what extent this represents a platform versus a transcriptomic effect is of less significance to the applied or translational scientist, as long as the effect is appropriately measured and offset. Additional discussion relative to this subject is presented in the *Supplemental Discussion*.

Another potential limitation of this investigation is that it is unable to draw conclusions regarding the effects of the observed deviations from normality on the accuracy of downstream gene calling, functional annotation, and classification, which limits our ability to apply specific strategies to compensate unequivocally for these distribution-dependent effects. Rather than reflecting a limit in study design, however, this situation reflects the limitations in the current understanding of cancer biology. Without knowing definitively which genes are not just sufficient but necessary for the pathogenesis of different tumor types, what tumor subclasses are “real,” or what genotypic correlations are unequivocally significant clinically, it is not currently possible to conclude how the observed distribution-dependent effects affect the accuracy of gene calling, functional annotation, or molecular classification. Consequently, it is also not yet possible to test strategies to compensate for these effects, as it will remain uncertain if such strategies make the downstream results more or less accurate. The ultimate solution to this limitation is not a change in study design but rather to improve and enhance our fundamental understanding of tumor biology.

**References**

1. Giles PJ, Kipling D (2003) Normality of oligonucleotide microarray data and implications for parametric statistical analyses. Bioinformatics 19: 2254-2262.

2. Irizarry RA, Hobbs B, Collin F, Beazer-Barclay YD, Antonellis KJ, et al. (2003) Exploration, normalization, and summaries of high density oligonucleotide array probe level data. Biostatistics 4: 249-264.

3. Baldi P, Long AD (2001) A Bayesian framework for the analysis of microarray expression data: regularized t -test and statistical inferences of gene changes. Bioinformatics 17: 509-519.

4. Benjamini Y, Drai D, Elmer G, Kafkafi N, Golani I (2001) Controlling the false discovery rate in behavior genetics research. Behav Brain Res 125: 279-284.

5. Kooperberg C, Sipione S, LeBlanc M, Strand AD, Cattaneo E, et al. (2002) Evaluating test statistics to select interesting genes in microarray experiments. Hum Mol Genet 11: 2223-2232.

6. Tusher VG, Tibshirani R, Chu G (2001) Significance analysis of microarrays applied to the ionizing radiation response. Proc Natl Acad Sci U S A 98: 5116-5121.

7. Smyth G (2005) Limma: linear models for microarray data. Bioniformatics and Computational Biology Solutions using R and BioConductor. New York: Springer. pp. 397-420.

8. Kerr MK, Martin M, Churchill GA (2000) Analysis of variance for gene expression microarray data. J Comput Biol 7: 819-837.

9. Bae K, Mallick BK (2004) Gene selection using a two-level hierarchical Bayesian model. Bioinformatics 20: 3423-3430.

10. Lauretto MS, Pereira CA, Stern JM (2008) The full Bayesian significance test for mixture models: results in gene expression clustering. Genet Mol Res 7: 883-897.

11. Liang Y, Kelemen A (2008) Bayesian models and meta analysis for multiple tissue gene expression data following corticosteroid administration. BMC Bioinformatics 9: 354.

12. Ferrazzi F, Magni P, Bellazzi R (2005) Random walk models for bayesian clustering of gene expression profiles. Appl Bioinformatics 4: 263-276.

13. Hardin J, Wilson J (2009) A note on oligonucleotide expression values not being normally distributed. Biostatistics 10: 446-450.

14. Affymetrix Data (2011). Available:

http://bioconductor.org/packages/2.0/data/experiment/html/SpikeIn.html.

15. Posekany A, Felsenstein K, Sykacek P (2011) Biological assessment of robust noise models in microarray data analysis. Bioinformatics 27: 807-814.

16. Posekany A, Felenstein K, Skyacek P. Machine Learning in Systems Biology. In: Kramer S, Lawrence N, editors; 2011; Vienna. pp. 63-67.

17. Hochreiter S, Bodenhofer U, Heusel M, Mayr A, Mitterecker A, et al. (2010) FABIA: factor analysis for bicluster acquisition. Bioinformatics 26: 1520-1527.

18. Marko NF, Quackenbush J, Weil RJ (2011) Why is there a lack of consensus on molecular subgroups of glioblastoma? Understanding the nature of biological and statistical variability in glioblastoma expression data. PLoS One 6: e20826.

19. SWOG (2011) The RxPONDER Trial. Available: http://www.ecog.org/general/tailorx.html

20. ECOG (2011) The TAILORx Trial. Available: http://www.swog.org/Visitors/ViewProtocolDetails.asp?ProtocolID=2197.

**Supporting Tables**

**Table S1**

|  |  | **Log2 (Unsubtracted)** | | | |
| --- | --- | --- | --- | --- | --- |
|  |  | RMA | | DChip | |
|  |  | Statistic | Test Statistic | Statistic | Test Statistic |
| **Brain** | | | |  |  |
|  | Patients (features) | 180 (9,841,500) |  | 180 (9,841,500) |  |
| First Moment | Mean^##^ | 0.000 | 0.868, <0.0001* | 0.000 | 0.898, <0.0001* |
| Second Central Moment | Variance | 3.353 | <.0001 [3.334,3.354] ** | 5.747 | <.0001 [5.715,5.749] ** |
|  | Standard Deviation | 1.831 |  | 2.390 |  |
| Third Central Moment | Skew | 0.552 | 0.0008 (706.5)^#^ | -0.437 | 0.008 (-559.2)^#^ |
| Fourth Central Moment | Excess Kurtosis | 0.025 | 0.002 (15.7)^†^ | 0.659 | 0.002 (421.9)^†^ |
| One-Sample Kolmogorov-Smirnov | | 0.174 | <.0001 (±0.0004) ^‡^ | 0.192 | <.0001 (±0.0004) ^‡^ |
| Two-Sample Kolmogorov-Smirnov | | 0.174 | <.0001ᶲ | 0.192 | <.0001ᶲ |
|  |  |  |  |  |  |
| **Breast** |  |  |  | | |
|  | Patients (features) | 180 (9,841,500) |  | 180 (9,841,500) |  |
| First Moment | Mean^##^ | 0.000 | 0.8901, <0.0001* | 0.000 | 0.9057, <0.0001* |
| Second Central Moment | Variance | 4.915 | <.0001 [4.886,4.916] ** | 6.719 | <.0001 [6.682,6.721] ** |
|  | Standard Deviation | 2.217 |  | 2.592 |  |
| Third Central Moment | Skew | 0.884 | 0.0008 (1.1x10^3^)^#^ | 0.471 | 0.0008 (602.8)^#^ |
| Fourth Central Moment | Excess Kurtosis | 0.198 | 0.002 (126.7)^†^ | -0.281 | 0.002 (-179.9)^†^ |
| One-Sample Kolmogorov-Smirnov | | 0.280 | <.0001 (±0.0004) ^‡^ | 0.263 | <.0001 (±0.0004) ^‡^ |
| Two-Sample Kolmogorov-Smirnov | | 0.280 | <.0001ᶲ | 0.263 | <.0001ᶲ |
|  |  |  |  |  |  |
| **Colon** |  |  |  | | |
|  | Patients (features) | 180 (9,841,500) |  | 180 (9,841,500) |  |
| First Moment | Mean^##^ | 0.000 | 0.869, <0.0001* | 0.000 | 0.9089, <0.0001* |
| Second Central Moment | Variance | 3.405 | <.0001 [3.386,3.406] ** | 7.201 | <.0001 [7.161,7.203] ** |
|  | Standard Deviation | 1.845 |  | 2.683 |  |
| Third Central Moment | Skew | 0.629 | 0.0008 (805.9)^#^ | -0.294 | 0.0008 (-376.9)^#^ |
| Fourth Central Moment | Excess Kurtosis | 0.254 | 0.002 (162.7)^†^ | -0.198 | 0.002 (-126.6^3^)^†^ |
| One-Sample Kolmogorov-Smirnov | | 0.164 | <.0001 (±0.0004) ^‡^ | 0.236 | <.0001 (±0.0004) ^‡^ |
| Two-Sample Kolmogorov-Smirnov | | 0.164 | <.0001ᶲ | 0.236 | <.0001ᶲ |
|  |  |  |  |  |  |
| **Gastric** |  |  |  | | |
|  | Patients (features) | 180 (9,841,500) |  | 180 (9,841,500) |  |
| First Moment | Mean^##^ | 0.000 | 0.8868, <0.0001* | 0.000 | 0.9097, <0.0001* |
| Second Central Moment | Variance | 4.623 | <.0001 [4.597,4.624] ** | 7.335 | <.0001 [7.294,7.337] ** |
|  | Standard Deviation | 2.150 |  | 2.708 |  |
| Third Central Moment | Skew | 0.803 | 0.0008 (1.0x10^3^)^#^ | 0.339 | <0.0001 (433.7)^#^ |
| Fourth Central Moment | Excess Kurtosis | 0.158 | 0.002 (100.9)^†^ | -0.389 | 0.002 (-249.3)^†^ |
| One-Sample Kolmogorov-Smirnov | | 0.245 | <.0001 (±0.0004) ^‡^ | 0.263 | <.0001 (±0.0004) ^‡^ |
| Two-Sample Kolmogorov-Smirnov | | 0.245 | <.0001ᶲ | 0.264 | <.0001ᶲ |
|  |  |  |  |  |  |
| **Ovarian** |  |  |  | | |
|  | Patients (features) | 180 (9,841,500) |  | 180 (9,841,500) |  |
| First Moment | Mean^##^ | 0.000 | 0.8848, <0.0001* | 0.000 | 0.8952, <0.0001* |
| Second Central Moment | Variance | 4.455 | <.0001 [4.443,4.456] ** | 5.419 | <.0001 [5.389,5.420] ** |
|  | Standard Deviation | 2.110 |  | 2.328 |  |
| Third Central Moment | Skew | 0.816 | 0.0008 (1.05x10^3^)^#^ | 0.311 | 0.0008 (397.7)^#^ |
| Fourth Central Moment | Excess Kurtosis | 0.192 | 0.002 (123.0)^†^ | -0.146 | 0.002 (-93.1)† |
| One-Sample Kolmogorov-Smirnov | | 0.255 | <.0001 (±0.0004) ^‡^ | 0.218 | <.0001 (±0.0004) ^‡^ |
| Two-Sample Kolmogorov-Smirnov | | 0.256 | <.0001ᶲ | 0.218 | <.0001ᶲ |

**Table S2**

| ***Discrete Distributions*** |  |  |  |
| --- | --- | --- | --- |
|  |  |  |  |
| Bernoulli |  |  |  |
| Binomial |  |  |  |
| Discrete Uniform |  |  |  |
| Geometric |  |  |  |
| Hypergeometric |  |  |  |
| Logarithmic |  |  |  |
| Negative Binomail |  |  |  |
| Poisson |  |  |  |
|  |  |  |  |
| ***Continuous Distributions*** |  |  |  |
| **Bounded** | **Unbounded Distributions** | **Non-Negative Distributions** | **Advanced Distributions** |
|  |  |  |  |
| Beta | Cauchy | Burr | Generalized Extreme Value |
| Johnson SB | Error | Chi-Squared | Generalized Logistic |
| Kumaraswamy | Error Function | Dagum | Generalized Pareto |
| Pert | Gumbel Max | Erlang | Log-Pearson 3 |
| Power Function | Gumbel Min | Exponential | Phased Bi-Exponential |
| Reciprocal | Hyperbolic Secant | F Distribution | Phased Bi-Weibull |
| Triangular | Johnson SU | Fatigue Life (Birnbaum-Saunders) | Wakeby |
| Uniform | Laplace (Double Exponential) | Frechet |  |
|  | Logistic | Gamma |  |
|  | Normal | Generalized Gamma |  |
|  | Student's t | Inverse Gaussian |  |
|  |  | Levy |  |
|  |  | Log-Gamma |  |
|  |  | Log-Logistic |  |
|  |  | Lognormal |  |
|  |  | Nakagami |  |
|  |  | Pareto (First Kind) |  |
|  |  | Pareto (Second Kind) |  |
|  |  | Pearson Type 5 |  |
|  |  | Pearson Type 6 |  |
|  |  | Rayleigh |  |
|  |  | Rice |  |
|  |  | Weibull |  |

**Table S3**

| **Gene Calling by T-Test (Bonferroni Corrected, p=0.01)** | | | | |
| --- | --- | --- | --- | --- |
| **Parent** | |  | **Transformed** | |
| **Affymetrix ID** | **In Both?** |  | **Affymetrix ID** | **In Both?** |
| 1555575_a_at | TRUE |  | 1555575_a_at | TRUE |
| 1568619_s_at | TRUE |  | 1568619_s_at | TRUE |
| 200827_at | TRUE |  | 200827_at | TRUE |
| 201064_s_at | TRUE |  | 201064_s_at | TRUE |
| 201209_at | TRUE |  | 201209_at | TRUE |
| 201324_at | TRUE |  | 201324_at | TRUE |
| 201325_s_at | TRUE |  | 201325_s_at | TRUE |
| 202621_at | TRUE |  | 202621_at | TRUE |
| 203104_at | TRUE |  | 203104_at | TRUE |
| 203146_s_at | TRUE |  | 203146_s_at | TRUE |
| 203332_s_at | TRUE |  | 203332_s_at | TRUE |
| 204192_at | TRUE |  | 204192_at | TRUE |
| 204319_s_at | TRUE |  | 204319_s_at | TRUE |
| 205466_s_at | TRUE |  | 205466_s_at | TRUE |
| 205822_s_at | TRUE |  | 205822_s_at | TRUE |
| 206167_s_at | TRUE |  | 206167_s_at | TRUE |
| 206420_at | TRUE |  | 206420_at | TRUE |
| 207540_s_at | TRUE |  | 207540_s_at | TRUE |
| 207655_s_at | TRUE |  | 207655_s_at | TRUE |
| 209901_x_at | TRUE |  | 209901_x_at | TRUE |
| 211105_s_at | TRUE |  | 211105_s_at | TRUE |
| 211368_s_at | TRUE |  | 211368_s_at | TRUE |
| 212174_at | TRUE |  | 212174_at | TRUE |
| 213095_x_at | TRUE |  | 213095_x_at | TRUE |
| 215051_x_at | TRUE |  | 215051_x_at | TRUE |
| 215813_s_at | TRUE |  | 215813_s_at | TRUE |
| 217877_s_at | TRUE |  | 217877_s_at | TRUE |
| 219183_s_at | TRUE |  | 219183_s_at | TRUE |
| 220005_at | TRUE |  | 220005_at | TRUE |
| 220146_at | TRUE |  | 220146_at | TRUE |
| 220731_s_at | TRUE |  | 220731_s_at | TRUE |
| 221293_s_at | TRUE |  | 221293_s_at | TRUE |
| 221666_s_at | TRUE |  | 221666_s_at | TRUE |
| 221725_at | TRUE |  | 221725_at | TRUE |
| 221958_s_at | TRUE |  | 221958_s_at | TRUE |
| 222236_s_at | TRUE |  | 222236_s_at | TRUE |
| 222834_s_at | TRUE |  | 222834_s_at | TRUE |
| 225059_at | TRUE |  | 225059_at | TRUE |
| 226068_at | TRUE |  | 226068_at | TRUE |
| 226723_at | TRUE |  | 226723_at | TRUE |
| 227168_at | TRUE |  | 227168_at | TRUE |
| 227184_at | TRUE |  | 227184_at | TRUE |
| 227700_x_at | TRUE |  | 227700_x_at | TRUE |
| 227792_at | TRUE |  | 227792_at | TRUE |
| 228949_at | TRUE |  | 228949_at | TRUE |
| 230561_s_at | TRUE |  | 230561_s_at | TRUE |
| 233220_at | TRUE |  | 233220_at | TRUE |
| 236333_at | TRUE |  | 236333_at | TRUE |
| 37012_at | TRUE |  | 37012_at | TRUE |
| 206363_at | FALSE |  | 204446_s_at | FALSE |
|  |  |  | 205859_at | FALSE |
|  |  |  | 210166_at | FALSE |
|  |  |  | 212294_at | FALSE |
|  |  |  | 212587_s_at | FALSE |
|  |  |  | 218462_at | FALSE |

**Table S4**

| **Gene Calling by SAM (FDR=0.00)** | | | | |
| --- | --- | --- | --- | --- |
| **Parent** | |  | **Transformed** | |
| **Affymetrix ID** | **In Both?** |  | **Affymetrix ID** | **In Both?** |
| 1552365_at | TRUE |  | 1552365_at | TRUE |
| 1552367_a_at | TRUE |  | 1552367_a_at | TRUE |
| 1552703_s_at | TRUE |  | 1552703_s_at | TRUE |
| 1553954_at | TRUE |  | 1553954_at | TRUE |
| 1554099_a_at | TRUE |  | 1554099_a_at | TRUE |
| 1554503_a_at | TRUE |  | 1554503_a_at | TRUE |
| 1554791_a_at | TRUE |  | 1554791_a_at | TRUE |
| 1554899_s_at | TRUE |  | 1554899_s_at | TRUE |
| 1555240_s_at | TRUE |  | 1555240_s_at | TRUE |
| 1555575_a_at | TRUE |  | 1555575_a_at | TRUE |
| 1555736_a_at | TRUE |  | 1555736_a_at | TRUE |
| 1555756_a_at | TRUE |  | 1555756_a_at | TRUE |
| 1555958_at | TRUE |  | 1555958_at | TRUE |
| 1556147_at | TRUE |  | 1556147_at | TRUE |
| 1556444_a_at | TRUE |  | 1556444_a_at | TRUE |
| 1557433_at | TRUE |  | 1557433_at | TRUE |
| 1558034_s_at | TRUE |  | 1558034_s_at | TRUE |
| 1558705_at | TRUE |  | 1558705_at | TRUE |
| 1558706_a_at | TRUE |  | 1558706_a_at | TRUE |
| 1558725_at | TRUE |  | 1558725_at | TRUE |
| 1563187_at | TRUE |  | 1563187_at | TRUE |
| 1564807_at | TRUE |  | 1564807_at | TRUE |
| 1564856_s_at | TRUE |  | 1564856_s_at | TRUE |
| 1568619_s_at | TRUE |  | 1568619_s_at | TRUE |
| 1568873_at | TRUE |  | 1568873_at | TRUE |
| 200625_s_at | TRUE |  | 200625_s_at | TRUE |
| 200827_at | TRUE |  | 200827_at | TRUE |
| 200885_at | TRUE |  | 200885_at | TRUE |
| 200919_at | TRUE |  | 200919_at | TRUE |
| 201064_s_at | TRUE |  | 201064_s_at | TRUE |
| 201180_s_at | TRUE |  | 201180_s_at | TRUE |
| 201209_at | TRUE |  | 201209_at | TRUE |
| 201288_at | TRUE |  | 201288_at | TRUE |
| 201324_at | TRUE |  | 201324_at | TRUE |
| 201325_s_at | TRUE |  | 201325_s_at | TRUE |
| 201336_at | TRUE |  | 201336_at | TRUE |
| 201361_at | TRUE |  | 201361_at | TRUE |
| 201426_s_at | TRUE |  | 201426_s_at | TRUE |
| 201465_s_at | TRUE |  | 201465_s_at | TRUE |
| 201466_s_at | TRUE |  | 201466_s_at | TRUE |
| 201516_at | TRUE |  | 201516_at | TRUE |
| 201641_at | TRUE |  | 201641_at | TRUE |
| 201721_s_at | TRUE |  | 201721_s_at | TRUE |
| 201887_at | TRUE |  | 201887_at | TRUE |
| 201948_at | TRUE |  | 201948_at | TRUE |
| 201949_x_at | TRUE |  | 201949_x_at | TRUE |
| 201954_at | TRUE |  | 201954_at | TRUE |
| 202093_s_at | TRUE |  | 202093_s_at | TRUE |
| 202205_at | TRUE |  | 202205_at | TRUE |
| 202269_x_at | TRUE |  | 202269_x_at | TRUE |
| 202297_s_at | TRUE |  | 202297_s_at | TRUE |
| 202336_s_at | TRUE |  | 202336_s_at | TRUE |
| 202443_x_at | TRUE |  | 202443_x_at | TRUE |
| 202481_at | TRUE |  | 202481_at | TRUE |
| 202546_at | TRUE |  | 202546_at | TRUE |
| 202621_at | TRUE |  | 202621_at | TRUE |
| 202625_at | TRUE |  | 202625_at | TRUE |
| 202772_at | TRUE |  | 202772_at | TRUE |
| 202803_s_at | TRUE |  | 202803_s_at | TRUE |
| 202901_x_at | TRUE |  | 202901_x_at | TRUE |
| 202944_at | TRUE |  | 202944_at | TRUE |
| 202953_at | TRUE |  | 202953_at | TRUE |
| 202957_at | TRUE |  | 202957_at | TRUE |
| 203085_s_at | TRUE |  | 203085_s_at | TRUE |
| 203104_at | TRUE |  | 203104_at | TRUE |
| 203146_s_at | TRUE |  | 203146_s_at | TRUE |
| 203236_s_at | TRUE |  | 203236_s_at | TRUE |
| 203313_s_at | TRUE |  | 203313_s_at | TRUE |
| 203331_s_at | TRUE |  | 203331_s_at | TRUE |
| 203332_s_at | TRUE |  | 203332_s_at | TRUE |
| 203379_at | TRUE |  | 203379_at | TRUE |
| 203416_at | TRUE |  | 203416_at | TRUE |
| 203454_s_at | TRUE |  | 203454_s_at | TRUE |
| 203508_at | TRUE |  | 203508_at | TRUE |
| 203591_s_at | TRUE |  | 203591_s_at | TRUE |
| 203643_at | TRUE |  | 203643_at | TRUE |
| 203719_at | TRUE |  | 203719_at | TRUE |
| 203722_at | TRUE |  | 203722_at | TRUE |
| 203818_s_at | TRUE |  | 203818_s_at | TRUE |
| 203923_s_at | TRUE |  | 203923_s_at | TRUE |
| 203932_at | TRUE |  | 203932_at | TRUE |
| 204007_at | TRUE |  | 204007_at | TRUE |
| 204039_at | TRUE |  | 204039_at | TRUE |
| 204057_at | TRUE |  | 204057_at | TRUE |
| 204122_at | TRUE |  | 204122_at | TRUE |
| 204153_s_at | TRUE |  | 204153_s_at | TRUE |
| 204164_at | TRUE |  | 204164_at | TRUE |
| 204175_at | TRUE |  | 204175_at | TRUE |
| 204192_at | TRUE |  | 204192_at | TRUE |
| 204205_at | TRUE |  | 204205_at | TRUE |
| 204214_s_at | TRUE |  | 204214_s_at | TRUE |
| 204220_at | TRUE |  | 204220_at | TRUE |
| 204232_at | TRUE |  | 204232_at | TRUE |
| 204236_at | TRUE |  | 204236_at | TRUE |
| 204260_at | TRUE |  | 204260_at | TRUE |
| 204265_s_at | TRUE |  | 204265_s_at | TRUE |
| 204316_at | TRUE |  | 204316_at | TRUE |
| 204319_s_at | TRUE |  | 204319_s_at | TRUE |
| 204430_s_at | TRUE |  | 204430_s_at | TRUE |
| 204446_s_at | TRUE |  | 204446_s_at | TRUE |
| 204487_s_at | TRUE |  | 204487_s_at | TRUE |
| 204494_s_at | TRUE |  | 204494_s_at | TRUE |
| 204495_s_at | TRUE |  | 204495_s_at | TRUE |
| 204588_s_at | TRUE |  | 204588_s_at | TRUE |
| 204923_at | TRUE |  | 204923_at | TRUE |
| 204989_s_at | TRUE |  | 204989_s_at | TRUE |
| 204990_s_at | TRUE |  | 204990_s_at | TRUE |
| 205098_at | TRUE |  | 205098_at | TRUE |
| 205099_s_at | TRUE |  | 205099_s_at | TRUE |
| 205128_x_at | TRUE |  | 205128_x_at | TRUE |
| 205152_at | TRUE |  | 205152_at | TRUE |
| 205159_at | TRUE |  | 205159_at | TRUE |
| 205173_x_at | TRUE |  | 205173_x_at | TRUE |
| 205176_s_at | TRUE |  | 205176_s_at | TRUE |
| 205186_at | TRUE |  | 205186_at | TRUE |
| 205232_s_at | TRUE |  | 205232_s_at | TRUE |
| 205233_s_at | TRUE |  | 205233_s_at | TRUE |
| 205269_at | TRUE |  | 205269_at | TRUE |
| 205322_s_at | TRUE |  | 205322_s_at | TRUE |
| 205466_s_at | TRUE |  | 205466_s_at | TRUE |
| 205504_at | TRUE |  | 205504_at | TRUE |
| 205573_s_at | TRUE |  | 205573_s_at | TRUE |
| 205638_at | TRUE |  | 205638_at | TRUE |
| 205639_at | TRUE |  | 205639_at | TRUE |
| 205640_at | TRUE |  | 205640_at | TRUE |
| 205786_s_at | TRUE |  | 205786_s_at | TRUE |
| 205806_at | TRUE |  | 205806_at | TRUE |
| 205822_s_at | TRUE |  | 205822_s_at | TRUE |
| 205859_at | TRUE |  | 205859_at | TRUE |
| 205898_at | TRUE |  | 205898_at | TRUE |
| 205945_at | TRUE |  | 205945_at | TRUE |
| 205996_s_at | TRUE |  | 205996_s_at | TRUE |
| 206011_at | TRUE |  | 206011_at | TRUE |
| 206015_s_at | TRUE |  | 206015_s_at | TRUE |
| 206167_s_at | TRUE |  | 206167_s_at | TRUE |
| 206219_s_at | TRUE |  | 206219_s_at | TRUE |
| 206247_at | TRUE |  | 206247_at | TRUE |
| 206278_at | TRUE |  | 206278_at | TRUE |
| 206295_at | TRUE |  | 206295_at | TRUE |
| 206363_at | TRUE |  | 206363_at | TRUE |
| 206370_at | TRUE |  | 206370_at | TRUE |
| 206420_at | TRUE |  | 206420_at | TRUE |
| 206480_at | TRUE |  | 206480_at | TRUE |
| 206687_s_at | TRUE |  | 206687_s_at | TRUE |
| 206726_at | TRUE |  | 206726_at | TRUE |
| 206972_s_at | TRUE |  | 206972_s_at | TRUE |
| 207030_s_at | TRUE |  | 207030_s_at | TRUE |
| 207104_x_at | TRUE |  | 207104_x_at | TRUE |
| 207157_s_at | TRUE |  | 207157_s_at | TRUE |
| 207196_s_at | TRUE |  | 207196_s_at | TRUE |
| 207540_s_at | TRUE |  | 207540_s_at | TRUE |
| 207541_s_at | TRUE |  | 207541_s_at | TRUE |
| 207655_s_at | TRUE |  | 207655_s_at | TRUE |
| 207677_s_at | TRUE |  | 207677_s_at | TRUE |
| 207857_at | TRUE |  | 207857_at | TRUE |
| 207873_x_at | TRUE |  | 207873_x_at | TRUE |
| 208018_s_at | TRUE |  | 208018_s_at | TRUE |
| 208130_s_at | TRUE |  | 208130_s_at | TRUE |
| 208451_s_at | TRUE |  | 208451_s_at | TRUE |
| 208523_x_at | TRUE |  | 208523_x_at | TRUE |
| 208674_x_at | TRUE |  | 208674_x_at | TRUE |
| 208709_s_at | TRUE |  | 208709_s_at | TRUE |
| 208728_s_at | TRUE |  | 208728_s_at | TRUE |
| 208894_at | TRUE |  | 208894_at | TRUE |
| 208918_s_at | TRUE |  | 208918_s_at | TRUE |
| 208919_s_at | TRUE |  | 208919_s_at | TRUE |
| 208967_s_at | TRUE |  | 208967_s_at | TRUE |
| 208970_s_at | TRUE |  | 208970_s_at | TRUE |
| 208998_at | TRUE |  | 208998_at | TRUE |
| 209090_s_at | TRUE |  | 209090_s_at | TRUE |
| 209187_at | TRUE |  | 209187_at | TRUE |
| 209191_at | TRUE |  | 209191_at | TRUE |
| 209206_at | TRUE |  | 209206_at | TRUE |
| 209269_s_at | TRUE |  | 209269_s_at | TRUE |
| 209312_x_at | TRUE |  | 209312_x_at | TRUE |
| 209348_s_at | TRUE |  | 209348_s_at | TRUE |
| 209473_at | TRUE |  | 209473_at | TRUE |
| 209619_at | TRUE |  | 209619_at | TRUE |
| 209712_at | TRUE |  | 209712_at | TRUE |
| 209734_at | TRUE |  | 209734_at | TRUE |
| 209879_at | TRUE |  | 209879_at | TRUE |
| 209901_x_at | TRUE |  | 209901_x_at | TRUE |
| 209906_at | TRUE |  | 209906_at | TRUE |
| 209933_s_at | TRUE |  | 209933_s_at | TRUE |
| 209970_x_at | TRUE |  | 209970_x_at | TRUE |
| 210093_s_at | TRUE |  | 210093_s_at | TRUE |
| 210162_s_at | TRUE |  | 210162_s_at | TRUE |
| 210166_at | TRUE |  | 210166_at | TRUE |
| 210176_at | TRUE |  | 210176_at | TRUE |
| 210287_s_at | TRUE |  | 210287_s_at | TRUE |
| 210427_x_at | TRUE |  | 210427_x_at | TRUE |
| 210629_x_at | TRUE |  | 210629_x_at | TRUE |
| 210644_s_at | TRUE |  | 210644_s_at | TRUE |
| 210649_s_at | TRUE |  | 210649_s_at | TRUE |
| 210895_s_at | TRUE |  | 210895_s_at | TRUE |
| 211105_s_at | TRUE |  | 211105_s_at | TRUE |
| 211110_s_at | TRUE |  | 211110_s_at | TRUE |
| 211126_s_at | TRUE |  | 211126_s_at | TRUE |
| 211207_s_at | TRUE |  | 211207_s_at | TRUE |
| 211286_x_at | TRUE |  | 211286_x_at | TRUE |
| 211336_x_at | TRUE |  | 211336_x_at | TRUE |
| 211366_x_at | TRUE |  | 211366_x_at | TRUE |
| 211367_s_at | TRUE |  | 211367_s_at | TRUE |
| 211368_s_at | TRUE |  | 211368_s_at | TRUE |
| 211582_x_at | TRUE |  | 211582_x_at | TRUE |
| 211602_s_at | TRUE |  | 211602_s_at | TRUE |
| 211621_at | TRUE |  | 211621_at | TRUE |
| 211661_x_at | TRUE |  | 211661_x_at | TRUE |
| 211733_x_at | TRUE |  | 211733_x_at | TRUE |
| 211742_s_at | TRUE |  | 211742_s_at | TRUE |
| 211794_at | TRUE |  | 211794_at | TRUE |
| 211894_x_at | TRUE |  | 211894_x_at | TRUE |
| 211990_at | TRUE |  | 211990_at | TRUE |
| 212131_at | TRUE |  | 212131_at | TRUE |
| 212152_x_at | TRUE |  | 212152_x_at | TRUE |
| 212174_at | TRUE |  | 212174_at | TRUE |
| 212175_s_at | TRUE |  | 212175_s_at | TRUE |
| 212230_at | TRUE |  | 212230_at | TRUE |
| 212282_at | TRUE |  | 212282_at | TRUE |
| 212294_at | TRUE |  | 212294_at | TRUE |
| 212300_at | TRUE |  | 212300_at | TRUE |
| 212377_s_at | TRUE |  | 212377_s_at | TRUE |
| 212491_s_at | TRUE |  | 212491_s_at | TRUE |
| 212587_s_at | TRUE |  | 212587_s_at | TRUE |
| 212588_at | TRUE |  | 212588_at | TRUE |
| 212628_at | TRUE |  | 212628_at | TRUE |
| 212671_s_at | TRUE |  | 212671_s_at | TRUE |
| 212873_at | TRUE |  | 212873_at | TRUE |
| 212875_s_at | TRUE |  | 212875_s_at | TRUE |
| 212893_at | TRUE |  | 212893_at | TRUE |
| 212920_at | TRUE |  | 212920_at | TRUE |
| 212958_x_at | TRUE |  | 212958_x_at | TRUE |
| 213095_x_at | TRUE |  | 213095_x_at | TRUE |
| 213160_at | TRUE |  | 213160_at | TRUE |
| 213296_at | TRUE |  | 213296_at | TRUE |
| 213397_x_at | TRUE |  | 213397_x_at | TRUE |
| 213503_x_at | TRUE |  | 213503_x_at | TRUE |
| 213506_at | TRUE |  | 213506_at | TRUE |
| 213566_at | TRUE |  | 213566_at | TRUE |
| 213604_at | TRUE |  | 213604_at | TRUE |
| 213676_at | TRUE |  | 213676_at | TRUE |
| 213678_at | TRUE |  | 213678_at | TRUE |
| 213683_at | TRUE |  | 213683_at | TRUE |
| 213733_at | TRUE |  | 213733_at | TRUE |
| 213746_s_at | TRUE |  | 213746_s_at | TRUE |
| 213798_s_at | TRUE |  | 213798_s_at | TRUE |
| 213883_s_at | TRUE |  | 213883_s_at | TRUE |
| 213888_s_at | TRUE |  | 213888_s_at | TRUE |
| 213895_at | TRUE |  | 213895_at | TRUE |
| 214084_x_at | TRUE |  | 214084_x_at | TRUE |
| 214181_x_at | TRUE |  | 214181_x_at | TRUE |
| 214290_s_at | TRUE |  | 214290_s_at | TRUE |
| 214428_x_at | TRUE |  | 214428_x_at | TRUE |
| 214511_x_at | TRUE |  | 214511_x_at | TRUE |
| 214574_x_at | TRUE |  | 214574_x_at | TRUE |
| 214752_x_at | TRUE |  | 214752_x_at | TRUE |
| 214807_at | TRUE |  | 214807_at | TRUE |
| 215046_at | TRUE |  | 215046_at | TRUE |
| 215051_x_at | TRUE |  | 215051_x_at | TRUE |
| 215071_s_at | TRUE |  | 215071_s_at | TRUE |
| 215087_at | TRUE |  | 215087_at | TRUE |
| 215364_s_at | TRUE |  | 215364_s_at | TRUE |
| 215633_x_at | TRUE |  | 215633_x_at | TRUE |
| 215737_x_at | TRUE |  | 215737_x_at | TRUE |
| 215813_s_at | TRUE |  | 215813_s_at | TRUE |
| 216047_x_at | TRUE |  | 216047_x_at | TRUE |
| 216250_s_at | TRUE |  | 216250_s_at | TRUE |
| 216255_s_at | TRUE |  | 216255_s_at | TRUE |
| 216598_s_at | TRUE |  | 216598_s_at | TRUE |
| 217455_s_at | TRUE |  | 217455_s_at | TRUE |
| 217767_at | TRUE |  | 217767_at | TRUE |
| 217778_at | TRUE |  | 217778_at | TRUE |
| 217779_s_at | TRUE |  | 217779_s_at | TRUE |
| 217855_x_at | TRUE |  | 217855_x_at | TRUE |
| 217877_s_at | TRUE |  | 217877_s_at | TRUE |
| 217893_s_at | TRUE |  | 217893_s_at | TRUE |
| 217911_s_at | TRUE |  | 217911_s_at | TRUE |
| 218048_at | TRUE |  | 218048_at | TRUE |
| 218162_at | TRUE |  | 218162_at | TRUE |
| 218232_at | TRUE |  | 218232_at | TRUE |
| 218280_x_at | TRUE |  | 218280_x_at | TRUE |
| 218450_at | TRUE |  | 218450_at | TRUE |
| 218462_at | TRUE |  | 218462_at | TRUE |
| 218589_at | TRUE |  | 218589_at | TRUE |
| 218712_at | TRUE |  | 218712_at | TRUE |
| 218770_s_at | TRUE |  | 218770_s_at | TRUE |
| 218882_s_at | TRUE |  | 218882_s_at | TRUE |
| 218913_s_at | TRUE |  | 218913_s_at | TRUE |
| 218924_s_at | TRUE |  | 218924_s_at | TRUE |
| 218932_at | TRUE |  | 218932_at | TRUE |
| 219103_at | TRUE |  | 219103_at | TRUE |
| 219183_s_at | TRUE |  | 219183_s_at | TRUE |
| 219191_s_at | TRUE |  | 219191_s_at | TRUE |
| 219202_at | TRUE |  | 219202_at | TRUE |
| 219235_s_at | TRUE |  | 219235_s_at | TRUE |
| 219243_at | TRUE |  | 219243_at | TRUE |
| 219358_s_at | TRUE |  | 219358_s_at | TRUE |
| 219375_at | TRUE |  | 219375_at | TRUE |
| 219382_at | TRUE |  | 219382_at | TRUE |
| 219424_at | TRUE |  | 219424_at | TRUE |
| 219593_at | TRUE |  | 219593_at | TRUE |
| 219620_x_at | TRUE |  | 219620_x_at | TRUE |
| 219690_at | TRUE |  | 219690_at | TRUE |
| 219818_s_at | TRUE |  | 219818_s_at | TRUE |
| 219837_s_at | TRUE |  | 219837_s_at | TRUE |
| 219843_at | TRUE |  | 219843_at | TRUE |
| 219848_s_at | TRUE |  | 219848_s_at | TRUE |
| 219894_at | TRUE |  | 219894_at | TRUE |
| 219939_s_at | TRUE |  | 219939_s_at | TRUE |
| 220005_at | TRUE |  | 220005_at | TRUE |
| 220146_at | TRUE |  | 220146_at | TRUE |
| 220162_s_at | TRUE |  | 220162_s_at | TRUE |
| 220206_at | TRUE |  | 220206_at | TRUE |
| 220731_s_at | TRUE |  | 220731_s_at | TRUE |
| 220998_s_at | TRUE |  | 220998_s_at | TRUE |
| 221293_s_at | TRUE |  | 221293_s_at | TRUE |
| 221321_s_at | TRUE |  | 221321_s_at | TRUE |
| 221435_x_at | TRUE |  | 221435_x_at | TRUE |
| 221530_s_at | TRUE |  | 221530_s_at | TRUE |
| 221581_s_at | TRUE |  | 221581_s_at | TRUE |
| 221666_s_at | TRUE |  | 221666_s_at | TRUE |
| 221698_s_at | TRUE |  | 221698_s_at | TRUE |
| 221725_at | TRUE |  | 221725_at | TRUE |
| 221958_s_at | TRUE |  | 221958_s_at | TRUE |
| 222000_at | TRUE |  | 222000_at | TRUE |
| 222024_s_at | TRUE |  | 222024_s_at | TRUE |
| 222099_s_at | TRUE |  | 222099_s_at | TRUE |
| 222153_at | TRUE |  | 222153_at | TRUE |
| 222236_s_at | TRUE |  | 222236_s_at | TRUE |
| 222297_x_at | TRUE |  | 222297_x_at | TRUE |
| 222430_s_at | TRUE |  | 222430_s_at | TRUE |
| 222495_at | TRUE |  | 222495_at | TRUE |
| 222790_s_at | TRUE |  | 222790_s_at | TRUE |
| 222834_s_at | TRUE |  | 222834_s_at | TRUE |
| 222876_s_at | TRUE |  | 222876_s_at | TRUE |
| 222889_at | TRUE |  | 222889_at | TRUE |
| 222975_s_at | TRUE |  | 222975_s_at | TRUE |
| 223017_at | TRUE |  | 223017_at | TRUE |
| 223051_at | TRUE |  | 223051_at | TRUE |
| 223097_at | TRUE |  | 223097_at | TRUE |
| 223120_at | TRUE |  | 223120_at | TRUE |
| 223185_s_at | TRUE |  | 223185_s_at | TRUE |
| 223303_at | TRUE |  | 223303_at | TRUE |
| 223335_at | TRUE |  | 223335_at | TRUE |
| 223640_at | TRUE |  | 223640_at | TRUE |
| 223727_at | TRUE |  | 223727_at | TRUE |
| 223773_s_at | TRUE |  | 223773_s_at | TRUE |
| 223849_s_at | TRUE |  | 223849_s_at | TRUE |
| 223852_s_at | TRUE |  | 223852_s_at | TRUE |
| 224280_s_at | TRUE |  | 224280_s_at | TRUE |
| 224301_x_at | TRUE |  | 224301_x_at | TRUE |
| 224472_x_at | TRUE |  | 224472_x_at | TRUE |
| 224562_at | TRUE |  | 224562_at | TRUE |
| 224563_at | TRUE |  | 224563_at | TRUE |
| 224591_at | TRUE |  | 224591_at | TRUE |
| 224592_x_at | TRUE |  | 224592_x_at | TRUE |
| 224735_at | TRUE |  | 224735_at | TRUE |
| 224846_at | TRUE |  | 224846_at | TRUE |
| 224893_at | TRUE |  | 224893_at | TRUE |
| 224906_at | TRUE |  | 224906_at | TRUE |
| 224912_at | TRUE |  | 224912_at | TRUE |
| 224929_at | TRUE |  | 224929_at | TRUE |
| 224970_at | TRUE |  | 224970_at | TRUE |
| 224975_at | TRUE |  | 224975_at | TRUE |
| 225059_at | TRUE |  | 225059_at | TRUE |
| 225209_s_at | TRUE |  | 225209_s_at | TRUE |
| 225230_at | TRUE |  | 225230_at | TRUE |
| 225245_x_at | TRUE |  | 225245_x_at | TRUE |
| 225353_s_at | TRUE |  | 225353_s_at | TRUE |
| 225384_at | TRUE |  | 225384_at | TRUE |
| 225502_at | TRUE |  | 225502_at | TRUE |
| 225520_at | TRUE |  | 225520_at | TRUE |
| 225782_at | TRUE |  | 225782_at | TRUE |
| 225878_at | TRUE |  | 225878_at | TRUE |
| 225992_at | TRUE |  | 225992_at | TRUE |
| 226000_at | TRUE |  | 226000_at | TRUE |
| 226020_s_at | TRUE |  | 226020_s_at | TRUE |
| 226068_at | TRUE |  | 226068_at | TRUE |
| 226116_at | TRUE |  | 226116_at | TRUE |
| 226217_at | TRUE |  | 226217_at | TRUE |
| 226219_at | TRUE |  | 226219_at | TRUE |
| 226333_at | TRUE |  | 226333_at | TRUE |
| 226459_at | TRUE |  | 226459_at | TRUE |
| 226532_at | TRUE |  | 226532_at | TRUE |
| 226582_at | TRUE |  | 226582_at | TRUE |
| 226601_at | TRUE |  | 226601_at | TRUE |
| 226659_at | TRUE |  | 226659_at | TRUE |
| 226723_at | TRUE |  | 226723_at | TRUE |
| 226806_s_at | TRUE |  | 226806_s_at | TRUE |
| 226820_at | TRUE |  | 226820_at | TRUE |
| 226823_at | TRUE |  | 226823_at | TRUE |
| 226976_at | TRUE |  | 226976_at | TRUE |
| 226991_at | TRUE |  | 226991_at | TRUE |
| 227081_at | TRUE |  | 227081_at | TRUE |
| 227168_at | TRUE |  | 227168_at | TRUE |
| 227184_at | TRUE |  | 227184_at | TRUE |
| 227266_s_at | TRUE |  | 227266_s_at | TRUE |
| 227276_at | TRUE |  | 227276_at | TRUE |
| 227344_at | TRUE |  | 227344_at | TRUE |
| 227346_at | TRUE |  | 227346_at | TRUE |
| 227369_at | TRUE |  | 227369_at | TRUE |
| 227614_at | TRUE |  | 227614_at | TRUE |
| 227647_at | TRUE |  | 227647_at | TRUE |
| 227700_x_at | TRUE |  | 227700_x_at | TRUE |
| 227792_at | TRUE |  | 227792_at | TRUE |
| 227995_at | TRUE |  | 227995_at | TRUE |
| 228131_at | TRUE |  | 228131_at | TRUE |
| 228143_at | TRUE |  | 228143_at | TRUE |
| 228176_at | TRUE |  | 228176_at | TRUE |
| 228442_at | TRUE |  | 228442_at | TRUE |
| 228532_at | TRUE |  | 228532_at | TRUE |
| 228538_at | TRUE |  | 228538_at | TRUE |
| 228641_at | TRUE |  | 228641_at | TRUE |
| 228949_at | TRUE |  | 228949_at | TRUE |
| 229011_at | TRUE |  | 229011_at | TRUE |
| 229146_at | TRUE |  | 229146_at | TRUE |
| 229204_at | TRUE |  | 229204_at | TRUE |
| 229428_at | TRUE |  | 229428_at | TRUE |
| 229435_at | TRUE |  | 229435_at | TRUE |
| 229812_at | TRUE |  | 229812_at | TRUE |
| 229816_at | TRUE |  | 229816_at | TRUE |
| 229937_x_at | TRUE |  | 229937_x_at | TRUE |
| 229968_at | TRUE |  | 229968_at | TRUE |
| 230022_at | TRUE |  | 230022_at | TRUE |
| 230194_at | TRUE |  | 230194_at | TRUE |
| 230252_at | TRUE |  | 230252_at | TRUE |
| 230561_s_at | TRUE |  | 230561_s_at | TRUE |
| 230645_at | TRUE |  | 230645_at | TRUE |
| 230826_at | TRUE |  | 230826_at | TRUE |
| 230925_at | TRUE |  | 230925_at | TRUE |
| 231788_at | TRUE |  | 231788_at | TRUE |
| 231890_at | TRUE |  | 231890_at | TRUE |
| 232032_x_at | TRUE |  | 232032_x_at | TRUE |
| 232543_x_at | TRUE |  | 232543_x_at | TRUE |
| 232617_at | TRUE |  | 232617_at | TRUE |
| 232843_s_at | TRUE |  | 232843_s_at | TRUE |
| 233064_at | TRUE |  | 233064_at | TRUE |
| 233220_at | TRUE |  | 233220_at | TRUE |
| 233357_at | TRUE |  | 233357_at | TRUE |
| 234672_s_at | TRUE |  | 234672_s_at | TRUE |
| 234871_at | TRUE |  | 234871_at | TRUE |
| 235256_s_at | TRUE |  | 235256_s_at | TRUE |
| 235343_at | TRUE |  | 235343_at | TRUE |
| 235409_at | TRUE |  | 235409_at | TRUE |
| 235458_at | TRUE |  | 235458_at | TRUE |
| 235475_at | TRUE |  | 235475_at | TRUE |
| 235559_at | TRUE |  | 235559_at | TRUE |
| 235664_at | TRUE |  | 235664_at | TRUE |
| 235735_at | TRUE |  | 235735_at | TRUE |
| 235802_at | TRUE |  | 235802_at | TRUE |
| 236333_at | TRUE |  | 236333_at | TRUE |
| 237223_at | TRUE |  | 237223_at | TRUE |
| 238178_at | TRUE |  | 238178_at | TRUE |
| 238206_at | TRUE |  | 238206_at | TRUE |
| 238668_at | TRUE |  | 238668_at | TRUE |
| 239031_at | TRUE |  | 239031_at | TRUE |
| 239293_at | TRUE |  | 239293_at | TRUE |
| 239294_at | TRUE |  | 239294_at | TRUE |
| 239682_at | TRUE |  | 239682_at | TRUE |
| 241434_at | TRUE |  | 241434_at | TRUE |
| 241986_at | TRUE |  | 241986_at | TRUE |
| 242451_x_at | TRUE |  | 242451_x_at | TRUE |
| 242640_at | TRUE |  | 242640_at | TRUE |
| 242931_at | TRUE |  | 242931_at | TRUE |
| 243543_at | TRUE |  | 243543_at | TRUE |
| 243658_at | TRUE |  | 243658_at | TRUE |
| 243665_s_at | TRUE |  | 243665_s_at | TRUE |
| 243836_at | TRUE |  | 243836_at | TRUE |
| 244050_at | TRUE |  | 244050_at | TRUE |
| 244184_at | TRUE |  | 244184_at | TRUE |
| 31845_at | TRUE |  | 31845_at | TRUE |
| 33760_at | TRUE |  | 33760_at | TRUE |
| 37012_at | TRUE |  | 37012_at | TRUE |
| 37892_at | TRUE |  | 37892_at | TRUE |
| 38149_at | TRUE |  | 38149_at | TRUE |
| 38269_at | TRUE |  | 38269_at | TRUE |
| 46167_at | TRUE |  | 46167_at | TRUE |
| 1552303_a_at | FALSE |  | 215617_at | FALSE |
| 1552316_a_at | FALSE |  |  |  |
| 1553297_a_at | FALSE |  |  |  |
| 1553654_at | FALSE |  |  |  |
| 1553993_s_at | FALSE |  |  |  |
| 1554530_at | FALSE |  |  |  |
| 1554633_a_at | FALSE |  |  |  |
| 1555230_a_at | FALSE |  |  |  |
| 1555812_a_at | FALSE |  |  |  |
| 1555882_at | FALSE |  |  |  |
| 1556186_s_at | FALSE |  |  |  |
| 1556941_a_at | FALSE |  |  |  |
| 1557098_s_at | FALSE |  |  |  |
| 1557302_at | FALSE |  |  |  |
| 1557352_at | FALSE |  |  |  |
| 1557644_at | FALSE |  |  |  |
| 1559360_at | FALSE |  |  |  |
| 1560265_at | FALSE |  |  |  |
| 1563321_s_at | FALSE |  |  |  |
| 1565034_s_at | FALSE |  |  |  |
| 200070_at | FALSE |  |  |  |
| 200696_s_at | FALSE |  |  |  |
| 200859_x_at | FALSE |  |  |  |
| 200904_at | FALSE |  |  |  |
| 200905_x_at | FALSE |  |  |  |
| 201275_at | FALSE |  |  |  |
| 201309_x_at | FALSE |  |  |  |
| 201310_s_at | FALSE |  |  |  |
| 201339_s_at | FALSE |  |  |  |
| 201445_at | FALSE |  |  |  |
| 201464_x_at | FALSE |  |  |  |
| 201590_x_at | FALSE |  |  |  |
| 201627_s_at | FALSE |  |  |  |
| 201696_at | FALSE |  |  |  |
| 202108_at | FALSE |  |  |  |
| 202194_at | FALSE |  |  |  |
| 202201_at | FALSE |  |  |  |
| 202202_s_at | FALSE |  |  |  |
| 202270_at | FALSE |  |  |  |
| 202295_s_at | FALSE |  |  |  |
| 202299_s_at | FALSE |  |  |  |
| 202362_at | FALSE |  |  |  |
| 202408_s_at | FALSE |  |  |  |
| 202464_s_at | FALSE |  |  |  |
| 202502_at | FALSE |  |  |  |
| 202620_s_at | FALSE |  |  |  |
| 202626_s_at | FALSE |  |  |  |
| 202646_s_at | FALSE |  |  |  |
| 202664_at | FALSE |  |  |  |
| 202833_s_at | FALSE |  |  |  |
| 203028_s_at | FALSE |  |  |  |
| 203185_at | FALSE |  |  |  |
| 203267_s_at | FALSE |  |  |  |
| 203434_s_at | FALSE |  |  |  |
| 203470_s_at | FALSE |  |  |  |
| 203474_at | FALSE |  |  |  |
| 203476_at | FALSE |  |  |  |
| 203567_s_at | FALSE |  |  |  |
| 203623_at | FALSE |  |  |  |
| 203741_s_at | FALSE |  |  |  |
| 203922_s_at | FALSE |  |  |  |
| 204075_s_at | FALSE |  |  |  |
| 204264_at | FALSE |  |  |  |
| 204270_at | FALSE |  |  |  |
| 204320_at | FALSE |  |  |  |
| 204336_s_at | FALSE |  |  |  |
| 204463_s_at | FALSE |  |  |  |
| 204584_at | FALSE |  |  |  |
| 204613_at | FALSE |  |  |  |
| 204670_x_at | FALSE |  |  |  |
| 204689_at | FALSE |  |  |  |
| 204737_s_at | FALSE |  |  |  |
| 204773_at | FALSE |  |  |  |
| 204846_at | FALSE |  |  |  |
| 204882_at | FALSE |  |  |  |
| 204959_at | FALSE |  |  |  |
| 204961_s_at | FALSE |  |  |  |
| 205051_s_at | FALSE |  |  |  |
| 205111_s_at | FALSE |  |  |  |
| 205140_at | FALSE |  |  |  |
| 205263_at | FALSE |  |  |  |
| 205336_at | FALSE |  |  |  |
| 205349_at | FALSE |  |  |  |
| 205545_x_at | FALSE |  |  |  |
| 205685_at | FALSE |  |  |  |
| 205740_s_at | FALSE |  |  |  |
| 205952_at | FALSE |  |  |  |
| 205990_s_at | FALSE |  |  |  |
| 206013_s_at | FALSE |  |  |  |
| 206014_at | FALSE |  |  |  |
| 206101_at | FALSE |  |  |  |
| 206137_at | FALSE |  |  |  |
| 206171_at | FALSE |  |  |  |
| 206527_at | FALSE |  |  |  |
| 207233_s_at | FALSE |  |  |  |
| 207658_s_at | FALSE |  |  |  |
| 207691_x_at | FALSE |  |  |  |
| 208017_s_at | FALSE |  |  |  |
| 208064_s_at | FALSE |  |  |  |
| 208374_s_at | FALSE |  |  |  |
| 208628_s_at | FALSE |  |  |  |
| 208713_at | FALSE |  |  |  |
| 208923_at | FALSE |  |  |  |
| 209207_s_at | FALSE |  |  |  |
| 209282_at | FALSE |  |  |  |
| 209459_s_at | FALSE |  |  |  |
| 209460_at | FALSE |  |  |  |
| 209608_s_at | FALSE |  |  |  |
| 209716_at | FALSE |  |  |  |
| 210145_at | FALSE |  |  |  |
| 210246_s_at | FALSE |  |  |  |
| 210319_x_at | FALSE |  |  |  |
| 210912_x_at | FALSE |  |  |  |
| 210982_s_at | FALSE |  |  |  |
| 211795_s_at | FALSE |  |  |  |
| 211959_at | FALSE |  |  |  |
| 211962_s_at | FALSE |  |  |  |
| 212062_at | FALSE |  |  |  |
| 212119_at | FALSE |  |  |  |
| 212122_at | FALSE |  |  |  |
| 212226_s_at | FALSE |  |  |  |
| 212279_at | FALSE |  |  |  |
| 212281_s_at | FALSE |  |  |  |
| 212291_at | FALSE |  |  |  |
| 212337_at | FALSE |  |  |  |
| 212629_s_at | FALSE |  |  |  |
| 212730_at | FALSE |  |  |  |
| 212768_s_at | FALSE |  |  |  |
| 212788_x_at | FALSE |  |  |  |
| 213170_at | FALSE |  |  |  |
| 213281_at | FALSE |  |  |  |
| 213411_at | FALSE |  |  |  |
| 213425_at | FALSE |  |  |  |
| 213603_s_at | FALSE |  |  |  |
| 213607_x_at | FALSE |  |  |  |
| 213609_s_at | FALSE |  |  |  |
| 213742_at | FALSE |  |  |  |
| 214021_x_at | FALSE |  |  |  |
| 214104_at | FALSE |  |  |  |
| 214292_at | FALSE |  |  |  |
| 214495_at | FALSE |  |  |  |
| 214597_at | FALSE |  |  |  |
| 214620_x_at | FALSE |  |  |  |
| 214825_at | FALSE |  |  |  |
| 215193_x_at | FALSE |  |  |  |
| 215448_at | FALSE |  |  |  |
| 215783_s_at | FALSE |  |  |  |
| 215933_s_at | FALSE |  |  |  |
| 217478_s_at | FALSE |  |  |  |
| 217923_at | FALSE |  |  |  |
| 217950_at | FALSE |  |  |  |
| 218204_s_at | FALSE |  |  |  |
| 218341_at | FALSE |  |  |  |
| 218574_s_at | FALSE |  |  |  |
| 218684_at | FALSE |  |  |  |
| 218711_s_at | FALSE |  |  |  |
| 218720_x_at | FALSE |  |  |  |
| 218831_s_at | FALSE |  |  |  |
| 219284_at | FALSE |  |  |  |
| 219603_s_at | FALSE |  |  |  |
| 219666_at | FALSE |  |  |  |
| 219725_at | FALSE |  |  |  |
| 220088_at | FALSE |  |  |  |
| 220103_s_at | FALSE |  |  |  |
| 220585_at | FALSE |  |  |  |
| 221047_s_at | FALSE |  |  |  |
| 221269_s_at | FALSE |  |  |  |
| 221512_at | FALSE |  |  |  |
| 221727_at | FALSE |  |  |  |
| 221750_at | FALSE |  |  |  |
| 221923_s_at | FALSE |  |  |  |
| 221972_s_at | FALSE |  |  |  |
| 222154_s_at | FALSE |  |  |  |
| 222496_s_at | FALSE |  |  |  |
| 222579_at | FALSE |  |  |  |
| 222791_at | FALSE |  |  |  |
| 222866_s_at | FALSE |  |  |  |
| 223234_at | FALSE |  |  |  |
| 223452_s_at | FALSE |  |  |  |
| 223529_at | FALSE |  |  |  |
| 223553_s_at | FALSE |  |  |  |
| 223583_at | FALSE |  |  |  |
| 223692_at | FALSE |  |  |  |
| 223766_at | FALSE |  |  |  |
| 223774_at | FALSE |  |  |  |
| 223922_x_at | FALSE |  |  |  |
| 224217_s_at | FALSE |  |  |  |
| 224451_x_at | FALSE |  |  |  |
| 224708_at | FALSE |  |  |  |
| 224772_at | FALSE |  |  |  |
| 224773_at | FALSE |  |  |  |
| 224901_at | FALSE |  |  |  |
| 224916_at | FALSE |  |  |  |
| 224989_at | FALSE |  |  |  |
| 225005_at | FALSE |  |  |  |
| 225328_at | FALSE |  |  |  |
| 225356_at | FALSE |  |  |  |
| 225373_at | FALSE |  |  |  |
| 225475_at | FALSE |  |  |  |
| 225627_s_at | FALSE |  |  |  |
| 226066_at | FALSE |  |  |  |
| 226192_at | FALSE |  |  |  |
| 226197_at | FALSE |  |  |  |
| 226271_at | FALSE |  |  |  |
| 226474_at | FALSE |  |  |  |
| 226592_at | FALSE |  |  |  |
| 226865_at | FALSE |  |  |  |
| 226909_at | FALSE |  |  |  |
| 226989_at | FALSE |  |  |  |
| 227182_at | FALSE |  |  |  |
| 227253_at | FALSE |  |  |  |
| 227300_at | FALSE |  |  |  |
| 227339_at | FALSE |  |  |  |
| 227340_s_at | FALSE |  |  |  |
| 227365_at | FALSE |  |  |  |
| 227584_at | FALSE |  |  |  |
| 227983_at | FALSE |  |  |  |
| 228261_at | FALSE |  |  |  |
| 228410_at | FALSE |  |  |  |
| 228605_at | FALSE |  |  |  |
| 228658_at | FALSE |  |  |  |
| 228680_at | FALSE |  |  |  |
| 228733_at | FALSE |  |  |  |
| 228761_at | FALSE |  |  |  |
| 228950_s_at | FALSE |  |  |  |
| 229029_at | FALSE |  |  |  |
| 229134_at | FALSE |  |  |  |
| 229271_x_at | FALSE |  |  |  |
| 229498_at | FALSE |  |  |  |
| 229550_at | FALSE |  |  |  |
| 229715_at | FALSE |  |  |  |
| 229725_at | FALSE |  |  |  |
| 229818_at | FALSE |  |  |  |
| 229823_at | FALSE |  |  |  |
| 230112_at | FALSE |  |  |  |
| 230117_at | FALSE |  |  |  |
| 230258_at | FALSE |  |  |  |
| 230259_at | FALSE |  |  |  |
| 230272_at | FALSE |  |  |  |
| 230369_at | FALSE |  |  |  |
| 230391_at | FALSE |  |  |  |
| 230417_at | FALSE |  |  |  |
| 231608_at | FALSE |  |  |  |
| 231650_s_at | FALSE |  |  |  |
| 231714_s_at | FALSE |  |  |  |
| 231740_at | FALSE |  |  |  |
| 231804_at | FALSE |  |  |  |
| 231972_at | FALSE |  |  |  |
| 233310_at | FALSE |  |  |  |
| 233337_s_at | FALSE |  |  |  |
| 233406_at | FALSE |  |  |  |
| 233587_s_at | FALSE |  |  |  |
| 234710_s_at | FALSE |  |  |  |
| 235031_at | FALSE |  |  |  |
| 235384_at | FALSE |  |  |  |
| 235412_at | FALSE |  |  |  |
| 235452_at | FALSE |  |  |  |
| 235506_at | FALSE |  |  |  |
| 235742_at | FALSE |  |  |  |
| 235961_at | FALSE |  |  |  |
| 237973_at | FALSE |  |  |  |
| 238013_at | FALSE |  |  |  |
| 238194_at | FALSE |  |  |  |
| 238661_at | FALSE |  |  |  |
| 238789_at | FALSE |  |  |  |
| 238975_at | FALSE |  |  |  |
| 239250_at | FALSE |  |  |  |
| 240703_s_at | FALSE |  |  |  |
| 242091_at | FALSE |  |  |  |
| 242193_at | FALSE |  |  |  |
| 242523_at | FALSE |  |  |  |
| 242600_at | FALSE |  |  |  |
| 242618_at | FALSE |  |  |  |
| 242761_s_at | FALSE |  |  |  |
| 242870_at | FALSE |  |  |  |
| 243061_at | FALSE |  |  |  |
| 243430_at | FALSE |  |  |  |
| 243666_at | FALSE |  |  |  |
| 244071_at | FALSE |  |  |  |
| 244099_at | FALSE |  |  |  |
| 244795_at | FALSE |  |  |  |
| 37996_s_at | FALSE |  |  |  |

Table S5

| **Gene Calling by Kruskal-Wallis  (Benjamini-Hochberg corrected, FDR=0.05)** | | | | |
| --- | --- | --- | --- | --- |
| **Parent** | |  | **Transformed** | |
| **Affymetrix ID** | **In Both?** |  | **Affymetrix ID** | **In Both?** |
| 1294_at | TRUE |  | 1294_at | TRUE |
| 1552256_a_at | TRUE |  | 1552256_a_at | TRUE |
| 1552301_a_at | TRUE |  | 1552301_a_at | TRUE |
| 1552302_at | TRUE |  | 1552302_at | TRUE |
| 1552303_a_at | TRUE |  | 1552303_a_at | TRUE |
| 1552315_at | TRUE |  | 1552315_at | TRUE |
| 1552316_a_at | TRUE |  | 1552316_a_at | TRUE |
| 1552318_at | TRUE |  | 1552318_at | TRUE |
| 1552344_s_at | TRUE |  | 1552344_s_at | TRUE |
| 1552365_at | TRUE |  | 1552365_at | TRUE |
| 1552367_a_at | TRUE |  | 1552367_a_at | TRUE |
| 1552381_at | TRUE |  | 1552381_at | TRUE |
| 1552691_at | TRUE |  | 1552691_at | TRUE |
| 1552703_s_at | TRUE |  | 1552703_s_at | TRUE |
| 1552715_a_at | TRUE |  | 1552715_a_at | TRUE |
| 1552806_a_at | TRUE |  | 1552806_a_at | TRUE |
| 1553043_a_at | TRUE |  | 1553043_a_at | TRUE |
| 1553268_at | TRUE |  | 1553268_at | TRUE |
| 1553286_at | TRUE |  | 1553286_at | TRUE |
| 1553297_a_at | TRUE |  | 1553297_a_at | TRUE |
| 1553311_at | TRUE |  | 1553311_at | TRUE |
| 1553410_a_at | TRUE |  | 1553410_a_at | TRUE |
| 1553415_at | TRUE |  | 1553415_at | TRUE |
| 1553654_at | TRUE |  | 1553654_at | TRUE |
| 1553732_s_at | TRUE |  | 1553732_s_at | TRUE |
| 1553764_a_at | TRUE |  | 1553764_a_at | TRUE |
| 1553796_at | TRUE |  | 1553796_at | TRUE |
| 1553797_a_at | TRUE |  | 1553797_a_at | TRUE |
| 1553864_at | TRUE |  | 1553864_at | TRUE |
| 1553885_x_at | TRUE |  | 1553885_x_at | TRUE |
| 1553954_at | TRUE |  | 1553954_at | TRUE |
| 1553993_s_at | TRUE |  | 1553993_s_at | TRUE |
| 1554008_at | TRUE |  | 1554008_at | TRUE |
| 1554034_a_at | TRUE |  | 1554034_a_at | TRUE |
| 1554099_a_at | TRUE |  | 1554099_a_at | TRUE |
| 1554127_s_at | TRUE |  | 1554127_s_at | TRUE |
| 1554140_at | TRUE |  | 1554140_at | TRUE |
| 1554141_s_at | TRUE |  | 1554141_s_at | TRUE |
| 1554205_s_at | TRUE |  | 1554205_s_at | TRUE |
| 1554225_a_at | TRUE |  | 1554225_a_at | TRUE |
| 1554240_a_at | TRUE |  | 1554240_a_at | TRUE |
| 1554322_a_at | TRUE |  | 1554322_a_at | TRUE |
| 1554406_a_at | TRUE |  | 1554406_a_at | TRUE |
| 1554503_a_at | TRUE |  | 1554503_a_at | TRUE |
| 1554530_at | TRUE |  | 1554530_at | TRUE |
| 1554592_a_at | TRUE |  | 1554592_a_at | TRUE |
| 1554637_a_at | TRUE |  | 1554637_a_at | TRUE |
| 1554663_a_at | TRUE |  | 1554663_a_at | TRUE |
| 1554791_a_at | TRUE |  | 1554791_a_at | TRUE |
| 1554899_s_at | TRUE |  | 1554899_s_at | TRUE |
| 1555024_at | TRUE |  | 1555024_at | TRUE |
| 1555123_at | TRUE |  | 1555123_at | TRUE |
| 1555202_a_at | TRUE |  | 1555202_a_at | TRUE |
| 1555230_a_at | TRUE |  | 1555230_a_at | TRUE |
| 1555240_s_at | TRUE |  | 1555240_s_at | TRUE |
| 1555313_a_at | TRUE |  | 1555313_a_at | TRUE |
| 1555316_a_at | TRUE |  | 1555316_a_at | TRUE |
| 1555349_a_at | TRUE |  | 1555349_a_at | TRUE |
| 1555575_a_at | TRUE |  | 1555575_a_at | TRUE |
| 1555694_a_at | TRUE |  | 1555694_a_at | TRUE |
| 1555736_a_at | TRUE |  | 1555736_a_at | TRUE |
| 1555756_a_at | TRUE |  | 1555756_a_at | TRUE |
| 1555765_a_at | TRUE |  | 1555765_a_at | TRUE |
| 1555812_a_at | TRUE |  | 1555812_a_at | TRUE |
| 1555867_at | TRUE |  | 1555867_at | TRUE |
| 1555882_at | TRUE |  | 1555882_at | TRUE |
| 1555883_s_at | TRUE |  | 1555883_s_at | TRUE |
| 1555900_at | TRUE |  | 1555900_at | TRUE |
| 1555933_at | TRUE |  | 1555933_at | TRUE |
| 1555938_x_at | TRUE |  | 1555938_x_at | TRUE |
| 1555958_at | TRUE |  | 1555958_at | TRUE |
| 1556034_s_at | TRUE |  | 1556034_s_at | TRUE |
| 1556147_at | TRUE |  | 1556147_at | TRUE |
| 1556148_s_at | TRUE |  | 1556148_s_at | TRUE |
| 1556186_s_at | TRUE |  | 1556186_s_at | TRUE |
| 1556236_at | TRUE |  | 1556236_at | TRUE |
| 1556332_at | TRUE |  | 1556332_at | TRUE |
| 1556444_a_at | TRUE |  | 1556444_a_at | TRUE |
| 1556457_s_at | TRUE |  | 1556457_s_at | TRUE |
| 1556629_a_at | TRUE |  | 1556629_a_at | TRUE |
| 1556704_s_at | TRUE |  | 1556704_s_at | TRUE |
| 1556935_at | TRUE |  | 1556935_at | TRUE |
| 1556941_a_at | TRUE |  | 1556941_a_at | TRUE |
| 1557073_s_at | TRUE |  | 1557073_s_at | TRUE |
| 1557098_s_at | TRUE |  | 1557098_s_at | TRUE |
| 1557267_s_at | TRUE |  | 1557267_s_at | TRUE |
| 1557302_at | TRUE |  | 1557302_at | TRUE |
| 1557352_at | TRUE |  | 1557352_at | TRUE |
| 1557415_s_at | TRUE |  | 1557415_s_at | TRUE |
| 1557433_at | TRUE |  | 1557433_at | TRUE |
| 1557451_at | TRUE |  | 1557451_at | TRUE |
| 1557527_at | TRUE |  | 1557527_at | TRUE |
| 1557567_a_at | TRUE |  | 1557567_a_at | TRUE |
| 1557613_at | TRUE |  | 1557613_at | TRUE |
| 1557637_at | TRUE |  | 1557637_at | TRUE |
| 1557644_at | TRUE |  | 1557644_at | TRUE |
| 1557924_s_at | TRUE |  | 1557924_s_at | TRUE |
| 1558034_s_at | TRUE |  | 1558034_s_at | TRUE |
| 1558102_at | TRUE |  | 1558102_at | TRUE |
| 1558122_s_at | TRUE |  | 1558122_s_at | TRUE |
| 1558236_at | TRUE |  | 1558236_at | TRUE |
| 1558237_x_at | TRUE |  | 1558237_x_at | TRUE |
| 1558473_at | TRUE |  | 1558473_at | TRUE |
| 1558693_s_at | TRUE |  | 1558693_s_at | TRUE |
| 1558705_at | TRUE |  | 1558705_at | TRUE |
| 1558706_a_at | TRUE |  | 1558706_a_at | TRUE |
| 1558725_at | TRUE |  | 1558725_at | TRUE |
| 1559034_at | TRUE |  | 1559034_at | TRUE |
| 1559051_s_at | TRUE |  | 1559051_s_at | TRUE |
| 1559360_at | TRUE |  | 1559360_at | TRUE |
| 1559584_a_at | TRUE |  | 1559584_a_at | TRUE |
| 1559675_at | TRUE |  | 1559675_at | TRUE |
| 1559821_at | TRUE |  | 1559821_at | TRUE |
| 1559979_at | TRUE |  | 1559979_at | TRUE |
| 1560068_a_at | TRUE |  | 1560068_a_at | TRUE |
| 1560094_at | TRUE |  | 1560094_at | TRUE |
| 1560108_at | TRUE |  | 1560108_at | TRUE |
| 1560142_at | TRUE |  | 1560142_at | TRUE |
| 1560265_at | TRUE |  | 1560265_at | TRUE |
| 1560316_s_at | TRUE |  | 1560316_s_at | TRUE |
| 1560433_at | TRUE |  | 1560433_at | TRUE |
| 1560734_at | TRUE |  | 1560734_at | TRUE |
| 1561726_s_at | TRUE |  | 1561726_s_at | TRUE |
| 1561872_at | TRUE |  | 1561872_at | TRUE |
| 1562940_at | TRUE |  | 1562940_at | TRUE |
| 1562965_at | TRUE |  | 1562965_at | TRUE |
| 1562991_at | TRUE |  | 1562991_at | TRUE |
| 1563187_at | TRUE |  | 1563187_at | TRUE |
| 1563321_s_at | TRUE |  | 1563321_s_at | TRUE |
| 1564807_at | TRUE |  | 1564807_at | TRUE |
| 1564856_s_at | TRUE |  | 1564856_s_at | TRUE |
| 1565034_s_at | TRUE |  | 1565034_s_at | TRUE |
| 1565598_at | TRUE |  | 1565598_at | TRUE |
| 1565833_at | TRUE |  | 1565833_at | TRUE |
| 1566324_a_at | TRUE |  | 1566324_a_at | TRUE |
| 1566513_a_at | TRUE |  | 1566513_a_at | TRUE |
| 1566772_at | TRUE |  | 1566772_at | TRUE |
| 1566901_at | TRUE |  | 1566901_at | TRUE |
| 1566967_at | TRUE |  | 1566967_at | TRUE |
| 1568619_s_at | TRUE |  | 1568619_s_at | TRUE |
| 1568851_at | TRUE |  | 1568851_at | TRUE |
| 1568873_at | TRUE |  | 1568873_at | TRUE |
| 1568931_at | TRUE |  | 1568931_at | TRUE |
| 1568934_at | TRUE |  | 1568934_at | TRUE |
| 1569146_s_at | TRUE |  | 1569146_s_at | TRUE |
| 1569631_at | TRUE |  | 1569631_at | TRUE |
| 1569652_at | TRUE |  | 1569652_at | TRUE |
| 1569656_at | TRUE |  | 1569656_at | TRUE |
| 1569673_at | TRUE |  | 1569673_at | TRUE |
| 1569830_at | TRUE |  | 1569830_at | TRUE |
| 1569998_at | TRUE |  | 1569998_at | TRUE |
| 1570107_at | TRUE |  | 1570107_at | TRUE |
| 1570395_a_at | TRUE |  | 1570395_a_at | TRUE |
| 1570397_x_at | TRUE |  | 1570397_x_at | TRUE |
| 200070_at | TRUE |  | 200070_at | TRUE |
| 200620_at | TRUE |  | 200620_at | TRUE |
| 200625_s_at | TRUE |  | 200625_s_at | TRUE |
| 200649_at | TRUE |  | 200649_at | TRUE |
| 200660_at | TRUE |  | 200660_at | TRUE |
| 200696_s_at | TRUE |  | 200696_s_at | TRUE |
| 200758_s_at | TRUE |  | 200758_s_at | TRUE |
| 200759_x_at | TRUE |  | 200759_x_at | TRUE |
| 200791_s_at | TRUE |  | 200791_s_at | TRUE |
| 200827_at | TRUE |  | 200827_at | TRUE |
| 200859_x_at | TRUE |  | 200859_x_at | TRUE |
| 200872_at | TRUE |  | 200872_at | TRUE |
| 200878_at | TRUE |  | 200878_at | TRUE |
| 200879_s_at | TRUE |  | 200879_s_at | TRUE |
| 200885_at | TRUE |  | 200885_at | TRUE |
| 200902_at | TRUE |  | 200902_at | TRUE |
| 200904_at | TRUE |  | 200904_at | TRUE |
| 200905_x_at | TRUE |  | 200905_x_at | TRUE |
| 200919_at | TRUE |  | 200919_at | TRUE |
| 200931_s_at | TRUE |  | 200931_s_at | TRUE |
| 200975_at | TRUE |  | 200975_at | TRUE |
| 201012_at | TRUE |  | 201012_at | TRUE |
| 201064_s_at | TRUE |  | 201064_s_at | TRUE |
| 201125_s_at | TRUE |  | 201125_s_at | TRUE |
| 201137_s_at | TRUE |  | 201137_s_at | TRUE |
| 201160_s_at | TRUE |  | 201160_s_at | TRUE |
| 201161_s_at | TRUE |  | 201161_s_at | TRUE |
| 201180_s_at | TRUE |  | 201180_s_at | TRUE |
| 201209_at | TRUE |  | 201209_at | TRUE |
| 201215_at | TRUE |  | 201215_at | TRUE |
| 201225_s_at | TRUE |  | 201225_s_at | TRUE |
| 201247_at | TRUE |  | 201247_at | TRUE |
| 201275_at | TRUE |  | 201275_at | TRUE |
| 201278_at | TRUE |  | 201278_at | TRUE |
| 201288_at | TRUE |  | 201288_at | TRUE |
| 201309_x_at | TRUE |  | 201309_x_at | TRUE |
| 201310_s_at | TRUE |  | 201310_s_at | TRUE |
| 201319_at | TRUE |  | 201319_at | TRUE |
| 201324_at | TRUE |  | 201324_at | TRUE |
| 201325_s_at | TRUE |  | 201325_s_at | TRUE |
| 201336_at | TRUE |  | 201336_at | TRUE |
| 201339_s_at | TRUE |  | 201339_s_at | TRUE |
| 201356_at | TRUE |  | 201356_at | TRUE |
| 201361_at | TRUE |  | 201361_at | TRUE |
| 201369_s_at | TRUE |  | 201369_s_at | TRUE |
| 201419_at | TRUE |  | 201419_at | TRUE |
| 201422_at | TRUE |  | 201422_at | TRUE |
| 201426_s_at | TRUE |  | 201426_s_at | TRUE |
| 201445_at | TRUE |  | 201445_at | TRUE |
| 201464_x_at | TRUE |  | 201464_x_at | TRUE |
| 201465_s_at | TRUE |  | 201465_s_at | TRUE |
| 201466_s_at | TRUE |  | 201466_s_at | TRUE |
| 201487_at | TRUE |  | 201487_at | TRUE |
| 201493_s_at | TRUE |  | 201493_s_at | TRUE |
| 201516_at | TRUE |  | 201516_at | TRUE |
| 201590_x_at | TRUE |  | 201590_x_at | TRUE |
| 201625_s_at | TRUE |  | 201625_s_at | TRUE |
| 201627_s_at | TRUE |  | 201627_s_at | TRUE |
| 201641_at | TRUE |  | 201641_at | TRUE |
| 201696_at | TRUE |  | 201696_at | TRUE |
| 201721_s_at | TRUE |  | 201721_s_at | TRUE |
| 201778_s_at | TRUE |  | 201778_s_at | TRUE |
| 201819_at | TRUE |  | 201819_at | TRUE |
| 201838_s_at | TRUE |  | 201838_s_at | TRUE |
| 201850_at | TRUE |  | 201850_at | TRUE |
| 201883_s_at | TRUE |  | 201883_s_at | TRUE |
| 201887_at | TRUE |  | 201887_at | TRUE |
| 201888_s_at | TRUE |  | 201888_s_at | TRUE |
| 201935_s_at | TRUE |  | 201935_s_at | TRUE |
| 201948_at | TRUE |  | 201948_at | TRUE |
| 201949_x_at | TRUE |  | 201949_x_at | TRUE |
| 201950_x_at | TRUE |  | 201950_x_at | TRUE |
| 201954_at | TRUE |  | 201954_at | TRUE |
| 201997_s_at | TRUE |  | 201997_s_at | TRUE |
| 201998_at | TRUE |  | 201998_at | TRUE |
| 202022_at | TRUE |  | 202022_at | TRUE |
| 202033_s_at | TRUE |  | 202033_s_at | TRUE |
| 202067_s_at | TRUE |  | 202067_s_at | TRUE |
| 202068_s_at | TRUE |  | 202068_s_at | TRUE |
| 202093_s_at | TRUE |  | 202093_s_at | TRUE |
| 202108_at | TRUE |  | 202108_at | TRUE |
| 202111_at | TRUE |  | 202111_at | TRUE |
| 202115_s_at | TRUE |  | 202115_s_at | TRUE |
| 202153_s_at | TRUE |  | 202153_s_at | TRUE |
| 202193_at | TRUE |  | 202193_at | TRUE |
| 202194_at | TRUE |  | 202194_at | TRUE |
| 202201_at | TRUE |  | 202201_at | TRUE |
| 202202_s_at | TRUE |  | 202202_s_at | TRUE |
| 202205_at | TRUE |  | 202205_at | TRUE |
| 202264_s_at | TRUE |  | 202264_s_at | TRUE |
| 202265_at | TRUE |  | 202265_at | TRUE |
| 202269_x_at | TRUE |  | 202269_x_at | TRUE |
| 202270_at | TRUE |  | 202270_at | TRUE |
| 202292_x_at | TRUE |  | 202292_x_at | TRUE |
| 202295_s_at | TRUE |  | 202295_s_at | TRUE |
| 202297_s_at | TRUE |  | 202297_s_at | TRUE |
| 202299_s_at | TRUE |  | 202299_s_at | TRUE |
| 202336_s_at | TRUE |  | 202336_s_at | TRUE |
| 202362_at | TRUE |  | 202362_at | TRUE |
| 202368_s_at | TRUE |  | 202368_s_at | TRUE |
| 202369_s_at | TRUE |  | 202369_s_at | TRUE |
| 202376_at | TRUE |  | 202376_at | TRUE |
| 202377_at | TRUE |  | 202377_at | TRUE |
| 202408_s_at | TRUE |  | 202408_s_at | TRUE |
| 202412_s_at | TRUE |  | 202412_s_at | TRUE |
| 202443_x_at | TRUE |  | 202443_x_at | TRUE |
| 202445_s_at | TRUE |  | 202445_s_at | TRUE |
| 202464_s_at | TRUE |  | 202464_s_at | TRUE |
| 202470_s_at | TRUE |  | 202470_s_at | TRUE |
| 202475_at | TRUE |  | 202475_at | TRUE |
| 202481_at | TRUE |  | 202481_at | TRUE |
| 202502_at | TRUE |  | 202502_at | TRUE |
| 202522_at | TRUE |  | 202522_at | TRUE |
| 202528_at | TRUE |  | 202528_at | TRUE |
| 202539_s_at | TRUE |  | 202539_s_at | TRUE |
| 202540_s_at | TRUE |  | 202540_s_at | TRUE |
| 202546_at | TRUE |  | 202546_at | TRUE |
| 202620_s_at | TRUE |  | 202620_s_at | TRUE |
| 202621_at | TRUE |  | 202621_at | TRUE |
| 202625_at | TRUE |  | 202625_at | TRUE |
| 202626_s_at | TRUE |  | 202626_s_at | TRUE |
| 202636_at | TRUE |  | 202636_at | TRUE |
| 202638_s_at | TRUE |  | 202638_s_at | TRUE |
| 202646_s_at | TRUE |  | 202646_s_at | TRUE |
| 202664_at | TRUE |  | 202664_at | TRUE |
| 202687_s_at | TRUE |  | 202687_s_at | TRUE |
| 202748_at | TRUE |  | 202748_at | TRUE |
| 202772_at | TRUE |  | 202772_at | TRUE |
| 202803_s_at | TRUE |  | 202803_s_at | TRUE |
| 202819_s_at | TRUE |  | 202819_s_at | TRUE |
| 202833_s_at | TRUE |  | 202833_s_at | TRUE |
| 202838_at | TRUE |  | 202838_at | TRUE |
| 202863_at | TRUE |  | 202863_at | TRUE |
| 202864_s_at | TRUE |  | 202864_s_at | TRUE |
| 202901_x_at | TRUE |  | 202901_x_at | TRUE |
| 202902_s_at | TRUE |  | 202902_s_at | TRUE |
| 202939_at | TRUE |  | 202939_at | TRUE |
| 202943_s_at | TRUE |  | 202943_s_at | TRUE |
| 202944_at | TRUE |  | 202944_at | TRUE |
| 202947_s_at | TRUE |  | 202947_s_at | TRUE |
| 202950_at | TRUE |  | 202950_at | TRUE |
| 202953_at | TRUE |  | 202953_at | TRUE |
| 202957_at | TRUE |  | 202957_at | TRUE |
| 202990_at | TRUE |  | 202990_at | TRUE |
| 202997_s_at | TRUE |  | 202997_s_at | TRUE |
| 202998_s_at | TRUE |  | 202998_s_at | TRUE |
| 203020_at | TRUE |  | 203020_at | TRUE |
| 203027_s_at | TRUE |  | 203027_s_at | TRUE |
| 203028_s_at | TRUE |  | 203028_s_at | TRUE |
| 203052_at | TRUE |  | 203052_at | TRUE |
| 203055_s_at | TRUE |  | 203055_s_at | TRUE |
| 203068_at | TRUE |  | 203068_at | TRUE |
| 203085_s_at | TRUE |  | 203085_s_at | TRUE |
| 203104_at | TRUE |  | 203104_at | TRUE |
| 203145_at | TRUE |  | 203145_at | TRUE |
| 203146_s_at | TRUE |  | 203146_s_at | TRUE |
| 203175_at | TRUE |  | 203175_at | TRUE |
| 203182_s_at | TRUE |  | 203182_s_at | TRUE |
| 203185_at | TRUE |  | 203185_at | TRUE |
| 203197_s_at | TRUE |  | 203197_s_at | TRUE |
| 203205_at | TRUE |  | 203205_at | TRUE |
| 203236_s_at | TRUE |  | 203236_s_at | TRUE |
| 203240_at | TRUE |  | 203240_at | TRUE |
| 203267_s_at | TRUE |  | 203267_s_at | TRUE |
| 203268_s_at | TRUE |  | 203268_s_at | TRUE |
| 203271_s_at | TRUE |  | 203271_s_at | TRUE |
| 203277_at | TRUE |  | 203277_at | TRUE |
| 203282_at | TRUE |  | 203282_at | TRUE |
| 203283_s_at | TRUE |  | 203283_s_at | TRUE |
| 203313_s_at | TRUE |  | 203313_s_at | TRUE |
| 203317_at | TRUE |  | 203317_at | TRUE |
| 203331_s_at | TRUE |  | 203331_s_at | TRUE |
| 203332_s_at | TRUE |  | 203332_s_at | TRUE |
| 203340_s_at | TRUE |  | 203340_s_at | TRUE |
| 203345_s_at | TRUE |  | 203345_s_at | TRUE |
| 203346_s_at | TRUE |  | 203346_s_at | TRUE |
| 203359_s_at | TRUE |  | 203359_s_at | TRUE |
| 203360_s_at | TRUE |  | 203360_s_at | TRUE |
| 203379_at | TRUE |  | 203379_at | TRUE |
| 203416_at | TRUE |  | 203416_at | TRUE |
| 203442_x_at | TRUE |  | 203442_x_at | TRUE |
| 203445_s_at | TRUE |  | 203445_s_at | TRUE |
| 203454_s_at | TRUE |  | 203454_s_at | TRUE |
| 203470_s_at | TRUE |  | 203470_s_at | TRUE |
| 203471_s_at | TRUE |  | 203471_s_at | TRUE |
| 203473_at | TRUE |  | 203473_at | TRUE |
| 203474_at | TRUE |  | 203474_at | TRUE |
| 203476_at | TRUE |  | 203476_at | TRUE |
| 203507_at | TRUE |  | 203507_at | TRUE |
| 203508_at | TRUE |  | 203508_at | TRUE |
| 203527_s_at | TRUE |  | 203527_s_at | TRUE |
| 203561_at | TRUE |  | 203561_at | TRUE |
| 203567_s_at | TRUE |  | 203567_s_at | TRUE |
| 203568_s_at | TRUE |  | 203568_s_at | TRUE |
| 203591_s_at | TRUE |  | 203591_s_at | TRUE |
| 203611_at | TRUE |  | 203611_at | TRUE |
| 203623_at | TRUE |  | 203623_at | TRUE |
| 203643_at | TRUE |  | 203643_at | TRUE |
| 203646_at | TRUE |  | 203646_at | TRUE |
| 203665_at | TRUE |  | 203665_at | TRUE |
| 203719_at | TRUE |  | 203719_at | TRUE |
| 203720_s_at | TRUE |  | 203720_s_at | TRUE |
| 203722_at | TRUE |  | 203722_at | TRUE |
| 203741_s_at | TRUE |  | 203741_s_at | TRUE |
| 203748_x_at | TRUE |  | 203748_x_at | TRUE |
| 203810_at | TRUE |  | 203810_at | TRUE |
| 203818_s_at | TRUE |  | 203818_s_at | TRUE |
| 203857_s_at | TRUE |  | 203857_s_at | TRUE |
| 203922_s_at | TRUE |  | 203922_s_at | TRUE |
| 203923_s_at | TRUE |  | 203923_s_at | TRUE |
| 203925_at | TRUE |  | 203925_at | TRUE |
| 203932_at | TRUE |  | 203932_at | TRUE |
| 203959_s_at | TRUE |  | 203959_s_at | TRUE |
| 204007_at | TRUE |  | 204007_at | TRUE |
| 204039_at | TRUE |  | 204039_at | TRUE |
| 204046_at | TRUE |  | 204046_at | TRUE |
| 204051_s_at | TRUE |  | 204051_s_at | TRUE |
| 204057_at | TRUE |  | 204057_at | TRUE |
| 204070_at | TRUE |  | 204070_at | TRUE |
| 204075_s_at | TRUE |  | 204075_s_at | TRUE |
| 204120_s_at | TRUE |  | 204120_s_at | TRUE |
| 204122_at | TRUE |  | 204122_at | TRUE |
| 204153_s_at | TRUE |  | 204153_s_at | TRUE |
| 204158_s_at | TRUE |  | 204158_s_at | TRUE |
| 204164_at | TRUE |  | 204164_at | TRUE |
| 204175_at | TRUE |  | 204175_at | TRUE |
| 204190_at | TRUE |  | 204190_at | TRUE |
| 204192_at | TRUE |  | 204192_at | TRUE |
| 204197_s_at | TRUE |  | 204197_s_at | TRUE |
| 204205_at | TRUE |  | 204205_at | TRUE |
| 204214_s_at | TRUE |  | 204214_s_at | TRUE |
| 204215_at | TRUE |  | 204215_at | TRUE |
| 204220_at | TRUE |  | 204220_at | TRUE |
| 204225_at | TRUE |  | 204225_at | TRUE |
| 204226_at | TRUE |  | 204226_at | TRUE |
| 204228_at | TRUE |  | 204228_at | TRUE |
| 204232_at | TRUE |  | 204232_at | TRUE |
| 204236_at | TRUE |  | 204236_at | TRUE |
| 204260_at | TRUE |  | 204260_at | TRUE |
| 204264_at | TRUE |  | 204264_at | TRUE |
| 204265_s_at | TRUE |  | 204265_s_at | TRUE |
| 204270_at | TRUE |  | 204270_at | TRUE |
| 204316_at | TRUE |  | 204316_at | TRUE |
| 204319_s_at | TRUE |  | 204319_s_at | TRUE |
| 204320_at | TRUE |  | 204320_at | TRUE |
| 204336_s_at | TRUE |  | 204336_s_at | TRUE |
| 204411_at | TRUE |  | 204411_at | TRUE |
| 204425_at | TRUE |  | 204425_at | TRUE |
| 204430_s_at | TRUE |  | 204430_s_at | TRUE |
| 204446_s_at | TRUE |  | 204446_s_at | TRUE |
| 204463_s_at | TRUE |  | 204463_s_at | TRUE |
| 204464_s_at | TRUE |  | 204464_s_at | TRUE |
| 204465_s_at | TRUE |  | 204465_s_at | TRUE |
| 204472_at | TRUE |  | 204472_at | TRUE |
| 204487_s_at | TRUE |  | 204487_s_at | TRUE |
| 204493_at | TRUE |  | 204493_at | TRUE |
| 204494_s_at | TRUE |  | 204494_s_at | TRUE |
| 204495_s_at | TRUE |  | 204495_s_at | TRUE |
| 204573_at | TRUE |  | 204573_at | TRUE |
| 204584_at | TRUE |  | 204584_at | TRUE |
| 204585_s_at | TRUE |  | 204585_s_at | TRUE |
| 204588_s_at | TRUE |  | 204588_s_at | TRUE |
| 204612_at | TRUE |  | 204612_at | TRUE |
| 204613_at | TRUE |  | 204613_at | TRUE |
| 204639_at | TRUE |  | 204639_at | TRUE |
| 204670_x_at | TRUE |  | 204670_x_at | TRUE |
| 204685_s_at | TRUE |  | 204685_s_at | TRUE |
| 204689_at | TRUE |  | 204689_at | TRUE |
| 204697_s_at | TRUE |  | 204697_s_at | TRUE |
| 204723_at | TRUE |  | 204723_at | TRUE |
| 204737_s_at | TRUE |  | 204737_s_at | TRUE |
| 204749_at | TRUE |  | 204749_at | TRUE |
| 204773_at | TRUE |  | 204773_at | TRUE |
| 204786_s_at | TRUE |  | 204786_s_at | TRUE |
| 204806_x_at | TRUE |  | 204806_x_at | TRUE |
| 204811_s_at | TRUE |  | 204811_s_at | TRUE |
| 204813_at | TRUE |  | 204813_at | TRUE |
| 204834_at | TRUE |  | 204834_at | TRUE |
| 204846_at | TRUE |  | 204846_at | TRUE |
| 204850_s_at | TRUE |  | 204850_s_at | TRUE |
| 204882_at | TRUE |  | 204882_at | TRUE |
| 204908_s_at | TRUE |  | 204908_s_at | TRUE |
| 204912_at | TRUE |  | 204912_at | TRUE |
| 204923_at | TRUE |  | 204923_at | TRUE |
| 204929_s_at | TRUE |  | 204929_s_at | TRUE |
| 204932_at | TRUE |  | 204932_at | TRUE |
| 204953_at | TRUE |  | 204953_at | TRUE |
| 204959_at | TRUE |  | 204959_at | TRUE |
| 204961_s_at | TRUE |  | 204961_s_at | TRUE |
| 204989_s_at | TRUE |  | 204989_s_at | TRUE |
| 204990_s_at | TRUE |  | 204990_s_at | TRUE |
| 205002_at | TRUE |  | 205002_at | TRUE |
| 205039_s_at | TRUE |  | 205039_s_at | TRUE |
| 205051_s_at | TRUE |  | 205051_s_at | TRUE |
| 205087_at | TRUE |  | 205087_at | TRUE |
| 205098_at | TRUE |  | 205098_at | TRUE |
| 205099_s_at | TRUE |  | 205099_s_at | TRUE |
| 205111_s_at | TRUE |  | 205111_s_at | TRUE |
| 205112_at | TRUE |  | 205112_at | TRUE |
| 205128_x_at | TRUE |  | 205128_x_at | TRUE |
| 205140_at | TRUE |  | 205140_at | TRUE |
| 205143_at | TRUE |  | 205143_at | TRUE |
| 205147_x_at | TRUE |  | 205147_x_at | TRUE |
| 205152_at | TRUE |  | 205152_at | TRUE |
| 205159_at | TRUE |  | 205159_at | TRUE |
| 205173_x_at | TRUE |  | 205173_x_at | TRUE |
| 205176_s_at | TRUE |  | 205176_s_at | TRUE |
| 205184_at | TRUE |  | 205184_at | TRUE |
| 205186_at | TRUE |  | 205186_at | TRUE |
| 205232_s_at | TRUE |  | 205232_s_at | TRUE |
| 205233_s_at | TRUE |  | 205233_s_at | TRUE |
| 205245_at | TRUE |  | 205245_at | TRUE |
| 205269_at | TRUE |  | 205269_at | TRUE |
| 205285_s_at | TRUE |  | 205285_s_at | TRUE |
| 205288_at | TRUE |  | 205288_at | TRUE |
| 205289_at | TRUE |  | 205289_at | TRUE |
| 205290_s_at | TRUE |  | 205290_s_at | TRUE |
| 205312_at | TRUE |  | 205312_at | TRUE |
| 205322_s_at | TRUE |  | 205322_s_at | TRUE |
| 205336_at | TRUE |  | 205336_at | TRUE |
| 205349_at | TRUE |  | 205349_at | TRUE |
| 205382_s_at | TRUE |  | 205382_s_at | TRUE |
| 205436_s_at | TRUE |  | 205436_s_at | TRUE |
| 205437_at | TRUE |  | 205437_at | TRUE |
| 205465_x_at | TRUE |  | 205465_x_at | TRUE |
| 205466_s_at | TRUE |  | 205466_s_at | TRUE |
| 205467_at | TRUE |  | 205467_at | TRUE |
| 205479_s_at | TRUE |  | 205479_s_at | TRUE |
| 205504_at | TRUE |  | 205504_at | TRUE |
| 205509_at | TRUE |  | 205509_at | TRUE |
| 205524_s_at | TRUE |  | 205524_s_at | TRUE |
| 205545_x_at | TRUE |  | 205545_x_at | TRUE |
| 205573_s_at | TRUE |  | 205573_s_at | TRUE |
| 205630_at | TRUE |  | 205630_at | TRUE |
| 205638_at | TRUE |  | 205638_at | TRUE |
| 205639_at | TRUE |  | 205639_at | TRUE |
| 205640_at | TRUE |  | 205640_at | TRUE |
| 205668_at | TRUE |  | 205668_at | TRUE |
| 205685_at | TRUE |  | 205685_at | TRUE |
| 205686_s_at | TRUE |  | 205686_s_at | TRUE |
| 205715_at | TRUE |  | 205715_at | TRUE |
| 205740_s_at | TRUE |  | 205740_s_at | TRUE |
| 205786_s_at | TRUE |  | 205786_s_at | TRUE |
| 205791_x_at | TRUE |  | 205791_x_at | TRUE |
| 205806_at | TRUE |  | 205806_at | TRUE |
| 205822_s_at | TRUE |  | 205822_s_at | TRUE |
| 205854_at | TRUE |  | 205854_at | TRUE |
| 205859_at | TRUE |  | 205859_at | TRUE |
| 205898_at | TRUE |  | 205898_at | TRUE |
| 205945_at | TRUE |  | 205945_at | TRUE |
| 205952_at | TRUE |  | 205952_at | TRUE |
| 205988_at | TRUE |  | 205988_at | TRUE |
| 205990_s_at | TRUE |  | 205990_s_at | TRUE |
| 205996_s_at | TRUE |  | 205996_s_at | TRUE |
| 206011_at | TRUE |  | 206011_at | TRUE |
| 206013_s_at | TRUE |  | 206013_s_at | TRUE |
| 206014_at | TRUE |  | 206014_at | TRUE |
| 206015_s_at | TRUE |  | 206015_s_at | TRUE |
| 206018_at | TRUE |  | 206018_at | TRUE |
| 206034_at | TRUE |  | 206034_at | TRUE |
| 206101_at | TRUE |  | 206101_at | TRUE |
| 206111_at | TRUE |  | 206111_at | TRUE |
| 206137_at | TRUE |  | 206137_at | TRUE |
| 206167_s_at | TRUE |  | 206167_s_at | TRUE |
| 206171_at | TRUE |  | 206171_at | TRUE |
| 206219_s_at | TRUE |  | 206219_s_at | TRUE |
| 206220_s_at | TRUE |  | 206220_s_at | TRUE |
| 206247_at | TRUE |  | 206247_at | TRUE |
| 206254_at | TRUE |  | 206254_at | TRUE |
| 206274_s_at | TRUE |  | 206274_s_at | TRUE |
| 206278_at | TRUE |  | 206278_at | TRUE |
| 206283_s_at | TRUE |  | 206283_s_at | TRUE |
| 206295_at | TRUE |  | 206295_at | TRUE |
| 206363_at | TRUE |  | 206363_at | TRUE |
| 206370_at | TRUE |  | 206370_at | TRUE |
| 206383_s_at | TRUE |  | 206383_s_at | TRUE |
| 206404_at | TRUE |  | 206404_at | TRUE |
| 206420_at | TRUE |  | 206420_at | TRUE |
| 206480_at | TRUE |  | 206480_at | TRUE |
| 206522_at | TRUE |  | 206522_at | TRUE |
| 206527_at | TRUE |  | 206527_at | TRUE |
| 206584_at | TRUE |  | 206584_at | TRUE |
| 206657_s_at | TRUE |  | 206657_s_at | TRUE |
| 206687_s_at | TRUE |  | 206687_s_at | TRUE |
| 206711_at | TRUE |  | 206711_at | TRUE |
| 206715_at | TRUE |  | 206715_at | TRUE |
| 206726_at | TRUE |  | 206726_at | TRUE |
| 206772_at | TRUE |  | 206772_at | TRUE |
| 206885_x_at | TRUE |  | 206885_x_at | TRUE |
| 206972_s_at | TRUE |  | 206972_s_at | TRUE |
| 207030_s_at | TRUE |  | 207030_s_at | TRUE |
| 207039_at | TRUE |  | 207039_at | TRUE |
| 207060_at | TRUE |  | 207060_at | TRUE |
| 207075_at | TRUE |  | 207075_at | TRUE |
| 207085_x_at | TRUE |  | 207085_x_at | TRUE |
| 207104_x_at | TRUE |  | 207104_x_at | TRUE |
| 207156_at | TRUE |  | 207156_at | TRUE |
| 207157_s_at | TRUE |  | 207157_s_at | TRUE |
| 207164_s_at | TRUE |  | 207164_s_at | TRUE |
| 207170_s_at | TRUE |  | 207170_s_at | TRUE |
| 207196_s_at | TRUE |  | 207196_s_at | TRUE |
| 207232_s_at | TRUE |  | 207232_s_at | TRUE |
| 207233_s_at | TRUE |  | 207233_s_at | TRUE |
| 207238_s_at | TRUE |  | 207238_s_at | TRUE |
| 207266_x_at | TRUE |  | 207266_x_at | TRUE |
| 207358_x_at | TRUE |  | 207358_x_at | TRUE |
| 207361_at | TRUE |  | 207361_at | TRUE |
| 207375_s_at | TRUE |  | 207375_s_at | TRUE |
| 207428_x_at | TRUE |  | 207428_x_at | TRUE |
| 207440_at | TRUE |  | 207440_at | TRUE |
| 207455_at | TRUE |  | 207455_at | TRUE |
| 207530_s_at | TRUE |  | 207530_s_at | TRUE |
| 207540_s_at | TRUE |  | 207540_s_at | TRUE |
| 207541_s_at | TRUE |  | 207541_s_at | TRUE |
| 207545_s_at | TRUE |  | 207545_s_at | TRUE |
| 207655_s_at | TRUE |  | 207655_s_at | TRUE |
| 207658_s_at | TRUE |  | 207658_s_at | TRUE |
| 207677_s_at | TRUE |  | 207677_s_at | TRUE |
| 207691_x_at | TRUE |  | 207691_x_at | TRUE |
| 207697_x_at | TRUE |  | 207697_x_at | TRUE |
| 207801_s_at | TRUE |  | 207801_s_at | TRUE |
| 207805_s_at | TRUE |  | 207805_s_at | TRUE |
| 207857_at | TRUE |  | 207857_at | TRUE |
| 207872_s_at | TRUE |  | 207872_s_at | TRUE |
| 207873_x_at | TRUE |  | 207873_x_at | TRUE |
| 207876_s_at | TRUE |  | 207876_s_at | TRUE |
| 207907_at | TRUE |  | 207907_at | TRUE |
| 207981_s_at | TRUE |  | 207981_s_at | TRUE |
| 208017_s_at | TRUE |  | 208017_s_at | TRUE |
| 208018_s_at | TRUE |  | 208018_s_at | TRUE |
| 208064_s_at | TRUE |  | 208064_s_at | TRUE |
| 208065_at | TRUE |  | 208065_at | TRUE |
| 208066_s_at | TRUE |  | 208066_s_at | TRUE |
| 208068_x_at | TRUE |  | 208068_x_at | TRUE |
| 208071_s_at | TRUE |  | 208071_s_at | TRUE |
| 208110_x_at | TRUE |  | 208110_x_at | TRUE |
| 208130_s_at | TRUE |  | 208130_s_at | TRUE |
| 208258_s_at | TRUE |  | 208258_s_at | TRUE |
| 208306_x_at | TRUE |  | 208306_x_at | TRUE |
| 208374_s_at | TRUE |  | 208374_s_at | TRUE |
| 208387_s_at | TRUE |  | 208387_s_at | TRUE |
| 208427_s_at | TRUE |  | 208427_s_at | TRUE |
| 208451_s_at | TRUE |  | 208451_s_at | TRUE |
| 208491_s_at | TRUE |  | 208491_s_at | TRUE |
| 208523_x_at | TRUE |  | 208523_x_at | TRUE |
| 208540_x_at | TRUE |  | 208540_x_at | TRUE |
| 208546_x_at | TRUE |  | 208546_x_at | TRUE |
| 208609_s_at | TRUE |  | 208609_s_at | TRUE |
| 208615_s_at | TRUE |  | 208615_s_at | TRUE |
| 208628_s_at | TRUE |  | 208628_s_at | TRUE |
| 208633_s_at | TRUE |  | 208633_s_at | TRUE |
| 208634_s_at | TRUE |  | 208634_s_at | TRUE |
| 208659_at | TRUE |  | 208659_at | TRUE |
| 208661_s_at | TRUE |  | 208661_s_at | TRUE |
| 208664_s_at | TRUE |  | 208664_s_at | TRUE |
| 208674_x_at | TRUE |  | 208674_x_at | TRUE |
| 208709_s_at | TRUE |  | 208709_s_at | TRUE |
| 208711_s_at | TRUE |  | 208711_s_at | TRUE |
| 208712_at | TRUE |  | 208712_at | TRUE |
| 208713_at | TRUE |  | 208713_at | TRUE |
| 208728_s_at | TRUE |  | 208728_s_at | TRUE |
| 208784_s_at | TRUE |  | 208784_s_at | TRUE |
| 208816_x_at | TRUE |  | 208816_x_at | TRUE |
| 208846_s_at | TRUE |  | 208846_s_at | TRUE |
| 208858_s_at | TRUE |  | 208858_s_at | TRUE |
| 208894_at | TRUE |  | 208894_at | TRUE |
| 208918_s_at | TRUE |  | 208918_s_at | TRUE |
| 208919_s_at | TRUE |  | 208919_s_at | TRUE |
| 208923_at | TRUE |  | 208923_at | TRUE |
| 208967_s_at | TRUE |  | 208967_s_at | TRUE |
| 208970_s_at | TRUE |  | 208970_s_at | TRUE |
| 208997_s_at | TRUE |  | 208997_s_at | TRUE |
| 208998_at | TRUE |  | 208998_at | TRUE |
| 209090_s_at | TRUE |  | 209090_s_at | TRUE |
| 209129_at | TRUE |  | 209129_at | TRUE |
| 209130_at | TRUE |  | 209130_at | TRUE |
| 209159_s_at | TRUE |  | 209159_s_at | TRUE |
| 209166_s_at | TRUE |  | 209166_s_at | TRUE |
| 209187_at | TRUE |  | 209187_at | TRUE |
| 209191_at | TRUE |  | 209191_at | TRUE |
| 209206_at | TRUE |  | 209206_at | TRUE |
| 209207_s_at | TRUE |  | 209207_s_at | TRUE |
| 209215_at | TRUE |  | 209215_at | TRUE |
| 209218_at | TRUE |  | 209218_at | TRUE |
| 209269_s_at | TRUE |  | 209269_s_at | TRUE |
| 209279_s_at | TRUE |  | 209279_s_at | TRUE |
| 209282_at | TRUE |  | 209282_at | TRUE |
| 209312_x_at | TRUE |  | 209312_x_at | TRUE |
| 209348_s_at | TRUE |  | 209348_s_at | TRUE |
| 209360_s_at | TRUE |  | 209360_s_at | TRUE |
| 209367_at | TRUE |  | 209367_at | TRUE |
| 209459_s_at | TRUE |  | 209459_s_at | TRUE |
| 209460_at | TRUE |  | 209460_at | TRUE |
| 209467_s_at | TRUE |  | 209467_s_at | TRUE |
| 209469_at | TRUE |  | 209469_at | TRUE |
| 209470_s_at | TRUE |  | 209470_s_at | TRUE |
| 209473_at | TRUE |  | 209473_at | TRUE |
| 209534_x_at | TRUE |  | 209534_x_at | TRUE |
| 209570_s_at | TRUE |  | 209570_s_at | TRUE |
| 209608_s_at | TRUE |  | 209608_s_at | TRUE |
| 209619_at | TRUE |  | 209619_at | TRUE |
| 209669_s_at | TRUE |  | 209669_s_at | TRUE |
| 209707_at | TRUE |  | 209707_at | TRUE |
| 209709_s_at | TRUE |  | 209709_s_at | TRUE |
| 209711_at | TRUE |  | 209711_at | TRUE |
| 209712_at | TRUE |  | 209712_at | TRUE |
| 209716_at | TRUE |  | 209716_at | TRUE |
| 209728_at | TRUE |  | 209728_at | TRUE |
| 209732_at | TRUE |  | 209732_at | TRUE |
| 209734_at | TRUE |  | 209734_at | TRUE |
| 209827_s_at | TRUE |  | 209827_s_at | TRUE |
| 209868_s_at | TRUE |  | 209868_s_at | TRUE |
| 209879_at | TRUE |  | 209879_at | TRUE |
| 209901_x_at | TRUE |  | 209901_x_at | TRUE |
| 209906_at | TRUE |  | 209906_at | TRUE |
| 209914_s_at | TRUE |  | 209914_s_at | TRUE |
| 209915_s_at | TRUE |  | 209915_s_at | TRUE |
| 209933_s_at | TRUE |  | 209933_s_at | TRUE |
| 209960_at | TRUE |  | 209960_at | TRUE |
| 209966_x_at | TRUE |  | 209966_x_at | TRUE |
| 209970_x_at | TRUE |  | 209970_x_at | TRUE |
| 210015_s_at | TRUE |  | 210015_s_at | TRUE |
| 210042_s_at | TRUE |  | 210042_s_at | TRUE |
| 210044_s_at | TRUE |  | 210044_s_at | TRUE |
| 210089_s_at | TRUE |  | 210089_s_at | TRUE |
| 210093_s_at | TRUE |  | 210093_s_at | TRUE |
| 210101_x_at | TRUE |  | 210101_x_at | TRUE |
| 210113_s_at | TRUE |  | 210113_s_at | TRUE |
| 210114_at | TRUE |  | 210114_at | TRUE |
| 210123_s_at | TRUE |  | 210123_s_at | TRUE |
| 210145_at | TRUE |  | 210145_at | TRUE |
| 210162_s_at | TRUE |  | 210162_s_at | TRUE |
| 210166_at | TRUE |  | 210166_at | TRUE |
| 210176_at | TRUE |  | 210176_at | TRUE |
| 210184_at | TRUE |  | 210184_at | TRUE |
| 210241_s_at | TRUE |  | 210241_s_at | TRUE |
| 210246_s_at | TRUE |  | 210246_s_at | TRUE |
| 210252_s_at | TRUE |  | 210252_s_at | TRUE |
| 210287_s_at | TRUE |  | 210287_s_at | TRUE |
| 210340_s_at | TRUE |  | 210340_s_at | TRUE |
| 210423_s_at | TRUE |  | 210423_s_at | TRUE |
| 210427_x_at | TRUE |  | 210427_x_at | TRUE |
| 210436_at | TRUE |  | 210436_at | TRUE |
| 210466_s_at | TRUE |  | 210466_s_at | TRUE |
| 210560_at | TRUE |  | 210560_at | TRUE |
| 210582_s_at | TRUE |  | 210582_s_at | TRUE |
| 210593_at | TRUE |  | 210593_at | TRUE |
| 210607_at | TRUE |  | 210607_at | TRUE |
| 210629_x_at | TRUE |  | 210629_x_at | TRUE |
| 210644_s_at | TRUE |  | 210644_s_at | TRUE |
| 210645_s_at | TRUE |  | 210645_s_at | TRUE |
| 210649_s_at | TRUE |  | 210649_s_at | TRUE |
| 210660_at | TRUE |  | 210660_at | TRUE |
| 210697_at | TRUE |  | 210697_at | TRUE |
| 210743_s_at | TRUE |  | 210743_s_at | TRUE |
| 210754_s_at | TRUE |  | 210754_s_at | TRUE |
| 210886_x_at | TRUE |  | 210886_x_at | TRUE |
| 210895_s_at | TRUE |  | 210895_s_at | TRUE |
| 210912_x_at | TRUE |  | 210912_x_at | TRUE |
| 210982_s_at | TRUE |  | 210982_s_at | TRUE |
| 211101_x_at | TRUE |  | 211101_x_at | TRUE |
| 211105_s_at | TRUE |  | 211105_s_at | TRUE |
| 211110_s_at | TRUE |  | 211110_s_at | TRUE |
| 211126_s_at | TRUE |  | 211126_s_at | TRUE |
| 211190_x_at | TRUE |  | 211190_x_at | TRUE |
| 211192_s_at | TRUE |  | 211192_s_at | TRUE |
| 211207_s_at | TRUE |  | 211207_s_at | TRUE |
| 211217_s_at | TRUE |  | 211217_s_at | TRUE |
| 211241_at | TRUE |  | 211241_at | TRUE |
| 211286_x_at | TRUE |  | 211286_x_at | TRUE |
| 211289_x_at | TRUE |  | 211289_x_at | TRUE |
| 211336_x_at | TRUE |  | 211336_x_at | TRUE |
| 211366_x_at | TRUE |  | 211366_x_at | TRUE |
| 211367_s_at | TRUE |  | 211367_s_at | TRUE |
| 211368_s_at | TRUE |  | 211368_s_at | TRUE |
| 211423_s_at | TRUE |  | 211423_s_at | TRUE |
| 211429_s_at | TRUE |  | 211429_s_at | TRUE |
| 211507_s_at | TRUE |  | 211507_s_at | TRUE |
| 211552_s_at | TRUE |  | 211552_s_at | TRUE |
| 211581_x_at | TRUE |  | 211581_x_at | TRUE |
| 211582_x_at | TRUE |  | 211582_x_at | TRUE |
| 211602_s_at | TRUE |  | 211602_s_at | TRUE |
| 211615_s_at | TRUE |  | 211615_s_at | TRUE |
| 211621_at | TRUE |  | 211621_at | TRUE |
| 211661_x_at | TRUE |  | 211661_x_at | TRUE |
| 211725_s_at | TRUE |  | 211725_s_at | TRUE |
| 211733_x_at | TRUE |  | 211733_x_at | TRUE |
| 211742_s_at | TRUE |  | 211742_s_at | TRUE |
| 211794_at | TRUE |  | 211794_at | TRUE |
| 211795_s_at | TRUE |  | 211795_s_at | TRUE |
| 211799_x_at | TRUE |  | 211799_x_at | TRUE |
| 211864_s_at | TRUE |  | 211864_s_at | TRUE |
| 211894_x_at | TRUE |  | 211894_x_at | TRUE |
| 211950_at | TRUE |  | 211950_at | TRUE |
| 211958_at | TRUE |  | 211958_at | TRUE |
| 211959_at | TRUE |  | 211959_at | TRUE |
| 211962_s_at | TRUE |  | 211962_s_at | TRUE |
| 211990_at | TRUE |  | 211990_at | TRUE |
| 212005_at | TRUE |  | 212005_at | TRUE |
| 212017_at | TRUE |  | 212017_at | TRUE |
| 212049_at | TRUE |  | 212049_at | TRUE |
| 212062_at | TRUE |  | 212062_at | TRUE |
| 212101_at | TRUE |  | 212101_at | TRUE |
| 212112_s_at | TRUE |  | 212112_s_at | TRUE |
| 212119_at | TRUE |  | 212119_at | TRUE |
| 212122_at | TRUE |  | 212122_at | TRUE |
| 212131_at | TRUE |  | 212131_at | TRUE |
| 212132_at | TRUE |  | 212132_at | TRUE |
| 212152_x_at | TRUE |  | 212152_x_at | TRUE |
| 212174_at | TRUE |  | 212174_at | TRUE |
| 212175_s_at | TRUE |  | 212175_s_at | TRUE |
| 212184_s_at | TRUE |  | 212184_s_at | TRUE |
| 212186_at | TRUE |  | 212186_at | TRUE |
| 212226_s_at | TRUE |  | 212226_s_at | TRUE |
| 212230_at | TRUE |  | 212230_at | TRUE |
| 212279_at | TRUE |  | 212279_at | TRUE |
| 212281_s_at | TRUE |  | 212281_s_at | TRUE |
| 212282_at | TRUE |  | 212282_at | TRUE |
| 212291_at | TRUE |  | 212291_at | TRUE |
| 212293_at | TRUE |  | 212293_at | TRUE |
| 212294_at | TRUE |  | 212294_at | TRUE |
| 212300_at | TRUE |  | 212300_at | TRUE |
| 212337_at | TRUE |  | 212337_at | TRUE |
| 212345_s_at | TRUE |  | 212345_s_at | TRUE |
| 212377_s_at | TRUE |  | 212377_s_at | TRUE |
| 212383_at | TRUE |  | 212383_at | TRUE |
| 212388_at | TRUE |  | 212388_at | TRUE |
| 212395_s_at | TRUE |  | 212395_s_at | TRUE |
| 212401_s_at | TRUE |  | 212401_s_at | TRUE |
| 212491_s_at | TRUE |  | 212491_s_at | TRUE |
| 212587_s_at | TRUE |  | 212587_s_at | TRUE |
| 212588_at | TRUE |  | 212588_at | TRUE |
| 212600_s_at | TRUE |  | 212600_s_at | TRUE |
| 212607_at | TRUE |  | 212607_at | TRUE |
| 212628_at | TRUE |  | 212628_at | TRUE |
| 212629_s_at | TRUE |  | 212629_s_at | TRUE |
| 212641_at | TRUE |  | 212641_at | TRUE |
| 212658_at | TRUE |  | 212658_at | TRUE |
| 212663_at | TRUE |  | 212663_at | TRUE |
| 212671_s_at | TRUE |  | 212671_s_at | TRUE |
| 212695_at | TRUE |  | 212695_at | TRUE |
| 212704_at | TRUE |  | 212704_at | TRUE |
| 212725_s_at | TRUE |  | 212725_s_at | TRUE |
| 212730_at | TRUE |  | 212730_at | TRUE |
| 212743_at | TRUE |  | 212743_at | TRUE |
| 212765_at | TRUE |  | 212765_at | TRUE |
| 212768_s_at | TRUE |  | 212768_s_at | TRUE |
| 212774_at | TRUE |  | 212774_at | TRUE |
| 212788_x_at | TRUE |  | 212788_x_at | TRUE |
| 212873_at | TRUE |  | 212873_at | TRUE |
| 212875_s_at | TRUE |  | 212875_s_at | TRUE |
| 212893_at | TRUE |  | 212893_at | TRUE |
| 212906_at | TRUE |  | 212906_at | TRUE |
| 212920_at | TRUE |  | 212920_at | TRUE |
| 212958_x_at | TRUE |  | 212958_x_at | TRUE |
| 213053_at | TRUE |  | 213053_at | TRUE |
| 213060_s_at | TRUE |  | 213060_s_at | TRUE |
| 213076_at | TRUE |  | 213076_at | TRUE |
| 213095_x_at | TRUE |  | 213095_x_at | TRUE |
| 213111_at | TRUE |  | 213111_at | TRUE |
| 213160_at | TRUE |  | 213160_at | TRUE |
| 213170_at | TRUE |  | 213170_at | TRUE |
| 213171_s_at | TRUE |  | 213171_s_at | TRUE |
| 213186_at | TRUE |  | 213186_at | TRUE |
| 213187_x_at | TRUE |  | 213187_x_at | TRUE |
| 213278_at | TRUE |  | 213278_at | TRUE |
| 213281_at | TRUE |  | 213281_at | TRUE |
| 213296_at | TRUE |  | 213296_at | TRUE |
| 213308_at | TRUE |  | 213308_at | TRUE |
| 213309_at | TRUE |  | 213309_at | TRUE |
| 213373_s_at | TRUE |  | 213373_s_at | TRUE |
| 213397_x_at | TRUE |  | 213397_x_at | TRUE |
| 213405_at | TRUE |  | 213405_at | TRUE |
| 213407_at | TRUE |  | 213407_at | TRUE |
| 213411_at | TRUE |  | 213411_at | TRUE |
| 213415_at | TRUE |  | 213415_at | TRUE |
| 213425_at | TRUE |  | 213425_at | TRUE |
| 213486_at | TRUE |  | 213486_at | TRUE |
| 213497_at | TRUE |  | 213497_at | TRUE |
| 213503_x_at | TRUE |  | 213503_x_at | TRUE |
| 213506_at | TRUE |  | 213506_at | TRUE |
| 213521_at | TRUE |  | 213521_at | TRUE |
| 213566_at | TRUE |  | 213566_at | TRUE |
| 213572_s_at | TRUE |  | 213572_s_at | TRUE |
| 213601_at | TRUE |  | 213601_at | TRUE |
| 213603_s_at | TRUE |  | 213603_s_at | TRUE |
| 213604_at | TRUE |  | 213604_at | TRUE |
| 213607_x_at | TRUE |  | 213607_x_at | TRUE |
| 213609_s_at | TRUE |  | 213609_s_at | TRUE |
| 213676_at | TRUE |  | 213676_at | TRUE |
| 213678_at | TRUE |  | 213678_at | TRUE |
| 213683_at | TRUE |  | 213683_at | TRUE |
| 213695_at | TRUE |  | 213695_at | TRUE |
| 213698_at | TRUE |  | 213698_at | TRUE |
| 213716_s_at | TRUE |  | 213716_s_at | TRUE |
| 213733_at | TRUE |  | 213733_at | TRUE |
| 213742_at | TRUE |  | 213742_at | TRUE |
| 213798_s_at | TRUE |  | 213798_s_at | TRUE |
| 213804_at | TRUE |  | 213804_at | TRUE |
| 213813_x_at | TRUE |  | 213813_x_at | TRUE |
| 213883_s_at | TRUE |  | 213883_s_at | TRUE |
| 213888_s_at | TRUE |  | 213888_s_at | TRUE |
| 213895_at | TRUE |  | 213895_at | TRUE |
| 213975_s_at | TRUE |  | 213975_s_at | TRUE |
| 213982_s_at | TRUE |  | 213982_s_at | TRUE |
| 214000_s_at | TRUE |  | 214000_s_at | TRUE |
| 214020_x_at | TRUE |  | 214020_x_at | TRUE |
| 214021_x_at | TRUE |  | 214021_x_at | TRUE |
| 214084_x_at | TRUE |  | 214084_x_at | TRUE |
| 214104_at | TRUE |  | 214104_at | TRUE |
| 214128_at | TRUE |  | 214128_at | TRUE |
| 214179_s_at | TRUE |  | 214179_s_at | TRUE |
| 214181_x_at | TRUE |  | 214181_x_at | TRUE |
| 214290_s_at | TRUE |  | 214290_s_at | TRUE |
| 214292_at | TRUE |  | 214292_at | TRUE |
| 214364_at | TRUE |  | 214364_at | TRUE |
| 214366_s_at | TRUE |  | 214366_s_at | TRUE |
| 214376_at | TRUE |  | 214376_at | TRUE |
| 214383_x_at | TRUE |  | 214383_x_at | TRUE |
| 214428_x_at | TRUE |  | 214428_x_at | TRUE |
| 214438_at | TRUE |  | 214438_at | TRUE |
| 214467_at | TRUE |  | 214467_at | TRUE |
| 214495_at | TRUE |  | 214495_at | TRUE |
| 214511_x_at | TRUE |  | 214511_x_at | TRUE |
| 214574_x_at | TRUE |  | 214574_x_at | TRUE |
| 214597_at | TRUE |  | 214597_at | TRUE |
| 214620_x_at | TRUE |  | 214620_x_at | TRUE |
| 214622_at | TRUE |  | 214622_at | TRUE |
| 214752_x_at | TRUE |  | 214752_x_at | TRUE |
| 214770_at | TRUE |  | 214770_at | TRUE |
| 214778_at | TRUE |  | 214778_at | TRUE |
| 214791_at | TRUE |  | 214791_at | TRUE |
| 214807_at | TRUE |  | 214807_at | TRUE |
| 214825_at | TRUE |  | 214825_at | TRUE |
| 214840_at | TRUE |  | 214840_at | TRUE |
| 214879_x_at | TRUE |  | 214879_x_at | TRUE |
| 214884_at | TRUE |  | 214884_at | TRUE |
| 214909_s_at | TRUE |  | 214909_s_at | TRUE |
| 214939_x_at | TRUE |  | 214939_x_at | TRUE |
| 214949_at | TRUE |  | 214949_at | TRUE |
| 214993_at | TRUE |  | 214993_at | TRUE |
| 215046_at | TRUE |  | 215046_at | TRUE |
| 215051_x_at | TRUE |  | 215051_x_at | TRUE |
| 215064_at | TRUE |  | 215064_at | TRUE |
| 215071_s_at | TRUE |  | 215071_s_at | TRUE |
| 215084_s_at | TRUE |  | 215084_s_at | TRUE |
| 215087_at | TRUE |  | 215087_at | TRUE |
| 215127_s_at | TRUE |  | 215127_s_at | TRUE |
| 215193_x_at | TRUE |  | 215193_x_at | TRUE |
| 215252_at | TRUE |  | 215252_at | TRUE |
| 215285_s_at | TRUE |  | 215285_s_at | TRUE |
| 215364_s_at | TRUE |  | 215364_s_at | TRUE |
| 215419_at | TRUE |  | 215419_at | TRUE |
| 215448_at | TRUE |  | 215448_at | TRUE |
| 215498_s_at | TRUE |  | 215498_s_at | TRUE |
| 215499_at | TRUE |  | 215499_at | TRUE |
| 215537_x_at | TRUE |  | 215537_x_at | TRUE |
| 215566_x_at | TRUE |  | 215566_x_at | TRUE |
| 215617_at | TRUE |  | 215617_at | TRUE |
| 215633_x_at | TRUE |  | 215633_x_at | TRUE |
| 215635_at | TRUE |  | 215635_at | TRUE |
| 215691_x_at | TRUE |  | 215691_x_at | TRUE |
| 215737_x_at | TRUE |  | 215737_x_at | TRUE |
| 215783_s_at | TRUE |  | 215783_s_at | TRUE |
| 215813_s_at | TRUE |  | 215813_s_at | TRUE |
| 215933_s_at | TRUE |  | 215933_s_at | TRUE |
| 216047_x_at | TRUE |  | 216047_x_at | TRUE |
| 216061_x_at | TRUE |  | 216061_x_at | TRUE |
| 216073_at | TRUE |  | 216073_at | TRUE |
| 216184_s_at | TRUE |  | 216184_s_at | TRUE |
| 216221_s_at | TRUE |  | 216221_s_at | TRUE |
| 216224_s_at | TRUE |  | 216224_s_at | TRUE |
| 216235_s_at | TRUE |  | 216235_s_at | TRUE |
| 216250_s_at | TRUE |  | 216250_s_at | TRUE |
| 216255_s_at | TRUE |  | 216255_s_at | TRUE |
| 216264_s_at | TRUE |  | 216264_s_at | TRUE |
| 216438_s_at | TRUE |  | 216438_s_at | TRUE |
| 216457_s_at | TRUE |  | 216457_s_at | TRUE |
| 216598_s_at | TRUE |  | 216598_s_at | TRUE |
| 216606_x_at | TRUE |  | 216606_x_at | TRUE |
| 216672_s_at | TRUE |  | 216672_s_at | TRUE |
| 216705_s_at | TRUE |  | 216705_s_at | TRUE |
| 216850_at | TRUE |  | 216850_at | TRUE |
| 216873_s_at | TRUE |  | 216873_s_at | TRUE |
| 216933_x_at | TRUE |  | 216933_x_at | TRUE |
| 216942_s_at | TRUE |  | 216942_s_at | TRUE |
| 216945_x_at | TRUE |  | 216945_x_at | TRUE |
| 217004_s_at | TRUE |  | 217004_s_at | TRUE |
| 217066_s_at | TRUE |  | 217066_s_at | TRUE |
| 217078_s_at | TRUE |  | 217078_s_at | TRUE |
| 217173_s_at | TRUE |  | 217173_s_at | TRUE |
| 217196_s_at | TRUE |  | 217196_s_at | TRUE |
| 217208_s_at | TRUE |  | 217208_s_at | TRUE |
| 217436_x_at | TRUE |  | 217436_x_at | TRUE |
| 217455_s_at | TRUE |  | 217455_s_at | TRUE |
| 217456_x_at | TRUE |  | 217456_x_at | TRUE |
| 217478_s_at | TRUE |  | 217478_s_at | TRUE |
| 217572_at | TRUE |  | 217572_at | TRUE |
| 217722_s_at | TRUE |  | 217722_s_at | TRUE |
| 217757_at | TRUE |  | 217757_at | TRUE |
| 217767_at | TRUE |  | 217767_at | TRUE |
| 217778_at | TRUE |  | 217778_at | TRUE |
| 217779_s_at | TRUE |  | 217779_s_at | TRUE |
| 217844_at | TRUE |  | 217844_at | TRUE |
| 217855_x_at | TRUE |  | 217855_x_at | TRUE |
| 217865_at | TRUE |  | 217865_at | TRUE |
| 217877_s_at | TRUE |  | 217877_s_at | TRUE |
| 217893_s_at | TRUE |  | 217893_s_at | TRUE |
| 217911_s_at | TRUE |  | 217911_s_at | TRUE |
| 217923_at | TRUE |  | 217923_at | TRUE |
| 217929_s_at | TRUE |  | 217929_s_at | TRUE |
| 217933_s_at | TRUE |  | 217933_s_at | TRUE |
| 217944_at | TRUE |  | 217944_at | TRUE |
| 217947_at | TRUE |  | 217947_at | TRUE |
| 217950_at | TRUE |  | 217950_at | TRUE |
| 217966_s_at | TRUE |  | 217966_s_at | TRUE |
| 217979_at | TRUE |  | 217979_at | TRUE |
| 217983_s_at | TRUE |  | 217983_s_at | TRUE |
| 217984_at | TRUE |  | 217984_at | TRUE |
| 217994_x_at | TRUE |  | 217994_x_at | TRUE |
| 217995_at | TRUE |  | 217995_at | TRUE |
| 218022_at | TRUE |  | 218022_at | TRUE |
| 218035_s_at | TRUE |  | 218035_s_at | TRUE |
| 218037_at | TRUE |  | 218037_at | TRUE |
| 218048_at | TRUE |  | 218048_at | TRUE |
| 218080_x_at | TRUE |  | 218080_x_at | TRUE |
| 218088_s_at | TRUE |  | 218088_s_at | TRUE |
| 218162_at | TRUE |  | 218162_at | TRUE |
| 218194_at | TRUE |  | 218194_at | TRUE |
| 218197_s_at | TRUE |  | 218197_s_at | TRUE |
| 218204_s_at | TRUE |  | 218204_s_at | TRUE |
| 218232_at | TRUE |  | 218232_at | TRUE |
| 218280_x_at | TRUE |  | 218280_x_at | TRUE |
| 218299_at | TRUE |  | 218299_at | TRUE |
| 218306_s_at | TRUE |  | 218306_s_at | TRUE |
| 218332_at | TRUE |  | 218332_at | TRUE |
| 218341_at | TRUE |  | 218341_at | TRUE |
| 218368_s_at | TRUE |  | 218368_s_at | TRUE |
| 218370_s_at | TRUE |  | 218370_s_at | TRUE |
| 218409_s_at | TRUE |  | 218409_s_at | TRUE |
| 218424_s_at | TRUE |  | 218424_s_at | TRUE |
| 218433_at | TRUE |  | 218433_at | TRUE |
| 218450_at | TRUE |  | 218450_at | TRUE |
| 218462_at | TRUE |  | 218462_at | TRUE |
| 218550_s_at | TRUE |  | 218550_s_at | TRUE |
| 218551_at | TRUE |  | 218551_at | TRUE |
| 218574_s_at | TRUE |  | 218574_s_at | TRUE |
| 218583_s_at | TRUE |  | 218583_s_at | TRUE |
| 218589_at | TRUE |  | 218589_at | TRUE |
| 218623_at | TRUE |  | 218623_at | TRUE |
| 218632_at | TRUE |  | 218632_at | TRUE |
| 218684_at | TRUE |  | 218684_at | TRUE |
| 218711_s_at | TRUE |  | 218711_s_at | TRUE |
| 218712_at | TRUE |  | 218712_at | TRUE |
| 218717_s_at | TRUE |  | 218717_s_at | TRUE |
| 218720_x_at | TRUE |  | 218720_x_at | TRUE |
| 218770_s_at | TRUE |  | 218770_s_at | TRUE |
| 218831_s_at | TRUE |  | 218831_s_at | TRUE |
| 218839_at | TRUE |  | 218839_at | TRUE |
| 218865_at | TRUE |  | 218865_at | TRUE |
| 218870_at | TRUE |  | 218870_at | TRUE |
| 218882_s_at | TRUE |  | 218882_s_at | TRUE |
| 218913_s_at | TRUE |  | 218913_s_at | TRUE |
| 218917_s_at | TRUE |  | 218917_s_at | TRUE |
| 218924_s_at | TRUE |  | 218924_s_at | TRUE |
| 218932_at | TRUE |  | 218932_at | TRUE |
| 218952_at | TRUE |  | 218952_at | TRUE |
| 218977_s_at | TRUE |  | 218977_s_at | TRUE |
| 218994_s_at | TRUE |  | 218994_s_at | TRUE |
| 219011_at | TRUE |  | 219011_at | TRUE |
| 219090_at | TRUE |  | 219090_at | TRUE |
| 219103_at | TRUE |  | 219103_at | TRUE |
| 219126_at | TRUE |  | 219126_at | TRUE |
| 219183_s_at | TRUE |  | 219183_s_at | TRUE |
| 219191_s_at | TRUE |  | 219191_s_at | TRUE |
| 219202_at | TRUE |  | 219202_at | TRUE |
| 219230_at | TRUE |  | 219230_at | TRUE |
| 219235_s_at | TRUE |  | 219235_s_at | TRUE |
| 219243_at | TRUE |  | 219243_at | TRUE |
| 219284_at | TRUE |  | 219284_at | TRUE |
| 219286_s_at | TRUE |  | 219286_s_at | TRUE |
| 219316_s_at | TRUE |  | 219316_s_at | TRUE |
| 219322_s_at | TRUE |  | 219322_s_at | TRUE |
| 219344_at | TRUE |  | 219344_at | TRUE |
| 219358_s_at | TRUE |  | 219358_s_at | TRUE |
| 219375_at | TRUE |  | 219375_at | TRUE |
| 219382_at | TRUE |  | 219382_at | TRUE |
| 219409_at | TRUE |  | 219409_at | TRUE |
| 219424_at | TRUE |  | 219424_at | TRUE |
| 219426_at | TRUE |  | 219426_at | TRUE |
| 219441_s_at | TRUE |  | 219441_s_at | TRUE |
| 219505_at | TRUE |  | 219505_at | TRUE |
| 219509_at | TRUE |  | 219509_at | TRUE |
| 219570_at | TRUE |  | 219570_at | TRUE |
| 219593_at | TRUE |  | 219593_at | TRUE |
| 219603_s_at | TRUE |  | 219603_s_at | TRUE |
| 219619_at | TRUE |  | 219619_at | TRUE |
| 219620_x_at | TRUE |  | 219620_x_at | TRUE |
| 219666_at | TRUE |  | 219666_at | TRUE |
| 219690_at | TRUE |  | 219690_at | TRUE |
| 219725_at | TRUE |  | 219725_at | TRUE |
| 219740_at | TRUE |  | 219740_at | TRUE |
| 219758_at | TRUE |  | 219758_at | TRUE |
| 219807_x_at | TRUE |  | 219807_x_at | TRUE |
| 219815_at | TRUE |  | 219815_at | TRUE |
| 219818_s_at | TRUE |  | 219818_s_at | TRUE |
| 219826_at | TRUE |  | 219826_at | TRUE |
| 219837_s_at | TRUE |  | 219837_s_at | TRUE |
| 219843_at | TRUE |  | 219843_at | TRUE |
| 219848_s_at | TRUE |  | 219848_s_at | TRUE |
| 219877_at | TRUE |  | 219877_at | TRUE |
| 219894_at | TRUE |  | 219894_at | TRUE |
| 219939_s_at | TRUE |  | 219939_s_at | TRUE |
| 219994_at | TRUE |  | 219994_at | TRUE |
| 220005_at | TRUE |  | 220005_at | TRUE |
| 220033_at | TRUE |  | 220033_at | TRUE |
| 220072_at | TRUE |  | 220072_at | TRUE |
| 220079_s_at | TRUE |  | 220079_s_at | TRUE |
| 220088_at | TRUE |  | 220088_at | TRUE |
| 220103_s_at | TRUE |  | 220103_s_at | TRUE |
| 220146_at | TRUE |  | 220146_at | TRUE |
| 220157_x_at | TRUE |  | 220157_x_at | TRUE |
| 220162_s_at | TRUE |  | 220162_s_at | TRUE |
| 220206_at | TRUE |  | 220206_at | TRUE |
| 220324_at | TRUE |  | 220324_at | TRUE |
| 220361_at | TRUE |  | 220361_at | TRUE |
| 220387_s_at | TRUE |  | 220387_s_at | TRUE |
| 220416_at | TRUE |  | 220416_at | TRUE |
| 220434_at | TRUE |  | 220434_at | TRUE |
| 220462_at | TRUE |  | 220462_at | TRUE |
| 220585_at | TRUE |  | 220585_at | TRUE |
| 220731_s_at | TRUE |  | 220731_s_at | TRUE |
| 220750_s_at | TRUE |  | 220750_s_at | TRUE |
| 220769_s_at | TRUE |  | 220769_s_at | TRUE |
| 220911_s_at | TRUE |  | 220911_s_at | TRUE |
| 220998_s_at | TRUE |  | 220998_s_at | TRUE |
| 221047_s_at | TRUE |  | 221047_s_at | TRUE |
| 221123_x_at | TRUE |  | 221123_x_at | TRUE |
| 221178_at | TRUE |  | 221178_at | TRUE |
| 221204_s_at | TRUE |  | 221204_s_at | TRUE |
| 221269_s_at | TRUE |  | 221269_s_at | TRUE |
| 221293_s_at | TRUE |  | 221293_s_at | TRUE |
| 221321_s_at | TRUE |  | 221321_s_at | TRUE |
| 221427_s_at | TRUE |  | 221427_s_at | TRUE |
| 221435_x_at | TRUE |  | 221435_x_at | TRUE |
| 221476_s_at | TRUE |  | 221476_s_at | TRUE |
| 221486_at | TRUE |  | 221486_at | TRUE |
| 221512_at | TRUE |  | 221512_at | TRUE |
| 221530_s_at | TRUE |  | 221530_s_at | TRUE |
| 221581_s_at | TRUE |  | 221581_s_at | TRUE |
| 221666_s_at | TRUE |  | 221666_s_at | TRUE |
| 221698_s_at | TRUE |  | 221698_s_at | TRUE |
| 221710_x_at | TRUE |  | 221710_x_at | TRUE |
| 221725_at | TRUE |  | 221725_at | TRUE |
| 221727_at | TRUE |  | 221727_at | TRUE |
| 221750_at | TRUE |  | 221750_at | TRUE |
| 221773_at | TRUE |  | 221773_at | TRUE |
| 221875_x_at | TRUE |  | 221875_x_at | TRUE |
| 221898_at | TRUE |  | 221898_at | TRUE |
| 221900_at | TRUE |  | 221900_at | TRUE |
| 221923_s_at | TRUE |  | 221923_s_at | TRUE |
| 221958_s_at | TRUE |  | 221958_s_at | TRUE |
| 221972_s_at | TRUE |  | 221972_s_at | TRUE |
| 221984_s_at | TRUE |  | 221984_s_at | TRUE |
| 222000_at | TRUE |  | 222000_at | TRUE |
| 222024_s_at | TRUE |  | 222024_s_at | TRUE |
| 222028_at | TRUE |  | 222028_at | TRUE |
| 222031_at | TRUE |  | 222031_at | TRUE |
| 222099_s_at | TRUE |  | 222099_s_at | TRUE |
| 222145_at | TRUE |  | 222145_at | TRUE |
| 222153_at | TRUE |  | 222153_at | TRUE |
| 222154_s_at | TRUE |  | 222154_s_at | TRUE |
| 222170_at | TRUE |  | 222170_at | TRUE |
| 222199_s_at | TRUE |  | 222199_s_at | TRUE |
| 222212_s_at | TRUE |  | 222212_s_at | TRUE |
| 222236_s_at | TRUE |  | 222236_s_at | TRUE |
| 222244_s_at | TRUE |  | 222244_s_at | TRUE |
| 222297_x_at | TRUE |  | 222297_x_at | TRUE |
| 222322_at | TRUE |  | 222322_at | TRUE |
| 222401_s_at | TRUE |  | 222401_s_at | TRUE |
| 222430_s_at | TRUE |  | 222430_s_at | TRUE |
| 222440_s_at | TRUE |  | 222440_s_at | TRUE |
| 222452_s_at | TRUE |  | 222452_s_at | TRUE |
| 222459_at | TRUE |  | 222459_at | TRUE |
| 222478_at | TRUE |  | 222478_at | TRUE |
| 222495_at | TRUE |  | 222495_at | TRUE |
| 222496_s_at | TRUE |  | 222496_s_at | TRUE |
| 222506_at | TRUE |  | 222506_at | TRUE |
| 222528_s_at | TRUE |  | 222528_s_at | TRUE |
| 222579_at | TRUE |  | 222579_at | TRUE |
| 222621_at | TRUE |  | 222621_at | TRUE |
| 222640_at | TRUE |  | 222640_at | TRUE |
| 222717_at | TRUE |  | 222717_at | TRUE |
| 222775_s_at | TRUE |  | 222775_s_at | TRUE |
| 222790_s_at | TRUE |  | 222790_s_at | TRUE |
| 222791_at | TRUE |  | 222791_at | TRUE |
| 222800_at | TRUE |  | 222800_at | TRUE |
| 222833_at | TRUE |  | 222833_at | TRUE |
| 222834_s_at | TRUE |  | 222834_s_at | TRUE |
| 222866_s_at | TRUE |  | 222866_s_at | TRUE |
| 222868_s_at | TRUE |  | 222868_s_at | TRUE |
| 222872_x_at | TRUE |  | 222872_x_at | TRUE |
| 222876_s_at | TRUE |  | 222876_s_at | TRUE |
| 222889_at | TRUE |  | 222889_at | TRUE |
| 222929_at | TRUE |  | 222929_at | TRUE |
| 222975_s_at | TRUE |  | 222975_s_at | TRUE |
| 223015_at | TRUE |  | 223015_at | TRUE |
| 223017_at | TRUE |  | 223017_at | TRUE |
| 223051_at | TRUE |  | 223051_at | TRUE |
| 223053_x_at | TRUE |  | 223053_x_at | TRUE |
| 223077_at | TRUE |  | 223077_at | TRUE |
| 223097_at | TRUE |  | 223097_at | TRUE |
| 223119_s_at | TRUE |  | 223119_s_at | TRUE |
| 223120_at | TRUE |  | 223120_at | TRUE |
| 223153_x_at | TRUE |  | 223153_x_at | TRUE |
| 223158_s_at | TRUE |  | 223158_s_at | TRUE |
| 223175_s_at | TRUE |  | 223175_s_at | TRUE |
| 223185_s_at | TRUE |  | 223185_s_at | TRUE |
| 223223_at | TRUE |  | 223223_at | TRUE |
| 223234_at | TRUE |  | 223234_at | TRUE |
| 223280_x_at | TRUE |  | 223280_x_at | TRUE |
| 223303_at | TRUE |  | 223303_at | TRUE |
| 223331_s_at | TRUE |  | 223331_s_at | TRUE |
| 223335_at | TRUE |  | 223335_at | TRUE |
| 223402_at | TRUE |  | 223402_at | TRUE |
| 223452_s_at | TRUE |  | 223452_s_at | TRUE |
| 223456_s_at | TRUE |  | 223456_s_at | TRUE |
| 223500_at | TRUE |  | 223500_at | TRUE |
| 223529_at | TRUE |  | 223529_at | TRUE |
| 223536_at | TRUE |  | 223536_at | TRUE |
| 223543_at | TRUE |  | 223543_at | TRUE |
| 223553_s_at | TRUE |  | 223553_s_at | TRUE |
| 223562_at | TRUE |  | 223562_at | TRUE |
| 223583_at | TRUE |  | 223583_at | TRUE |
| 223615_at | TRUE |  | 223615_at | TRUE |
| 223617_x_at | TRUE |  | 223617_x_at | TRUE |
| 223640_at | TRUE |  | 223640_at | TRUE |
| 223680_at | TRUE |  | 223680_at | TRUE |
| 223692_at | TRUE |  | 223692_at | TRUE |
| 223703_at | TRUE |  | 223703_at | TRUE |
| 223727_at | TRUE |  | 223727_at | TRUE |
| 223750_s_at | TRUE |  | 223750_s_at | TRUE |
| 223766_at | TRUE |  | 223766_at | TRUE |
| 223767_at | TRUE |  | 223767_at | TRUE |
| 223769_x_at | TRUE |  | 223769_x_at | TRUE |
| 223773_s_at | TRUE |  | 223773_s_at | TRUE |
| 223774_at | TRUE |  | 223774_at | TRUE |
| 223783_s_at | TRUE |  | 223783_s_at | TRUE |
| 223790_at | TRUE |  | 223790_at | TRUE |
| 223809_at | TRUE |  | 223809_at | TRUE |
| 223849_s_at | TRUE |  | 223849_s_at | TRUE |
| 223852_s_at | TRUE |  | 223852_s_at | TRUE |
| 223879_s_at | TRUE |  | 223879_s_at | TRUE |
| 223892_s_at | TRUE |  | 223892_s_at | TRUE |
| 223922_x_at | TRUE |  | 223922_x_at | TRUE |
| 224018_s_at | TRUE |  | 224018_s_at | TRUE |
| 224046_s_at | TRUE |  | 224046_s_at | TRUE |
| 224190_x_at | TRUE |  | 224190_x_at | TRUE |
| 224217_s_at | TRUE |  | 224217_s_at | TRUE |
| 224252_s_at | TRUE |  | 224252_s_at | TRUE |
| 224280_s_at | TRUE |  | 224280_s_at | TRUE |
| 224281_s_at | TRUE |  | 224281_s_at | TRUE |
| 224301_x_at | TRUE |  | 224301_x_at | TRUE |
| 224315_at | TRUE |  | 224315_at | TRUE |
| 224356_x_at | TRUE |  | 224356_x_at | TRUE |
| 224374_s_at | TRUE |  | 224374_s_at | TRUE |
| 224393_s_at | TRUE |  | 224393_s_at | TRUE |
| 224451_x_at | TRUE |  | 224451_x_at | TRUE |
| 224472_x_at | TRUE |  | 224472_x_at | TRUE |
| 224562_at | TRUE |  | 224562_at | TRUE |
| 224563_at | TRUE |  | 224563_at | TRUE |
| 224578_at | TRUE |  | 224578_at | TRUE |
| 224591_at | TRUE |  | 224591_at | TRUE |
| 224592_x_at | TRUE |  | 224592_x_at | TRUE |
| 224624_at | TRUE |  | 224624_at | TRUE |
| 224659_at | TRUE |  | 224659_at | TRUE |
| 224706_at | TRUE |  | 224706_at | TRUE |
| 224708_at | TRUE |  | 224708_at | TRUE |
| 224735_at | TRUE |  | 224735_at | TRUE |
| 224772_at | TRUE |  | 224772_at | TRUE |
| 224773_at | TRUE |  | 224773_at | TRUE |
| 224796_at | TRUE |  | 224796_at | TRUE |
| 224807_at | TRUE |  | 224807_at | TRUE |
| 224820_at | TRUE |  | 224820_at | TRUE |
| 224846_at | TRUE |  | 224846_at | TRUE |
| 224884_at | TRUE |  | 224884_at | TRUE |
| 224893_at | TRUE |  | 224893_at | TRUE |
| 224901_at | TRUE |  | 224901_at | TRUE |
| 224906_at | TRUE |  | 224906_at | TRUE |
| 224912_at | TRUE |  | 224912_at | TRUE |
| 224916_at | TRUE |  | 224916_at | TRUE |
| 224921_at | TRUE |  | 224921_at | TRUE |
| 224923_at | TRUE |  | 224923_at | TRUE |
| 224929_at | TRUE |  | 224929_at | TRUE |
| 224954_at | TRUE |  | 224954_at | TRUE |
| 224959_at | TRUE |  | 224959_at | TRUE |
| 224970_at | TRUE |  | 224970_at | TRUE |
| 224975_at | TRUE |  | 224975_at | TRUE |
| 224989_at | TRUE |  | 224989_at | TRUE |
| 225000_at | TRUE |  | 225000_at | TRUE |
| 225005_at | TRUE |  | 225005_at | TRUE |
| 225016_at | TRUE |  | 225016_at | TRUE |
| 225056_at | TRUE |  | 225056_at | TRUE |
| 225059_at | TRUE |  | 225059_at | TRUE |
| 225130_at | TRUE |  | 225130_at | TRUE |
| 225136_at | TRUE |  | 225136_at | TRUE |
| 225208_s_at | TRUE |  | 225208_s_at | TRUE |
| 225209_s_at | TRUE |  | 225209_s_at | TRUE |
| 225217_s_at | TRUE |  | 225217_s_at | TRUE |
| 225220_at | TRUE |  | 225220_at | TRUE |
| 225225_at | TRUE |  | 225225_at | TRUE |
| 225228_at | TRUE |  | 225228_at | TRUE |
| 225230_at | TRUE |  | 225230_at | TRUE |
| 225245_x_at | TRUE |  | 225245_x_at | TRUE |
| 225255_at | TRUE |  | 225255_at | TRUE |
| 225257_at | TRUE |  | 225257_at | TRUE |
| 225265_at | TRUE |  | 225265_at | TRUE |
| 225269_s_at | TRUE |  | 225269_s_at | TRUE |
| 225288_at | TRUE |  | 225288_at | TRUE |
| 225292_at | TRUE |  | 225292_at | TRUE |
| 225293_at | TRUE |  | 225293_at | TRUE |
| 225323_at | TRUE |  | 225323_at | TRUE |
| 225328_at | TRUE |  | 225328_at | TRUE |
| 225332_at | TRUE |  | 225332_at | TRUE |
| 225345_s_at | TRUE |  | 225345_s_at | TRUE |
| 225353_s_at | TRUE |  | 225353_s_at | TRUE |
| 225356_at | TRUE |  | 225356_at | TRUE |
| 225358_at | TRUE |  | 225358_at | TRUE |
| 225373_at | TRUE |  | 225373_at | TRUE |
| 225384_at | TRUE |  | 225384_at | TRUE |
| 225401_at | TRUE |  | 225401_at | TRUE |
| 225475_at | TRUE |  | 225475_at | TRUE |
| 225502_at | TRUE |  | 225502_at | TRUE |
| 225520_at | TRUE |  | 225520_at | TRUE |
| 225577_at | TRUE |  | 225577_at | TRUE |
| 225579_at | TRUE |  | 225579_at | TRUE |
| 225593_at | TRUE |  | 225593_at | TRUE |
| 225605_at | TRUE |  | 225605_at | TRUE |
| 225627_s_at | TRUE |  | 225627_s_at | TRUE |
| 225646_at | TRUE |  | 225646_at | TRUE |
| 225755_at | TRUE |  | 225755_at | TRUE |
| 225763_at | TRUE |  | 225763_at | TRUE |
| 225782_at | TRUE |  | 225782_at | TRUE |
| 225790_at | TRUE |  | 225790_at | TRUE |
| 225803_at | TRUE |  | 225803_at | TRUE |
| 225806_at | TRUE |  | 225806_at | TRUE |
| 225807_at | TRUE |  | 225807_at | TRUE |
| 225869_s_at | TRUE |  | 225869_s_at | TRUE |
| 225878_at | TRUE |  | 225878_at | TRUE |
| 225890_at | TRUE |  | 225890_at | TRUE |
| 225900_at | TRUE |  | 225900_at | TRUE |
| 225992_at | TRUE |  | 225992_at | TRUE |
| 226000_at | TRUE |  | 226000_at | TRUE |
| 226017_at | TRUE |  | 226017_at | TRUE |
| 226019_at | TRUE |  | 226019_at | TRUE |
| 226020_s_at | TRUE |  | 226020_s_at | TRUE |
| 226066_at | TRUE |  | 226066_at | TRUE |
| 226068_at | TRUE |  | 226068_at | TRUE |
| 226093_at | TRUE |  | 226093_at | TRUE |
| 226113_at | TRUE |  | 226113_at | TRUE |
| 226114_at | TRUE |  | 226114_at | TRUE |
| 226116_at | TRUE |  | 226116_at | TRUE |
| 226133_s_at | TRUE |  | 226133_s_at | TRUE |
| 226170_at | TRUE |  | 226170_at | TRUE |
| 226191_at | TRUE |  | 226191_at | TRUE |
| 226192_at | TRUE |  | 226192_at | TRUE |
| 226197_at | TRUE |  | 226197_at | TRUE |
| 226198_at | TRUE |  | 226198_at | TRUE |
| 226217_at | TRUE |  | 226217_at | TRUE |
| 226219_at | TRUE |  | 226219_at | TRUE |
| 226261_at | TRUE |  | 226261_at | TRUE |
| 226269_at | TRUE |  | 226269_at | TRUE |
| 226271_at | TRUE |  | 226271_at | TRUE |
| 226303_at | TRUE |  | 226303_at | TRUE |
| 226333_at | TRUE |  | 226333_at | TRUE |
| 226350_at | TRUE |  | 226350_at | TRUE |
| 226373_at | TRUE |  | 226373_at | TRUE |
| 226375_at | TRUE |  | 226375_at | TRUE |
| 226377_at | TRUE |  | 226377_at | TRUE |
| 226390_at | TRUE |  | 226390_at | TRUE |
| 226430_at | TRUE |  | 226430_at | TRUE |
| 226459_at | TRUE |  | 226459_at | TRUE |
| 226474_at | TRUE |  | 226474_at | TRUE |
| 226494_at | TRUE |  | 226494_at | TRUE |
| 226498_at | TRUE |  | 226498_at | TRUE |
| 226532_at | TRUE |  | 226532_at | TRUE |
| 226582_at | TRUE |  | 226582_at | TRUE |
| 226592_at | TRUE |  | 226592_at | TRUE |
| 226599_at | TRUE |  | 226599_at | TRUE |
| 226601_at | TRUE |  | 226601_at | TRUE |
| 226653_at | TRUE |  | 226653_at | TRUE |
| 226659_at | TRUE |  | 226659_at | TRUE |
| 226697_at | TRUE |  | 226697_at | TRUE |
| 226723_at | TRUE |  | 226723_at | TRUE |
| 226725_at | TRUE |  | 226725_at | TRUE |
| 226806_s_at | TRUE |  | 226806_s_at | TRUE |
| 226811_at | TRUE |  | 226811_at | TRUE |
| 226818_at | TRUE |  | 226818_at | TRUE |
| 226820_at | TRUE |  | 226820_at | TRUE |
| 226823_at | TRUE |  | 226823_at | TRUE |
| 226841_at | TRUE |  | 226841_at | TRUE |
| 226865_at | TRUE |  | 226865_at | TRUE |
| 226878_at | TRUE |  | 226878_at | TRUE |
| 226884_at | TRUE |  | 226884_at | TRUE |
| 226906_s_at | TRUE |  | 226906_s_at | TRUE |
| 226907_at | TRUE |  | 226907_at | TRUE |
| 226909_at | TRUE |  | 226909_at | TRUE |
| 226964_at | TRUE |  | 226964_at | TRUE |
| 226968_at | TRUE |  | 226968_at | TRUE |
| 226976_at | TRUE |  | 226976_at | TRUE |
| 226989_at | TRUE |  | 226989_at | TRUE |
| 226991_at | TRUE |  | 226991_at | TRUE |
| 227019_at | TRUE |  | 227019_at | TRUE |
| 227052_at | TRUE |  | 227052_at | TRUE |
| 227081_at | TRUE |  | 227081_at | TRUE |
| 227091_at | TRUE |  | 227091_at | TRUE |
| 227095_at | TRUE |  | 227095_at | TRUE |
| 227096_at | TRUE |  | 227096_at | TRUE |
| 227124_at | TRUE |  | 227124_at | TRUE |
| 227143_s_at | TRUE |  | 227143_s_at | TRUE |
| 227150_at | TRUE |  | 227150_at | TRUE |
| 227168_at | TRUE |  | 227168_at | TRUE |
| 227182_at | TRUE |  | 227182_at | TRUE |
| 227184_at | TRUE |  | 227184_at | TRUE |
| 227188_at | TRUE |  | 227188_at | TRUE |
| 227189_at | TRUE |  | 227189_at | TRUE |
| 227206_at | TRUE |  | 227206_at | TRUE |
| 227253_at | TRUE |  | 227253_at | TRUE |
| 227266_s_at | TRUE |  | 227266_s_at | TRUE |
| 227276_at | TRUE |  | 227276_at | TRUE |
| 227300_at | TRUE |  | 227300_at | TRUE |
| 227313_at | TRUE |  | 227313_at | TRUE |
| 227339_at | TRUE |  | 227339_at | TRUE |
| 227340_s_at | TRUE |  | 227340_s_at | TRUE |
| 227344_at | TRUE |  | 227344_at | TRUE |
| 227346_at | TRUE |  | 227346_at | TRUE |
| 227353_at | TRUE |  | 227353_at | TRUE |
| 227365_at | TRUE |  | 227365_at | TRUE |
| 227369_at | TRUE |  | 227369_at | TRUE |
| 227453_at | TRUE |  | 227453_at | TRUE |
| 227514_at | TRUE |  | 227514_at | TRUE |
| 227516_at | TRUE |  | 227516_at | TRUE |
| 227584_at | TRUE |  | 227584_at | TRUE |
| 227614_at | TRUE |  | 227614_at | TRUE |
| 227644_at | TRUE |  | 227644_at | TRUE |
| 227645_at | TRUE |  | 227645_at | TRUE |
| 227647_at | TRUE |  | 227647_at | TRUE |
| 227700_x_at | TRUE |  | 227700_x_at | TRUE |
| 227726_at | TRUE |  | 227726_at | TRUE |
| 227748_at | TRUE |  | 227748_at | TRUE |
| 227769_at | TRUE |  | 227769_at | TRUE |
| 227792_at | TRUE |  | 227792_at | TRUE |
| 227847_at | TRUE |  | 227847_at | TRUE |
| 227889_at | TRUE |  | 227889_at | TRUE |
| 227898_s_at | TRUE |  | 227898_s_at | TRUE |
| 227924_at | TRUE |  | 227924_at | TRUE |
| 227954_at | TRUE |  | 227954_at | TRUE |
| 227965_at | TRUE |  | 227965_at | TRUE |
| 227975_at | TRUE |  | 227975_at | TRUE |
| 227983_at | TRUE |  | 227983_at | TRUE |
| 227995_at | TRUE |  | 227995_at | TRUE |
| 228028_at | TRUE |  | 228028_at | TRUE |
| 228056_s_at | TRUE |  | 228056_s_at | TRUE |
| 228062_at | TRUE |  | 228062_at | TRUE |
| 228064_at | TRUE |  | 228064_at | TRUE |
| 228083_at | TRUE |  | 228083_at | TRUE |
| 228100_at | TRUE |  | 228100_at | TRUE |
| 228127_at | TRUE |  | 228127_at | TRUE |
| 228131_at | TRUE |  | 228131_at | TRUE |
| 228143_at | TRUE |  | 228143_at | TRUE |
| 228174_at | TRUE |  | 228174_at | TRUE |
| 228176_at | TRUE |  | 228176_at | TRUE |
| 228221_at | TRUE |  | 228221_at | TRUE |
| 228234_at | TRUE |  | 228234_at | TRUE |
| 228261_at | TRUE |  | 228261_at | TRUE |
| 228269_x_at | TRUE |  | 228269_x_at | TRUE |
| 228305_at | TRUE |  | 228305_at | TRUE |
| 228349_at | TRUE |  | 228349_at | TRUE |
| 228369_at | TRUE |  | 228369_at | TRUE |
| 228375_at | TRUE |  | 228375_at | TRUE |
| 228388_at | TRUE |  | 228388_at | TRUE |
| 228410_at | TRUE |  | 228410_at | TRUE |
| 228422_at | TRUE |  | 228422_at | TRUE |
| 228433_at | TRUE |  | 228433_at | TRUE |
| 228442_at | TRUE |  | 228442_at | TRUE |
| 228480_at | TRUE |  | 228480_at | TRUE |
| 228509_at | TRUE |  | 228509_at | TRUE |
| 228521_s_at | TRUE |  | 228521_s_at | TRUE |
| 228532_at | TRUE |  | 228532_at | TRUE |
| 228538_at | TRUE |  | 228538_at | TRUE |
| 228547_at | TRUE |  | 228547_at | TRUE |
| 228548_at | TRUE |  | 228548_at | TRUE |
| 228566_at | TRUE |  | 228566_at | TRUE |
| 228604_at | TRUE |  | 228604_at | TRUE |
| 228605_at | TRUE |  | 228605_at | TRUE |
| 228641_at | TRUE |  | 228641_at | TRUE |
| 228658_at | TRUE |  | 228658_at | TRUE |
| 228680_at | TRUE |  | 228680_at | TRUE |
| 228733_at | TRUE |  | 228733_at | TRUE |
| 228741_s_at | TRUE |  | 228741_s_at | TRUE |
| 228761_at | TRUE |  | 228761_at | TRUE |
| 228804_at | TRUE |  | 228804_at | TRUE |
| 228821_at | TRUE |  | 228821_at | TRUE |
| 228869_at | TRUE |  | 228869_at | TRUE |
| 228882_at | TRUE |  | 228882_at | TRUE |
| 228890_at | TRUE |  | 228890_at | TRUE |
| 228920_at | TRUE |  | 228920_at | TRUE |
| 228949_at | TRUE |  | 228949_at | TRUE |
| 228950_s_at | TRUE |  | 228950_s_at | TRUE |
| 229011_at | TRUE |  | 229011_at | TRUE |
| 229134_at | TRUE |  | 229134_at | TRUE |
| 229146_at | TRUE |  | 229146_at | TRUE |
| 229204_at | TRUE |  | 229204_at | TRUE |
| 229221_at | TRUE |  | 229221_at | TRUE |
| 229265_at | TRUE |  | 229265_at | TRUE |
| 229271_x_at | TRUE |  | 229271_x_at | TRUE |
| 229313_at | TRUE |  | 229313_at | TRUE |
| 229335_at | TRUE |  | 229335_at | TRUE |
| 229370_at | TRUE |  | 229370_at | TRUE |
| 229425_at | TRUE |  | 229425_at | TRUE |
| 229428_at | TRUE |  | 229428_at | TRUE |
| 229435_at | TRUE |  | 229435_at | TRUE |
| 229498_at | TRUE |  | 229498_at | TRUE |
| 229507_at | TRUE |  | 229507_at | TRUE |
| 229550_at | TRUE |  | 229550_at | TRUE |
| 229560_at | TRUE |  | 229560_at | TRUE |
| 229597_s_at | TRUE |  | 229597_s_at | TRUE |
| 229651_at | TRUE |  | 229651_at | TRUE |
| 229672_at | TRUE |  | 229672_at | TRUE |
| 229715_at | TRUE |  | 229715_at | TRUE |
| 229725_at | TRUE |  | 229725_at | TRUE |
| 229743_at | TRUE |  | 229743_at | TRUE |
| 229812_at | TRUE |  | 229812_at | TRUE |
| 229816_at | TRUE |  | 229816_at | TRUE |
| 229818_at | TRUE |  | 229818_at | TRUE |
| 229823_at | TRUE |  | 229823_at | TRUE |
| 229824_at | TRUE |  | 229824_at | TRUE |
| 229874_x_at | TRUE |  | 229874_x_at | TRUE |
| 229875_at | TRUE |  | 229875_at | TRUE |
| 229937_x_at | TRUE |  | 229937_x_at | TRUE |
| 229953_x_at | TRUE |  | 229953_x_at | TRUE |
| 229968_at | TRUE |  | 229968_at | TRUE |
| 229991_s_at | TRUE |  | 229991_s_at | TRUE |
| 229997_at | TRUE |  | 229997_at | TRUE |
| 230022_at | TRUE |  | 230022_at | TRUE |
| 230069_at | TRUE |  | 230069_at | TRUE |
| 230112_at | TRUE |  | 230112_at | TRUE |
| 230137_at | TRUE |  | 230137_at | TRUE |
| 230194_at | TRUE |  | 230194_at | TRUE |
| 230230_at | TRUE |  | 230230_at | TRUE |
| 230252_at | TRUE |  | 230252_at | TRUE |
| 230258_at | TRUE |  | 230258_at | TRUE |
| 230259_at | TRUE |  | 230259_at | TRUE |
| 230261_at | TRUE |  | 230261_at | TRUE |
| 230272_at | TRUE |  | 230272_at | TRUE |
| 230275_at | TRUE |  | 230275_at | TRUE |
| 230280_at | TRUE |  | 230280_at | TRUE |
| 230296_at | TRUE |  | 230296_at | TRUE |
| 230369_at | TRUE |  | 230369_at | TRUE |
| 230372_at | TRUE |  | 230372_at | TRUE |
| 230391_at | TRUE |  | 230391_at | TRUE |
| 230417_at | TRUE |  | 230417_at | TRUE |
| 230418_s_at | TRUE |  | 230418_s_at | TRUE |
| 230422_at | TRUE |  | 230422_at | TRUE |
| 230433_at | TRUE |  | 230433_at | TRUE |
| 230445_at | TRUE |  | 230445_at | TRUE |
| 230475_at | TRUE |  | 230475_at | TRUE |
| 230496_at | TRUE |  | 230496_at | TRUE |
| 230550_at | TRUE |  | 230550_at | TRUE |
| 230551_at | TRUE |  | 230551_at | TRUE |
| 230561_s_at | TRUE |  | 230561_s_at | TRUE |
| 230635_at | TRUE |  | 230635_at | TRUE |
| 230645_at | TRUE |  | 230645_at | TRUE |
| 230657_at | TRUE |  | 230657_at | TRUE |
| 230821_at | TRUE |  | 230821_at | TRUE |
| 230826_at | TRUE |  | 230826_at | TRUE |
| 230866_at | TRUE |  | 230866_at | TRUE |
| 230869_at | TRUE |  | 230869_at | TRUE |
| 230917_at | TRUE |  | 230917_at | TRUE |
| 230925_at | TRUE |  | 230925_at | TRUE |
| 231018_at | TRUE |  | 231018_at | TRUE |
| 231130_at | TRUE |  | 231130_at | TRUE |
| 231185_at | TRUE |  | 231185_at | TRUE |
| 231260_at | TRUE |  | 231260_at | TRUE |
| 231364_at | TRUE |  | 231364_at | TRUE |
| 231526_at | TRUE |  | 231526_at | TRUE |
| 231577_s_at | TRUE |  | 231577_s_at | TRUE |
| 231608_at | TRUE |  | 231608_at | TRUE |
| 231650_s_at | TRUE |  | 231650_s_at | TRUE |
| 231714_s_at | TRUE |  | 231714_s_at | TRUE |
| 231740_at | TRUE |  | 231740_at | TRUE |
| 231741_at | TRUE |  | 231741_at | TRUE |
| 231747_at | TRUE |  | 231747_at | TRUE |
| 231765_at | TRUE |  | 231765_at | TRUE |
| 231788_at | TRUE |  | 231788_at | TRUE |
| 231804_at | TRUE |  | 231804_at | TRUE |
| 231836_at | TRUE |  | 231836_at | TRUE |
| 231887_s_at | TRUE |  | 231887_s_at | TRUE |
| 231890_at | TRUE |  | 231890_at | TRUE |
| 231935_at | TRUE |  | 231935_at | TRUE |
| 231972_at | TRUE |  | 231972_at | TRUE |
| 231990_at | TRUE |  | 231990_at | TRUE |
| 232015_at | TRUE |  | 232015_at | TRUE |
| 232032_x_at | TRUE |  | 232032_x_at | TRUE |
| 232054_at | TRUE |  | 232054_at | TRUE |
| 232081_at | TRUE |  | 232081_at | TRUE |
| 232231_at | TRUE |  | 232231_at | TRUE |
| 232244_at | TRUE |  | 232244_at | TRUE |
| 232317_at | TRUE |  | 232317_at | TRUE |
| 232350_x_at | TRUE |  | 232350_x_at | TRUE |
| 232543_x_at | TRUE |  | 232543_x_at | TRUE |
| 232589_at | TRUE |  | 232589_at | TRUE |
| 232617_at | TRUE |  | 232617_at | TRUE |
| 232693_s_at | TRUE |  | 232693_s_at | TRUE |
| 232780_s_at | TRUE |  | 232780_s_at | TRUE |
| 232843_s_at | TRUE |  | 232843_s_at | TRUE |
| 232904_at | TRUE |  | 232904_at | TRUE |
| 233064_at | TRUE |  | 233064_at | TRUE |
| 233220_at | TRUE |  | 233220_at | TRUE |
| 233295_at | TRUE |  | 233295_at | TRUE |
| 233310_at | TRUE |  | 233310_at | TRUE |
| 233337_s_at | TRUE |  | 233337_s_at | TRUE |
| 233357_at | TRUE |  | 233357_at | TRUE |
| 233406_at | TRUE |  | 233406_at | TRUE |
| 233433_at | TRUE |  | 233433_at | TRUE |
| 233510_s_at | TRUE |  | 233510_s_at | TRUE |
| 233587_s_at | TRUE |  | 233587_s_at | TRUE |
| 233602_at | TRUE |  | 233602_at | TRUE |
| 233613_x_at | TRUE |  | 233613_x_at | TRUE |
| 233625_x_at | TRUE |  | 233625_x_at | TRUE |
| 233688_at | TRUE |  | 233688_at | TRUE |
| 233917_s_at | TRUE |  | 233917_s_at | TRUE |
| 234170_at | TRUE |  | 234170_at | TRUE |
| 234340_at | TRUE |  | 234340_at | TRUE |
| 234672_s_at | TRUE |  | 234672_s_at | TRUE |
| 234710_s_at | TRUE |  | 234710_s_at | TRUE |
| 234871_at | TRUE |  | 234871_at | TRUE |
| 234880_x_at | TRUE |  | 234880_x_at | TRUE |
| 234974_at | TRUE |  | 234974_at | TRUE |
| 234976_x_at | TRUE |  | 234976_x_at | TRUE |
| 235031_at | TRUE |  | 235031_at | TRUE |
| 235224_s_at | TRUE |  | 235224_s_at | TRUE |
| 235230_at | TRUE |  | 235230_at | TRUE |
| 235256_s_at | TRUE |  | 235256_s_at | TRUE |
| 235306_at | TRUE |  | 235306_at | TRUE |
| 235319_at | TRUE |  | 235319_at | TRUE |
| 235343_at | TRUE |  | 235343_at | TRUE |
| 235359_at | TRUE |  | 235359_at | TRUE |
| 235384_at | TRUE |  | 235384_at | TRUE |
| 235388_at | TRUE |  | 235388_at | TRUE |
| 235409_at | TRUE |  | 235409_at | TRUE |
| 235412_at | TRUE |  | 235412_at | TRUE |
| 235414_at | TRUE |  | 235414_at | TRUE |
| 235417_at | TRUE |  | 235417_at | TRUE |
| 235452_at | TRUE |  | 235452_at | TRUE |
| 235458_at | TRUE |  | 235458_at | TRUE |
| 235459_at | TRUE |  | 235459_at | TRUE |
| 235475_at | TRUE |  | 235475_at | TRUE |
| 235492_at | TRUE |  | 235492_at | TRUE |
| 235506_at | TRUE |  | 235506_at | TRUE |
| 235559_at | TRUE |  | 235559_at | TRUE |
| 235573_at | TRUE |  | 235573_at | TRUE |
| 235664_at | TRUE |  | 235664_at | TRUE |
| 235702_at | TRUE |  | 235702_at | TRUE |
| 235735_at | TRUE |  | 235735_at | TRUE |
| 235742_at | TRUE |  | 235742_at | TRUE |
| 235802_at | TRUE |  | 235802_at | TRUE |
| 235856_at | TRUE |  | 235856_at | TRUE |
| 235880_at | TRUE |  | 235880_at | TRUE |
| 235949_at | TRUE |  | 235949_at | TRUE |
| 235957_at | TRUE |  | 235957_at | TRUE |
| 235960_at | TRUE |  | 235960_at | TRUE |
| 235961_at | TRUE |  | 235961_at | TRUE |
| 236079_at | TRUE |  | 236079_at | TRUE |
| 236108_at | TRUE |  | 236108_at | TRUE |
| 236114_at | TRUE |  | 236114_at | TRUE |
| 236204_at | TRUE |  | 236204_at | TRUE |
| 236308_at | TRUE |  | 236308_at | TRUE |
| 236313_at | TRUE |  | 236313_at | TRUE |
| 236330_at | TRUE |  | 236330_at | TRUE |
| 236333_at | TRUE |  | 236333_at | TRUE |
| 236343_at | TRUE |  | 236343_at | TRUE |
| 236401_at | TRUE |  | 236401_at | TRUE |
| 236592_at | TRUE |  | 236592_at | TRUE |
| 236638_at | TRUE |  | 236638_at | TRUE |
| 236646_at | TRUE |  | 236646_at | TRUE |
| 236739_at | TRUE |  | 236739_at | TRUE |
| 237223_at | TRUE |  | 237223_at | TRUE |
| 237322_at | TRUE |  | 237322_at | TRUE |
| 237442_at | TRUE |  | 237442_at | TRUE |
| 237450_at | TRUE |  | 237450_at | TRUE |
| 237452_at | TRUE |  | 237452_at | TRUE |
| 237571_at | TRUE |  | 237571_at | TRUE |
| 237623_at | TRUE |  | 237623_at | TRUE |
| 237823_at | TRUE |  | 237823_at | TRUE |
| 237973_at | TRUE |  | 237973_at | TRUE |
| 238013_at | TRUE |  | 238013_at | TRUE |
| 238063_at | TRUE |  | 238063_at | TRUE |
| 238126_at | TRUE |  | 238126_at | TRUE |
| 238135_at | TRUE |  | 238135_at | TRUE |
| 238151_at | TRUE |  | 238151_at | TRUE |
| 238178_at | TRUE |  | 238178_at | TRUE |
| 238194_at | TRUE |  | 238194_at | TRUE |
| 238206_at | TRUE |  | 238206_at | TRUE |
| 238365_s_at | TRUE |  | 238365_s_at | TRUE |
| 238427_at | TRUE |  | 238427_at | TRUE |
| 238436_s_at | TRUE |  | 238436_s_at | TRUE |
| 238444_at | TRUE |  | 238444_at | TRUE |
| 238490_at | TRUE |  | 238490_at | TRUE |
| 238512_at | TRUE |  | 238512_at | TRUE |
| 238591_at | TRUE |  | 238591_at | TRUE |
| 238623_at | TRUE |  | 238623_at | TRUE |
| 238661_at | TRUE |  | 238661_at | TRUE |
| 238668_at | TRUE |  | 238668_at | TRUE |
| 238669_at | TRUE |  | 238669_at | TRUE |
| 238789_at | TRUE |  | 238789_at | TRUE |
| 238975_at | TRUE |  | 238975_at | TRUE |
| 239021_at | TRUE |  | 239021_at | TRUE |
| 239024_at | TRUE |  | 239024_at | TRUE |
| 239027_at | TRUE |  | 239027_at | TRUE |
| 239031_at | TRUE |  | 239031_at | TRUE |
| 239168_at | TRUE |  | 239168_at | TRUE |
| 239229_at | TRUE |  | 239229_at | TRUE |
| 239235_at | TRUE |  | 239235_at | TRUE |
| 239250_at | TRUE |  | 239250_at | TRUE |
| 239275_at | TRUE |  | 239275_at | TRUE |
| 239283_at | TRUE |  | 239283_at | TRUE |
| 239293_at | TRUE |  | 239293_at | TRUE |
| 239294_at | TRUE |  | 239294_at | TRUE |
| 239358_at | TRUE |  | 239358_at | TRUE |
| 239407_at | TRUE |  | 239407_at | TRUE |
| 239448_at | TRUE |  | 239448_at | TRUE |
| 239525_at | TRUE |  | 239525_at | TRUE |
| 239606_at | TRUE |  | 239606_at | TRUE |
| 239678_at | TRUE |  | 239678_at | TRUE |
| 239682_at | TRUE |  | 239682_at | TRUE |
| 239729_at | TRUE |  | 239729_at | TRUE |
| 239903_at | TRUE |  | 239903_at | TRUE |
| 239984_at | TRUE |  | 239984_at | TRUE |
| 239992_at | TRUE |  | 239992_at | TRUE |
| 240011_at | TRUE |  | 240011_at | TRUE |
| 240037_at | TRUE |  | 240037_at | TRUE |
| 240041_at | TRUE |  | 240041_at | TRUE |
| 240064_at | TRUE |  | 240064_at | TRUE |
| 240065_at | TRUE |  | 240065_at | TRUE |
| 240574_at | TRUE |  | 240574_at | TRUE |
| 240673_at | TRUE |  | 240673_at | TRUE |
| 240703_s_at | TRUE |  | 240703_s_at | TRUE |
| 240841_at | TRUE |  | 240841_at | TRUE |
| 240861_at | TRUE |  | 240861_at | TRUE |
| 240974_at | TRUE |  | 240974_at | TRUE |
| 241068_at | TRUE |  | 241068_at | TRUE |
| 241365_at | TRUE |  | 241365_at | TRUE |
| 241389_at | TRUE |  | 241389_at | TRUE |
| 241393_at | TRUE |  | 241393_at | TRUE |
| 241398_at | TRUE |  | 241398_at | TRUE |
| 241434_at | TRUE |  | 241434_at | TRUE |
| 241470_x_at | TRUE |  | 241470_x_at | TRUE |
| 241583_x_at | TRUE |  | 241583_x_at | TRUE |
| 241612_at | TRUE |  | 241612_at | TRUE |
| 241730_at | TRUE |  | 241730_at | TRUE |
| 241742_at | TRUE |  | 241742_at | TRUE |
| 241763_s_at | TRUE |  | 241763_s_at | TRUE |
| 241808_at | TRUE |  | 241808_at | TRUE |
| 241812_at | TRUE |  | 241812_at | TRUE |
| 241891_at | TRUE |  | 241891_at | TRUE |
| 241947_at | TRUE |  | 241947_at | TRUE |
| 241986_at | TRUE |  | 241986_at | TRUE |
| 241991_at | TRUE |  | 241991_at | TRUE |
| 241999_at | TRUE |  | 241999_at | TRUE |
| 242082_at | TRUE |  | 242082_at | TRUE |
| 242091_at | TRUE |  | 242091_at | TRUE |
| 242123_at | TRUE |  | 242123_at | TRUE |
| 242193_at | TRUE |  | 242193_at | TRUE |
| 242263_at | TRUE |  | 242263_at | TRUE |
| 242285_at | TRUE |  | 242285_at | TRUE |
| 242451_x_at | TRUE |  | 242451_x_at | TRUE |
| 242487_at | TRUE |  | 242487_at | TRUE |
| 242499_at | TRUE |  | 242499_at | TRUE |
| 242523_at | TRUE |  | 242523_at | TRUE |
| 242600_at | TRUE |  | 242600_at | TRUE |
| 242618_at | TRUE |  | 242618_at | TRUE |
| 242640_at | TRUE |  | 242640_at | TRUE |
| 242761_s_at | TRUE |  | 242761_s_at | TRUE |
| 242794_at | TRUE |  | 242794_at | TRUE |
| 242870_at | TRUE |  | 242870_at | TRUE |
| 242907_at | TRUE |  | 242907_at | TRUE |
| 242931_at | TRUE |  | 242931_at | TRUE |
| 242953_at | TRUE |  | 242953_at | TRUE |
| 243027_at | TRUE |  | 243027_at | TRUE |
| 243061_at | TRUE |  | 243061_at | TRUE |
| 243099_at | TRUE |  | 243099_at | TRUE |
| 243430_at | TRUE |  | 243430_at | TRUE |
| 243504_at | TRUE |  | 243504_at | TRUE |
| 243543_at | TRUE |  | 243543_at | TRUE |
| 243549_at | TRUE |  | 243549_at | TRUE |
| 243658_at | TRUE |  | 243658_at | TRUE |
| 243665_s_at | TRUE |  | 243665_s_at | TRUE |
| 243666_at | TRUE |  | 243666_at | TRUE |
| 243681_at | TRUE |  | 243681_at | TRUE |
| 243747_at | TRUE |  | 243747_at | TRUE |
| 243813_at | TRUE |  | 243813_at | TRUE |
| 243836_at | TRUE |  | 243836_at | TRUE |
| 243871_at | TRUE |  | 243871_at | TRUE |
| 243872_at | TRUE |  | 243872_at | TRUE |
| 243929_at | TRUE |  | 243929_at | TRUE |
| 243967_at | TRUE |  | 243967_at | TRUE |
| 244029_at | TRUE |  | 244029_at | TRUE |
| 244050_at | TRUE |  | 244050_at | TRUE |
| 244061_at | TRUE |  | 244061_at | TRUE |
| 244071_at | TRUE |  | 244071_at | TRUE |
| 244099_at | TRUE |  | 244099_at | TRUE |
| 244184_at | TRUE |  | 244184_at | TRUE |
| 244208_at | TRUE |  | 244208_at | TRUE |
| 244227_at | TRUE |  | 244227_at | TRUE |
| 244251_at | TRUE |  | 244251_at | TRUE |
| 244352_at | TRUE |  | 244352_at | TRUE |
| 244398_x_at | TRUE |  | 244398_x_at | TRUE |
| 244413_at | TRUE |  | 244413_at | TRUE |
| 244435_at | TRUE |  | 244435_at | TRUE |
| 244609_at | TRUE |  | 244609_at | TRUE |
| 244650_at | TRUE |  | 244650_at | TRUE |
| 244662_at | TRUE |  | 244662_at | TRUE |
| 244664_at | TRUE |  | 244664_at | TRUE |
| 244795_at | TRUE |  | 244795_at | TRUE |
| 31845_at | TRUE |  | 31845_at | TRUE |
| 33197_at | TRUE |  | 33197_at | TRUE |
| 33760_at | TRUE |  | 33760_at | TRUE |
| 35666_at | TRUE |  | 35666_at | TRUE |
| 36030_at | TRUE |  | 36030_at | TRUE |
| 37012_at | TRUE |  | 37012_at | TRUE |
| 37549_g_at | TRUE |  | 37549_g_at | TRUE |
| 37892_at | TRUE |  | 37892_at | TRUE |
| 37996_s_at | TRUE |  | 37996_s_at | TRUE |
| 38149_at | TRUE |  | 38149_at | TRUE |
| 38269_at | TRUE |  | 38269_at | TRUE |
| 39891_at | TRUE |  | 39891_at | TRUE |
| 40020_at | TRUE |  | 40020_at | TRUE |
| 44783_s_at | TRUE |  | 44783_s_at | TRUE |
| 46167_at | TRUE |  | 46167_at | TRUE |
| 48659_at | TRUE |  | 48659_at | TRUE |
| 52078_at | TRUE |  | 52078_at | TRUE |
| 52164_at | TRUE |  | 52164_at | TRUE |
| 58367_s_at | TRUE |  | 58367_s_at | TRUE |
| 81737_at | TRUE |  | 81737_at | TRUE |
| 90610_at | TRUE |  | 90610_at | TRUE |
| 235355_at | FALSE |  |  |  |

Table S6

| **Gene Calling by LIMMA (α=0.05)** | | | | |
| --- | --- | --- | --- | --- |
| **Parent** | |  | **Transformed** | |
| **Affymetrix ID** | **In Both?** |  | **Affymetrix ID** | **In Both?** |
| 1294_at | TRUE |  | 1294_at | TRUE |
| 1552256_a_at | TRUE |  | 1552256_a_at | TRUE |
| 1552301_a_at | TRUE |  | 1552301_a_at | TRUE |
| 1552302_at | TRUE |  | 1552302_at | TRUE |
| 1552303_a_at | TRUE |  | 1552303_a_at | TRUE |
| 1552315_at | TRUE |  | 1552315_at | TRUE |
| 1552316_a_at | TRUE |  | 1552316_a_at | TRUE |
| 1552318_at | TRUE |  | 1552318_at | TRUE |
| 1552344_s_at | TRUE |  | 1552344_s_at | TRUE |
| 1552365_at | TRUE |  | 1552365_at | TRUE |
| 1552367_a_at | TRUE |  | 1552367_a_at | TRUE |
| 1552381_at | TRUE |  | 1552381_at | TRUE |
| 1552584_at | TRUE |  | 1552584_at | TRUE |
| 1552646_at | TRUE |  | 1552646_at | TRUE |
| 1552695_a_at | TRUE |  | 1552695_a_at | TRUE |
| 1552701_a_at | TRUE |  | 1552701_a_at | TRUE |
| 1552703_s_at | TRUE |  | 1552703_s_at | TRUE |
| 1552714_at | TRUE |  | 1552714_at | TRUE |
| 1552715_a_at | TRUE |  | 1552715_a_at | TRUE |
| 1552734_at | TRUE |  | 1552734_at | TRUE |
| 1552807_a_at | TRUE |  | 1552807_a_at | TRUE |
| 1553009_s_at | TRUE |  | 1553009_s_at | TRUE |
| 1553043_a_at | TRUE |  | 1553043_a_at | TRUE |
| 1553268_at | TRUE |  | 1553268_at | TRUE |
| 1553286_at | TRUE |  | 1553286_at | TRUE |
| 1553297_a_at | TRUE |  | 1553297_a_at | TRUE |
| 1553350_at | TRUE |  | 1553350_at | TRUE |
| 1553410_a_at | TRUE |  | 1553410_a_at | TRUE |
| 1553415_at | TRUE |  | 1553415_at | TRUE |
| 1553422_s_at | TRUE |  | 1553422_s_at | TRUE |
| 1553654_at | TRUE |  | 1553654_at | TRUE |
| 1553732_s_at | TRUE |  | 1553732_s_at | TRUE |
| 1553764_a_at | TRUE |  | 1553764_a_at | TRUE |
| 1553787_at | TRUE |  | 1553787_at | TRUE |
| 1553796_at | TRUE |  | 1553796_at | TRUE |
| 1553797_a_at | TRUE |  | 1553797_a_at | TRUE |
| 1553864_at | TRUE |  | 1553864_at | TRUE |
| 1553883_at | TRUE |  | 1553883_at | TRUE |
| 1553885_x_at | TRUE |  | 1553885_x_at | TRUE |
| 1553954_at | TRUE |  | 1553954_at | TRUE |
| 1553962_s_at | TRUE |  | 1553962_s_at | TRUE |
| 1553993_s_at | TRUE |  | 1553993_s_at | TRUE |
| 1553997_a_at | TRUE |  | 1553997_a_at | TRUE |
| 1554008_at | TRUE |  | 1554008_at | TRUE |
| 1554034_a_at | TRUE |  | 1554034_a_at | TRUE |
| 1554060_s_at | TRUE |  | 1554060_s_at | TRUE |
| 1554099_a_at | TRUE |  | 1554099_a_at | TRUE |
| 1554127_s_at | TRUE |  | 1554127_s_at | TRUE |
| 1554140_at | TRUE |  | 1554140_at | TRUE |
| 1554141_s_at | TRUE |  | 1554141_s_at | TRUE |
| 1554205_s_at | TRUE |  | 1554205_s_at | TRUE |
| 1554240_a_at | TRUE |  | 1554240_a_at | TRUE |
| 1554251_at | TRUE |  | 1554251_at | TRUE |
| 1554285_at | TRUE |  | 1554285_at | TRUE |
| 1554322_a_at | TRUE |  | 1554322_a_at | TRUE |
| 1554328_at | TRUE |  | 1554328_at | TRUE |
| 1554406_a_at | TRUE |  | 1554406_a_at | TRUE |
| 1554448_at | TRUE |  | 1554448_at | TRUE |
| 1554470_s_at | TRUE |  | 1554470_s_at | TRUE |
| 1554500_a_at | TRUE |  | 1554500_a_at | TRUE |
| 1554503_a_at | TRUE |  | 1554503_a_at | TRUE |
| 1554524_a_at | TRUE |  | 1554524_a_at | TRUE |
| 1554526_at | TRUE |  | 1554526_at | TRUE |
| 1554530_at | TRUE |  | 1554530_at | TRUE |
| 1554592_a_at | TRUE |  | 1554592_a_at | TRUE |
| 1554607_at | TRUE |  | 1554607_at | TRUE |
| 1554633_a_at | TRUE |  | 1554633_a_at | TRUE |
| 1554637_a_at | TRUE |  | 1554637_a_at | TRUE |
| 1554663_a_at | TRUE |  | 1554663_a_at | TRUE |
| 1554675_a_at | TRUE |  | 1554675_a_at | TRUE |
| 1554717_a_at | TRUE |  | 1554717_a_at | TRUE |
| 1554791_a_at | TRUE |  | 1554791_a_at | TRUE |
| 1554873_at | TRUE |  | 1554873_at | TRUE |
| 1554883_a_at | TRUE |  | 1554883_a_at | TRUE |
| 1554899_s_at | TRUE |  | 1554899_s_at | TRUE |
| 1555024_at | TRUE |  | 1555024_at | TRUE |
| 1555097_a_at | TRUE |  | 1555097_a_at | TRUE |
| 1555123_at | TRUE |  | 1555123_at | TRUE |
| 1555146_at | TRUE |  | 1555146_at | TRUE |
| 1555202_a_at | TRUE |  | 1555202_a_at | TRUE |
| 1555230_a_at | TRUE |  | 1555230_a_at | TRUE |
| 1555240_s_at | TRUE |  | 1555240_s_at | TRUE |
| 1555266_a_at | TRUE |  | 1555266_a_at | TRUE |
| 1555279_at | TRUE |  | 1555279_at | TRUE |
| 1555281_x_at | TRUE |  | 1555281_x_at | TRUE |
| 1555313_a_at | TRUE |  | 1555313_a_at | TRUE |
| 1555316_a_at | TRUE |  | 1555316_a_at | TRUE |
| 1555330_at | TRUE |  | 1555330_at | TRUE |
| 1555349_a_at | TRUE |  | 1555349_a_at | TRUE |
| 1555486_a_at | TRUE |  | 1555486_a_at | TRUE |
| 1555514_a_at | TRUE |  | 1555514_a_at | TRUE |
| 1555557_a_at | TRUE |  | 1555557_a_at | TRUE |
| 1555562_a_at | TRUE |  | 1555562_a_at | TRUE |
| 1555575_a_at | TRUE |  | 1555575_a_at | TRUE |
| 1555600_s_at | TRUE |  | 1555600_s_at | TRUE |
| 1555638_a_at | TRUE |  | 1555638_a_at | TRUE |
| 1555694_a_at | TRUE |  | 1555694_a_at | TRUE |
| 1555735_a_at | TRUE |  | 1555735_a_at | TRUE |
| 1555736_a_at | TRUE |  | 1555736_a_at | TRUE |
| 1555756_a_at | TRUE |  | 1555756_a_at | TRUE |
| 1555765_a_at | TRUE |  | 1555765_a_at | TRUE |
| 1555781_at | TRUE |  | 1555781_at | TRUE |
| 1555783_x_at | TRUE |  | 1555783_x_at | TRUE |
| 1555801_s_at | TRUE |  | 1555801_s_at | TRUE |
| 1555812_a_at | TRUE |  | 1555812_a_at | TRUE |
| 1555830_s_at | TRUE |  | 1555830_s_at | TRUE |
| 1555832_s_at | TRUE |  | 1555832_s_at | TRUE |
| 1555882_at | TRUE |  | 1555882_at | TRUE |
| 1555883_s_at | TRUE |  | 1555883_s_at | TRUE |
| 1555900_at | TRUE |  | 1555900_at | TRUE |
| 1555935_s_at | TRUE |  | 1555935_s_at | TRUE |
| 1555958_at | TRUE |  | 1555958_at | TRUE |
| 1556017_at | TRUE |  | 1556017_at | TRUE |
| 1556029_s_at | TRUE |  | 1556029_s_at | TRUE |
| 1556034_s_at | TRUE |  | 1556034_s_at | TRUE |
| 1556046_a_at | TRUE |  | 1556046_a_at | TRUE |
| 1556147_at | TRUE |  | 1556147_at | TRUE |
| 1556148_s_at | TRUE |  | 1556148_s_at | TRUE |
| 1556186_s_at | TRUE |  | 1556186_s_at | TRUE |
| 1556236_at | TRUE |  | 1556236_at | TRUE |
| 1556331_a_at | TRUE |  | 1556331_a_at | TRUE |
| 1556332_at | TRUE |  | 1556332_at | TRUE |
| 1556351_at | TRUE |  | 1556351_at | TRUE |
| 1556444_a_at | TRUE |  | 1556444_a_at | TRUE |
| 1556457_s_at | TRUE |  | 1556457_s_at | TRUE |
| 1556471_at | TRUE |  | 1556471_at | TRUE |
| 1556583_a_at | TRUE |  | 1556583_a_at | TRUE |
| 1556629_a_at | TRUE |  | 1556629_a_at | TRUE |
| 1556641_at | TRUE |  | 1556641_at | TRUE |
| 1556730_at | TRUE |  | 1556730_at | TRUE |
| 1556760_a_at | TRUE |  | 1556760_a_at | TRUE |
| 1556763_at | TRUE |  | 1556763_at | TRUE |
| 1556940_at | TRUE |  | 1556940_at | TRUE |
| 1556941_a_at | TRUE |  | 1556941_a_at | TRUE |
| 1557073_s_at | TRUE |  | 1557073_s_at | TRUE |
| 1557098_s_at | TRUE |  | 1557098_s_at | TRUE |
| 1557122_s_at | TRUE |  | 1557122_s_at | TRUE |
| 1557181_s_at | TRUE |  | 1557181_s_at | TRUE |
| 1557267_s_at | TRUE |  | 1557267_s_at | TRUE |
| 1557302_at | TRUE |  | 1557302_at | TRUE |
| 1557326_at | TRUE |  | 1557326_at | TRUE |
| 1557346_a_at | TRUE |  | 1557346_a_at | TRUE |
| 1557352_at | TRUE |  | 1557352_at | TRUE |
| 1557415_s_at | TRUE |  | 1557415_s_at | TRUE |
| 1557422_at | TRUE |  | 1557422_at | TRUE |
| 1557433_at | TRUE |  | 1557433_at | TRUE |
| 1557451_at | TRUE |  | 1557451_at | TRUE |
| 1557475_at | TRUE |  | 1557475_at | TRUE |
| 1557567_a_at | TRUE |  | 1557567_a_at | TRUE |
| 1557637_at | TRUE |  | 1557637_at | TRUE |
| 1557644_at | TRUE |  | 1557644_at | TRUE |
| 1557720_s_at | TRUE |  | 1557720_s_at | TRUE |
| 1557746_at | TRUE |  | 1557746_at | TRUE |
| 1557924_s_at | TRUE |  | 1557924_s_at | TRUE |
| 1558034_s_at | TRUE |  | 1558034_s_at | TRUE |
| 1558041_a_at | TRUE |  | 1558041_a_at | TRUE |
| 1558044_s_at | TRUE |  | 1558044_s_at | TRUE |
| 1558046_x_at | TRUE |  | 1558046_x_at | TRUE |
| 1558122_s_at | TRUE |  | 1558122_s_at | TRUE |
| 1558144_at | TRUE |  | 1558144_at | TRUE |
| 1558236_at | TRUE |  | 1558236_at | TRUE |
| 1558237_x_at | TRUE |  | 1558237_x_at | TRUE |
| 1558463_s_at | TRUE |  | 1558463_s_at | TRUE |
| 1558473_at | TRUE |  | 1558473_at | TRUE |
| 1558672_at | TRUE |  | 1558672_at | TRUE |
| 1558705_at | TRUE |  | 1558705_at | TRUE |
| 1558706_a_at | TRUE |  | 1558706_a_at | TRUE |
| 1558725_at | TRUE |  | 1558725_at | TRUE |
| 1559023_a_at | TRUE |  | 1559023_a_at | TRUE |
| 1559034_at | TRUE |  | 1559034_at | TRUE |
| 1559051_s_at | TRUE |  | 1559051_s_at | TRUE |
| 1559159_at | TRUE |  | 1559159_at | TRUE |
| 1559203_s_at | TRUE |  | 1559203_s_at | TRUE |
| 1559342_a_at | TRUE |  | 1559342_a_at | TRUE |
| 1559351_at | TRUE |  | 1559351_at | TRUE |
| 1559360_at | TRUE |  | 1559360_at | TRUE |
| 1559545_at | TRUE |  | 1559545_at | TRUE |
| 1559584_a_at | TRUE |  | 1559584_a_at | TRUE |
| 1559821_at | TRUE |  | 1559821_at | TRUE |
| 1559946_s_at | TRUE |  | 1559946_s_at | TRUE |
| 1559979_at | TRUE |  | 1559979_at | TRUE |
| 1560019_at | TRUE |  | 1560019_at | TRUE |
| 1560068_a_at | TRUE |  | 1560068_a_at | TRUE |
| 1560094_at | TRUE |  | 1560094_at | TRUE |
| 1560108_at | TRUE |  | 1560108_at | TRUE |
| 1560142_at | TRUE |  | 1560142_at | TRUE |
| 1560250_s_at | TRUE |  | 1560250_s_at | TRUE |
| 1560265_at | TRUE |  | 1560265_at | TRUE |
| 1560316_s_at | TRUE |  | 1560316_s_at | TRUE |
| 1560433_at | TRUE |  | 1560433_at | TRUE |
| 1560482_at | TRUE |  | 1560482_at | TRUE |
| 1560503_a_at | TRUE |  | 1560503_a_at | TRUE |
| 1560648_s_at | TRUE |  | 1560648_s_at | TRUE |
| 1560692_at | TRUE |  | 1560692_at | TRUE |
| 1560703_at | TRUE |  | 1560703_at | TRUE |
| 1560734_at | TRUE |  | 1560734_at | TRUE |
| 1561158_at | TRUE |  | 1561158_at | TRUE |
| 1561642_at | TRUE |  | 1561642_at | TRUE |
| 1561726_s_at | TRUE |  | 1561726_s_at | TRUE |
| 1562240_at | TRUE |  | 1562240_at | TRUE |
| 1562275_at | TRUE |  | 1562275_at | TRUE |
| 1562380_at | TRUE |  | 1562380_at | TRUE |
| 1562443_at | TRUE |  | 1562443_at | TRUE |
| 1562458_at | TRUE |  | 1562458_at | TRUE |
| 1562940_at | TRUE |  | 1562940_at | TRUE |
| 1562991_at | TRUE |  | 1562991_at | TRUE |
| 1563187_at | TRUE |  | 1563187_at | TRUE |
| 1563321_s_at | TRUE |  | 1563321_s_at | TRUE |
| 1563680_at | TRUE |  | 1563680_at | TRUE |
| 1563809_a_at | TRUE |  | 1563809_a_at | TRUE |
| 1564807_at | TRUE |  | 1564807_at | TRUE |
| 1564856_s_at | TRUE |  | 1564856_s_at | TRUE |
| 1565034_s_at | TRUE |  | 1565034_s_at | TRUE |
| 1565690_at | TRUE |  | 1565690_at | TRUE |
| 1565833_at | TRUE |  | 1565833_at | TRUE |
| 1565951_s_at | TRUE |  | 1565951_s_at | TRUE |
| 1566108_at | TRUE |  | 1566108_at | TRUE |
| 1566324_a_at | TRUE |  | 1566324_a_at | TRUE |
| 1566513_a_at | TRUE |  | 1566513_a_at | TRUE |
| 1566772_at | TRUE |  | 1566772_at | TRUE |
| 1566901_at | TRUE |  | 1566901_at | TRUE |
| 1567628_at | TRUE |  | 1567628_at | TRUE |
| 1568612_at | TRUE |  | 1568612_at | TRUE |
| 1568617_a_at | TRUE |  | 1568617_a_at | TRUE |
| 1568619_s_at | TRUE |  | 1568619_s_at | TRUE |
| 1568625_at | TRUE |  | 1568625_at | TRUE |
| 1568656_at | TRUE |  | 1568656_at | TRUE |
| 1568791_s_at | TRUE |  | 1568791_s_at | TRUE |
| 1568870_at | TRUE |  | 1568870_at | TRUE |
| 1568873_at | TRUE |  | 1568873_at | TRUE |
| 1568931_at | TRUE |  | 1568931_at | TRUE |
| 1568934_at | TRUE |  | 1568934_at | TRUE |
| 1569146_s_at | TRUE |  | 1569146_s_at | TRUE |
| 1569631_at | TRUE |  | 1569631_at | TRUE |
| 1569652_at | TRUE |  | 1569652_at | TRUE |
| 1569656_at | TRUE |  | 1569656_at | TRUE |
| 1569998_at | TRUE |  | 1569998_at | TRUE |
| 1570107_at | TRUE |  | 1570107_at | TRUE |
| 1570136_at | TRUE |  | 1570136_at | TRUE |
| 1570190_at | TRUE |  | 1570190_at | TRUE |
| 1570339_x_at | TRUE |  | 1570339_x_at | TRUE |
| 1570395_a_at | TRUE |  | 1570395_a_at | TRUE |
| 1570397_x_at | TRUE |  | 1570397_x_at | TRUE |
| 200001_at | TRUE |  | 200001_at | TRUE |
| 200070_at | TRUE |  | 200070_at | TRUE |
| 200600_at | TRUE |  | 200600_at | TRUE |
| 200601_at | TRUE |  | 200601_at | TRUE |
| 200620_at | TRUE |  | 200620_at | TRUE |
| 200625_s_at | TRUE |  | 200625_s_at | TRUE |
| 200632_s_at | TRUE |  | 200632_s_at | TRUE |
| 200649_at | TRUE |  | 200649_at | TRUE |
| 200650_s_at | TRUE |  | 200650_s_at | TRUE |
| 200660_at | TRUE |  | 200660_at | TRUE |
| 200686_s_at | TRUE |  | 200686_s_at | TRUE |
| 200696_s_at | TRUE |  | 200696_s_at | TRUE |
| 200758_s_at | TRUE |  | 200758_s_at | TRUE |
| 200759_x_at | TRUE |  | 200759_x_at | TRUE |
| 200782_at | TRUE |  | 200782_at | TRUE |
| 200791_s_at | TRUE |  | 200791_s_at | TRUE |
| 200813_s_at | TRUE |  | 200813_s_at | TRUE |
| 200827_at | TRUE |  | 200827_at | TRUE |
| 200859_x_at | TRUE |  | 200859_x_at | TRUE |
| 200872_at | TRUE |  | 200872_at | TRUE |
| 200878_at | TRUE |  | 200878_at | TRUE |
| 200879_s_at | TRUE |  | 200879_s_at | TRUE |
| 200885_at | TRUE |  | 200885_at | TRUE |
| 200902_at | TRUE |  | 200902_at | TRUE |
| 200904_at | TRUE |  | 200904_at | TRUE |
| 200905_x_at | TRUE |  | 200905_x_at | TRUE |
| 200919_at | TRUE |  | 200919_at | TRUE |
| 200922_at | TRUE |  | 200922_at | TRUE |
| 200931_s_at | TRUE |  | 200931_s_at | TRUE |
| 200975_at | TRUE |  | 200975_at | TRUE |
| 201003_x_at | TRUE |  | 201003_x_at | TRUE |
| 201012_at | TRUE |  | 201012_at | TRUE |
| 201064_s_at | TRUE |  | 201064_s_at | TRUE |
| 201125_s_at | TRUE |  | 201125_s_at | TRUE |
| 201137_s_at | TRUE |  | 201137_s_at | TRUE |
| 201155_s_at | TRUE |  | 201155_s_at | TRUE |
| 201160_s_at | TRUE |  | 201160_s_at | TRUE |
| 201161_s_at | TRUE |  | 201161_s_at | TRUE |
| 201177_s_at | TRUE |  | 201177_s_at | TRUE |
| 201180_s_at | TRUE |  | 201180_s_at | TRUE |
| 201183_s_at | TRUE |  | 201183_s_at | TRUE |
| 201209_at | TRUE |  | 201209_at | TRUE |
| 201214_s_at | TRUE |  | 201214_s_at | TRUE |
| 201215_at | TRUE |  | 201215_at | TRUE |
| 201224_s_at | TRUE |  | 201224_s_at | TRUE |
| 201225_s_at | TRUE |  | 201225_s_at | TRUE |
| 201247_at | TRUE |  | 201247_at | TRUE |
| 201275_at | TRUE |  | 201275_at | TRUE |
| 201288_at | TRUE |  | 201288_at | TRUE |
| 201309_x_at | TRUE |  | 201309_x_at | TRUE |
| 201310_s_at | TRUE |  | 201310_s_at | TRUE |
| 201315_x_at | TRUE |  | 201315_x_at | TRUE |
| 201324_at | TRUE |  | 201324_at | TRUE |
| 201325_s_at | TRUE |  | 201325_s_at | TRUE |
| 201336_at | TRUE |  | 201336_at | TRUE |
| 201339_s_at | TRUE |  | 201339_s_at | TRUE |
| 201356_at | TRUE |  | 201356_at | TRUE |
| 201357_s_at | TRUE |  | 201357_s_at | TRUE |
| 201361_at | TRUE |  | 201361_at | TRUE |
| 201367_s_at | TRUE |  | 201367_s_at | TRUE |
| 201368_at | TRUE |  | 201368_at | TRUE |
| 201369_s_at | TRUE |  | 201369_s_at | TRUE |
| 201370_s_at | TRUE |  | 201370_s_at | TRUE |
| 201419_at | TRUE |  | 201419_at | TRUE |
| 201420_s_at | TRUE |  | 201420_s_at | TRUE |
| 201421_s_at | TRUE |  | 201421_s_at | TRUE |
| 201426_s_at | TRUE |  | 201426_s_at | TRUE |
| 201445_at | TRUE |  | 201445_at | TRUE |
| 201464_x_at | TRUE |  | 201464_x_at | TRUE |
| 201465_s_at | TRUE |  | 201465_s_at | TRUE |
| 201466_s_at | TRUE |  | 201466_s_at | TRUE |
| 201481_s_at | TRUE |  | 201481_s_at | TRUE |
| 201487_at | TRUE |  | 201487_at | TRUE |
| 201493_s_at | TRUE |  | 201493_s_at | TRUE |
| 201516_at | TRUE |  | 201516_at | TRUE |
| 201535_at | TRUE |  | 201535_at | TRUE |
| 201590_x_at | TRUE |  | 201590_x_at | TRUE |
| 201598_s_at | TRUE |  | 201598_s_at | TRUE |
| 201601_x_at | TRUE |  | 201601_x_at | TRUE |
| 201625_s_at | TRUE |  | 201625_s_at | TRUE |
| 201626_at | TRUE |  | 201626_at | TRUE |
| 201627_s_at | TRUE |  | 201627_s_at | TRUE |
| 201630_s_at | TRUE |  | 201630_s_at | TRUE |
| 201641_at | TRUE |  | 201641_at | TRUE |
| 201696_at | TRUE |  | 201696_at | TRUE |
| 201720_s_at | TRUE |  | 201720_s_at | TRUE |
| 201721_s_at | TRUE |  | 201721_s_at | TRUE |
| 201743_at | TRUE |  | 201743_at | TRUE |
| 201770_at | TRUE |  | 201770_at | TRUE |
| 201778_s_at | TRUE |  | 201778_s_at | TRUE |
| 201804_x_at | TRUE |  | 201804_x_at | TRUE |
| 201838_s_at | TRUE |  | 201838_s_at | TRUE |
| 201850_at | TRUE |  | 201850_at | TRUE |
| 201883_s_at | TRUE |  | 201883_s_at | TRUE |
| 201887_at | TRUE |  | 201887_at | TRUE |
| 201888_s_at | TRUE |  | 201888_s_at | TRUE |
| 201932_at | TRUE |  | 201932_at | TRUE |
| 201935_s_at | TRUE |  | 201935_s_at | TRUE |
| 201936_s_at | TRUE |  | 201936_s_at | TRUE |
| 201948_at | TRUE |  | 201948_at | TRUE |
| 201949_x_at | TRUE |  | 201949_x_at | TRUE |
| 201950_x_at | TRUE |  | 201950_x_at | TRUE |
| 201954_at | TRUE |  | 201954_at | TRUE |
| 201997_s_at | TRUE |  | 201997_s_at | TRUE |
| 201998_at | TRUE |  | 201998_at | TRUE |
| 202022_at | TRUE |  | 202022_at | TRUE |
| 202023_at | TRUE |  | 202023_at | TRUE |
| 202033_s_at | TRUE |  | 202033_s_at | TRUE |
| 202034_x_at | TRUE |  | 202034_x_at | TRUE |
| 202057_at | TRUE |  | 202057_at | TRUE |
| 202067_s_at | TRUE |  | 202067_s_at | TRUE |
| 202068_s_at | TRUE |  | 202068_s_at | TRUE |
| 202074_s_at | TRUE |  | 202074_s_at | TRUE |
| 202093_s_at | TRUE |  | 202093_s_at | TRUE |
| 202098_s_at | TRUE |  | 202098_s_at | TRUE |
| 202108_at | TRUE |  | 202108_at | TRUE |
| 202111_at | TRUE |  | 202111_at | TRUE |
| 202115_s_at | TRUE |  | 202115_s_at | TRUE |
| 202120_x_at | TRUE |  | 202120_x_at | TRUE |
| 202132_at | TRUE |  | 202132_at | TRUE |
| 202133_at | TRUE |  | 202133_at | TRUE |
| 202150_s_at | TRUE |  | 202150_s_at | TRUE |
| 202152_x_at | TRUE |  | 202152_x_at | TRUE |
| 202153_s_at | TRUE |  | 202153_s_at | TRUE |
| 202193_at | TRUE |  | 202193_at | TRUE |
| 202194_at | TRUE |  | 202194_at | TRUE |
| 202201_at | TRUE |  | 202201_at | TRUE |
| 202202_s_at | TRUE |  | 202202_s_at | TRUE |
| 202205_at | TRUE |  | 202205_at | TRUE |
| 202241_at | TRUE |  | 202241_at | TRUE |
| 202252_at | TRUE |  | 202252_at | TRUE |
| 202260_s_at | TRUE |  | 202260_s_at | TRUE |
| 202262_x_at | TRUE |  | 202262_x_at | TRUE |
| 202264_s_at | TRUE |  | 202264_s_at | TRUE |
| 202269_x_at | TRUE |  | 202269_x_at | TRUE |
| 202270_at | TRUE |  | 202270_at | TRUE |
| 202288_at | TRUE |  | 202288_at | TRUE |
| 202295_s_at | TRUE |  | 202295_s_at | TRUE |
| 202297_s_at | TRUE |  | 202297_s_at | TRUE |
| 202299_s_at | TRUE |  | 202299_s_at | TRUE |
| 202336_s_at | TRUE |  | 202336_s_at | TRUE |
| 202362_at | TRUE |  | 202362_at | TRUE |
| 202365_at | TRUE |  | 202365_at | TRUE |
| 202368_s_at | TRUE |  | 202368_s_at | TRUE |
| 202377_at | TRUE |  | 202377_at | TRUE |
| 202391_at | TRUE |  | 202391_at | TRUE |
| 202408_s_at | TRUE |  | 202408_s_at | TRUE |
| 202412_s_at | TRUE |  | 202412_s_at | TRUE |
| 202443_x_at | TRUE |  | 202443_x_at | TRUE |
| 202445_s_at | TRUE |  | 202445_s_at | TRUE |
| 202457_s_at | TRUE |  | 202457_s_at | TRUE |
| 202464_s_at | TRUE |  | 202464_s_at | TRUE |
| 202470_s_at | TRUE |  | 202470_s_at | TRUE |
| 202475_at | TRUE |  | 202475_at | TRUE |
| 202481_at | TRUE |  | 202481_at | TRUE |
| 202502_at | TRUE |  | 202502_at | TRUE |
| 202503_s_at | TRUE |  | 202503_s_at | TRUE |
| 202507_s_at | TRUE |  | 202507_s_at | TRUE |
| 202508_s_at | TRUE |  | 202508_s_at | TRUE |
| 202514_at | TRUE |  | 202514_at | TRUE |
| 202515_at | TRUE |  | 202515_at | TRUE |
| 202516_s_at | TRUE |  | 202516_s_at | TRUE |
| 202522_at | TRUE |  | 202522_at | TRUE |
| 202528_at | TRUE |  | 202528_at | TRUE |
| 202539_s_at | TRUE |  | 202539_s_at | TRUE |
| 202540_s_at | TRUE |  | 202540_s_at | TRUE |
| 202546_at | TRUE |  | 202546_at | TRUE |
| 202553_s_at | TRUE |  | 202553_s_at | TRUE |
| 202564_x_at | TRUE |  | 202564_x_at | TRUE |
| 202619_s_at | TRUE |  | 202619_s_at | TRUE |
| 202620_s_at | TRUE |  | 202620_s_at | TRUE |
| 202621_at | TRUE |  | 202621_at | TRUE |
| 202625_at | TRUE |  | 202625_at | TRUE |
| 202626_s_at | TRUE |  | 202626_s_at | TRUE |
| 202636_at | TRUE |  | 202636_at | TRUE |
| 202646_s_at | TRUE |  | 202646_s_at | TRUE |
| 202664_at | TRUE |  | 202664_at | TRUE |
| 202672_s_at | TRUE |  | 202672_s_at | TRUE |
| 202686_s_at | TRUE |  | 202686_s_at | TRUE |
| 202687_s_at | TRUE |  | 202687_s_at | TRUE |
| 202688_at | TRUE |  | 202688_at | TRUE |
| 202712_s_at | TRUE |  | 202712_s_at | TRUE |
| 202748_at | TRUE |  | 202748_at | TRUE |
| 202754_at | TRUE |  | 202754_at | TRUE |
| 202770_s_at | TRUE |  | 202770_s_at | TRUE |
| 202772_at | TRUE |  | 202772_at | TRUE |
| 202803_s_at | TRUE |  | 202803_s_at | TRUE |
| 202819_s_at | TRUE |  | 202819_s_at | TRUE |
| 202833_s_at | TRUE |  | 202833_s_at | TRUE |
| 202838_at | TRUE |  | 202838_at | TRUE |
| 202856_s_at | TRUE |  | 202856_s_at | TRUE |
| 202863_at | TRUE |  | 202863_at | TRUE |
| 202864_s_at | TRUE |  | 202864_s_at | TRUE |
| 202868_s_at | TRUE |  | 202868_s_at | TRUE |
| 202901_x_at | TRUE |  | 202901_x_at | TRUE |
| 202902_s_at | TRUE |  | 202902_s_at | TRUE |
| 202934_at | TRUE |  | 202934_at | TRUE |
| 202939_at | TRUE |  | 202939_at | TRUE |
| 202943_s_at | TRUE |  | 202943_s_at | TRUE |
| 202944_at | TRUE |  | 202944_at | TRUE |
| 202947_s_at | TRUE |  | 202947_s_at | TRUE |
| 202950_at | TRUE |  | 202950_at | TRUE |
| 202951_at | TRUE |  | 202951_at | TRUE |
| 202953_at | TRUE |  | 202953_at | TRUE |
| 202957_at | TRUE |  | 202957_at | TRUE |
| 202990_at | TRUE |  | 202990_at | TRUE |
| 202997_s_at | TRUE |  | 202997_s_at | TRUE |
| 203000_at | TRUE |  | 203000_at | TRUE |
| 203001_s_at | TRUE |  | 203001_s_at | TRUE |
| 203020_at | TRUE |  | 203020_at | TRUE |
| 203027_s_at | TRUE |  | 203027_s_at | TRUE |
| 203028_s_at | TRUE |  | 203028_s_at | TRUE |
| 203030_s_at | TRUE |  | 203030_s_at | TRUE |
| 203055_s_at | TRUE |  | 203055_s_at | TRUE |
| 203068_at | TRUE |  | 203068_at | TRUE |
| 203073_at | TRUE |  | 203073_at | TRUE |
| 203085_s_at | TRUE |  | 203085_s_at | TRUE |
| 203104_at | TRUE |  | 203104_at | TRUE |
| 203145_at | TRUE |  | 203145_at | TRUE |
| 203146_s_at | TRUE |  | 203146_s_at | TRUE |
| 203175_at | TRUE |  | 203175_at | TRUE |
| 203178_at | TRUE |  | 203178_at | TRUE |
| 203185_at | TRUE |  | 203185_at | TRUE |
| 203197_s_at | TRUE |  | 203197_s_at | TRUE |
| 203205_at | TRUE |  | 203205_at | TRUE |
| 203208_s_at | TRUE |  | 203208_s_at | TRUE |
| 203221_at | TRUE |  | 203221_at | TRUE |
| 203236_s_at | TRUE |  | 203236_s_at | TRUE |
| 203240_at | TRUE |  | 203240_at | TRUE |
| 203248_at | TRUE |  | 203248_at | TRUE |
| 203254_s_at | TRUE |  | 203254_s_at | TRUE |
| 203263_s_at | TRUE |  | 203263_s_at | TRUE |
| 203264_s_at | TRUE |  | 203264_s_at | TRUE |
| 203266_s_at | TRUE |  | 203266_s_at | TRUE |
| 203267_s_at | TRUE |  | 203267_s_at | TRUE |
| 203268_s_at | TRUE |  | 203268_s_at | TRUE |
| 203271_s_at | TRUE |  | 203271_s_at | TRUE |
| 203277_at | TRUE |  | 203277_at | TRUE |
| 203282_at | TRUE |  | 203282_at | TRUE |
| 203283_s_at | TRUE |  | 203283_s_at | TRUE |
| 203284_s_at | TRUE |  | 203284_s_at | TRUE |
| 203285_s_at | TRUE |  | 203285_s_at | TRUE |
| 203310_at | TRUE |  | 203310_at | TRUE |
| 203313_s_at | TRUE |  | 203313_s_at | TRUE |
| 203317_at | TRUE |  | 203317_at | TRUE |
| 203320_at | TRUE |  | 203320_at | TRUE |
| 203331_s_at | TRUE |  | 203331_s_at | TRUE |
| 203332_s_at | TRUE |  | 203332_s_at | TRUE |
| 203339_at | TRUE |  | 203339_at | TRUE |
| 203340_s_at | TRUE |  | 203340_s_at | TRUE |
| 203345_s_at | TRUE |  | 203345_s_at | TRUE |
| 203359_s_at | TRUE |  | 203359_s_at | TRUE |
| 203360_s_at | TRUE |  | 203360_s_at | TRUE |
| 203379_at | TRUE |  | 203379_at | TRUE |
| 203388_at | TRUE |  | 203388_at | TRUE |
| 203389_at | TRUE |  | 203389_at | TRUE |
| 203390_s_at | TRUE |  | 203390_s_at | TRUE |
| 203416_at | TRUE |  | 203416_at | TRUE |
| 203434_s_at | TRUE |  | 203434_s_at | TRUE |
| 203435_s_at | TRUE |  | 203435_s_at | TRUE |
| 203442_x_at | TRUE |  | 203442_x_at | TRUE |
| 203445_s_at | TRUE |  | 203445_s_at | TRUE |
| 203454_s_at | TRUE |  | 203454_s_at | TRUE |
| 203470_s_at | TRUE |  | 203470_s_at | TRUE |
| 203471_s_at | TRUE |  | 203471_s_at | TRUE |
| 203473_at | TRUE |  | 203473_at | TRUE |
| 203474_at | TRUE |  | 203474_at | TRUE |
| 203476_at | TRUE |  | 203476_at | TRUE |
| 203490_at | TRUE |  | 203490_at | TRUE |
| 203502_at | TRUE |  | 203502_at | TRUE |
| 203503_s_at | TRUE |  | 203503_s_at | TRUE |
| 203505_at | TRUE |  | 203505_at | TRUE |
| 203507_at | TRUE |  | 203507_at | TRUE |
| 203508_at | TRUE |  | 203508_at | TRUE |
| 203511_s_at | TRUE |  | 203511_s_at | TRUE |
| 203512_at | TRUE |  | 203512_at | TRUE |
| 203527_s_at | TRUE |  | 203527_s_at | TRUE |
| 203547_at | TRUE |  | 203547_at | TRUE |
| 203561_at | TRUE |  | 203561_at | TRUE |
| 203567_s_at | TRUE |  | 203567_s_at | TRUE |
| 203568_s_at | TRUE |  | 203568_s_at | TRUE |
| 203576_at | TRUE |  | 203576_at | TRUE |
| 203591_s_at | TRUE |  | 203591_s_at | TRUE |
| 203623_at | TRUE |  | 203623_at | TRUE |
| 203643_at | TRUE |  | 203643_at | TRUE |
| 203646_at | TRUE |  | 203646_at | TRUE |
| 203665_at | TRUE |  | 203665_at | TRUE |
| 203703_s_at | TRUE |  | 203703_s_at | TRUE |
| 203719_at | TRUE |  | 203719_at | TRUE |
| 203720_s_at | TRUE |  | 203720_s_at | TRUE |
| 203722_at | TRUE |  | 203722_at | TRUE |
| 203739_at | TRUE |  | 203739_at | TRUE |
| 203741_s_at | TRUE |  | 203741_s_at | TRUE |
| 203748_x_at | TRUE |  | 203748_x_at | TRUE |
| 203797_at | TRUE |  | 203797_at | TRUE |
| 203798_s_at | TRUE |  | 203798_s_at | TRUE |
| 203810_at | TRUE |  | 203810_at | TRUE |
| 203818_s_at | TRUE |  | 203818_s_at | TRUE |
| 203838_s_at | TRUE |  | 203838_s_at | TRUE |
| 203857_s_at | TRUE |  | 203857_s_at | TRUE |
| 203900_at | TRUE |  | 203900_at | TRUE |
| 203911_at | TRUE |  | 203911_at | TRUE |
| 203922_s_at | TRUE |  | 203922_s_at | TRUE |
| 203923_s_at | TRUE |  | 203923_s_at | TRUE |
| 203932_at | TRUE |  | 203932_at | TRUE |
| 203958_s_at | TRUE |  | 203958_s_at | TRUE |
| 203959_s_at | TRUE |  | 203959_s_at | TRUE |
| 203998_s_at | TRUE |  | 203998_s_at | TRUE |
| 203999_at | TRUE |  | 203999_at | TRUE |
| 204006_s_at | TRUE |  | 204006_s_at | TRUE |
| 204007_at | TRUE |  | 204007_at | TRUE |
| 204034_at | TRUE |  | 204034_at | TRUE |
| 204039_at | TRUE |  | 204039_at | TRUE |
| 204046_at | TRUE |  | 204046_at | TRUE |
| 204057_at | TRUE |  | 204057_at | TRUE |
| 204068_at | TRUE |  | 204068_at | TRUE |
| 204070_at | TRUE |  | 204070_at | TRUE |
| 204074_s_at | TRUE |  | 204074_s_at | TRUE |
| 204075_s_at | TRUE |  | 204075_s_at | TRUE |
| 204081_at | TRUE |  | 204081_at | TRUE |
| 204104_at | TRUE |  | 204104_at | TRUE |
| 204120_s_at | TRUE |  | 204120_s_at | TRUE |
| 204122_at | TRUE |  | 204122_at | TRUE |
| 204132_s_at | TRUE |  | 204132_s_at | TRUE |
| 204134_at | TRUE |  | 204134_at | TRUE |
| 204140_at | TRUE |  | 204140_at | TRUE |
| 204153_s_at | TRUE |  | 204153_s_at | TRUE |
| 204158_s_at | TRUE |  | 204158_s_at | TRUE |
| 204164_at | TRUE |  | 204164_at | TRUE |
| 204168_at | TRUE |  | 204168_at | TRUE |
| 204175_at | TRUE |  | 204175_at | TRUE |
| 204190_at | TRUE |  | 204190_at | TRUE |
| 204192_at | TRUE |  | 204192_at | TRUE |
| 204197_s_at | TRUE |  | 204197_s_at | TRUE |
| 204205_at | TRUE |  | 204205_at | TRUE |
| 204214_s_at | TRUE |  | 204214_s_at | TRUE |
| 204215_at | TRUE |  | 204215_at | TRUE |
| 204220_at | TRUE |  | 204220_at | TRUE |
| 204225_at | TRUE |  | 204225_at | TRUE |
| 204226_at | TRUE |  | 204226_at | TRUE |
| 204228_at | TRUE |  | 204228_at | TRUE |
| 204229_at | TRUE |  | 204229_at | TRUE |
| 204230_s_at | TRUE |  | 204230_s_at | TRUE |
| 204232_at | TRUE |  | 204232_at | TRUE |
| 204236_at | TRUE |  | 204236_at | TRUE |
| 204249_s_at | TRUE |  | 204249_s_at | TRUE |
| 204260_at | TRUE |  | 204260_at | TRUE |
| 204264_at | TRUE |  | 204264_at | TRUE |
| 204265_s_at | TRUE |  | 204265_s_at | TRUE |
| 204270_at | TRUE |  | 204270_at | TRUE |
| 204316_at | TRUE |  | 204316_at | TRUE |
| 204319_s_at | TRUE |  | 204319_s_at | TRUE |
| 204320_at | TRUE |  | 204320_at | TRUE |
| 204336_s_at | TRUE |  | 204336_s_at | TRUE |
| 204337_at | TRUE |  | 204337_at | TRUE |
| 204338_s_at | TRUE |  | 204338_s_at | TRUE |
| 204339_s_at | TRUE |  | 204339_s_at | TRUE |
| 204369_at | TRUE |  | 204369_at | TRUE |
| 204411_at | TRUE |  | 204411_at | TRUE |
| 204412_s_at | TRUE |  | 204412_s_at | TRUE |
| 204425_at | TRUE |  | 204425_at | TRUE |
| 204430_s_at | TRUE |  | 204430_s_at | TRUE |
| 204446_s_at | TRUE |  | 204446_s_at | TRUE |
| 204463_s_at | TRUE |  | 204463_s_at | TRUE |
| 204464_s_at | TRUE |  | 204464_s_at | TRUE |
| 204465_s_at | TRUE |  | 204465_s_at | TRUE |
| 204472_at | TRUE |  | 204472_at | TRUE |
| 204487_s_at | TRUE |  | 204487_s_at | TRUE |
| 204489_s_at | TRUE |  | 204489_s_at | TRUE |
| 204490_s_at | TRUE |  | 204490_s_at | TRUE |
| 204493_at | TRUE |  | 204493_at | TRUE |
| 204494_s_at | TRUE |  | 204494_s_at | TRUE |
| 204495_s_at | TRUE |  | 204495_s_at | TRUE |
| 204505_s_at | TRUE |  | 204505_s_at | TRUE |
| 204573_at | TRUE |  | 204573_at | TRUE |
| 204584_at | TRUE |  | 204584_at | TRUE |
| 204585_s_at | TRUE |  | 204585_s_at | TRUE |
| 204588_s_at | TRUE |  | 204588_s_at | TRUE |
| 204612_at | TRUE |  | 204612_at | TRUE |
| 204613_at | TRUE |  | 204613_at | TRUE |
| 204624_at | TRUE |  | 204624_at | TRUE |
| 204639_at | TRUE |  | 204639_at | TRUE |
| 204670_x_at | TRUE |  | 204670_x_at | TRUE |
| 204685_s_at | TRUE |  | 204685_s_at | TRUE |
| 204689_at | TRUE |  | 204689_at | TRUE |
| 204697_s_at | TRUE |  | 204697_s_at | TRUE |
| 204718_at | TRUE |  | 204718_at | TRUE |
| 204722_at | TRUE |  | 204722_at | TRUE |
| 204723_at | TRUE |  | 204723_at | TRUE |
| 204729_s_at | TRUE |  | 204729_s_at | TRUE |
| 204737_s_at | TRUE |  | 204737_s_at | TRUE |
| 204743_at | TRUE |  | 204743_at | TRUE |
| 204749_at | TRUE |  | 204749_at | TRUE |
| 204773_at | TRUE |  | 204773_at | TRUE |
| 204786_s_at | TRUE |  | 204786_s_at | TRUE |
| 204787_at | TRUE |  | 204787_at | TRUE |
| 204811_s_at | TRUE |  | 204811_s_at | TRUE |
| 204813_at | TRUE |  | 204813_at | TRUE |
| 204834_at | TRUE |  | 204834_at | TRUE |
| 204846_at | TRUE |  | 204846_at | TRUE |
| 204869_at | TRUE |  | 204869_at | TRUE |
| 204870_s_at | TRUE |  | 204870_s_at | TRUE |
| 204882_at | TRUE |  | 204882_at | TRUE |
| 204897_at | TRUE |  | 204897_at | TRUE |
| 204908_s_at | TRUE |  | 204908_s_at | TRUE |
| 204912_at | TRUE |  | 204912_at | TRUE |
| 204923_at | TRUE |  | 204923_at | TRUE |
| 204928_s_at | TRUE |  | 204928_s_at | TRUE |
| 204945_at | TRUE |  | 204945_at | TRUE |
| 204953_at | TRUE |  | 204953_at | TRUE |
| 204959_at | TRUE |  | 204959_at | TRUE |
| 204961_s_at | TRUE |  | 204961_s_at | TRUE |
| 204989_s_at | TRUE |  | 204989_s_at | TRUE |
| 204990_s_at | TRUE |  | 204990_s_at | TRUE |
| 205002_at | TRUE |  | 205002_at | TRUE |
| 205039_s_at | TRUE |  | 205039_s_at | TRUE |
| 205051_s_at | TRUE |  | 205051_s_at | TRUE |
| 205087_at | TRUE |  | 205087_at | TRUE |
| 205095_s_at | TRUE |  | 205095_s_at | TRUE |
| 205098_at | TRUE |  | 205098_at | TRUE |
| 205099_s_at | TRUE |  | 205099_s_at | TRUE |
| 205111_s_at | TRUE |  | 205111_s_at | TRUE |
| 205112_at | TRUE |  | 205112_at | TRUE |
| 205113_at | TRUE |  | 205113_at | TRUE |
| 205119_s_at | TRUE |  | 205119_s_at | TRUE |
| 205128_x_at | TRUE |  | 205128_x_at | TRUE |
| 205140_at | TRUE |  | 205140_at | TRUE |
| 205141_at | TRUE |  | 205141_at | TRUE |
| 205143_at | TRUE |  | 205143_at | TRUE |
| 205147_x_at | TRUE |  | 205147_x_at | TRUE |
| 205152_at | TRUE |  | 205152_at | TRUE |
| 205158_at | TRUE |  | 205158_at | TRUE |
| 205159_at | TRUE |  | 205159_at | TRUE |
| 205165_at | TRUE |  | 205165_at | TRUE |
| 205173_x_at | TRUE |  | 205173_x_at | TRUE |
| 205176_s_at | TRUE |  | 205176_s_at | TRUE |
| 205184_at | TRUE |  | 205184_at | TRUE |
| 205186_at | TRUE |  | 205186_at | TRUE |
| 205230_at | TRUE |  | 205230_at | TRUE |
| 205232_s_at | TRUE |  | 205232_s_at | TRUE |
| 205233_s_at | TRUE |  | 205233_s_at | TRUE |
| 205234_at | TRUE |  | 205234_at | TRUE |
| 205245_at | TRUE |  | 205245_at | TRUE |
| 205263_at | TRUE |  | 205263_at | TRUE |
| 205264_at | TRUE |  | 205264_at | TRUE |
| 205269_at | TRUE |  | 205269_at | TRUE |
| 205270_s_at | TRUE |  | 205270_s_at | TRUE |
| 205282_at | TRUE |  | 205282_at | TRUE |
| 205285_s_at | TRUE |  | 205285_s_at | TRUE |
| 205288_at | TRUE |  | 205288_at | TRUE |
| 205289_at | TRUE |  | 205289_at | TRUE |
| 205290_s_at | TRUE |  | 205290_s_at | TRUE |
| 205312_at | TRUE |  | 205312_at | TRUE |
| 205322_s_at | TRUE |  | 205322_s_at | TRUE |
| 205323_s_at | TRUE |  | 205323_s_at | TRUE |
| 205336_at | TRUE |  | 205336_at | TRUE |
| 205344_at | TRUE |  | 205344_at | TRUE |
| 205349_at | TRUE |  | 205349_at | TRUE |
| 205382_s_at | TRUE |  | 205382_s_at | TRUE |
| 205414_s_at | TRUE |  | 205414_s_at | TRUE |
| 205436_s_at | TRUE |  | 205436_s_at | TRUE |
| 205437_at | TRUE |  | 205437_at | TRUE |
| 205465_x_at | TRUE |  | 205465_x_at | TRUE |
| 205466_s_at | TRUE |  | 205466_s_at | TRUE |
| 205467_at | TRUE |  | 205467_at | TRUE |
| 205504_at | TRUE |  | 205504_at | TRUE |
| 205509_at | TRUE |  | 205509_at | TRUE |
| 205521_at | TRUE |  | 205521_at | TRUE |
| 205524_s_at | TRUE |  | 205524_s_at | TRUE |
| 205545_x_at | TRUE |  | 205545_x_at | TRUE |
| 205551_at | TRUE |  | 205551_at | TRUE |
| 205555_s_at | TRUE |  | 205555_s_at | TRUE |
| 205573_s_at | TRUE |  | 205573_s_at | TRUE |
| 205591_at | TRUE |  | 205591_at | TRUE |
| 205625_s_at | TRUE |  | 205625_s_at | TRUE |
| 205626_s_at | TRUE |  | 205626_s_at | TRUE |
| 205630_at | TRUE |  | 205630_at | TRUE |
| 205635_at | TRUE |  | 205635_at | TRUE |
| 205638_at | TRUE |  | 205638_at | TRUE |
| 205639_at | TRUE |  | 205639_at | TRUE |
| 205640_at | TRUE |  | 205640_at | TRUE |
| 205668_at | TRUE |  | 205668_at | TRUE |
| 205678_at | TRUE |  | 205678_at | TRUE |
| 205685_at | TRUE |  | 205685_at | TRUE |
| 205686_s_at | TRUE |  | 205686_s_at | TRUE |
| 205697_at | TRUE |  | 205697_at | TRUE |
| 205699_at | TRUE |  | 205699_at | TRUE |
| 205715_at | TRUE |  | 205715_at | TRUE |
| 205723_at | TRUE |  | 205723_at | TRUE |
| 205735_s_at | TRUE |  | 205735_s_at | TRUE |
| 205736_at | TRUE |  | 205736_at | TRUE |
| 205740_s_at | TRUE |  | 205740_s_at | TRUE |
| 205763_s_at | TRUE |  | 205763_s_at | TRUE |
| 205777_at | TRUE |  | 205777_at | TRUE |
| 205786_s_at | TRUE |  | 205786_s_at | TRUE |
| 205791_x_at | TRUE |  | 205791_x_at | TRUE |
| 205803_s_at | TRUE |  | 205803_s_at | TRUE |
| 205806_at | TRUE |  | 205806_at | TRUE |
| 205822_s_at | TRUE |  | 205822_s_at | TRUE |
| 205827_at | TRUE |  | 205827_at | TRUE |
| 205850_s_at | TRUE |  | 205850_s_at | TRUE |
| 205852_at | TRUE |  | 205852_at | TRUE |
| 205854_at | TRUE |  | 205854_at | TRUE |
| 205859_at | TRUE |  | 205859_at | TRUE |
| 205898_at | TRUE |  | 205898_at | TRUE |
| 205945_at | TRUE |  | 205945_at | TRUE |
| 205952_at | TRUE |  | 205952_at | TRUE |
| 205976_at | TRUE |  | 205976_at | TRUE |
| 205988_at | TRUE |  | 205988_at | TRUE |
| 205990_s_at | TRUE |  | 205990_s_at | TRUE |
| 205996_s_at | TRUE |  | 205996_s_at | TRUE |
| 205997_at | TRUE |  | 205997_at | TRUE |
| 206011_at | TRUE |  | 206011_at | TRUE |
| 206013_s_at | TRUE |  | 206013_s_at | TRUE |
| 206014_at | TRUE |  | 206014_at | TRUE |
| 206015_s_at | TRUE |  | 206015_s_at | TRUE |
| 206023_at | TRUE |  | 206023_at | TRUE |
| 206101_at | TRUE |  | 206101_at | TRUE |
| 206111_at | TRUE |  | 206111_at | TRUE |
| 206120_at | TRUE |  | 206120_at | TRUE |
| 206137_at | TRUE |  | 206137_at | TRUE |
| 206167_s_at | TRUE |  | 206167_s_at | TRUE |
| 206171_at | TRUE |  | 206171_at | TRUE |
| 206196_s_at | TRUE |  | 206196_s_at | TRUE |
| 206208_at | TRUE |  | 206208_at | TRUE |
| 206219_s_at | TRUE |  | 206219_s_at | TRUE |
| 206220_s_at | TRUE |  | 206220_s_at | TRUE |
| 206221_at | TRUE |  | 206221_at | TRUE |
| 206223_at | TRUE |  | 206223_at | TRUE |
| 206241_at | TRUE |  | 206241_at | TRUE |
| 206247_at | TRUE |  | 206247_at | TRUE |
| 206278_at | TRUE |  | 206278_at | TRUE |
| 206283_s_at | TRUE |  | 206283_s_at | TRUE |
| 206290_s_at | TRUE |  | 206290_s_at | TRUE |
| 206295_at | TRUE |  | 206295_at | TRUE |
| 206298_at | TRUE |  | 206298_at | TRUE |
| 206330_s_at | TRUE |  | 206330_s_at | TRUE |
| 206332_s_at | TRUE |  | 206332_s_at | TRUE |
| 206343_s_at | TRUE |  | 206343_s_at | TRUE |
| 206363_at | TRUE |  | 206363_at | TRUE |
| 206369_s_at | TRUE |  | 206369_s_at | TRUE |
| 206370_at | TRUE |  | 206370_at | TRUE |
| 206381_at | TRUE |  | 206381_at | TRUE |
| 206384_at | TRUE |  | 206384_at | TRUE |
| 206404_at | TRUE |  | 206404_at | TRUE |
| 206420_at | TRUE |  | 206420_at | TRUE |
| 206425_s_at | TRUE |  | 206425_s_at | TRUE |
| 206429_at | TRUE |  | 206429_at | TRUE |
| 206436_at | TRUE |  | 206436_at | TRUE |
| 206443_at | TRUE |  | 206443_at | TRUE |
| 206445_s_at | TRUE |  | 206445_s_at | TRUE |
| 206463_s_at | TRUE |  | 206463_s_at | TRUE |
| 206480_at | TRUE |  | 206480_at | TRUE |
| 206522_at | TRUE |  | 206522_at | TRUE |
| 206527_at | TRUE |  | 206527_at | TRUE |
| 206584_at | TRUE |  | 206584_at | TRUE |
| 206600_s_at | TRUE |  | 206600_s_at | TRUE |
| 206657_s_at | TRUE |  | 206657_s_at | TRUE |
| 206678_at | TRUE |  | 206678_at | TRUE |
| 206687_s_at | TRUE |  | 206687_s_at | TRUE |
| 206695_x_at | TRUE |  | 206695_x_at | TRUE |
| 206711_at | TRUE |  | 206711_at | TRUE |
| 206726_at | TRUE |  | 206726_at | TRUE |
| 206734_at | TRUE |  | 206734_at | TRUE |
| 206772_at | TRUE |  | 206772_at | TRUE |
| 206780_at | TRUE |  | 206780_at | TRUE |
| 206849_at | TRUE |  | 206849_at | TRUE |
| 206885_x_at | TRUE |  | 206885_x_at | TRUE |
| 206902_s_at | TRUE |  | 206902_s_at | TRUE |
| 206972_s_at | TRUE |  | 206972_s_at | TRUE |
| 206984_s_at | TRUE |  | 206984_s_at | TRUE |
| 207020_at | TRUE |  | 207020_at | TRUE |
| 207030_s_at | TRUE |  | 207030_s_at | TRUE |
| 207039_at | TRUE |  | 207039_at | TRUE |
| 207060_at | TRUE |  | 207060_at | TRUE |
| 207070_at | TRUE |  | 207070_at | TRUE |
| 207075_at | TRUE |  | 207075_at | TRUE |
| 207079_s_at | TRUE |  | 207079_s_at | TRUE |
| 207082_at | TRUE |  | 207082_at | TRUE |
| 207085_x_at | TRUE |  | 207085_x_at | TRUE |
| 207090_x_at | TRUE |  | 207090_x_at | TRUE |
| 207099_s_at | TRUE |  | 207099_s_at | TRUE |
| 207104_x_at | TRUE |  | 207104_x_at | TRUE |
| 207135_at | TRUE |  | 207135_at | TRUE |
| 207156_at | TRUE |  | 207156_at | TRUE |
| 207157_s_at | TRUE |  | 207157_s_at | TRUE |
| 207164_s_at | TRUE |  | 207164_s_at | TRUE |
| 207170_s_at | TRUE |  | 207170_s_at | TRUE |
| 207177_at | TRUE |  | 207177_at | TRUE |
| 207196_s_at | TRUE |  | 207196_s_at | TRUE |
| 207232_s_at | TRUE |  | 207232_s_at | TRUE |
| 207233_s_at | TRUE |  | 207233_s_at | TRUE |
| 207238_s_at | TRUE |  | 207238_s_at | TRUE |
| 207266_x_at | TRUE |  | 207266_x_at | TRUE |
| 207304_at | TRUE |  | 207304_at | TRUE |
| 207358_x_at | TRUE |  | 207358_x_at | TRUE |
| 207361_at | TRUE |  | 207361_at | TRUE |
| 207375_s_at | TRUE |  | 207375_s_at | TRUE |
| 207428_x_at | TRUE |  | 207428_x_at | TRUE |
| 207440_at | TRUE |  | 207440_at | TRUE |
| 207455_at | TRUE |  | 207455_at | TRUE |
| 207530_s_at | TRUE |  | 207530_s_at | TRUE |
| 207540_s_at | TRUE |  | 207540_s_at | TRUE |
| 207541_s_at | TRUE |  | 207541_s_at | TRUE |
| 207545_s_at | TRUE |  | 207545_s_at | TRUE |
| 207557_s_at | TRUE |  | 207557_s_at | TRUE |
| 207564_x_at | TRUE |  | 207564_x_at | TRUE |
| 207643_s_at | TRUE |  | 207643_s_at | TRUE |
| 207655_s_at | TRUE |  | 207655_s_at | TRUE |
| 207658_s_at | TRUE |  | 207658_s_at | TRUE |
| 207677_s_at | TRUE |  | 207677_s_at | TRUE |
| 207691_x_at | TRUE |  | 207691_x_at | TRUE |
| 207697_x_at | TRUE |  | 207697_x_at | TRUE |
| 207768_at | TRUE |  | 207768_at | TRUE |
| 207772_s_at | TRUE |  | 207772_s_at | TRUE |
| 207781_s_at | TRUE |  | 207781_s_at | TRUE |
| 207801_s_at | TRUE |  | 207801_s_at | TRUE |
| 207805_s_at | TRUE |  | 207805_s_at | TRUE |
| 207853_s_at | TRUE |  | 207853_s_at | TRUE |
| 207857_at | TRUE |  | 207857_at | TRUE |
| 207872_s_at | TRUE |  | 207872_s_at | TRUE |
| 207873_x_at | TRUE |  | 207873_x_at | TRUE |
| 207876_s_at | TRUE |  | 207876_s_at | TRUE |
| 207907_at | TRUE |  | 207907_at | TRUE |
| 207957_s_at | TRUE |  | 207957_s_at | TRUE |
| 208017_s_at | TRUE |  | 208017_s_at | TRUE |
| 208018_s_at | TRUE |  | 208018_s_at | TRUE |
| 208024_s_at | TRUE |  | 208024_s_at | TRUE |
| 208037_s_at | TRUE |  | 208037_s_at | TRUE |
| 208064_s_at | TRUE |  | 208064_s_at | TRUE |
| 208065_at | TRUE |  | 208065_at | TRUE |
| 208066_s_at | TRUE |  | 208066_s_at | TRUE |
| 208071_s_at | TRUE |  | 208071_s_at | TRUE |
| 208073_x_at | TRUE |  | 208073_x_at | TRUE |
| 208123_at | TRUE |  | 208123_at | TRUE |
| 208130_s_at | TRUE |  | 208130_s_at | TRUE |
| 208213_s_at | TRUE |  | 208213_s_at | TRUE |
| 208221_s_at | TRUE |  | 208221_s_at | TRUE |
| 208258_s_at | TRUE |  | 208258_s_at | TRUE |
| 208306_x_at | TRUE |  | 208306_x_at | TRUE |
| 208320_at | TRUE |  | 208320_at | TRUE |
| 208321_s_at | TRUE |  | 208321_s_at | TRUE |
| 208374_s_at | TRUE |  | 208374_s_at | TRUE |
| 208387_s_at | TRUE |  | 208387_s_at | TRUE |
| 208405_s_at | TRUE |  | 208405_s_at | TRUE |
| 208427_s_at | TRUE |  | 208427_s_at | TRUE |
| 208451_s_at | TRUE |  | 208451_s_at | TRUE |
| 208492_at | TRUE |  | 208492_at | TRUE |
| 208504_x_at | TRUE |  | 208504_x_at | TRUE |
| 208523_x_at | TRUE |  | 208523_x_at | TRUE |
| 208540_x_at | TRUE |  | 208540_x_at | TRUE |
| 208546_x_at | TRUE |  | 208546_x_at | TRUE |
| 208603_s_at | TRUE |  | 208603_s_at | TRUE |
| 208615_s_at | TRUE |  | 208615_s_at | TRUE |
| 208617_s_at | TRUE |  | 208617_s_at | TRUE |
| 208628_s_at | TRUE |  | 208628_s_at | TRUE |
| 208633_s_at | TRUE |  | 208633_s_at | TRUE |
| 208634_s_at | TRUE |  | 208634_s_at | TRUE |
| 208659_at | TRUE |  | 208659_at | TRUE |
| 208661_s_at | TRUE |  | 208661_s_at | TRUE |
| 208664_s_at | TRUE |  | 208664_s_at | TRUE |
| 208668_x_at | TRUE |  | 208668_x_at | TRUE |
| 208674_x_at | TRUE |  | 208674_x_at | TRUE |
| 208675_s_at | TRUE |  | 208675_s_at | TRUE |
| 208680_at | TRUE |  | 208680_at | TRUE |
| 208709_s_at | TRUE |  | 208709_s_at | TRUE |
| 208712_at | TRUE |  | 208712_at | TRUE |
| 208713_at | TRUE |  | 208713_at | TRUE |
| 208728_s_at | TRUE |  | 208728_s_at | TRUE |
| 208756_at | TRUE |  | 208756_at | TRUE |
| 208782_at | TRUE |  | 208782_at | TRUE |
| 208784_s_at | TRUE |  | 208784_s_at | TRUE |
| 208789_at | TRUE |  | 208789_at | TRUE |
| 208806_at | TRUE |  | 208806_at | TRUE |
| 208816_x_at | TRUE |  | 208816_x_at | TRUE |
| 208839_s_at | TRUE |  | 208839_s_at | TRUE |
| 208846_s_at | TRUE |  | 208846_s_at | TRUE |
| 208858_s_at | TRUE |  | 208858_s_at | TRUE |
| 208885_at | TRUE |  | 208885_at | TRUE |
| 208894_at | TRUE |  | 208894_at | TRUE |
| 208916_at | TRUE |  | 208916_at | TRUE |
| 208918_s_at | TRUE |  | 208918_s_at | TRUE |
| 208919_s_at | TRUE |  | 208919_s_at | TRUE |
| 208923_at | TRUE |  | 208923_at | TRUE |
| 208961_s_at | TRUE |  | 208961_s_at | TRUE |
| 208965_s_at | TRUE |  | 208965_s_at | TRUE |
| 208966_x_at | TRUE |  | 208966_x_at | TRUE |
| 208967_s_at | TRUE |  | 208967_s_at | TRUE |
| 208970_s_at | TRUE |  | 208970_s_at | TRUE |
| 208971_at | TRUE |  | 208971_at | TRUE |
| 208997_s_at | TRUE |  | 208997_s_at | TRUE |
| 208998_at | TRUE |  | 208998_at | TRUE |
| 208999_at | TRUE |  | 208999_at | TRUE |
| 209010_s_at | TRUE |  | 209010_s_at | TRUE |
| 209015_s_at | TRUE |  | 209015_s_at | TRUE |
| 209083_at | TRUE |  | 209083_at | TRUE |
| 209090_s_at | TRUE |  | 209090_s_at | TRUE |
| 209091_s_at | TRUE |  | 209091_s_at | TRUE |
| 209129_at | TRUE |  | 209129_at | TRUE |
| 209130_at | TRUE |  | 209130_at | TRUE |
| 209146_at | TRUE |  | 209146_at | TRUE |
| 209159_s_at | TRUE |  | 209159_s_at | TRUE |
| 209166_s_at | TRUE |  | 209166_s_at | TRUE |
| 209187_at | TRUE |  | 209187_at | TRUE |
| 209191_at | TRUE |  | 209191_at | TRUE |
| 209206_at | TRUE |  | 209206_at | TRUE |
| 209207_s_at | TRUE |  | 209207_s_at | TRUE |
| 209269_s_at | TRUE |  | 209269_s_at | TRUE |
| 209279_s_at | TRUE |  | 209279_s_at | TRUE |
| 209282_at | TRUE |  | 209282_at | TRUE |
| 209312_x_at | TRUE |  | 209312_x_at | TRUE |
| 209347_s_at | TRUE |  | 209347_s_at | TRUE |
| 209348_s_at | TRUE |  | 209348_s_at | TRUE |
| 209349_at | TRUE |  | 209349_at | TRUE |
| 209355_s_at | TRUE |  | 209355_s_at | TRUE |
| 209360_s_at | TRUE |  | 209360_s_at | TRUE |
| 209367_at | TRUE |  | 209367_at | TRUE |
| 209459_s_at | TRUE |  | 209459_s_at | TRUE |
| 209460_at | TRUE |  | 209460_at | TRUE |
| 209467_s_at | TRUE |  | 209467_s_at | TRUE |
| 209469_at | TRUE |  | 209469_at | TRUE |
| 209473_at | TRUE |  | 209473_at | TRUE |
| 209474_s_at | TRUE |  | 209474_s_at | TRUE |
| 209534_x_at | TRUE |  | 209534_x_at | TRUE |
| 209569_x_at | TRUE |  | 209569_x_at | TRUE |
| 209570_s_at | TRUE |  | 209570_s_at | TRUE |
| 209608_s_at | TRUE |  | 209608_s_at | TRUE |
| 209619_at | TRUE |  | 209619_at | TRUE |
| 209669_s_at | TRUE |  | 209669_s_at | TRUE |
| 209681_at | TRUE |  | 209681_at | TRUE |
| 209696_at | TRUE |  | 209696_at | TRUE |
| 209707_at | TRUE |  | 209707_at | TRUE |
| 209709_s_at | TRUE |  | 209709_s_at | TRUE |
| 209711_at | TRUE |  | 209711_at | TRUE |
| 209712_at | TRUE |  | 209712_at | TRUE |
| 209716_at | TRUE |  | 209716_at | TRUE |
| 209728_at | TRUE |  | 209728_at | TRUE |
| 209734_at | TRUE |  | 209734_at | TRUE |
| 209755_at | TRUE |  | 209755_at | TRUE |
| 209768_s_at | TRUE |  | 209768_s_at | TRUE |
| 209806_at | TRUE |  | 209806_at | TRUE |
| 209823_x_at | TRUE |  | 209823_x_at | TRUE |
| 209827_s_at | TRUE |  | 209827_s_at | TRUE |
| 209835_x_at | TRUE |  | 209835_x_at | TRUE |
| 209868_s_at | TRUE |  | 209868_s_at | TRUE |
| 209879_at | TRUE |  | 209879_at | TRUE |
| 209891_at | TRUE |  | 209891_at | TRUE |
| 209893_s_at | TRUE |  | 209893_s_at | TRUE |
| 209901_x_at | TRUE |  | 209901_x_at | TRUE |
| 209906_at | TRUE |  | 209906_at | TRUE |
| 209912_s_at | TRUE |  | 209912_s_at | TRUE |
| 209914_s_at | TRUE |  | 209914_s_at | TRUE |
| 209915_s_at | TRUE |  | 209915_s_at | TRUE |
| 209933_s_at | TRUE |  | 209933_s_at | TRUE |
| 209940_at | TRUE |  | 209940_at | TRUE |
| 209966_x_at | TRUE |  | 209966_x_at | TRUE |
| 209970_x_at | TRUE |  | 209970_x_at | TRUE |
| 209980_s_at | TRUE |  | 209980_s_at | TRUE |
| 209987_s_at | TRUE |  | 209987_s_at | TRUE |
| 210012_s_at | TRUE |  | 210012_s_at | TRUE |
| 210015_s_at | TRUE |  | 210015_s_at | TRUE |
| 210016_at | TRUE |  | 210016_at | TRUE |
| 210018_x_at | TRUE |  | 210018_x_at | TRUE |
| 210040_at | TRUE |  | 210040_at | TRUE |
| 210042_s_at | TRUE |  | 210042_s_at | TRUE |
| 210044_s_at | TRUE |  | 210044_s_at | TRUE |
| 210077_s_at | TRUE |  | 210077_s_at | TRUE |
| 210093_s_at | TRUE |  | 210093_s_at | TRUE |
| 210101_x_at | TRUE |  | 210101_x_at | TRUE |
| 210106_at | TRUE |  | 210106_at | TRUE |
| 210113_s_at | TRUE |  | 210113_s_at | TRUE |
| 210114_at | TRUE |  | 210114_at | TRUE |
| 210123_s_at | TRUE |  | 210123_s_at | TRUE |
| 210145_at | TRUE |  | 210145_at | TRUE |
| 210152_at | TRUE |  | 210152_at | TRUE |
| 210162_s_at | TRUE |  | 210162_s_at | TRUE |
| 210166_at | TRUE |  | 210166_at | TRUE |
| 210176_at | TRUE |  | 210176_at | TRUE |
| 210181_s_at | TRUE |  | 210181_s_at | TRUE |
| 210184_at | TRUE |  | 210184_at | TRUE |
| 210192_at | TRUE |  | 210192_at | TRUE |
| 210222_s_at | TRUE |  | 210222_s_at | TRUE |
| 210241_s_at | TRUE |  | 210241_s_at | TRUE |
| 210246_s_at | TRUE |  | 210246_s_at | TRUE |
| 210252_s_at | TRUE |  | 210252_s_at | TRUE |
| 210287_s_at | TRUE |  | 210287_s_at | TRUE |
| 210319_x_at | TRUE |  | 210319_x_at | TRUE |
| 210323_at | TRUE |  | 210323_at | TRUE |
| 210340_s_at | TRUE |  | 210340_s_at | TRUE |
| 210349_at | TRUE |  | 210349_at | TRUE |
| 210427_x_at | TRUE |  | 210427_x_at | TRUE |
| 210436_at | TRUE |  | 210436_at | TRUE |
| 210466_s_at | TRUE |  | 210466_s_at | TRUE |
| 210471_s_at | TRUE |  | 210471_s_at | TRUE |
| 210474_s_at | TRUE |  | 210474_s_at | TRUE |
| 210560_at | TRUE |  | 210560_at | TRUE |
| 210582_s_at | TRUE |  | 210582_s_at | TRUE |
| 210593_at | TRUE |  | 210593_at | TRUE |
| 210607_at | TRUE |  | 210607_at | TRUE |
| 210629_x_at | TRUE |  | 210629_x_at | TRUE |
| 210644_s_at | TRUE |  | 210644_s_at | TRUE |
| 210645_s_at | TRUE |  | 210645_s_at | TRUE |
| 210649_s_at | TRUE |  | 210649_s_at | TRUE |
| 210655_s_at | TRUE |  | 210655_s_at | TRUE |
| 210660_at | TRUE |  | 210660_at | TRUE |
| 210672_s_at | TRUE |  | 210672_s_at | TRUE |
| 210697_at | TRUE |  | 210697_at | TRUE |
| 210735_s_at | TRUE |  | 210735_s_at | TRUE |
| 210743_s_at | TRUE |  | 210743_s_at | TRUE |
| 210754_s_at | TRUE |  | 210754_s_at | TRUE |
| 210814_at | TRUE |  | 210814_at | TRUE |
| 210840_s_at | TRUE |  | 210840_s_at | TRUE |
| 210886_x_at | TRUE |  | 210886_x_at | TRUE |
| 210895_s_at | TRUE |  | 210895_s_at | TRUE |
| 210912_x_at | TRUE |  | 210912_x_at | TRUE |
| 210959_s_at | TRUE |  | 210959_s_at | TRUE |
| 210982_s_at | TRUE |  | 210982_s_at | TRUE |
| 211004_s_at | TRUE |  | 211004_s_at | TRUE |
| 211020_at | TRUE |  | 211020_at | TRUE |
| 211022_s_at | TRUE |  | 211022_s_at | TRUE |
| 211056_s_at | TRUE |  | 211056_s_at | TRUE |
| 211100_x_at | TRUE |  | 211100_x_at | TRUE |
| 211101_x_at | TRUE |  | 211101_x_at | TRUE |
| 211102_s_at | TRUE |  | 211102_s_at | TRUE |
| 211105_s_at | TRUE |  | 211105_s_at | TRUE |
| 211110_s_at | TRUE |  | 211110_s_at | TRUE |
| 211124_s_at | TRUE |  | 211124_s_at | TRUE |
| 211126_s_at | TRUE |  | 211126_s_at | TRUE |
| 211180_x_at | TRUE |  | 211180_x_at | TRUE |
| 211190_x_at | TRUE |  | 211190_x_at | TRUE |
| 211192_s_at | TRUE |  | 211192_s_at | TRUE |
| 211207_s_at | TRUE |  | 211207_s_at | TRUE |
| 211276_at | TRUE |  | 211276_at | TRUE |
| 211286_x_at | TRUE |  | 211286_x_at | TRUE |
| 211289_x_at | TRUE |  | 211289_x_at | TRUE |
| 211336_x_at | TRUE |  | 211336_x_at | TRUE |
| 211366_x_at | TRUE |  | 211366_x_at | TRUE |
| 211367_s_at | TRUE |  | 211367_s_at | TRUE |
| 211368_s_at | TRUE |  | 211368_s_at | TRUE |
| 211423_s_at | TRUE |  | 211423_s_at | TRUE |
| 211429_s_at | TRUE |  | 211429_s_at | TRUE |
| 211507_s_at | TRUE |  | 211507_s_at | TRUE |
| 211534_x_at | TRUE |  | 211534_x_at | TRUE |
| 211552_s_at | TRUE |  | 211552_s_at | TRUE |
| 211559_s_at | TRUE |  | 211559_s_at | TRUE |
| 211568_at | TRUE |  | 211568_at | TRUE |
| 211581_x_at | TRUE |  | 211581_x_at | TRUE |
| 211582_x_at | TRUE |  | 211582_x_at | TRUE |
| 211586_s_at | TRUE |  | 211586_s_at | TRUE |
| 211602_s_at | TRUE |  | 211602_s_at | TRUE |
| 211612_s_at | TRUE |  | 211612_s_at | TRUE |
| 211615_s_at | TRUE |  | 211615_s_at | TRUE |
| 211616_s_at | TRUE |  | 211616_s_at | TRUE |
| 211621_at | TRUE |  | 211621_at | TRUE |
| 211656_x_at | TRUE |  | 211656_x_at | TRUE |
| 211661_x_at | TRUE |  | 211661_x_at | TRUE |
| 211725_s_at | TRUE |  | 211725_s_at | TRUE |
| 211733_x_at | TRUE |  | 211733_x_at | TRUE |
| 211742_s_at | TRUE |  | 211742_s_at | TRUE |
| 211744_s_at | TRUE |  | 211744_s_at | TRUE |
| 211759_x_at | TRUE |  | 211759_x_at | TRUE |
| 211794_at | TRUE |  | 211794_at | TRUE |
| 211795_s_at | TRUE |  | 211795_s_at | TRUE |
| 211876_x_at | TRUE |  | 211876_x_at | TRUE |
| 211894_x_at | TRUE |  | 211894_x_at | TRUE |
| 211905_s_at | TRUE |  | 211905_s_at | TRUE |
| 211930_at | TRUE |  | 211930_at | TRUE |
| 211950_at | TRUE |  | 211950_at | TRUE |
| 211958_at | TRUE |  | 211958_at | TRUE |
| 211959_at | TRUE |  | 211959_at | TRUE |
| 211962_s_at | TRUE |  | 211962_s_at | TRUE |
| 211971_s_at | TRUE |  | 211971_s_at | TRUE |
| 211990_at | TRUE |  | 211990_at | TRUE |
| 211991_s_at | TRUE |  | 211991_s_at | TRUE |
| 212005_at | TRUE |  | 212005_at | TRUE |
| 212014_x_at | TRUE |  | 212014_x_at | TRUE |
| 212024_x_at | TRUE |  | 212024_x_at | TRUE |
| 212049_at | TRUE |  | 212049_at | TRUE |
| 212062_at | TRUE |  | 212062_at | TRUE |
| 212063_at | TRUE |  | 212063_at | TRUE |
| 212065_s_at | TRUE |  | 212065_s_at | TRUE |
| 212099_at | TRUE |  | 212099_at | TRUE |
| 212101_at | TRUE |  | 212101_at | TRUE |
| 212103_at | TRUE |  | 212103_at | TRUE |
| 212111_at | TRUE |  | 212111_at | TRUE |
| 212112_s_at | TRUE |  | 212112_s_at | TRUE |
| 212113_at | TRUE |  | 212113_at | TRUE |
| 212119_at | TRUE |  | 212119_at | TRUE |
| 212120_at | TRUE |  | 212120_at | TRUE |
| 212122_at | TRUE |  | 212122_at | TRUE |
| 212131_at | TRUE |  | 212131_at | TRUE |
| 212132_at | TRUE |  | 212132_at | TRUE |
| 212152_x_at | TRUE |  | 212152_x_at | TRUE |
| 212173_at | TRUE |  | 212173_at | TRUE |
| 212174_at | TRUE |  | 212174_at | TRUE |
| 212175_s_at | TRUE |  | 212175_s_at | TRUE |
| 212184_s_at | TRUE |  | 212184_s_at | TRUE |
| 212186_at | TRUE |  | 212186_at | TRUE |
| 212203_x_at | TRUE |  | 212203_x_at | TRUE |
| 212214_at | TRUE |  | 212214_at | TRUE |
| 212226_s_at | TRUE |  | 212226_s_at | TRUE |
| 212230_at | TRUE |  | 212230_at | TRUE |
| 212239_at | TRUE |  | 212239_at | TRUE |
| 212279_at | TRUE |  | 212279_at | TRUE |
| 212281_s_at | TRUE |  | 212281_s_at | TRUE |
| 212282_at | TRUE |  | 212282_at | TRUE |
| 212291_at | TRUE |  | 212291_at | TRUE |
| 212293_at | TRUE |  | 212293_at | TRUE |
| 212294_at | TRUE |  | 212294_at | TRUE |
| 212300_at | TRUE |  | 212300_at | TRUE |
| 212308_at | TRUE |  | 212308_at | TRUE |
| 212337_at | TRUE |  | 212337_at | TRUE |
| 212345_s_at | TRUE |  | 212345_s_at | TRUE |
| 212377_s_at | TRUE |  | 212377_s_at | TRUE |
| 212383_at | TRUE |  | 212383_at | TRUE |
| 212385_at | TRUE |  | 212385_at | TRUE |
| 212388_at | TRUE |  | 212388_at | TRUE |
| 212394_at | TRUE |  | 212394_at | TRUE |
| 212395_s_at | TRUE |  | 212395_s_at | TRUE |
| 212401_s_at | TRUE |  | 212401_s_at | TRUE |
| 212415_at | TRUE |  | 212415_at | TRUE |
| 212482_at | TRUE |  | 212482_at | TRUE |
| 212491_s_at | TRUE |  | 212491_s_at | TRUE |
| 212510_at | TRUE |  | 212510_at | TRUE |
| 212522_at | TRUE |  | 212522_at | TRUE |
| 212577_at | TRUE |  | 212577_at | TRUE |
| 212587_s_at | TRUE |  | 212587_s_at | TRUE |
| 212588_at | TRUE |  | 212588_at | TRUE |
| 212607_at | TRUE |  | 212607_at | TRUE |
| 212615_at | TRUE |  | 212615_at | TRUE |
| 212616_at | TRUE |  | 212616_at | TRUE |
| 212624_s_at | TRUE |  | 212624_s_at | TRUE |
| 212628_at | TRUE |  | 212628_at | TRUE |
| 212629_s_at | TRUE |  | 212629_s_at | TRUE |
| 212641_at | TRUE |  | 212641_at | TRUE |
| 212642_s_at | TRUE |  | 212642_s_at | TRUE |
| 212658_at | TRUE |  | 212658_at | TRUE |
| 212663_at | TRUE |  | 212663_at | TRUE |
| 212671_s_at | TRUE |  | 212671_s_at | TRUE |
| 212699_at | TRUE |  | 212699_at | TRUE |
| 212704_at | TRUE |  | 212704_at | TRUE |
| 212725_s_at | TRUE |  | 212725_s_at | TRUE |
| 212730_at | TRUE |  | 212730_at | TRUE |
| 212743_at | TRUE |  | 212743_at | TRUE |
| 212744_at | TRUE |  | 212744_at | TRUE |
| 212765_at | TRUE |  | 212765_at | TRUE |
| 212768_s_at | TRUE |  | 212768_s_at | TRUE |
| 212774_at | TRUE |  | 212774_at | TRUE |
| 212788_x_at | TRUE |  | 212788_x_at | TRUE |
| 212816_s_at | TRUE |  | 212816_s_at | TRUE |
| 212820_at | TRUE |  | 212820_at | TRUE |
| 212873_at | TRUE |  | 212873_at | TRUE |
| 212875_s_at | TRUE |  | 212875_s_at | TRUE |
| 212893_at | TRUE |  | 212893_at | TRUE |
| 212906_at | TRUE |  | 212906_at | TRUE |
| 212920_at | TRUE |  | 212920_at | TRUE |
| 212922_s_at | TRUE |  | 212922_s_at | TRUE |
| 212944_at | TRUE |  | 212944_at | TRUE |
| 212945_s_at | TRUE |  | 212945_s_at | TRUE |
| 212958_x_at | TRUE |  | 212958_x_at | TRUE |
| 212969_x_at | TRUE |  | 212969_x_at | TRUE |
| 212998_x_at | TRUE |  | 212998_x_at | TRUE |
| 213038_at | TRUE |  | 213038_at | TRUE |
| 213052_at | TRUE |  | 213052_at | TRUE |
| 213053_at | TRUE |  | 213053_at | TRUE |
| 213076_at | TRUE |  | 213076_at | TRUE |
| 213095_x_at | TRUE |  | 213095_x_at | TRUE |
| 213111_at | TRUE |  | 213111_at | TRUE |
| 213114_at | TRUE |  | 213114_at | TRUE |
| 213125_at | TRUE |  | 213125_at | TRUE |
| 213131_at | TRUE |  | 213131_at | TRUE |
| 213160_at | TRUE |  | 213160_at | TRUE |
| 213170_at | TRUE |  | 213170_at | TRUE |
| 213171_s_at | TRUE |  | 213171_s_at | TRUE |
| 213186_at | TRUE |  | 213186_at | TRUE |
| 213187_x_at | TRUE |  | 213187_x_at | TRUE |
| 213227_at | TRUE |  | 213227_at | TRUE |
| 213231_at | TRUE |  | 213231_at | TRUE |
| 213278_at | TRUE |  | 213278_at | TRUE |
| 213280_at | TRUE |  | 213280_at | TRUE |
| 213281_at | TRUE |  | 213281_at | TRUE |
| 213296_at | TRUE |  | 213296_at | TRUE |
| 213307_at | TRUE |  | 213307_at | TRUE |
| 213309_at | TRUE |  | 213309_at | TRUE |
| 213338_at | TRUE |  | 213338_at | TRUE |
| 213361_at | TRUE |  | 213361_at | TRUE |
| 213369_at | TRUE |  | 213369_at | TRUE |
| 213373_s_at | TRUE |  | 213373_s_at | TRUE |
| 213397_x_at | TRUE |  | 213397_x_at | TRUE |
| 213405_at | TRUE |  | 213405_at | TRUE |
| 213407_at | TRUE |  | 213407_at | TRUE |
| 213411_at | TRUE |  | 213411_at | TRUE |
| 213415_at | TRUE |  | 213415_at | TRUE |
| 213425_at | TRUE |  | 213425_at | TRUE |
| 213439_x_at | TRUE |  | 213439_x_at | TRUE |
| 213454_at | TRUE |  | 213454_at | TRUE |
| 213484_at | TRUE |  | 213484_at | TRUE |
| 213497_at | TRUE |  | 213497_at | TRUE |
| 213503_x_at | TRUE |  | 213503_x_at | TRUE |
| 213506_at | TRUE |  | 213506_at | TRUE |
| 213521_at | TRUE |  | 213521_at | TRUE |
| 213526_s_at | TRUE |  | 213526_s_at | TRUE |
| 213533_at | TRUE |  | 213533_at | TRUE |
| 213549_at | TRUE |  | 213549_at | TRUE |
| 213558_at | TRUE |  | 213558_at | TRUE |
| 213566_at | TRUE |  | 213566_at | TRUE |
| 213572_s_at | TRUE |  | 213572_s_at | TRUE |
| 213603_s_at | TRUE |  | 213603_s_at | TRUE |
| 213604_at | TRUE |  | 213604_at | TRUE |
| 213607_x_at | TRUE |  | 213607_x_at | TRUE |
| 213609_s_at | TRUE |  | 213609_s_at | TRUE |
| 213628_at | TRUE |  | 213628_at | TRUE |
| 213636_at | TRUE |  | 213636_at | TRUE |
| 213638_at | TRUE |  | 213638_at | TRUE |
| 213676_at | TRUE |  | 213676_at | TRUE |
| 213678_at | TRUE |  | 213678_at | TRUE |
| 213683_at | TRUE |  | 213683_at | TRUE |
| 213686_at | TRUE |  | 213686_at | TRUE |
| 213694_at | TRUE |  | 213694_at | TRUE |
| 213695_at | TRUE |  | 213695_at | TRUE |
| 213698_at | TRUE |  | 213698_at | TRUE |
| 213716_s_at | TRUE |  | 213716_s_at | TRUE |
| 213733_at | TRUE |  | 213733_at | TRUE |
| 213742_at | TRUE |  | 213742_at | TRUE |
| 213746_s_at | TRUE |  | 213746_s_at | TRUE |
| 213798_s_at | TRUE |  | 213798_s_at | TRUE |
| 213804_at | TRUE |  | 213804_at | TRUE |
| 213834_at | TRUE |  | 213834_at | TRUE |
| 213845_at | TRUE |  | 213845_at | TRUE |
| 213856_at | TRUE |  | 213856_at | TRUE |
| 213883_s_at | TRUE |  | 213883_s_at | TRUE |
| 213888_s_at | TRUE |  | 213888_s_at | TRUE |
| 213895_at | TRUE |  | 213895_at | TRUE |
| 213920_at | TRUE |  | 213920_at | TRUE |
| 213927_at | TRUE |  | 213927_at | TRUE |
| 213982_s_at | TRUE |  | 213982_s_at | TRUE |
| 213990_s_at | TRUE |  | 213990_s_at | TRUE |
| 214000_s_at | TRUE |  | 214000_s_at | TRUE |
| 214020_x_at | TRUE |  | 214020_x_at | TRUE |
| 214021_x_at | TRUE |  | 214021_x_at | TRUE |
| 214079_at | TRUE |  | 214079_at | TRUE |
| 214084_x_at | TRUE |  | 214084_x_at | TRUE |
| 214098_at | TRUE |  | 214098_at | TRUE |
| 214104_at | TRUE |  | 214104_at | TRUE |
| 214120_at | TRUE |  | 214120_at | TRUE |
| 214128_at | TRUE |  | 214128_at | TRUE |
| 214179_s_at | TRUE |  | 214179_s_at | TRUE |
| 214180_at | TRUE |  | 214180_at | TRUE |
| 214181_x_at | TRUE |  | 214181_x_at | TRUE |
| 214194_at | TRUE |  | 214194_at | TRUE |
| 214257_s_at | TRUE |  | 214257_s_at | TRUE |
| 214262_at | TRUE |  | 214262_at | TRUE |
| 214274_s_at | TRUE |  | 214274_s_at | TRUE |
| 214290_s_at | TRUE |  | 214290_s_at | TRUE |
| 214292_at | TRUE |  | 214292_at | TRUE |
| 214329_x_at | TRUE |  | 214329_x_at | TRUE |
| 214364_at | TRUE |  | 214364_at | TRUE |
| 214366_s_at | TRUE |  | 214366_s_at | TRUE |
| 214376_at | TRUE |  | 214376_at | TRUE |
| 214383_x_at | TRUE |  | 214383_x_at | TRUE |
| 214408_s_at | TRUE |  | 214408_s_at | TRUE |
| 214428_x_at | TRUE |  | 214428_x_at | TRUE |
| 214438_at | TRUE |  | 214438_at | TRUE |
| 214448_x_at | TRUE |  | 214448_x_at | TRUE |
| 214495_at | TRUE |  | 214495_at | TRUE |
| 214511_x_at | TRUE |  | 214511_x_at | TRUE |
| 214512_s_at | TRUE |  | 214512_s_at | TRUE |
| 214514_at | TRUE |  | 214514_at | TRUE |
| 214574_x_at | TRUE |  | 214574_x_at | TRUE |
| 214597_at | TRUE |  | 214597_at | TRUE |
| 214620_x_at | TRUE |  | 214620_x_at | TRUE |
| 214661_s_at | TRUE |  | 214661_s_at | TRUE |
| 214752_x_at | TRUE |  | 214752_x_at | TRUE |
| 214770_at | TRUE |  | 214770_at | TRUE |
| 214778_at | TRUE |  | 214778_at | TRUE |
| 214791_at | TRUE |  | 214791_at | TRUE |
| 214807_at | TRUE |  | 214807_at | TRUE |
| 214813_at | TRUE |  | 214813_at | TRUE |
| 214825_at | TRUE |  | 214825_at | TRUE |
| 214839_at | TRUE |  | 214839_at | TRUE |
| 214840_at | TRUE |  | 214840_at | TRUE |
| 214852_x_at | TRUE |  | 214852_x_at | TRUE |
| 214857_at | TRUE |  | 214857_at | TRUE |
| 214879_x_at | TRUE |  | 214879_x_at | TRUE |
| 214884_at | TRUE |  | 214884_at | TRUE |
| 214894_x_at | TRUE |  | 214894_x_at | TRUE |
| 214909_s_at | TRUE |  | 214909_s_at | TRUE |
| 214929_s_at | TRUE |  | 214929_s_at | TRUE |
| 214939_x_at | TRUE |  | 214939_x_at | TRUE |
| 214945_at | TRUE |  | 214945_at | TRUE |
| 214949_at | TRUE |  | 214949_at | TRUE |
| 214956_at | TRUE |  | 214956_at | TRUE |
| 214972_at | TRUE |  | 214972_at | TRUE |
| 214993_at | TRUE |  | 214993_at | TRUE |
| 215017_s_at | TRUE |  | 215017_s_at | TRUE |
| 215045_at | TRUE |  | 215045_at | TRUE |
| 215046_at | TRUE |  | 215046_at | TRUE |
| 215051_x_at | TRUE |  | 215051_x_at | TRUE |
| 215064_at | TRUE |  | 215064_at | TRUE |
| 215071_s_at | TRUE |  | 215071_s_at | TRUE |
| 215084_s_at | TRUE |  | 215084_s_at | TRUE |
| 215087_at | TRUE |  | 215087_at | TRUE |
| 215127_s_at | TRUE |  | 215127_s_at | TRUE |
| 215193_x_at | TRUE |  | 215193_x_at | TRUE |
| 215222_x_at | TRUE |  | 215222_x_at | TRUE |
| 215227_x_at | TRUE |  | 215227_x_at | TRUE |
| 215252_at | TRUE |  | 215252_at | TRUE |
| 215262_at | TRUE |  | 215262_at | TRUE |
| 215267_s_at | TRUE |  | 215267_s_at | TRUE |
| 215285_s_at | TRUE |  | 215285_s_at | TRUE |
| 215304_at | TRUE |  | 215304_at | TRUE |
| 215364_s_at | TRUE |  | 215364_s_at | TRUE |
| 215389_s_at | TRUE |  | 215389_s_at | TRUE |
| 215396_at | TRUE |  | 215396_at | TRUE |
| 215419_at | TRUE |  | 215419_at | TRUE |
| 215423_at | TRUE |  | 215423_at | TRUE |
| 215448_at | TRUE |  | 215448_at | TRUE |
| 215499_at | TRUE |  | 215499_at | TRUE |
| 215518_at | TRUE |  | 215518_at | TRUE |
| 215532_x_at | TRUE |  | 215532_x_at | TRUE |
| 215537_x_at | TRUE |  | 215537_x_at | TRUE |
| 215566_x_at | TRUE |  | 215566_x_at | TRUE |
| 215617_at | TRUE |  | 215617_at | TRUE |
| 215633_x_at | TRUE |  | 215633_x_at | TRUE |
| 215691_x_at | TRUE |  | 215691_x_at | TRUE |
| 215736_at | TRUE |  | 215736_at | TRUE |
| 215737_x_at | TRUE |  | 215737_x_at | TRUE |
| 215758_x_at | TRUE |  | 215758_x_at | TRUE |
| 215783_s_at | TRUE |  | 215783_s_at | TRUE |
| 215813_s_at | TRUE |  | 215813_s_at | TRUE |
| 215854_at | TRUE |  | 215854_at | TRUE |
| 215933_s_at | TRUE |  | 215933_s_at | TRUE |
| 215972_at | TRUE |  | 215972_at | TRUE |
| 216047_x_at | TRUE |  | 216047_x_at | TRUE |
| 216061_x_at | TRUE |  | 216061_x_at | TRUE |
| 216073_at | TRUE |  | 216073_at | TRUE |
| 216096_s_at | TRUE |  | 216096_s_at | TRUE |
| 216139_s_at | TRUE |  | 216139_s_at | TRUE |
| 216167_at | TRUE |  | 216167_at | TRUE |
| 216184_s_at | TRUE |  | 216184_s_at | TRUE |
| 216194_s_at | TRUE |  | 216194_s_at | TRUE |
| 216218_s_at | TRUE |  | 216218_s_at | TRUE |
| 216221_s_at | TRUE |  | 216221_s_at | TRUE |
| 216224_s_at | TRUE |  | 216224_s_at | TRUE |
| 216235_s_at | TRUE |  | 216235_s_at | TRUE |
| 216250_s_at | TRUE |  | 216250_s_at | TRUE |
| 216255_s_at | TRUE |  | 216255_s_at | TRUE |
| 216259_at | TRUE |  | 216259_at | TRUE |
| 216264_s_at | TRUE |  | 216264_s_at | TRUE |
| 216347_s_at | TRUE |  | 216347_s_at | TRUE |
| 216352_x_at | TRUE |  | 216352_x_at | TRUE |
| 216384_x_at | TRUE |  | 216384_x_at | TRUE |
| 216410_at | TRUE |  | 216410_at | TRUE |
| 216438_s_at | TRUE |  | 216438_s_at | TRUE |
| 216457_s_at | TRUE |  | 216457_s_at | TRUE |
| 216526_x_at | TRUE |  | 216526_x_at | TRUE |
| 216598_s_at | TRUE |  | 216598_s_at | TRUE |
| 216652_s_at | TRUE |  | 216652_s_at | TRUE |
| 216672_s_at | TRUE |  | 216672_s_at | TRUE |
| 216695_s_at | TRUE |  | 216695_s_at | TRUE |
| 216705_s_at | TRUE |  | 216705_s_at | TRUE |
| 216850_at | TRUE |  | 216850_at | TRUE |
| 216908_x_at | TRUE |  | 216908_x_at | TRUE |
| 216933_x_at | TRUE |  | 216933_x_at | TRUE |
| 216942_s_at | TRUE |  | 216942_s_at | TRUE |
| 216950_s_at | TRUE |  | 216950_s_at | TRUE |
| 217004_s_at | TRUE |  | 217004_s_at | TRUE |
| 217066_s_at | TRUE |  | 217066_s_at | TRUE |
| 217078_s_at | TRUE |  | 217078_s_at | TRUE |
| 217130_at | TRUE |  | 217130_at | TRUE |
| 217173_s_at | TRUE |  | 217173_s_at | TRUE |
| 217196_s_at | TRUE |  | 217196_s_at | TRUE |
| 217208_s_at | TRUE |  | 217208_s_at | TRUE |
| 217362_x_at | TRUE |  | 217362_x_at | TRUE |
| 217455_s_at | TRUE |  | 217455_s_at | TRUE |
| 217456_x_at | TRUE |  | 217456_x_at | TRUE |
| 217475_s_at | TRUE |  | 217475_s_at | TRUE |
| 217478_s_at | TRUE |  | 217478_s_at | TRUE |
| 217513_at | TRUE |  | 217513_at | TRUE |
| 217572_at | TRUE |  | 217572_at | TRUE |
| 217615_at | TRUE |  | 217615_at | TRUE |
| 217722_s_at | TRUE |  | 217722_s_at | TRUE |
| 217728_at | TRUE |  | 217728_at | TRUE |
| 217741_s_at | TRUE |  | 217741_s_at | TRUE |
| 217757_at | TRUE |  | 217757_at | TRUE |
| 217766_s_at | TRUE |  | 217766_s_at | TRUE |
| 217767_at | TRUE |  | 217767_at | TRUE |
| 217778_at | TRUE |  | 217778_at | TRUE |
| 217779_s_at | TRUE |  | 217779_s_at | TRUE |
| 217784_at | TRUE |  | 217784_at | TRUE |
| 217812_at | TRUE |  | 217812_at | TRUE |
| 217844_at | TRUE |  | 217844_at | TRUE |
| 217855_x_at | TRUE |  | 217855_x_at | TRUE |
| 217865_at | TRUE |  | 217865_at | TRUE |
| 217877_s_at | TRUE |  | 217877_s_at | TRUE |
| 217893_s_at | TRUE |  | 217893_s_at | TRUE |
| 217911_s_at | TRUE |  | 217911_s_at | TRUE |
| 217923_at | TRUE |  | 217923_at | TRUE |
| 217929_s_at | TRUE |  | 217929_s_at | TRUE |
| 217933_s_at | TRUE |  | 217933_s_at | TRUE |
| 217944_at | TRUE |  | 217944_at | TRUE |
| 217947_at | TRUE |  | 217947_at | TRUE |
| 217950_at | TRUE |  | 217950_at | TRUE |
| 217966_s_at | TRUE |  | 217966_s_at | TRUE |
| 217967_s_at | TRUE |  | 217967_s_at | TRUE |
| 217979_at | TRUE |  | 217979_at | TRUE |
| 217983_s_at | TRUE |  | 217983_s_at | TRUE |
| 217984_at | TRUE |  | 217984_at | TRUE |
| 217994_x_at | TRUE |  | 217994_x_at | TRUE |
| 218022_at | TRUE |  | 218022_at | TRUE |
| 218035_s_at | TRUE |  | 218035_s_at | TRUE |
| 218037_at | TRUE |  | 218037_at | TRUE |
| 218040_at | TRUE |  | 218040_at | TRUE |
| 218048_at | TRUE |  | 218048_at | TRUE |
| 218080_x_at | TRUE |  | 218080_x_at | TRUE |
| 218087_s_at | TRUE |  | 218087_s_at | TRUE |
| 218088_s_at | TRUE |  | 218088_s_at | TRUE |
| 218116_at | TRUE |  | 218116_at | TRUE |
| 218162_at | TRUE |  | 218162_at | TRUE |
| 218194_at | TRUE |  | 218194_at | TRUE |
| 218197_s_at | TRUE |  | 218197_s_at | TRUE |
| 218204_s_at | TRUE |  | 218204_s_at | TRUE |
| 218217_at | TRUE |  | 218217_at | TRUE |
| 218232_at | TRUE |  | 218232_at | TRUE |
| 218237_s_at | TRUE |  | 218237_s_at | TRUE |
| 218280_x_at | TRUE |  | 218280_x_at | TRUE |
| 218284_at | TRUE |  | 218284_at | TRUE |
| 218299_at | TRUE |  | 218299_at | TRUE |
| 218302_at | TRUE |  | 218302_at | TRUE |
| 218306_s_at | TRUE |  | 218306_s_at | TRUE |
| 218318_s_at | TRUE |  | 218318_s_at | TRUE |
| 218332_at | TRUE |  | 218332_at | TRUE |
| 218341_at | TRUE |  | 218341_at | TRUE |
| 218344_s_at | TRUE |  | 218344_s_at | TRUE |
| 218362_s_at | TRUE |  | 218362_s_at | TRUE |
| 218370_s_at | TRUE |  | 218370_s_at | TRUE |
| 218409_s_at | TRUE |  | 218409_s_at | TRUE |
| 218418_s_at | TRUE |  | 218418_s_at | TRUE |
| 218433_at | TRUE |  | 218433_at | TRUE |
| 218442_at | TRUE |  | 218442_at | TRUE |
| 218450_at | TRUE |  | 218450_at | TRUE |
| 218454_at | TRUE |  | 218454_at | TRUE |
| 218458_at | TRUE |  | 218458_at | TRUE |
| 218462_at | TRUE |  | 218462_at | TRUE |
| 218530_at | TRUE |  | 218530_at | TRUE |
| 218551_at | TRUE |  | 218551_at | TRUE |
| 218574_s_at | TRUE |  | 218574_s_at | TRUE |
| 218583_s_at | TRUE |  | 218583_s_at | TRUE |
| 218589_at | TRUE |  | 218589_at | TRUE |
| 218623_at | TRUE |  | 218623_at | TRUE |
| 218632_at | TRUE |  | 218632_at | TRUE |
| 218684_at | TRUE |  | 218684_at | TRUE |
| 218711_s_at | TRUE |  | 218711_s_at | TRUE |
| 218712_at | TRUE |  | 218712_at | TRUE |
| 218717_s_at | TRUE |  | 218717_s_at | TRUE |
| 218720_x_at | TRUE |  | 218720_x_at | TRUE |
| 218727_at | TRUE |  | 218727_at | TRUE |
| 218736_s_at | TRUE |  | 218736_s_at | TRUE |
| 218751_s_at | TRUE |  | 218751_s_at | TRUE |
| 218766_s_at | TRUE |  | 218766_s_at | TRUE |
| 218770_s_at | TRUE |  | 218770_s_at | TRUE |
| 218831_s_at | TRUE |  | 218831_s_at | TRUE |
| 218839_at | TRUE |  | 218839_at | TRUE |
| 218844_at | TRUE |  | 218844_at | TRUE |
| 218865_at | TRUE |  | 218865_at | TRUE |
| 218870_at | TRUE |  | 218870_at | TRUE |
| 218882_s_at | TRUE |  | 218882_s_at | TRUE |
| 218913_s_at | TRUE |  | 218913_s_at | TRUE |
| 218917_s_at | TRUE |  | 218917_s_at | TRUE |
| 218918_at | TRUE |  | 218918_at | TRUE |
| 218924_s_at | TRUE |  | 218924_s_at | TRUE |
| 218932_at | TRUE |  | 218932_at | TRUE |
| 218952_at | TRUE |  | 218952_at | TRUE |
| 218971_s_at | TRUE |  | 218971_s_at | TRUE |
| 218977_s_at | TRUE |  | 218977_s_at | TRUE |
| 218994_s_at | TRUE |  | 218994_s_at | TRUE |
| 218998_at | TRUE |  | 218998_at | TRUE |
| 219011_at | TRUE |  | 219011_at | TRUE |
| 219015_s_at | TRUE |  | 219015_s_at | TRUE |
| 219090_at | TRUE |  | 219090_at | TRUE |
| 219094_at | TRUE |  | 219094_at | TRUE |
| 219103_at | TRUE |  | 219103_at | TRUE |
| 219126_at | TRUE |  | 219126_at | TRUE |
| 219180_s_at | TRUE |  | 219180_s_at | TRUE |
| 219181_at | TRUE |  | 219181_at | TRUE |
| 219183_s_at | TRUE |  | 219183_s_at | TRUE |
| 219191_s_at | TRUE |  | 219191_s_at | TRUE |
| 219202_at | TRUE |  | 219202_at | TRUE |
| 219211_at | TRUE |  | 219211_at | TRUE |
| 219235_s_at | TRUE |  | 219235_s_at | TRUE |
| 219243_at | TRUE |  | 219243_at | TRUE |
| 219275_at | TRUE |  | 219275_at | TRUE |
| 219277_s_at | TRUE |  | 219277_s_at | TRUE |
| 219284_at | TRUE |  | 219284_at | TRUE |
| 219286_s_at | TRUE |  | 219286_s_at | TRUE |
| 219289_at | TRUE |  | 219289_at | TRUE |
| 219316_s_at | TRUE |  | 219316_s_at | TRUE |
| 219322_s_at | TRUE |  | 219322_s_at | TRUE |
| 219330_at | TRUE |  | 219330_at | TRUE |
| 219344_at | TRUE |  | 219344_at | TRUE |
| 219358_s_at | TRUE |  | 219358_s_at | TRUE |
| 219368_at | TRUE |  | 219368_at | TRUE |
| 219375_at | TRUE |  | 219375_at | TRUE |
| 219382_at | TRUE |  | 219382_at | TRUE |
| 219409_at | TRUE |  | 219409_at | TRUE |
| 219424_at | TRUE |  | 219424_at | TRUE |
| 219425_at | TRUE |  | 219425_at | TRUE |
| 219426_at | TRUE |  | 219426_at | TRUE |
| 219441_s_at | TRUE |  | 219441_s_at | TRUE |
| 219473_at | TRUE |  | 219473_at | TRUE |
| 219505_at | TRUE |  | 219505_at | TRUE |
| 219509_at | TRUE |  | 219509_at | TRUE |
| 219532_at | TRUE |  | 219532_at | TRUE |
| 219570_at | TRUE |  | 219570_at | TRUE |
| 219581_at | TRUE |  | 219581_at | TRUE |
| 219593_at | TRUE |  | 219593_at | TRUE |
| 219603_s_at | TRUE |  | 219603_s_at | TRUE |
| 219619_at | TRUE |  | 219619_at | TRUE |
| 219620_x_at | TRUE |  | 219620_x_at | TRUE |
| 219666_at | TRUE |  | 219666_at | TRUE |
| 219688_at | TRUE |  | 219688_at | TRUE |
| 219690_at | TRUE |  | 219690_at | TRUE |
| 219694_at | TRUE |  | 219694_at | TRUE |
| 219725_at | TRUE |  | 219725_at | TRUE |
| 219740_at | TRUE |  | 219740_at | TRUE |
| 219758_at | TRUE |  | 219758_at | TRUE |
| 219777_at | TRUE |  | 219777_at | TRUE |
| 219788_at | TRUE |  | 219788_at | TRUE |
| 219807_x_at | TRUE |  | 219807_x_at | TRUE |
| 219815_at | TRUE |  | 219815_at | TRUE |
| 219818_s_at | TRUE |  | 219818_s_at | TRUE |
| 219826_at | TRUE |  | 219826_at | TRUE |
| 219837_s_at | TRUE |  | 219837_s_at | TRUE |
| 219843_at | TRUE |  | 219843_at | TRUE |
| 219848_s_at | TRUE |  | 219848_s_at | TRUE |
| 219863_at | TRUE |  | 219863_at | TRUE |
| 219877_at | TRUE |  | 219877_at | TRUE |
| 219894_at | TRUE |  | 219894_at | TRUE |
| 219939_s_at | TRUE |  | 219939_s_at | TRUE |
| 219994_at | TRUE |  | 219994_at | TRUE |
| 220005_at | TRUE |  | 220005_at | TRUE |
| 220032_at | TRUE |  | 220032_at | TRUE |
| 220033_at | TRUE |  | 220033_at | TRUE |
| 220045_at | TRUE |  | 220045_at | TRUE |
| 220072_at | TRUE |  | 220072_at | TRUE |
| 220079_s_at | TRUE |  | 220079_s_at | TRUE |
| 220088_at | TRUE |  | 220088_at | TRUE |
| 220103_s_at | TRUE |  | 220103_s_at | TRUE |
| 220146_at | TRUE |  | 220146_at | TRUE |
| 220162_s_at | TRUE |  | 220162_s_at | TRUE |
| 220178_at | TRUE |  | 220178_at | TRUE |
| 220188_at | TRUE |  | 220188_at | TRUE |
| 220206_at | TRUE |  | 220206_at | TRUE |
| 220298_s_at | TRUE |  | 220298_s_at | TRUE |
| 220299_at | TRUE |  | 220299_at | TRUE |
| 220324_at | TRUE |  | 220324_at | TRUE |
| 220330_s_at | TRUE |  | 220330_s_at | TRUE |
| 220361_at | TRUE |  | 220361_at | TRUE |
| 220387_s_at | TRUE |  | 220387_s_at | TRUE |
| 220416_at | TRUE |  | 220416_at | TRUE |
| 220434_at | TRUE |  | 220434_at | TRUE |
| 220447_at | TRUE |  | 220447_at | TRUE |
| 220462_at | TRUE |  | 220462_at | TRUE |
| 220526_s_at | TRUE |  | 220526_s_at | TRUE |
| 220551_at | TRUE |  | 220551_at | TRUE |
| 220585_at | TRUE |  | 220585_at | TRUE |
| 220657_at | TRUE |  | 220657_at | TRUE |
| 220731_s_at | TRUE |  | 220731_s_at | TRUE |
| 220748_s_at | TRUE |  | 220748_s_at | TRUE |
| 220750_s_at | TRUE |  | 220750_s_at | TRUE |
| 220769_s_at | TRUE |  | 220769_s_at | TRUE |
| 220840_s_at | TRUE |  | 220840_s_at | TRUE |
| 220911_s_at | TRUE |  | 220911_s_at | TRUE |
| 220915_s_at | TRUE |  | 220915_s_at | TRUE |
| 220938_s_at | TRUE |  | 220938_s_at | TRUE |
| 220998_s_at | TRUE |  | 220998_s_at | TRUE |
| 221026_s_at | TRUE |  | 221026_s_at | TRUE |
| 221047_s_at | TRUE |  | 221047_s_at | TRUE |
| 221081_s_at | TRUE |  | 221081_s_at | TRUE |
| 221086_s_at | TRUE |  | 221086_s_at | TRUE |
| 221123_x_at | TRUE |  | 221123_x_at | TRUE |
| 221178_at | TRUE |  | 221178_at | TRUE |
| 221204_s_at | TRUE |  | 221204_s_at | TRUE |
| 221217_s_at | TRUE |  | 221217_s_at | TRUE |
| 221269_s_at | TRUE |  | 221269_s_at | TRUE |
| 221279_at | TRUE |  | 221279_at | TRUE |
| 221293_s_at | TRUE |  | 221293_s_at | TRUE |
| 221317_x_at | TRUE |  | 221317_x_at | TRUE |
| 221321_s_at | TRUE |  | 221321_s_at | TRUE |
| 221430_s_at | TRUE |  | 221430_s_at | TRUE |
| 221435_x_at | TRUE |  | 221435_x_at | TRUE |
| 221437_s_at | TRUE |  | 221437_s_at | TRUE |
| 221486_at | TRUE |  | 221486_at | TRUE |
| 221510_s_at | TRUE |  | 221510_s_at | TRUE |
| 221512_at | TRUE |  | 221512_at | TRUE |
| 221530_s_at | TRUE |  | 221530_s_at | TRUE |
| 221561_at | TRUE |  | 221561_at | TRUE |
| 221581_s_at | TRUE |  | 221581_s_at | TRUE |
| 221631_at | TRUE |  | 221631_at | TRUE |
| 221666_s_at | TRUE |  | 221666_s_at | TRUE |
| 221675_s_at | TRUE |  | 221675_s_at | TRUE |
| 221698_s_at | TRUE |  | 221698_s_at | TRUE |
| 221710_x_at | TRUE |  | 221710_x_at | TRUE |
| 221718_s_at | TRUE |  | 221718_s_at | TRUE |
| 221725_at | TRUE |  | 221725_at | TRUE |
| 221727_at | TRUE |  | 221727_at | TRUE |
| 221750_at | TRUE |  | 221750_at | TRUE |
| 221773_at | TRUE |  | 221773_at | TRUE |
| 221801_x_at | TRUE |  | 221801_x_at | TRUE |
| 221805_at | TRUE |  | 221805_at | TRUE |
| 221813_at | TRUE |  | 221813_at | TRUE |
| 221816_s_at | TRUE |  | 221816_s_at | TRUE |
| 221875_x_at | TRUE |  | 221875_x_at | TRUE |
| 221910_at | TRUE |  | 221910_at | TRUE |
| 221914_at | TRUE |  | 221914_at | TRUE |
| 221916_at | TRUE |  | 221916_at | TRUE |
| 221923_s_at | TRUE |  | 221923_s_at | TRUE |
| 221942_s_at | TRUE |  | 221942_s_at | TRUE |
| 221944_at | TRUE |  | 221944_at | TRUE |
| 221945_at | TRUE |  | 221945_at | TRUE |
| 221952_x_at | TRUE |  | 221952_x_at | TRUE |
| 221958_s_at | TRUE |  | 221958_s_at | TRUE |
| 221972_s_at | TRUE |  | 221972_s_at | TRUE |
| 221984_s_at | TRUE |  | 221984_s_at | TRUE |
| 221986_s_at | TRUE |  | 221986_s_at | TRUE |
| 221998_s_at | TRUE |  | 221998_s_at | TRUE |
| 222000_at | TRUE |  | 222000_at | TRUE |
| 222024_s_at | TRUE |  | 222024_s_at | TRUE |
| 222028_at | TRUE |  | 222028_at | TRUE |
| 222031_at | TRUE |  | 222031_at | TRUE |
| 222099_s_at | TRUE |  | 222099_s_at | TRUE |
| 222145_at | TRUE |  | 222145_at | TRUE |
| 222153_at | TRUE |  | 222153_at | TRUE |
| 222154_s_at | TRUE |  | 222154_s_at | TRUE |
| 222170_at | TRUE |  | 222170_at | TRUE |
| 222212_s_at | TRUE |  | 222212_s_at | TRUE |
| 222218_s_at | TRUE |  | 222218_s_at | TRUE |
| 222236_s_at | TRUE |  | 222236_s_at | TRUE |
| 222244_s_at | TRUE |  | 222244_s_at | TRUE |
| 222297_x_at | TRUE |  | 222297_x_at | TRUE |
| 222344_at | TRUE |  | 222344_at | TRUE |
| 222360_at | TRUE |  | 222360_at | TRUE |
| 222401_s_at | TRUE |  | 222401_s_at | TRUE |
| 222430_s_at | TRUE |  | 222430_s_at | TRUE |
| 222440_s_at | TRUE |  | 222440_s_at | TRUE |
| 222447_at | TRUE |  | 222447_at | TRUE |
| 222452_s_at | TRUE |  | 222452_s_at | TRUE |
| 222459_at | TRUE |  | 222459_at | TRUE |
| 222468_at | TRUE |  | 222468_at | TRUE |
| 222478_at | TRUE |  | 222478_at | TRUE |
| 222495_at | TRUE |  | 222495_at | TRUE |
| 222496_s_at | TRUE |  | 222496_s_at | TRUE |
| 222505_at | TRUE |  | 222505_at | TRUE |
| 222506_at | TRUE |  | 222506_at | TRUE |
| 222514_at | TRUE |  | 222514_at | TRUE |
| 222528_s_at | TRUE |  | 222528_s_at | TRUE |
| 222529_at | TRUE |  | 222529_at | TRUE |
| 222553_x_at | TRUE |  | 222553_x_at | TRUE |
| 222557_at | TRUE |  | 222557_at | TRUE |
| 222579_at | TRUE |  | 222579_at | TRUE |
| 222589_at | TRUE |  | 222589_at | TRUE |
| 222621_at | TRUE |  | 222621_at | TRUE |
| 222701_s_at | TRUE |  | 222701_s_at | TRUE |
| 222717_at | TRUE |  | 222717_at | TRUE |
| 222729_at | TRUE |  | 222729_at | TRUE |
| 222788_s_at | TRUE |  | 222788_s_at | TRUE |
| 222789_at | TRUE |  | 222789_at | TRUE |
| 222790_s_at | TRUE |  | 222790_s_at | TRUE |
| 222791_at | TRUE |  | 222791_at | TRUE |
| 222799_at | TRUE |  | 222799_at | TRUE |
| 222800_at | TRUE |  | 222800_at | TRUE |
| 222801_s_at | TRUE |  | 222801_s_at | TRUE |
| 222833_at | TRUE |  | 222833_at | TRUE |
| 222834_s_at | TRUE |  | 222834_s_at | TRUE |
| 222835_at | TRUE |  | 222835_at | TRUE |
| 222866_s_at | TRUE |  | 222866_s_at | TRUE |
| 222872_x_at | TRUE |  | 222872_x_at | TRUE |
| 222876_s_at | TRUE |  | 222876_s_at | TRUE |
| 222880_at | TRUE |  | 222880_at | TRUE |
| 222881_at | TRUE |  | 222881_at | TRUE |
| 222889_at | TRUE |  | 222889_at | TRUE |
| 222920_s_at | TRUE |  | 222920_s_at | TRUE |
| 222929_at | TRUE |  | 222929_at | TRUE |
| 222975_s_at | TRUE |  | 222975_s_at | TRUE |
| 222999_s_at | TRUE |  | 222999_s_at | TRUE |
| 223015_at | TRUE |  | 223015_at | TRUE |
| 223017_at | TRUE |  | 223017_at | TRUE |
| 223022_s_at | TRUE |  | 223022_s_at | TRUE |
| 223027_at | TRUE |  | 223027_at | TRUE |
| 223028_s_at | TRUE |  | 223028_s_at | TRUE |
| 223051_at | TRUE |  | 223051_at | TRUE |
| 223053_x_at | TRUE |  | 223053_x_at | TRUE |
| 223077_at | TRUE |  | 223077_at | TRUE |
| 223097_at | TRUE |  | 223097_at | TRUE |
| 223119_s_at | TRUE |  | 223119_s_at | TRUE |
| 223120_at | TRUE |  | 223120_at | TRUE |
| 223158_s_at | TRUE |  | 223158_s_at | TRUE |
| 223175_s_at | TRUE |  | 223175_s_at | TRUE |
| 223185_s_at | TRUE |  | 223185_s_at | TRUE |
| 223216_x_at | TRUE |  | 223216_x_at | TRUE |
| 223223_at | TRUE |  | 223223_at | TRUE |
| 223234_at | TRUE |  | 223234_at | TRUE |
| 223275_at | TRUE |  | 223275_at | TRUE |
| 223280_x_at | TRUE |  | 223280_x_at | TRUE |
| 223303_at | TRUE |  | 223303_at | TRUE |
| 223331_s_at | TRUE |  | 223331_s_at | TRUE |
| 223335_at | TRUE |  | 223335_at | TRUE |
| 223343_at | TRUE |  | 223343_at | TRUE |
| 223394_at | TRUE |  | 223394_at | TRUE |
| 223398_at | TRUE |  | 223398_at | TRUE |
| 223402_at | TRUE |  | 223402_at | TRUE |
| 223434_at | TRUE |  | 223434_at | TRUE |
| 223452_s_at | TRUE |  | 223452_s_at | TRUE |
| 223454_at | TRUE |  | 223454_at | TRUE |
| 223456_s_at | TRUE |  | 223456_s_at | TRUE |
| 223458_at | TRUE |  | 223458_at | TRUE |
| 223500_at | TRUE |  | 223500_at | TRUE |
| 223529_at | TRUE |  | 223529_at | TRUE |
| 223534_s_at | TRUE |  | 223534_s_at | TRUE |
| 223536_at | TRUE |  | 223536_at | TRUE |
| 223543_at | TRUE |  | 223543_at | TRUE |
| 223553_s_at | TRUE |  | 223553_s_at | TRUE |
| 223562_at | TRUE |  | 223562_at | TRUE |
| 223583_at | TRUE |  | 223583_at | TRUE |
| 223591_at | TRUE |  | 223591_at | TRUE |
| 223615_at | TRUE |  | 223615_at | TRUE |
| 223617_x_at | TRUE |  | 223617_x_at | TRUE |
| 223622_s_at | TRUE |  | 223622_s_at | TRUE |
| 223640_at | TRUE |  | 223640_at | TRUE |
| 223654_s_at | TRUE |  | 223654_s_at | TRUE |
| 223680_at | TRUE |  | 223680_at | TRUE |
| 223692_at | TRUE |  | 223692_at | TRUE |
| 223703_at | TRUE |  | 223703_at | TRUE |
| 223727_at | TRUE |  | 223727_at | TRUE |
| 223750_s_at | TRUE |  | 223750_s_at | TRUE |
| 223751_x_at | TRUE |  | 223751_x_at | TRUE |
| 223766_at | TRUE |  | 223766_at | TRUE |
| 223767_at | TRUE |  | 223767_at | TRUE |
| 223769_x_at | TRUE |  | 223769_x_at | TRUE |
| 223773_s_at | TRUE |  | 223773_s_at | TRUE |
| 223774_at | TRUE |  | 223774_at | TRUE |
| 223783_s_at | TRUE |  | 223783_s_at | TRUE |
| 223786_at | TRUE |  | 223786_at | TRUE |
| 223809_at | TRUE |  | 223809_at | TRUE |
| 223849_s_at | TRUE |  | 223849_s_at | TRUE |
| 223852_s_at | TRUE |  | 223852_s_at | TRUE |
| 223879_s_at | TRUE |  | 223879_s_at | TRUE |
| 223881_at | TRUE |  | 223881_at | TRUE |
| 223892_s_at | TRUE |  | 223892_s_at | TRUE |
| 223913_s_at | TRUE |  | 223913_s_at | TRUE |
| 223922_x_at | TRUE |  | 223922_x_at | TRUE |
| 223966_at | TRUE |  | 223966_at | TRUE |
| 224018_s_at | TRUE |  | 224018_s_at | TRUE |
| 224046_s_at | TRUE |  | 224046_s_at | TRUE |
| 224190_x_at | TRUE |  | 224190_x_at | TRUE |
| 224209_s_at | TRUE |  | 224209_s_at | TRUE |
| 224217_s_at | TRUE |  | 224217_s_at | TRUE |
| 224229_s_at | TRUE |  | 224229_s_at | TRUE |
| 224252_s_at | TRUE |  | 224252_s_at | TRUE |
| 224280_s_at | TRUE |  | 224280_s_at | TRUE |
| 224281_s_at | TRUE |  | 224281_s_at | TRUE |
| 224301_x_at | TRUE |  | 224301_x_at | TRUE |
| 224312_x_at | TRUE |  | 224312_x_at | TRUE |
| 224315_at | TRUE |  | 224315_at | TRUE |
| 224356_x_at | TRUE |  | 224356_x_at | TRUE |
| 224359_s_at | TRUE |  | 224359_s_at | TRUE |
| 224393_s_at | TRUE |  | 224393_s_at | TRUE |
| 224451_x_at | TRUE |  | 224451_x_at | TRUE |
| 224472_x_at | TRUE |  | 224472_x_at | TRUE |
| 224496_s_at | TRUE |  | 224496_s_at | TRUE |
| 224562_at | TRUE |  | 224562_at | TRUE |
| 224563_at | TRUE |  | 224563_at | TRUE |
| 224578_at | TRUE |  | 224578_at | TRUE |
| 224580_at | TRUE |  | 224580_at | TRUE |
| 224586_x_at | TRUE |  | 224586_x_at | TRUE |
| 224591_at | TRUE |  | 224591_at | TRUE |
| 224592_x_at | TRUE |  | 224592_x_at | TRUE |
| 224624_at | TRUE |  | 224624_at | TRUE |
| 224650_at | TRUE |  | 224650_at | TRUE |
| 224659_at | TRUE |  | 224659_at | TRUE |
| 224706_at | TRUE |  | 224706_at | TRUE |
| 224708_at | TRUE |  | 224708_at | TRUE |
| 224727_at | TRUE |  | 224727_at | TRUE |
| 224735_at | TRUE |  | 224735_at | TRUE |
| 224772_at | TRUE |  | 224772_at | TRUE |
| 224773_at | TRUE |  | 224773_at | TRUE |
| 224807_at | TRUE |  | 224807_at | TRUE |
| 224811_at | TRUE |  | 224811_at | TRUE |
| 224819_at | TRUE |  | 224819_at | TRUE |
| 224820_at | TRUE |  | 224820_at | TRUE |
| 224846_at | TRUE |  | 224846_at | TRUE |
| 224884_at | TRUE |  | 224884_at | TRUE |
| 224893_at | TRUE |  | 224893_at | TRUE |
| 224898_at | TRUE |  | 224898_at | TRUE |
| 224901_at | TRUE |  | 224901_at | TRUE |
| 224906_at | TRUE |  | 224906_at | TRUE |
| 224912_at | TRUE |  | 224912_at | TRUE |
| 224916_at | TRUE |  | 224916_at | TRUE |
| 224921_at | TRUE |  | 224921_at | TRUE |
| 224923_at | TRUE |  | 224923_at | TRUE |
| 224929_at | TRUE |  | 224929_at | TRUE |
| 224941_at | TRUE |  | 224941_at | TRUE |
| 224954_at | TRUE |  | 224954_at | TRUE |
| 224970_at | TRUE |  | 224970_at | TRUE |
| 224975_at | TRUE |  | 224975_at | TRUE |
| 224985_at | TRUE |  | 224985_at | TRUE |
| 224989_at | TRUE |  | 224989_at | TRUE |
| 225000_at | TRUE |  | 225000_at | TRUE |
| 225005_at | TRUE |  | 225005_at | TRUE |
| 225011_at | TRUE |  | 225011_at | TRUE |
| 225023_at | TRUE |  | 225023_at | TRUE |
| 225043_at | TRUE |  | 225043_at | TRUE |
| 225051_at | TRUE |  | 225051_at | TRUE |
| 225056_at | TRUE |  | 225056_at | TRUE |
| 225059_at | TRUE |  | 225059_at | TRUE |
| 225111_s_at | TRUE |  | 225111_s_at | TRUE |
| 225123_at | TRUE |  | 225123_at | TRUE |
| 225130_at | TRUE |  | 225130_at | TRUE |
| 225136_at | TRUE |  | 225136_at | TRUE |
| 225180_at | TRUE |  | 225180_at | TRUE |
| 225208_s_at | TRUE |  | 225208_s_at | TRUE |
| 225209_s_at | TRUE |  | 225209_s_at | TRUE |
| 225217_s_at | TRUE |  | 225217_s_at | TRUE |
| 225220_at | TRUE |  | 225220_at | TRUE |
| 225225_at | TRUE |  | 225225_at | TRUE |
| 225228_at | TRUE |  | 225228_at | TRUE |
| 225230_at | TRUE |  | 225230_at | TRUE |
| 225245_x_at | TRUE |  | 225245_x_at | TRUE |
| 225255_at | TRUE |  | 225255_at | TRUE |
| 225257_at | TRUE |  | 225257_at | TRUE |
| 225265_at | TRUE |  | 225265_at | TRUE |
| 225269_s_at | TRUE |  | 225269_s_at | TRUE |
| 225279_s_at | TRUE |  | 225279_s_at | TRUE |
| 225288_at | TRUE |  | 225288_at | TRUE |
| 225292_at | TRUE |  | 225292_at | TRUE |
| 225293_at | TRUE |  | 225293_at | TRUE |
| 225308_s_at | TRUE |  | 225308_s_at | TRUE |
| 225323_at | TRUE |  | 225323_at | TRUE |
| 225327_at | TRUE |  | 225327_at | TRUE |
| 225328_at | TRUE |  | 225328_at | TRUE |
| 225332_at | TRUE |  | 225332_at | TRUE |
| 225345_s_at | TRUE |  | 225345_s_at | TRUE |
| 225353_s_at | TRUE |  | 225353_s_at | TRUE |
| 225356_at | TRUE |  | 225356_at | TRUE |
| 225373_at | TRUE |  | 225373_at | TRUE |
| 225384_at | TRUE |  | 225384_at | TRUE |
| 225401_at | TRUE |  | 225401_at | TRUE |
| 225414_at | TRUE |  | 225414_at | TRUE |
| 225475_at | TRUE |  | 225475_at | TRUE |
| 225502_at | TRUE |  | 225502_at | TRUE |
| 225520_at | TRUE |  | 225520_at | TRUE |
| 225577_at | TRUE |  | 225577_at | TRUE |
| 225578_at | TRUE |  | 225578_at | TRUE |
| 225579_at | TRUE |  | 225579_at | TRUE |
| 225593_at | TRUE |  | 225593_at | TRUE |
| 225605_at | TRUE |  | 225605_at | TRUE |
| 225612_s_at | TRUE |  | 225612_s_at | TRUE |
| 225618_at | TRUE |  | 225618_at | TRUE |
| 225627_s_at | TRUE |  | 225627_s_at | TRUE |
| 225646_at | TRUE |  | 225646_at | TRUE |
| 225698_at | TRUE |  | 225698_at | TRUE |
| 225700_at | TRUE |  | 225700_at | TRUE |
| 225755_at | TRUE |  | 225755_at | TRUE |
| 225763_at | TRUE |  | 225763_at | TRUE |
| 225777_at | TRUE |  | 225777_at | TRUE |
| 225782_at | TRUE |  | 225782_at | TRUE |
| 225784_s_at | TRUE |  | 225784_s_at | TRUE |
| 225790_at | TRUE |  | 225790_at | TRUE |
| 225803_at | TRUE |  | 225803_at | TRUE |
| 225806_at | TRUE |  | 225806_at | TRUE |
| 225807_at | TRUE |  | 225807_at | TRUE |
| 225829_at | TRUE |  | 225829_at | TRUE |
| 225840_at | TRUE |  | 225840_at | TRUE |
| 225869_s_at | TRUE |  | 225869_s_at | TRUE |
| 225878_at | TRUE |  | 225878_at | TRUE |
| 225890_at | TRUE |  | 225890_at | TRUE |
| 225897_at | TRUE |  | 225897_at | TRUE |
| 225900_at | TRUE |  | 225900_at | TRUE |
| 225992_at | TRUE |  | 225992_at | TRUE |
| 226000_at | TRUE |  | 226000_at | TRUE |
| 226017_at | TRUE |  | 226017_at | TRUE |
| 226019_at | TRUE |  | 226019_at | TRUE |
| 226020_s_at | TRUE |  | 226020_s_at | TRUE |
| 226066_at | TRUE |  | 226066_at | TRUE |
| 226068_at | TRUE |  | 226068_at | TRUE |
| 226086_at | TRUE |  | 226086_at | TRUE |
| 226088_at | TRUE |  | 226088_at | TRUE |
| 226093_at | TRUE |  | 226093_at | TRUE |
| 226096_at | TRUE |  | 226096_at | TRUE |
| 226113_at | TRUE |  | 226113_at | TRUE |
| 226114_at | TRUE |  | 226114_at | TRUE |
| 226116_at | TRUE |  | 226116_at | TRUE |
| 226133_s_at | TRUE |  | 226133_s_at | TRUE |
| 226160_at | TRUE |  | 226160_at | TRUE |
| 226170_at | TRUE |  | 226170_at | TRUE |
| 226191_at | TRUE |  | 226191_at | TRUE |
| 226192_at | TRUE |  | 226192_at | TRUE |
| 226197_at | TRUE |  | 226197_at | TRUE |
| 226198_at | TRUE |  | 226198_at | TRUE |
| 226217_at | TRUE |  | 226217_at | TRUE |
| 226219_at | TRUE |  | 226219_at | TRUE |
| 226239_at | TRUE |  | 226239_at | TRUE |
| 226261_at | TRUE |  | 226261_at | TRUE |
| 226269_at | TRUE |  | 226269_at | TRUE |
| 226271_at | TRUE |  | 226271_at | TRUE |
| 226296_s_at | TRUE |  | 226296_s_at | TRUE |
| 226303_at | TRUE |  | 226303_at | TRUE |
| 226333_at | TRUE |  | 226333_at | TRUE |
| 226343_at | TRUE |  | 226343_at | TRUE |
| 226350_at | TRUE |  | 226350_at | TRUE |
| 226373_at | TRUE |  | 226373_at | TRUE |
| 226375_at | TRUE |  | 226375_at | TRUE |
| 226377_at | TRUE |  | 226377_at | TRUE |
| 226390_at | TRUE |  | 226390_at | TRUE |
| 226397_s_at | TRUE |  | 226397_s_at | TRUE |
| 226430_at | TRUE |  | 226430_at | TRUE |
| 226459_at | TRUE |  | 226459_at | TRUE |
| 226474_at | TRUE |  | 226474_at | TRUE |
| 226494_at | TRUE |  | 226494_at | TRUE |
| 226497_s_at | TRUE |  | 226497_s_at | TRUE |
| 226498_at | TRUE |  | 226498_at | TRUE |
| 226532_at | TRUE |  | 226532_at | TRUE |
| 226573_at | TRUE |  | 226573_at | TRUE |
| 226582_at | TRUE |  | 226582_at | TRUE |
| 226590_at | TRUE |  | 226590_at | TRUE |
| 226592_at | TRUE |  | 226592_at | TRUE |
| 226599_at | TRUE |  | 226599_at | TRUE |
| 226601_at | TRUE |  | 226601_at | TRUE |
| 226612_at | TRUE |  | 226612_at | TRUE |
| 226618_at | TRUE |  | 226618_at | TRUE |
| 226627_at | TRUE |  | 226627_at | TRUE |
| 226640_at | TRUE |  | 226640_at | TRUE |
| 226653_at | TRUE |  | 226653_at | TRUE |
| 226659_at | TRUE |  | 226659_at | TRUE |
| 226723_at | TRUE |  | 226723_at | TRUE |
| 226725_at | TRUE |  | 226725_at | TRUE |
| 226761_at | TRUE |  | 226761_at | TRUE |
| 226796_at | TRUE |  | 226796_at | TRUE |
| 226800_at | TRUE |  | 226800_at | TRUE |
| 226802_s_at | TRUE |  | 226802_s_at | TRUE |
| 226806_s_at | TRUE |  | 226806_s_at | TRUE |
| 226811_at | TRUE |  | 226811_at | TRUE |
| 226818_at | TRUE |  | 226818_at | TRUE |
| 226820_at | TRUE |  | 226820_at | TRUE |
| 226823_at | TRUE |  | 226823_at | TRUE |
| 226841_at | TRUE |  | 226841_at | TRUE |
| 226864_at | TRUE |  | 226864_at | TRUE |
| 226865_at | TRUE |  | 226865_at | TRUE |
| 226878_at | TRUE |  | 226878_at | TRUE |
| 226881_at | TRUE |  | 226881_at | TRUE |
| 226895_at | TRUE |  | 226895_at | TRUE |
| 226899_at | TRUE |  | 226899_at | TRUE |
| 226907_at | TRUE |  | 226907_at | TRUE |
| 226909_at | TRUE |  | 226909_at | TRUE |
| 226961_at | TRUE |  | 226961_at | TRUE |
| 226964_at | TRUE |  | 226964_at | TRUE |
| 226968_at | TRUE |  | 226968_at | TRUE |
| 226976_at | TRUE |  | 226976_at | TRUE |
| 226989_at | TRUE |  | 226989_at | TRUE |
| 226991_at | TRUE |  | 226991_at | TRUE |
| 227019_at | TRUE |  | 227019_at | TRUE |
| 227039_at | TRUE |  | 227039_at | TRUE |
| 227053_at | TRUE |  | 227053_at | TRUE |
| 227066_at | TRUE |  | 227066_at | TRUE |
| 227081_at | TRUE |  | 227081_at | TRUE |
| 227091_at | TRUE |  | 227091_at | TRUE |
| 227095_at | TRUE |  | 227095_at | TRUE |
| 227096_at | TRUE |  | 227096_at | TRUE |
| 227105_at | TRUE |  | 227105_at | TRUE |
| 227107_at | TRUE |  | 227107_at | TRUE |
| 227124_at | TRUE |  | 227124_at | TRUE |
| 227143_s_at | TRUE |  | 227143_s_at | TRUE |
| 227150_at | TRUE |  | 227150_at | TRUE |
| 227168_at | TRUE |  | 227168_at | TRUE |
| 227176_at | TRUE |  | 227176_at | TRUE |
| 227182_at | TRUE |  | 227182_at | TRUE |
| 227184_at | TRUE |  | 227184_at | TRUE |
| 227188_at | TRUE |  | 227188_at | TRUE |
| 227189_at | TRUE |  | 227189_at | TRUE |
| 227195_at | TRUE |  | 227195_at | TRUE |
| 227196_at | TRUE |  | 227196_at | TRUE |
| 227206_at | TRUE |  | 227206_at | TRUE |
| 227210_at | TRUE |  | 227210_at | TRUE |
| 227214_at | TRUE |  | 227214_at | TRUE |
| 227240_at | TRUE |  | 227240_at | TRUE |
| 227253_at | TRUE |  | 227253_at | TRUE |
| 227265_at | TRUE |  | 227265_at | TRUE |
| 227266_s_at | TRUE |  | 227266_s_at | TRUE |
| 227276_at | TRUE |  | 227276_at | TRUE |
| 227284_at | TRUE |  | 227284_at | TRUE |
| 227300_at | TRUE |  | 227300_at | TRUE |
| 227313_at | TRUE |  | 227313_at | TRUE |
| 227327_at | TRUE |  | 227327_at | TRUE |
| 227339_at | TRUE |  | 227339_at | TRUE |
| 227340_s_at | TRUE |  | 227340_s_at | TRUE |
| 227344_at | TRUE |  | 227344_at | TRUE |
| 227346_at | TRUE |  | 227346_at | TRUE |
| 227353_at | TRUE |  | 227353_at | TRUE |
| 227355_at | TRUE |  | 227355_at | TRUE |
| 227365_at | TRUE |  | 227365_at | TRUE |
| 227369_at | TRUE |  | 227369_at | TRUE |
| 227434_at | TRUE |  | 227434_at | TRUE |
| 227448_at | TRUE |  | 227448_at | TRUE |
| 227453_at | TRUE |  | 227453_at | TRUE |
| 227484_at | TRUE |  | 227484_at | TRUE |
| 227514_at | TRUE |  | 227514_at | TRUE |
| 227516_at | TRUE |  | 227516_at | TRUE |
| 227562_at | TRUE |  | 227562_at | TRUE |
| 227584_at | TRUE |  | 227584_at | TRUE |
| 227585_at | TRUE |  | 227585_at | TRUE |
| 227614_at | TRUE |  | 227614_at | TRUE |
| 227644_at | TRUE |  | 227644_at | TRUE |
| 227645_at | TRUE |  | 227645_at | TRUE |
| 227647_at | TRUE |  | 227647_at | TRUE |
| 227700_x_at | TRUE |  | 227700_x_at | TRUE |
| 227726_at | TRUE |  | 227726_at | TRUE |
| 227748_at | TRUE |  | 227748_at | TRUE |
| 227756_at | TRUE |  | 227756_at | TRUE |
| 227769_at | TRUE |  | 227769_at | TRUE |
| 227792_at | TRUE |  | 227792_at | TRUE |
| 227817_at | TRUE |  | 227817_at | TRUE |
| 227824_at | TRUE |  | 227824_at | TRUE |
| 227847_at | TRUE |  | 227847_at | TRUE |
| 227889_at | TRUE |  | 227889_at | TRUE |
| 227898_s_at | TRUE |  | 227898_s_at | TRUE |
| 227924_at | TRUE |  | 227924_at | TRUE |
| 227954_at | TRUE |  | 227954_at | TRUE |
| 227965_at | TRUE |  | 227965_at | TRUE |
| 227975_at | TRUE |  | 227975_at | TRUE |
| 227983_at | TRUE |  | 227983_at | TRUE |
| 227992_s_at | TRUE |  | 227992_s_at | TRUE |
| 227995_at | TRUE |  | 227995_at | TRUE |
| 228028_at | TRUE |  | 228028_at | TRUE |
| 228062_at | TRUE |  | 228062_at | TRUE |
| 228063_s_at | TRUE |  | 228063_s_at | TRUE |
| 228064_at | TRUE |  | 228064_at | TRUE |
| 228083_at | TRUE |  | 228083_at | TRUE |
| 228100_at | TRUE |  | 228100_at | TRUE |
| 228127_at | TRUE |  | 228127_at | TRUE |
| 228129_at | TRUE |  | 228129_at | TRUE |
| 228131_at | TRUE |  | 228131_at | TRUE |
| 228140_s_at | TRUE |  | 228140_s_at | TRUE |
| 228143_at | TRUE |  | 228143_at | TRUE |
| 228174_at | TRUE |  | 228174_at | TRUE |
| 228176_at | TRUE |  | 228176_at | TRUE |
| 228184_at | TRUE |  | 228184_at | TRUE |
| 228221_at | TRUE |  | 228221_at | TRUE |
| 228245_s_at | TRUE |  | 228245_s_at | TRUE |
| 228261_at | TRUE |  | 228261_at | TRUE |
| 228269_x_at | TRUE |  | 228269_x_at | TRUE |
| 228285_at | TRUE |  | 228285_at | TRUE |
| 228305_at | TRUE |  | 228305_at | TRUE |
| 228349_at | TRUE |  | 228349_at | TRUE |
| 228369_at | TRUE |  | 228369_at | TRUE |
| 228375_at | TRUE |  | 228375_at | TRUE |
| 228388_at | TRUE |  | 228388_at | TRUE |
| 228410_at | TRUE |  | 228410_at | TRUE |
| 228433_at | TRUE |  | 228433_at | TRUE |
| 228442_at | TRUE |  | 228442_at | TRUE |
| 228450_at | TRUE |  | 228450_at | TRUE |
| 228464_at | TRUE |  | 228464_at | TRUE |
| 228480_at | TRUE |  | 228480_at | TRUE |
| 228509_at | TRUE |  | 228509_at | TRUE |
| 228521_s_at | TRUE |  | 228521_s_at | TRUE |
| 228532_at | TRUE |  | 228532_at | TRUE |
| 228538_at | TRUE |  | 228538_at | TRUE |
| 228544_s_at | TRUE |  | 228544_s_at | TRUE |
| 228548_at | TRUE |  | 228548_at | TRUE |
| 228550_at | TRUE |  | 228550_at | TRUE |
| 228566_at | TRUE |  | 228566_at | TRUE |
| 228605_at | TRUE |  | 228605_at | TRUE |
| 228641_at | TRUE |  | 228641_at | TRUE |
| 228658_at | TRUE |  | 228658_at | TRUE |
| 228661_s_at | TRUE |  | 228661_s_at | TRUE |
| 228674_s_at | TRUE |  | 228674_s_at | TRUE |
| 228677_s_at | TRUE |  | 228677_s_at | TRUE |
| 228680_at | TRUE |  | 228680_at | TRUE |
| 228726_at | TRUE |  | 228726_at | TRUE |
| 228728_at | TRUE |  | 228728_at | TRUE |
| 228733_at | TRUE |  | 228733_at | TRUE |
| 228741_s_at | TRUE |  | 228741_s_at | TRUE |
| 228761_at | TRUE |  | 228761_at | TRUE |
| 228778_at | TRUE |  | 228778_at | TRUE |
| 228796_at | TRUE |  | 228796_at | TRUE |
| 228804_at | TRUE |  | 228804_at | TRUE |
| 228805_at | TRUE |  | 228805_at | TRUE |
| 228811_at | TRUE |  | 228811_at | TRUE |
| 228813_at | TRUE |  | 228813_at | TRUE |
| 228855_at | TRUE |  | 228855_at | TRUE |
| 228858_at | TRUE |  | 228858_at | TRUE |
| 228869_at | TRUE |  | 228869_at | TRUE |
| 228882_at | TRUE |  | 228882_at | TRUE |
| 228890_at | TRUE |  | 228890_at | TRUE |
| 228920_at | TRUE |  | 228920_at | TRUE |
| 228949_at | TRUE |  | 228949_at | TRUE |
| 228950_s_at | TRUE |  | 228950_s_at | TRUE |
| 228990_at | TRUE |  | 228990_at | TRUE |
| 229011_at | TRUE |  | 229011_at | TRUE |
| 229019_at | TRUE |  | 229019_at | TRUE |
| 229029_at | TRUE |  | 229029_at | TRUE |
| 229057_at | TRUE |  | 229057_at | TRUE |
| 229083_at | TRUE |  | 229083_at | TRUE |
| 229084_at | TRUE |  | 229084_at | TRUE |
| 229123_at | TRUE |  | 229123_at | TRUE |
| 229134_at | TRUE |  | 229134_at | TRUE |
| 229145_at | TRUE |  | 229145_at | TRUE |
| 229146_at | TRUE |  | 229146_at | TRUE |
| 229160_at | TRUE |  | 229160_at | TRUE |
| 229201_at | TRUE |  | 229201_at | TRUE |
| 229204_at | TRUE |  | 229204_at | TRUE |
| 229221_at | TRUE |  | 229221_at | TRUE |
| 229234_at | TRUE |  | 229234_at | TRUE |
| 229259_at | TRUE |  | 229259_at | TRUE |
| 229265_at | TRUE |  | 229265_at | TRUE |
| 229271_x_at | TRUE |  | 229271_x_at | TRUE |
| 229295_at | TRUE |  | 229295_at | TRUE |
| 229300_at | TRUE |  | 229300_at | TRUE |
| 229308_at | TRUE |  | 229308_at | TRUE |
| 229313_at | TRUE |  | 229313_at | TRUE |
| 229335_at | TRUE |  | 229335_at | TRUE |
| 229357_at | TRUE |  | 229357_at | TRUE |
| 229370_at | TRUE |  | 229370_at | TRUE |
| 229406_at | TRUE |  | 229406_at | TRUE |
| 229419_at | TRUE |  | 229419_at | TRUE |
| 229425_at | TRUE |  | 229425_at | TRUE |
| 229428_at | TRUE |  | 229428_at | TRUE |
| 229435_at | TRUE |  | 229435_at | TRUE |
| 229497_at | TRUE |  | 229497_at | TRUE |
| 229498_at | TRUE |  | 229498_at | TRUE |
| 229507_at | TRUE |  | 229507_at | TRUE |
| 229550_at | TRUE |  | 229550_at | TRUE |
| 229560_at | TRUE |  | 229560_at | TRUE |
| 229597_s_at | TRUE |  | 229597_s_at | TRUE |
| 229651_at | TRUE |  | 229651_at | TRUE |
| 229664_at | TRUE |  | 229664_at | TRUE |
| 229672_at | TRUE |  | 229672_at | TRUE |
| 229691_at | TRUE |  | 229691_at | TRUE |
| 229709_at | TRUE |  | 229709_at | TRUE |
| 229712_at | TRUE |  | 229712_at | TRUE |
| 229715_at | TRUE |  | 229715_at | TRUE |
| 229722_at | TRUE |  | 229722_at | TRUE |
| 229725_at | TRUE |  | 229725_at | TRUE |
| 229743_at | TRUE |  | 229743_at | TRUE |
| 229759_s_at | TRUE |  | 229759_s_at | TRUE |
| 229760_at | TRUE |  | 229760_at | TRUE |
| 229774_at | TRUE |  | 229774_at | TRUE |
| 229812_at | TRUE |  | 229812_at | TRUE |
| 229816_at | TRUE |  | 229816_at | TRUE |
| 229818_at | TRUE |  | 229818_at | TRUE |
| 229823_at | TRUE |  | 229823_at | TRUE |
| 229824_at | TRUE |  | 229824_at | TRUE |
| 229852_at | TRUE |  | 229852_at | TRUE |
| 229874_x_at | TRUE |  | 229874_x_at | TRUE |
| 229875_at | TRUE |  | 229875_at | TRUE |
| 229893_at | TRUE |  | 229893_at | TRUE |
| 229922_at | TRUE |  | 229922_at | TRUE |
| 229932_at | TRUE |  | 229932_at | TRUE |
| 229937_x_at | TRUE |  | 229937_x_at | TRUE |
| 229953_x_at | TRUE |  | 229953_x_at | TRUE |
| 229968_at | TRUE |  | 229968_at | TRUE |
| 229997_at | TRUE |  | 229997_at | TRUE |
| 230012_at | TRUE |  | 230012_at | TRUE |
| 230022_at | TRUE |  | 230022_at | TRUE |
| 230069_at | TRUE |  | 230069_at | TRUE |
| 230112_at | TRUE |  | 230112_at | TRUE |
| 230117_at | TRUE |  | 230117_at | TRUE |
| 230123_at | TRUE |  | 230123_at | TRUE |
| 230137_at | TRUE |  | 230137_at | TRUE |
| 230194_at | TRUE |  | 230194_at | TRUE |
| 230204_at | TRUE |  | 230204_at | TRUE |
| 230217_at | TRUE |  | 230217_at | TRUE |
| 230230_at | TRUE |  | 230230_at | TRUE |
| 230252_at | TRUE |  | 230252_at | TRUE |
| 230258_at | TRUE |  | 230258_at | TRUE |
| 230259_at | TRUE |  | 230259_at | TRUE |
| 230272_at | TRUE |  | 230272_at | TRUE |
| 230275_at | TRUE |  | 230275_at | TRUE |
| 230280_at | TRUE |  | 230280_at | TRUE |
| 230296_at | TRUE |  | 230296_at | TRUE |
| 230316_at | TRUE |  | 230316_at | TRUE |
| 230368_at | TRUE |  | 230368_at | TRUE |
| 230369_at | TRUE |  | 230369_at | TRUE |
| 230372_at | TRUE |  | 230372_at | TRUE |
| 230391_at | TRUE |  | 230391_at | TRUE |
| 230417_at | TRUE |  | 230417_at | TRUE |
| 230418_s_at | TRUE |  | 230418_s_at | TRUE |
| 230433_at | TRUE |  | 230433_at | TRUE |
| 230434_at | TRUE |  | 230434_at | TRUE |
| 230445_at | TRUE |  | 230445_at | TRUE |
| 230475_at | TRUE |  | 230475_at | TRUE |
| 230551_at | TRUE |  | 230551_at | TRUE |
| 230561_s_at | TRUE |  | 230561_s_at | TRUE |
| 230570_at | TRUE |  | 230570_at | TRUE |
| 230635_at | TRUE |  | 230635_at | TRUE |
| 230645_at | TRUE |  | 230645_at | TRUE |
| 230657_at | TRUE |  | 230657_at | TRUE |
| 230664_at | TRUE |  | 230664_at | TRUE |
| 230706_s_at | TRUE |  | 230706_s_at | TRUE |
| 230755_at | TRUE |  | 230755_at | TRUE |
| 230821_at | TRUE |  | 230821_at | TRUE |
| 230826_at | TRUE |  | 230826_at | TRUE |
| 230839_at | TRUE |  | 230839_at | TRUE |
| 230848_s_at | TRUE |  | 230848_s_at | TRUE |
| 230859_at | TRUE |  | 230859_at | TRUE |
| 230866_at | TRUE |  | 230866_at | TRUE |
| 230869_at | TRUE |  | 230869_at | TRUE |
| 230908_at | TRUE |  | 230908_at | TRUE |
| 230925_at | TRUE |  | 230925_at | TRUE |
| 230968_at | TRUE |  | 230968_at | TRUE |
| 231003_at | TRUE |  | 231003_at | TRUE |
| 231015_at | TRUE |  | 231015_at | TRUE |
| 231040_at | TRUE |  | 231040_at | TRUE |
| 231106_at | TRUE |  | 231106_at | TRUE |
| 231130_at | TRUE |  | 231130_at | TRUE |
| 231185_at | TRUE |  | 231185_at | TRUE |
| 231220_at | TRUE |  | 231220_at | TRUE |
| 231260_at | TRUE |  | 231260_at | TRUE |
| 231262_at | TRUE |  | 231262_at | TRUE |
| 231323_at | TRUE |  | 231323_at | TRUE |
| 231336_at | TRUE |  | 231336_at | TRUE |
| 231341_at | TRUE |  | 231341_at | TRUE |
| 231364_at | TRUE |  | 231364_at | TRUE |
| 231391_at | TRUE |  | 231391_at | TRUE |
| 231470_at | TRUE |  | 231470_at | TRUE |
| 231489_x_at | TRUE |  | 231489_x_at | TRUE |
| 231514_at | TRUE |  | 231514_at | TRUE |
| 231526_at | TRUE |  | 231526_at | TRUE |
| 231557_at | TRUE |  | 231557_at | TRUE |
| 231577_s_at | TRUE |  | 231577_s_at | TRUE |
| 231608_at | TRUE |  | 231608_at | TRUE |
| 231650_s_at | TRUE |  | 231650_s_at | TRUE |
| 231714_s_at | TRUE |  | 231714_s_at | TRUE |
| 231737_at | TRUE |  | 231737_at | TRUE |
| 231740_at | TRUE |  | 231740_at | TRUE |
| 231747_at | TRUE |  | 231747_at | TRUE |
| 231765_at | TRUE |  | 231765_at | TRUE |
| 231788_at | TRUE |  | 231788_at | TRUE |
| 231791_at | TRUE |  | 231791_at | TRUE |
| 231804_at | TRUE |  | 231804_at | TRUE |
| 231808_at | TRUE |  | 231808_at | TRUE |
| 231836_at | TRUE |  | 231836_at | TRUE |
| 231854_at | TRUE |  | 231854_at | TRUE |
| 231867_at | TRUE |  | 231867_at | TRUE |
| 231887_s_at | TRUE |  | 231887_s_at | TRUE |
| 231890_at | TRUE |  | 231890_at | TRUE |
| 231935_at | TRUE |  | 231935_at | TRUE |
| 231950_at | TRUE |  | 231950_at | TRUE |
| 231954_at | TRUE |  | 231954_at | TRUE |
| 231972_at | TRUE |  | 231972_at | TRUE |
| 231986_at | TRUE |  | 231986_at | TRUE |
| 231990_at | TRUE |  | 231990_at | TRUE |
| 232015_at | TRUE |  | 232015_at | TRUE |
| 232032_x_at | TRUE |  | 232032_x_at | TRUE |
| 232045_at | TRUE |  | 232045_at | TRUE |
| 232054_at | TRUE |  | 232054_at | TRUE |
| 232081_at | TRUE |  | 232081_at | TRUE |
| 232105_at | TRUE |  | 232105_at | TRUE |
| 232111_at | TRUE |  | 232111_at | TRUE |
| 232122_s_at | TRUE |  | 232122_s_at | TRUE |
| 232172_at | TRUE |  | 232172_at | TRUE |
| 232244_at | TRUE |  | 232244_at | TRUE |
| 232295_at | TRUE |  | 232295_at | TRUE |
| 232317_at | TRUE |  | 232317_at | TRUE |
| 232350_x_at | TRUE |  | 232350_x_at | TRUE |
| 232487_at | TRUE |  | 232487_at | TRUE |
| 232543_x_at | TRUE |  | 232543_x_at | TRUE |
| 232571_at | TRUE |  | 232571_at | TRUE |
| 232589_at | TRUE |  | 232589_at | TRUE |
| 232617_at | TRUE |  | 232617_at | TRUE |
| 232693_s_at | TRUE |  | 232693_s_at | TRUE |
| 232717_at | TRUE |  | 232717_at | TRUE |
| 232738_at | TRUE |  | 232738_at | TRUE |
| 232748_at | TRUE |  | 232748_at | TRUE |
| 232758_s_at | TRUE |  | 232758_s_at | TRUE |
| 232760_at | TRUE |  | 232760_at | TRUE |
| 232780_s_at | TRUE |  | 232780_s_at | TRUE |
| 232843_s_at | TRUE |  | 232843_s_at | TRUE |
| 232869_at | TRUE |  | 232869_at | TRUE |
| 232904_at | TRUE |  | 232904_at | TRUE |
| 233030_at | TRUE |  | 233030_at | TRUE |
| 233035_at | TRUE |  | 233035_at | TRUE |
| 233050_at | TRUE |  | 233050_at | TRUE |
| 233064_at | TRUE |  | 233064_at | TRUE |
| 233128_at | TRUE |  | 233128_at | TRUE |
| 233171_at | TRUE |  | 233171_at | TRUE |
| 233220_at | TRUE |  | 233220_at | TRUE |
| 233239_at | TRUE |  | 233239_at | TRUE |
| 233295_at | TRUE |  | 233295_at | TRUE |
| 233298_at | TRUE |  | 233298_at | TRUE |
| 233310_at | TRUE |  | 233310_at | TRUE |
| 233337_s_at | TRUE |  | 233337_s_at | TRUE |
| 233357_at | TRUE |  | 233357_at | TRUE |
| 233406_at | TRUE |  | 233406_at | TRUE |
| 233409_at | TRUE |  | 233409_at | TRUE |
| 233433_at | TRUE |  | 233433_at | TRUE |
| 233437_at | TRUE |  | 233437_at | TRUE |
| 233487_s_at | TRUE |  | 233487_s_at | TRUE |
| 233510_s_at | TRUE |  | 233510_s_at | TRUE |
| 233528_s_at | TRUE |  | 233528_s_at | TRUE |
| 233563_s_at | TRUE |  | 233563_s_at | TRUE |
| 233581_at | TRUE |  | 233581_at | TRUE |
| 233587_s_at | TRUE |  | 233587_s_at | TRUE |
| 233602_at | TRUE |  | 233602_at | TRUE |
| 233613_x_at | TRUE |  | 233613_x_at | TRUE |
| 233625_x_at | TRUE |  | 233625_x_at | TRUE |
| 233688_at | TRUE |  | 233688_at | TRUE |
| 233713_at | TRUE |  | 233713_at | TRUE |
| 233786_at | TRUE |  | 233786_at | TRUE |
| 233814_at | TRUE |  | 233814_at | TRUE |
| 233895_at | TRUE |  | 233895_at | TRUE |
| 233917_s_at | TRUE |  | 233917_s_at | TRUE |
| 234170_at | TRUE |  | 234170_at | TRUE |
| 234268_at | TRUE |  | 234268_at | TRUE |
| 234340_at | TRUE |  | 234340_at | TRUE |
| 234409_at | TRUE |  | 234409_at | TRUE |
| 234660_s_at | TRUE |  | 234660_s_at | TRUE |
| 234672_s_at | TRUE |  | 234672_s_at | TRUE |
| 234710_s_at | TRUE |  | 234710_s_at | TRUE |
| 234859_at | TRUE |  | 234859_at | TRUE |
| 234871_at | TRUE |  | 234871_at | TRUE |
| 234880_x_at | TRUE |  | 234880_x_at | TRUE |
| 234974_at | TRUE |  | 234974_at | TRUE |
| 234976_x_at | TRUE |  | 234976_x_at | TRUE |
| 235018_at | TRUE |  | 235018_at | TRUE |
| 235031_at | TRUE |  | 235031_at | TRUE |
| 235058_at | TRUE |  | 235058_at | TRUE |
| 235070_at | TRUE |  | 235070_at | TRUE |
| 235077_at | TRUE |  | 235077_at | TRUE |
| 235085_at | TRUE |  | 235085_at | TRUE |
| 235111_at | TRUE |  | 235111_at | TRUE |
| 235113_at | TRUE |  | 235113_at | TRUE |
| 235134_at | TRUE |  | 235134_at | TRUE |
| 235166_at | TRUE |  | 235166_at | TRUE |
| 235224_s_at | TRUE |  | 235224_s_at | TRUE |
| 235225_at | TRUE |  | 235225_at | TRUE |
| 235227_at | TRUE |  | 235227_at | TRUE |
| 235230_at | TRUE |  | 235230_at | TRUE |
| 235256_s_at | TRUE |  | 235256_s_at | TRUE |
| 235275_at | TRUE |  | 235275_at | TRUE |
| 235296_at | TRUE |  | 235296_at | TRUE |
| 235306_at | TRUE |  | 235306_at | TRUE |
| 235319_at | TRUE |  | 235319_at | TRUE |
| 235326_at | TRUE |  | 235326_at | TRUE |
| 235343_at | TRUE |  | 235343_at | TRUE |
| 235355_at | TRUE |  | 235355_at | TRUE |
| 235359_at | TRUE |  | 235359_at | TRUE |
| 235363_at | TRUE |  | 235363_at | TRUE |
| 235384_at | TRUE |  | 235384_at | TRUE |
| 235388_at | TRUE |  | 235388_at | TRUE |
| 235409_at | TRUE |  | 235409_at | TRUE |
| 235412_at | TRUE |  | 235412_at | TRUE |
| 235414_at | TRUE |  | 235414_at | TRUE |
| 235452_at | TRUE |  | 235452_at | TRUE |
| 235458_at | TRUE |  | 235458_at | TRUE |
| 235459_at | TRUE |  | 235459_at | TRUE |
| 235468_at | TRUE |  | 235468_at | TRUE |
| 235475_at | TRUE |  | 235475_at | TRUE |
| 235492_at | TRUE |  | 235492_at | TRUE |
| 235506_at | TRUE |  | 235506_at | TRUE |
| 235559_at | TRUE |  | 235559_at | TRUE |
| 235573_at | TRUE |  | 235573_at | TRUE |
| 235642_at | TRUE |  | 235642_at | TRUE |
| 235664_at | TRUE |  | 235664_at | TRUE |
| 235702_at | TRUE |  | 235702_at | TRUE |
| 235735_at | TRUE |  | 235735_at | TRUE |
| 235742_at | TRUE |  | 235742_at | TRUE |
| 235758_at | TRUE |  | 235758_at | TRUE |
| 235802_at | TRUE |  | 235802_at | TRUE |
| 235819_at | TRUE |  | 235819_at | TRUE |
| 235843_at | TRUE |  | 235843_at | TRUE |
| 235868_at | TRUE |  | 235868_at | TRUE |
| 235880_at | TRUE |  | 235880_at | TRUE |
| 235885_at | TRUE |  | 235885_at | TRUE |
| 235949_at | TRUE |  | 235949_at | TRUE |
| 235957_at | TRUE |  | 235957_at | TRUE |
| 235960_at | TRUE |  | 235960_at | TRUE |
| 235961_at | TRUE |  | 235961_at | TRUE |
| 235963_at | TRUE |  | 235963_at | TRUE |
| 235969_at | TRUE |  | 235969_at | TRUE |
| 235989_at | TRUE |  | 235989_at | TRUE |
| 236042_at | TRUE |  | 236042_at | TRUE |
| 236076_at | TRUE |  | 236076_at | TRUE |
| 236079_at | TRUE |  | 236079_at | TRUE |
| 236127_at | TRUE |  | 236127_at | TRUE |
| 236151_at | TRUE |  | 236151_at | TRUE |
| 236156_at | TRUE |  | 236156_at | TRUE |
| 236166_at | TRUE |  | 236166_at | TRUE |
| 236180_at | TRUE |  | 236180_at | TRUE |
| 236204_at | TRUE |  | 236204_at | TRUE |
| 236215_at | TRUE |  | 236215_at | TRUE |
| 236247_at | TRUE |  | 236247_at | TRUE |
| 236297_at | TRUE |  | 236297_at | TRUE |
| 236308_at | TRUE |  | 236308_at | TRUE |
| 236313_at | TRUE |  | 236313_at | TRUE |
| 236329_at | TRUE |  | 236329_at | TRUE |
| 236330_at | TRUE |  | 236330_at | TRUE |
| 236333_at | TRUE |  | 236333_at | TRUE |
| 236343_at | TRUE |  | 236343_at | TRUE |
| 236366_at | TRUE |  | 236366_at | TRUE |
| 236385_at | TRUE |  | 236385_at | TRUE |
| 236391_at | TRUE |  | 236391_at | TRUE |
| 236401_at | TRUE |  | 236401_at | TRUE |
| 236428_at | TRUE |  | 236428_at | TRUE |
| 236584_at | TRUE |  | 236584_at | TRUE |
| 236592_at | TRUE |  | 236592_at | TRUE |
| 236638_at | TRUE |  | 236638_at | TRUE |
| 236646_at | TRUE |  | 236646_at | TRUE |
| 236677_at | TRUE |  | 236677_at | TRUE |
| 236739_at | TRUE |  | 236739_at | TRUE |
| 236847_at | TRUE |  | 236847_at | TRUE |
| 236901_at | TRUE |  | 236901_at | TRUE |
| 236935_at | TRUE |  | 236935_at | TRUE |
| 237007_at | TRUE |  | 237007_at | TRUE |
| 237032_x_at | TRUE |  | 237032_x_at | TRUE |
| 237131_at | TRUE |  | 237131_at | TRUE |
| 237158_s_at | TRUE |  | 237158_s_at | TRUE |
| 237177_at | TRUE |  | 237177_at | TRUE |
| 237203_at | TRUE |  | 237203_at | TRUE |
| 237223_at | TRUE |  | 237223_at | TRUE |
| 237304_at | TRUE |  | 237304_at | TRUE |
| 237322_at | TRUE |  | 237322_at | TRUE |
| 237324_s_at | TRUE |  | 237324_s_at | TRUE |
| 237442_at | TRUE |  | 237442_at | TRUE |
| 237450_at | TRUE |  | 237450_at | TRUE |
| 237598_at | TRUE |  | 237598_at | TRUE |
| 237602_at | TRUE |  | 237602_at | TRUE |
| 237714_at | TRUE |  | 237714_at | TRUE |
| 237823_at | TRUE |  | 237823_at | TRUE |
| 237828_at | TRUE |  | 237828_at | TRUE |
| 237908_at | TRUE |  | 237908_at | TRUE |
| 237968_at | TRUE |  | 237968_at | TRUE |
| 237973_at | TRUE |  | 237973_at | TRUE |
| 238013_at | TRUE |  | 238013_at | TRUE |
| 238063_at | TRUE |  | 238063_at | TRUE |
| 238126_at | TRUE |  | 238126_at | TRUE |
| 238135_at | TRUE |  | 238135_at | TRUE |
| 238151_at | TRUE |  | 238151_at | TRUE |
| 238178_at | TRUE |  | 238178_at | TRUE |
| 238194_at | TRUE |  | 238194_at | TRUE |
| 238206_at | TRUE |  | 238206_at | TRUE |
| 238332_at | TRUE |  | 238332_at | TRUE |
| 238365_s_at | TRUE |  | 238365_s_at | TRUE |
| 238427_at | TRUE |  | 238427_at | TRUE |
| 238432_at | TRUE |  | 238432_at | TRUE |
| 238436_s_at | TRUE |  | 238436_s_at | TRUE |
| 238437_at | TRUE |  | 238437_at | TRUE |
| 238444_at | TRUE |  | 238444_at | TRUE |
| 238453_at | TRUE |  | 238453_at | TRUE |
| 238490_at | TRUE |  | 238490_at | TRUE |
| 238573_at | TRUE |  | 238573_at | TRUE |
| 238623_at | TRUE |  | 238623_at | TRUE |
| 238661_at | TRUE |  | 238661_at | TRUE |
| 238668_at | TRUE |  | 238668_at | TRUE |
| 238669_at | TRUE |  | 238669_at | TRUE |
| 238752_at | TRUE |  | 238752_at | TRUE |
| 238780_s_at | TRUE |  | 238780_s_at | TRUE |
| 238781_at | TRUE |  | 238781_at | TRUE |
| 238786_at | TRUE |  | 238786_at | TRUE |
| 238789_at | TRUE |  | 238789_at | TRUE |
| 238850_at | TRUE |  | 238850_at | TRUE |
| 238924_at | TRUE |  | 238924_at | TRUE |
| 238966_at | TRUE |  | 238966_at | TRUE |
| 238975_at | TRUE |  | 238975_at | TRUE |
| 239010_at | TRUE |  | 239010_at | TRUE |
| 239021_at | TRUE |  | 239021_at | TRUE |
| 239024_at | TRUE |  | 239024_at | TRUE |
| 239027_at | TRUE |  | 239027_at | TRUE |
| 239031_at | TRUE |  | 239031_at | TRUE |
| 239035_at | TRUE |  | 239035_at | TRUE |
| 239168_at | TRUE |  | 239168_at | TRUE |
| 239229_at | TRUE |  | 239229_at | TRUE |
| 239250_at | TRUE |  | 239250_at | TRUE |
| 239275_at | TRUE |  | 239275_at | TRUE |
| 239283_at | TRUE |  | 239283_at | TRUE |
| 239290_at | TRUE |  | 239290_at | TRUE |
| 239293_at | TRUE |  | 239293_at | TRUE |
| 239294_at | TRUE |  | 239294_at | TRUE |
| 239347_at | TRUE |  | 239347_at | TRUE |
| 239354_at | TRUE |  | 239354_at | TRUE |
| 239357_at | TRUE |  | 239357_at | TRUE |
| 239358_at | TRUE |  | 239358_at | TRUE |
| 239359_at | TRUE |  | 239359_at | TRUE |
| 239390_at | TRUE |  | 239390_at | TRUE |
| 239406_at | TRUE |  | 239406_at | TRUE |
| 239407_at | TRUE |  | 239407_at | TRUE |
| 239448_at | TRUE |  | 239448_at | TRUE |
| 239525_at | TRUE |  | 239525_at | TRUE |
| 239537_at | TRUE |  | 239537_at | TRUE |
| 239577_at | TRUE |  | 239577_at | TRUE |
| 239606_at | TRUE |  | 239606_at | TRUE |
| 239671_at | TRUE |  | 239671_at | TRUE |
| 239677_at | TRUE |  | 239677_at | TRUE |
| 239678_at | TRUE |  | 239678_at | TRUE |
| 239682_at | TRUE |  | 239682_at | TRUE |
| 239726_at | TRUE |  | 239726_at | TRUE |
| 239729_at | TRUE |  | 239729_at | TRUE |
| 239738_at | TRUE |  | 239738_at | TRUE |
| 239760_at | TRUE |  | 239760_at | TRUE |
| 239765_at | TRUE |  | 239765_at | TRUE |
| 239824_s_at | TRUE |  | 239824_s_at | TRUE |
| 239840_at | TRUE |  | 239840_at | TRUE |
| 239864_at | TRUE |  | 239864_at | TRUE |
| 239888_at | TRUE |  | 239888_at | TRUE |
| 239897_at | TRUE |  | 239897_at | TRUE |
| 239903_at | TRUE |  | 239903_at | TRUE |
| 239992_at | TRUE |  | 239992_at | TRUE |
| 240011_at | TRUE |  | 240011_at | TRUE |
| 240041_at | TRUE |  | 240041_at | TRUE |
| 240064_at | TRUE |  | 240064_at | TRUE |
| 240089_at | TRUE |  | 240089_at | TRUE |
| 240131_at | TRUE |  | 240131_at | TRUE |
| 240152_at | TRUE |  | 240152_at | TRUE |
| 240211_at | TRUE |  | 240211_at | TRUE |
| 240236_at | TRUE |  | 240236_at | TRUE |
| 240240_at | TRUE |  | 240240_at | TRUE |
| 240405_at | TRUE |  | 240405_at | TRUE |
| 240448_at | TRUE |  | 240448_at | TRUE |
| 240455_at | TRUE |  | 240455_at | TRUE |
| 240503_at | TRUE |  | 240503_at | TRUE |
| 240574_at | TRUE |  | 240574_at | TRUE |
| 240614_at | TRUE |  | 240614_at | TRUE |
| 240703_s_at | TRUE |  | 240703_s_at | TRUE |
| 240709_at | TRUE |  | 240709_at | TRUE |
| 240830_at | TRUE |  | 240830_at | TRUE |
| 240841_at | TRUE |  | 240841_at | TRUE |
| 240908_at | TRUE |  | 240908_at | TRUE |
| 241068_at | TRUE |  | 241068_at | TRUE |
| 241188_at | TRUE |  | 241188_at | TRUE |
| 241365_at | TRUE |  | 241365_at | TRUE |
| 241382_at | TRUE |  | 241382_at | TRUE |
| 241389_at | TRUE |  | 241389_at | TRUE |
| 241393_at | TRUE |  | 241393_at | TRUE |
| 241398_at | TRUE |  | 241398_at | TRUE |
| 241434_at | TRUE |  | 241434_at | TRUE |
| 241567_at | TRUE |  | 241567_at | TRUE |
| 241627_x_at | TRUE |  | 241627_x_at | TRUE |
| 241729_at | TRUE |  | 241729_at | TRUE |
| 241730_at | TRUE |  | 241730_at | TRUE |
| 241741_at | TRUE |  | 241741_at | TRUE |
| 241742_at | TRUE |  | 241742_at | TRUE |
| 241808_at | TRUE |  | 241808_at | TRUE |
| 241812_at | TRUE |  | 241812_at | TRUE |
| 241844_x_at | TRUE |  | 241844_x_at | TRUE |
| 241871_at | TRUE |  | 241871_at | TRUE |
| 241891_at | TRUE |  | 241891_at | TRUE |
| 241946_at | TRUE |  | 241946_at | TRUE |
| 241947_at | TRUE |  | 241947_at | TRUE |
| 241966_at | TRUE |  | 241966_at | TRUE |
| 241986_at | TRUE |  | 241986_at | TRUE |
| 241991_at | TRUE |  | 241991_at | TRUE |
| 241999_at | TRUE |  | 241999_at | TRUE |
| 242082_at | TRUE |  | 242082_at | TRUE |
| 242088_at | TRUE |  | 242088_at | TRUE |
| 242091_at | TRUE |  | 242091_at | TRUE |
| 242123_at | TRUE |  | 242123_at | TRUE |
| 242193_at | TRUE |  | 242193_at | TRUE |
| 242285_at | TRUE |  | 242285_at | TRUE |
| 242344_at | TRUE |  | 242344_at | TRUE |
| 242399_at | TRUE |  | 242399_at | TRUE |
| 242422_at | TRUE |  | 242422_at | TRUE |
| 242451_x_at | TRUE |  | 242451_x_at | TRUE |
| 242463_x_at | TRUE |  | 242463_x_at | TRUE |
| 242487_at | TRUE |  | 242487_at | TRUE |
| 242499_at | TRUE |  | 242499_at | TRUE |
| 242523_at | TRUE |  | 242523_at | TRUE |
| 242538_at | TRUE |  | 242538_at | TRUE |
| 242592_at | TRUE |  | 242592_at | TRUE |
| 242599_at | TRUE |  | 242599_at | TRUE |
| 242600_at | TRUE |  | 242600_at | TRUE |
| 242611_at | TRUE |  | 242611_at | TRUE |
| 242618_at | TRUE |  | 242618_at | TRUE |
| 242626_at | TRUE |  | 242626_at | TRUE |
| 242640_at | TRUE |  | 242640_at | TRUE |
| 242761_s_at | TRUE |  | 242761_s_at | TRUE |
| 242794_at | TRUE |  | 242794_at | TRUE |
| 242852_at | TRUE |  | 242852_at | TRUE |
| 242870_at | TRUE |  | 242870_at | TRUE |
| 242907_at | TRUE |  | 242907_at | TRUE |
| 242931_at | TRUE |  | 242931_at | TRUE |
| 242950_x_at | TRUE |  | 242950_x_at | TRUE |
| 242953_at | TRUE |  | 242953_at | TRUE |
| 242965_at | TRUE |  | 242965_at | TRUE |
| 243027_at | TRUE |  | 243027_at | TRUE |
| 243036_at | TRUE |  | 243036_at | TRUE |
| 243061_at | TRUE |  | 243061_at | TRUE |
| 243099_at | TRUE |  | 243099_at | TRUE |
| 243208_x_at | TRUE |  | 243208_x_at | TRUE |
| 243219_x_at | TRUE |  | 243219_x_at | TRUE |
| 243319_at | TRUE |  | 243319_at | TRUE |
| 243426_at | TRUE |  | 243426_at | TRUE |
| 243430_at | TRUE |  | 243430_at | TRUE |
| 243504_at | TRUE |  | 243504_at | TRUE |
| 243542_at | TRUE |  | 243542_at | TRUE |
| 243543_at | TRUE |  | 243543_at | TRUE |
| 243549_at | TRUE |  | 243549_at | TRUE |
| 243550_at | TRUE |  | 243550_at | TRUE |
| 243624_at | TRUE |  | 243624_at | TRUE |
| 243658_at | TRUE |  | 243658_at | TRUE |
| 243665_s_at | TRUE |  | 243665_s_at | TRUE |
| 243666_at | TRUE |  | 243666_at | TRUE |
| 243681_at | TRUE |  | 243681_at | TRUE |
| 243689_s_at | TRUE |  | 243689_s_at | TRUE |
| 243747_at | TRUE |  | 243747_at | TRUE |
| 243770_at | TRUE |  | 243770_at | TRUE |
| 243813_at | TRUE |  | 243813_at | TRUE |
| 243821_at | TRUE |  | 243821_at | TRUE |
| 243836_at | TRUE |  | 243836_at | TRUE |
| 243850_at | TRUE |  | 243850_at | TRUE |
| 243872_at | TRUE |  | 243872_at | TRUE |
| 243881_at | TRUE |  | 243881_at | TRUE |
| 243929_at | TRUE |  | 243929_at | TRUE |
| 243931_at | TRUE |  | 243931_at | TRUE |
| 243945_at | TRUE |  | 243945_at | TRUE |
| 243967_at | TRUE |  | 243967_at | TRUE |
| 243998_at | TRUE |  | 243998_at | TRUE |
| 244001_at | TRUE |  | 244001_at | TRUE |
| 244029_at | TRUE |  | 244029_at | TRUE |
| 244050_at | TRUE |  | 244050_at | TRUE |
| 244071_at | TRUE |  | 244071_at | TRUE |
| 244099_at | TRUE |  | 244099_at | TRUE |
| 244111_at | TRUE |  | 244111_at | TRUE |
| 244118_at | TRUE |  | 244118_at | TRUE |
| 244130_at | TRUE |  | 244130_at | TRUE |
| 244170_at | TRUE |  | 244170_at | TRUE |
| 244184_at | TRUE |  | 244184_at | TRUE |
| 244227_at | TRUE |  | 244227_at | TRUE |
| 244251_at | TRUE |  | 244251_at | TRUE |
| 244259_s_at | TRUE |  | 244259_s_at | TRUE |
| 244352_at | TRUE |  | 244352_at | TRUE |
| 244360_at | TRUE |  | 244360_at | TRUE |
| 244382_at | TRUE |  | 244382_at | TRUE |
| 244398_x_at | TRUE |  | 244398_x_at | TRUE |
| 244418_at | TRUE |  | 244418_at | TRUE |
| 244609_at | TRUE |  | 244609_at | TRUE |
| 244650_at | TRUE |  | 244650_at | TRUE |
| 244662_at | TRUE |  | 244662_at | TRUE |
| 244664_at | TRUE |  | 244664_at | TRUE |
| 244708_at | TRUE |  | 244708_at | TRUE |
| 244724_at | TRUE |  | 244724_at | TRUE |
| 244764_at | TRUE |  | 244764_at | TRUE |
| 244795_at | TRUE |  | 244795_at | TRUE |
| 31845_at | TRUE |  | 31845_at | TRUE |
| 33760_at | TRUE |  | 33760_at | TRUE |
| 33767_at | TRUE |  | 33767_at | TRUE |
| 33778_at | TRUE |  | 33778_at | TRUE |
| 34187_at | TRUE |  | 34187_at | TRUE |
| 36564_at | TRUE |  | 36564_at | TRUE |
| 37012_at | TRUE |  | 37012_at | TRUE |
| 37549_g_at | TRUE |  | 37549_g_at | TRUE |
| 37566_at | TRUE |  | 37566_at | TRUE |
| 37892_at | TRUE |  | 37892_at | TRUE |
| 37996_s_at | TRUE |  | 37996_s_at | TRUE |
| 38149_at | TRUE |  | 38149_at | TRUE |
| 38269_at | TRUE |  | 38269_at | TRUE |
| 39891_at | TRUE |  | 39891_at | TRUE |
| 40020_at | TRUE |  | 40020_at | TRUE |
| 44040_at | TRUE |  | 44040_at | TRUE |
| 44783_s_at | TRUE |  | 44783_s_at | TRUE |
| 46167_at | TRUE |  | 46167_at | TRUE |
| 48659_at | TRUE |  | 48659_at | TRUE |
| 51146_at | TRUE |  | 51146_at | TRUE |
| 52078_at | TRUE |  | 52078_at | TRUE |
| 57588_at | TRUE |  | 57588_at | TRUE |
| 64408_s_at | TRUE |  | 64408_s_at | TRUE |
| 81737_at | TRUE |  | 81737_at | TRUE |
| 89977_at | TRUE |  | 89977_at | TRUE |
| 90610_at | TRUE |  | 90610_at | TRUE |
| 1552641_s_at | FALSE |  | 1552553_a_at | FALSE |
| 1552790_a_at | FALSE |  | 1552675_at | FALSE |
| 1553972_a_at | FALSE |  | 1552691_at | FALSE |
| 1554538_at | FALSE |  | 1552708_a_at | FALSE |
| 1555469_a_at | FALSE |  | 1552806_a_at | FALSE |
| 1555858_at | FALSE |  | 1552849_at | FALSE |
| 1556969_at | FALSE |  | 1552931_a_at | FALSE |
| 1558010_s_at | FALSE |  | 1553264_a_at | FALSE |
| 1559675_at | FALSE |  | 1553311_at | FALSE |
| 1562745_at | FALSE |  | 1553865_a_at | FALSE |
| 200006_at | FALSE |  | 1554785_at | FALSE |
| 200022_at | FALSE |  | 1554922_at | FALSE |
| 200662_s_at | FALSE |  | 1555019_at | FALSE |
| 200811_at | FALSE |  | 1555800_at | FALSE |
| 200816_s_at | FALSE |  | 1555938_x_at | FALSE |
| 200871_s_at | FALSE |  | 1556095_at | FALSE |
| 200918_s_at | FALSE |  | 1556261_a_at | FALSE |
| 201166_s_at | FALSE |  | 1556653_at | FALSE |
| 201295_s_at | FALSE |  | 1556704_s_at | FALSE |
| 201457_x_at | FALSE |  | 1556935_at | FALSE |
| 201742_x_at | FALSE |  | 1557430_at | FALSE |
| 201819_at | FALSE |  | 1558636_s_at | FALSE |
| 201976_s_at | FALSE |  | 1558887_at | FALSE |
| 202105_at | FALSE |  | 1558959_at | FALSE |
| 202292_x_at | FALSE |  | 1561116_at | FALSE |
| 202649_x_at | FALSE |  | 1562326_at | FALSE |
| 203057_s_at | FALSE |  | 1563110_at | FALSE |
| 203611_at | FALSE |  | 1568851_at | FALSE |
| 203884_s_at | FALSE |  | 1568900_a_at | FALSE |
| 204116_at | FALSE |  | 1569004_at | FALSE |
| 204300_at | FALSE |  | 1569072_s_at | FALSE |
| 204850_s_at | FALSE |  | 1569673_at | FALSE |
| 204993_at | FALSE |  | 1569830_at | FALSE |
| 205123_s_at | FALSE |  | 1569969_a_at | FALSE |
| 205830_at | FALSE |  | 1570394_at | FALSE |
| 207152_at | FALSE |  | 1570511_at | FALSE |
| 207727_s_at | FALSE |  | 201319_at | FALSE |
| 207981_s_at | FALSE |  | 201422_at | FALSE |
| 208068_x_at | FALSE |  | 201631_s_at | FALSE |
| 208110_x_at | FALSE |  | 201798_s_at | FALSE |
| 208352_x_at | FALSE |  | 202134_s_at | FALSE |
| 208512_s_at | FALSE |  | 202376_at | FALSE |
| 208627_s_at | FALSE |  | 202638_s_at | FALSE |
| 208662_s_at | FALSE |  | 202663_at | FALSE |
| 208897_s_at | FALSE |  | 202998_s_at | FALSE |
| 209118_s_at | FALSE |  | 203424_s_at | FALSE |
| 209215_at | FALSE |  | 204051_s_at | FALSE |
| 209218_at | FALSE |  | 204150_at | FALSE |
| 209470_s_at | FALSE |  | 204174_at | FALSE |
| 209650_s_at | FALSE |  | 204675_at | FALSE |
| 210275_s_at | FALSE |  | 204929_s_at | FALSE |
| 210570_x_at | FALSE |  | 204967_at | FALSE |
| 210605_s_at | FALSE |  | 205117_at | FALSE |
| 211047_x_at | FALSE |  | 205454_at | FALSE |
| 211217_s_at | FALSE |  | 205747_at | FALSE |
| 211755_s_at | FALSE |  | 206274_s_at | FALSE |
| 211935_at | FALSE |  | 206356_s_at | FALSE |
| 211965_at | FALSE |  | 206648_at | FALSE |
| 212032_s_at | FALSE |  | 206715_at | FALSE |
| 212069_s_at | FALSE |  | 207454_at | FALSE |
| 212155_at | FALSE |  | 207855_s_at | FALSE |
| 212162_at | FALSE |  | 208296_x_at | FALSE |
| 212461_at | FALSE |  | 208579_x_at | FALSE |
| 212600_s_at | FALSE |  | 208711_s_at | FALSE |
| 213486_at | FALSE |  | 209188_x_at | FALSE |
| 213601_at | FALSE |  | 209398_at | FALSE |
| 213787_s_at | FALSE |  | 209448_at | FALSE |
| 214948_s_at | FALSE |  | 209604_s_at | FALSE |
| 215119_at | FALSE |  | 209685_s_at | FALSE |
| 215568_x_at | FALSE |  | 209704_at | FALSE |
| 215649_s_at | FALSE |  | 209732_at | FALSE |
| 215873_x_at | FALSE |  | 209955_s_at | FALSE |
| 216120_s_at | FALSE |  | 210089_s_at | FALSE |
| 216382_s_at | FALSE |  | 210092_at | FALSE |
| 216606_x_at | FALSE |  | 210315_at | FALSE |
| 217963_s_at | FALSE |  | 210363_s_at | FALSE |
| 218223_s_at | FALSE |  | 210654_at | FALSE |
| 218289_s_at | FALSE |  | 210989_at | FALSE |
| 218381_s_at | FALSE |  | 210991_s_at | FALSE |
| 218754_at | FALSE |  | 211241_at | FALSE |
| 218809_at | FALSE |  | 211824_x_at | FALSE |
| 218956_s_at | FALSE |  | 212353_at | FALSE |
| 219093_at | FALSE |  | 212354_at | FALSE |
| 219608_s_at | FALSE |  | 212473_s_at | FALSE |
| 220642_x_at | FALSE |  | 213054_at | FALSE |
| 220688_s_at | FALSE |  | 213060_s_at | FALSE |
| 221041_s_at | FALSE |  | 213094_at | FALSE |
| 221864_at | FALSE |  | 213200_at | FALSE |
| 222473_s_at | FALSE |  | 213277_at | FALSE |
| 222624_s_at | FALSE |  | 213537_at | FALSE |
| 222736_s_at | FALSE |  | 213568_at | FALSE |
| 223068_at | FALSE |  | 213693_s_at | FALSE |
| 223118_s_at | FALSE |  | 213975_s_at | FALSE |
| 223148_at | FALSE |  | 214230_at | FALSE |
| 223194_s_at | FALSE |  | 214467_at | FALSE |
| 223222_at | FALSE |  | 214622_at | FALSE |
| 223298_s_at | FALSE |  | 215523_at | FALSE |
| 223389_s_at | FALSE |  | 215539_at | FALSE |
| 223482_at | FALSE |  | 215779_s_at | FALSE |
| 223531_x_at | FALSE |  | 215803_at | FALSE |
| 224579_at | FALSE |  | 216016_at | FALSE |
| 224584_at | FALSE |  | 216565_x_at | FALSE |
| 224838_at | FALSE |  | 217995_at | FALSE |
| 224885_s_at | FALSE |  | 218368_s_at | FALSE |
| 224888_at | FALSE |  | 218424_s_at | FALSE |
| 225090_at | FALSE |  | 218854_at | FALSE |
| 225138_at | FALSE |  | 218923_at | FALSE |
| 225358_at | FALSE |  | 219256_s_at | FALSE |
| 225541_at | FALSE |  | 219513_s_at | FALSE |
| 225556_at | FALSE |  | 219659_at | FALSE |
| 225598_at | FALSE |  | 220025_at | FALSE |
| 225619_at | FALSE |  | 220157_x_at | FALSE |
| 225693_s_at | FALSE |  | 220166_at | FALSE |
| 225805_at | FALSE |  | 220169_at | FALSE |
| 226022_at | FALSE |  | 220294_at | FALSE |
| 226130_at | FALSE |  | 220358_at | FALSE |
| 226143_at | FALSE |  | 221859_at | FALSE |
| 226327_at | FALSE |  | 221900_at | FALSE |
| 226649_at | FALSE |  | 221973_at | FALSE |
| 226751_at | FALSE |  | 222322_at | FALSE |
| 227052_at | FALSE |  | 222858_s_at | FALSE |
| 227933_at | FALSE |  | 222959_at | FALSE |
| 228260_at | FALSE |  | 223217_s_at | FALSE |
| 228422_at | FALSE |  | 223592_s_at | FALSE |
| 228547_at | FALSE |  | 223653_x_at | FALSE |
| 228771_at | FALSE |  | 225815_at | FALSE |
| 228821_at | FALSE |  | 226057_at | FALSE |
| 228955_at | FALSE |  | 226074_at | FALSE |
| 229563_s_at | FALSE |  | 226507_at | FALSE |
| 229757_at | FALSE |  | 226743_at | FALSE |
| 229844_at | FALSE |  | 226906_s_at | FALSE |
| 230320_at | FALSE |  | 227125_at | FALSE |
| 230426_at | FALSE |  | 227139_s_at | FALSE |
| 230692_at | FALSE |  | 228071_at | FALSE |
| 231164_at | FALSE |  | 228132_at | FALSE |
| 233141_s_at | FALSE |  | 228295_at | FALSE |
| 234665_x_at | FALSE |  | 228438_at | FALSE |
| 235017_s_at | FALSE |  | 228795_at | FALSE |
| 235024_at | FALSE |  | 228944_at | FALSE |
| 235118_at | FALSE |  | 229039_at | FALSE |
| 235167_at | FALSE |  | 229723_at | FALSE |
| 235324_at | FALSE |  | 230261_at | FALSE |
| 236059_at | FALSE |  | 230550_at | FALSE |
| 236259_at | FALSE |  | 230816_at | FALSE |
| 237033_at | FALSE |  | 231170_at | FALSE |
| 237109_at | FALSE |  | 231783_at | FALSE |
| 242206_at | FALSE |  | 232056_at | FALSE |
| 242393_x_at | FALSE |  | 232231_at | FALSE |
| 243052_at | FALSE |  | 232724_at | FALSE |
| 243937_x_at | FALSE |  | 233452_at | FALSE |
| 38398_at | FALSE |  | 233499_at | FALSE |
| 38964_r_at | FALSE |  | 233502_at | FALSE |
| 44702_at | FALSE |  | 233904_at | FALSE |
| 58367_s_at | FALSE |  | 234298_at | FALSE |
| AFFX-LysX-5_at | FALSE |  | 235844_at | FALSE |
| AFFX-r2-Bs-lys-M_at | FALSE |  | 235856_at | FALSE |
|  |  |  | 236532_at | FALSE |
|  |  |  | 236583_at | FALSE |
|  |  |  | 236738_at | FALSE |
|  |  |  | 237470_at | FALSE |
|  |  |  | 237623_at | FALSE |
|  |  |  | 238409_x_at | FALSE |
|  |  |  | 238653_at | FALSE |
|  |  |  | 238734_at | FALSE |
|  |  |  | 240673_at | FALSE |
|  |  |  | 240861_at | FALSE |
|  |  |  | 241292_at | FALSE |
|  |  |  | 241583_x_at | FALSE |
|  |  |  | 241763_s_at | FALSE |
|  |  |  | 242086_at | FALSE |
|  |  |  | 242521_at | FALSE |
|  |  |  | 242609_x_at | FALSE |
|  |  |  | 242628_at | FALSE |
|  |  |  | 242653_at | FALSE |
|  |  |  | 242714_at | FALSE |
|  |  |  | 243871_at | FALSE |
|  |  |  | 243994_at | FALSE |
|  |  |  | 244023_at | FALSE |
|  |  |  | 244061_at | FALSE |
|  |  |  | 244435_at | FALSE |
|  |  |  | 38487_at | FALSE |

Table S7

| **DAVID Functional Category Enrichment Analysis - Genes identified by SAM  (Benjamini-Hochberg corrected, FDR=0.05)** | | | |
| --- | --- | --- | --- |
| **Parent** | | **Transformed** | |
| **GO / KEGG Term** | **In Both?** | **GO / KEGG Term** | **In Both?** |
| GO:0001775~cell activation | TRUE | GO:0001775~cell activation | TRUE |
| GO:0001817~regulation of cytokine production | TRUE | GO:0001817~regulation of cytokine production | TRUE |
| GO:0002250~adaptive immune response | TRUE | GO:0002250~adaptive immune response | TRUE |
| GO:0002252~immune effector process | TRUE | GO:0002252~immune effector process | TRUE |
| GO:0002253~activation of immune response | TRUE | GO:0002253~activation of immune response | TRUE |
| GO:0002443~leukocyte mediated immunity | TRUE | GO:0002443~leukocyte mediated immunity | TRUE |
| GO:0002449~lymphocyte mediated immunity | TRUE | GO:0002449~lymphocyte mediated immunity | TRUE |
| GO:0002460~adaptive immune response based on somatic recombination of immune receptors built from immunoglobulin superfamily domains | TRUE | GO:0002460~adaptive immune response based on somatic recombination of immune receptors built from immunoglobulin superfamily domains | TRUE |
| GO:0002684~positive regulation of immune system process | TRUE | GO:0002684~positive regulation of immune system process | TRUE |
| GO:0005886~plasma membrane | TRUE | GO:0005886~plasma membrane | TRUE |
| GO:0005887~integral to plasma membrane | TRUE | GO:0005887~integral to plasma membrane | TRUE |
| GO:0006952~defense response | TRUE | GO:0006952~defense response | TRUE |
| GO:0006954~inflammatory response | TRUE | GO:0006954~inflammatory response | TRUE |
| GO:0006955~immune response | TRUE | GO:0006955~immune response | TRUE |
| GO:0016064~immunoglobulin mediated immune response | TRUE | GO:0016064~immunoglobulin mediated immune response | TRUE |
| GO:0019724~B cell mediated immunity | TRUE | GO:0019724~B cell mediated immunity | TRUE |
| GO:0031226~intrinsic to plasma membrane | TRUE | GO:0031226~intrinsic to plasma membrane | TRUE |
| GO:0045087~innate immune response | TRUE | GO:0045087~innate immune response | TRUE |
| GO:0045321~leukocyte activation | TRUE | GO:0045321~leukocyte activation | TRUE |
| GO:0046649~lymphocyte activation | TRUE | GO:0046649~lymphocyte activation | TRUE |
| GO:0048584~positive regulation of response to stimulus | TRUE | GO:0048584~positive regulation of response to stimulus | TRUE |
| GO:0050778~positive regulation of immune response | TRUE | GO:0050778~positive regulation of immune response | TRUE |
| immune response | TRUE | immune response | TRUE |
| membrane | TRUE | membrane | TRUE |
| phosphoprotein | TRUE | phosphoprotein | TRUE |
| topological domain:Cytoplasmic | TRUE | topological domain:Cytoplasmic | TRUE |
| transmembrane | TRUE | transmembrane | TRUE |
| transmembrane protein | TRUE | transmembrane protein | TRUE |
| GO:0002504~antigen processing and presentation of peptide or polysaccharide antigen via MHC class II | FALSE | GO:0001819~positive regulation of cytokine production | FALSE |
| GO:0007242~intracellular signaling cascade | FALSE | GO:0006959~humoral immune response | FALSE |
| GO:0030097~hemopoiesis | FALSE | GO:0009611~response to wounding | FALSE |
| GO:0042611~MHC protein complex | FALSE | GO:0019955~cytokine binding | FALSE |
| GO:0043235~receptor complex | FALSE | GO:0042110~T cell activation | FALSE |
| GO:0044459~plasma membrane part | FALSE | innate immunity | FALSE |
| GO:0045621~positive regulation of lymphocyte differentiation | FALSE | transmembrane region | FALSE |
| GO:0050867~positive regulation of cell activation | FALSE |  |  |
| GO:0051251~positive regulation of lymphocyte activation | FALSE |  |  |
| domain:Ig-like C1-type | FALSE |  |  |
| heterodimer | FALSE |  |  |

Table S8

| **DAVID Functional Category Enrichment Analysis - Genes identified by Kruskal-Wallis  (Benjamini-Hochberg corrected, FDR=0.05)** | | | |
| --- | --- | --- | --- |
| **Parent** | | **Transformed** | |
| **GO / KEGG Term** | **In Both?** | **GO / KEGG Term** | **In Both?** |
| alternative splicing | TRUE | alternative splicing | TRUE |
| cytoplasm | TRUE | cytoplasm | TRUE |
| domain:Ig-like C1-type | TRUE | domain:Ig-like C1-type | TRUE |
| GO:0001775~cell activation | TRUE | GO:0001775~cell activation | TRUE |
| GO:0002250~adaptive immune response | TRUE | GO:0002250~adaptive immune response | TRUE |
| GO:0002252~immune effector process | TRUE | GO:0002252~immune effector process | TRUE |
| GO:0002443~leukocyte mediated immunity | TRUE | GO:0002443~leukocyte mediated immunity | TRUE |
| GO:0002460~adaptive immune response based on somatic recombination of immune receptors built from immunoglobulin superfamily domains | TRUE | GO:0002460~adaptive immune response based on somatic recombination of immune receptors built from immunoglobulin superfamily domains | TRUE |
| GO:0002504~antigen processing and presentation of peptide or polysaccharide antigen via MHC class II | TRUE | GO:0002504~antigen processing and presentation of peptide or polysaccharide antigen via MHC class II | TRUE |
| GO:0002521~leukocyte differentiation | TRUE | GO:0002521~leukocyte differentiation | TRUE |
| GO:0002684~positive regulation of immune system process | TRUE | GO:0002684~positive regulation of immune system process | TRUE |
| GO:0006952~defense response | TRUE | GO:0006952~defense response | TRUE |
| GO:0006954~inflammatory response | TRUE | GO:0006954~inflammatory response | TRUE |
| GO:0006955~immune response | TRUE | GO:0006955~immune response | TRUE |
| GO:0006959~humoral immune response | TRUE | GO:0006959~humoral immune response | TRUE |
| GO:0007242~intracellular signaling cascade | TRUE | GO:0007242~intracellular signaling cascade | TRUE |
| GO:0009611~response to wounding | TRUE | GO:0009611~response to wounding | TRUE |
| GO:0019882~antigen processing and presentation | TRUE | GO:0019882~antigen processing and presentation | TRUE |
| GO:0019955~cytokine binding | TRUE | GO:0019955~cytokine binding | TRUE |
| GO:0030097~hemopoiesis | TRUE | GO:0030097~hemopoiesis | TRUE |
| GO:0032395~MHC class II receptor activity | TRUE | GO:0032395~MHC class II receptor activity | TRUE |
| GO:0042110~T cell activation | TRUE | GO:0042110~T cell activation | TRUE |
| GO:0042611~MHC protein complex | TRUE | GO:0042611~MHC protein complex | TRUE |
| GO:0043235~receptor complex | TRUE | GO:0043235~receptor complex | TRUE |
| GO:0044459~plasma membrane part | TRUE | GO:0044459~plasma membrane part | TRUE |
| GO:0045321~leukocyte activation | TRUE | GO:0045321~leukocyte activation | TRUE |
| GO:0045582~positive regulation of T cell differentiation | TRUE | GO:0045582~positive regulation of T cell differentiation | TRUE |
| GO:0045621~positive regulation of lymphocyte differentiation | TRUE | GO:0045621~positive regulation of lymphocyte differentiation | TRUE |
| GO:0046649~lymphocyte activation | TRUE | GO:0046649~lymphocyte activation | TRUE |
| GO:0046983~protein dimerization activity | TRUE | GO:0046983~protein dimerization activity | TRUE |
| GO:0048002~antigen processing and presentation of peptide antigen | TRUE | GO:0048002~antigen processing and presentation of peptide antigen | TRUE |
| GO:0048534~hemopoietic or lymphoid organ development | TRUE | GO:0048534~hemopoietic or lymphoid organ development | TRUE |
| GO:0048584~positive regulation of response to stimulus | TRUE | GO:0048584~positive regulation of response to stimulus | TRUE |
| GO:0050778~positive regulation of immune response | TRUE | GO:0050778~positive regulation of immune response | TRUE |
| GO:0050865~regulation of cell activation | TRUE | GO:0050865~regulation of cell activation | TRUE |
| heterodimer | TRUE | heterodimer | TRUE |
| immune response | TRUE | immune response | TRUE |
| IPR003597:Immunoglobulin C1-set | TRUE | IPR003597:Immunoglobulin C1-set | TRUE |
| membrane | TRUE | membrane | TRUE |
| mhc ii | TRUE | mhc ii | TRUE |
| mutagenesis site | TRUE | mutagenesis site | TRUE |
| phosphoprotein | TRUE | phosphoprotein | TRUE |
| region of interest:Alpha-1 | TRUE | region of interest:Alpha-1 | TRUE |
| region of interest:Alpha-2 | TRUE | region of interest:Alpha-2 | TRUE |
| region of interest:Connecting peptide | TRUE | region of interest:Connecting peptide | TRUE |
| SM00407:IGc1 | TRUE | SM00407:IGc1 | TRUE |
| splice variant | TRUE | splice variant | TRUE |
| transmembrane | TRUE | transmembrane | TRUE |
| transmembrane protein | TRUE | transmembrane protein | TRUE |

Table S9

| Source    Platform | **Normal** | **Tumor** |
| --- | --- | --- |
|  | **U133A Plus 2.0** | **U133A Plus 2.0** |
| Brain | GEO GSM176049 | GEO GSE4290 |
| Breast | GEO GSM175792 | GEO GSE21653 |
| Colon | GEO GSM175905 | GEO GSE14333 |
| Gastric | GEO GSM175943 | GEO GSE15460 |
| Ovarian | GEO GSM176131 | GEO GSE9899 |
|  |  |  |
| GEO indicates data is taken from the Gene Expression Omnibus | | |
